# A genome-wide association study identifies risk loci for spirometric measures among smokers of European and African ancestry

**DOI:** 10.1186/s12863-015-0299-4

**Published:** 2015-12-03

**Authors:** Sharon M. Lutz, Michael H. Cho, Kendra Young, Craig P. Hersh, Peter J. Castaldi, Merry-Lynn McDonald, Elizabeth Regan, Manuel Mattheisen, Dawn L. DeMeo, Margaret Parker, Marilyn Foreman, Barry J. Make, Robert L. Jensen, Richard Casaburi, David A. Lomas, Surya P. Bhatt, Per Bakke, Amund Gulsvik, James D. Crapo, Terri H. Beaty, Nan M. Laird, Christoph Lange, John E. Hokanson, Edwin K. Silverman, ECLIPSE Investigators, and COPDGene Investigators

**Affiliations:** Department of Biostatistics, University of Colorado Anschutz Medical Campus, 13001 E. 17th Place, B119 Bldg. 500, W3128, Aurora, CO 80045 USA; Channing Division of Network Medicine, Brigham and Women’s Hospital, Harvard Medical School, Boston, MA USA; Department of Epidemiology, Colorado School of Public Health, University of Colorado Anschutz Medical Campus, Aurora, CO USA; Department of Medicine, National Jewish Health, Denver, CO USA; Department of Epidemiology, Johns Hopkins Bloomberg School of Public Health, Baltimore, MD USA; Morehouse School of Medicine, Atlanta, GA USA; Division of Pulmonary, Allergy & Critical Care Medicine, LDS Hospital, Salt Lake City, UT USA; Los Angeles Biomedical Research Institute at Harbor-UCLA Medical Center, Torrance, CA USA; Wolfson Institute for Biomedical Research, University College London, London, UK; Division of Pulmonary, Allergy, and Critical Care Medicine, University of Alabama at Birmingham, Birmingham, AL USA; Department of Clinical Science, University of Bergen, Bergen, Norway; Department of Biostatistics, Harvard School of Public Health, Boston, MA USA

**Keywords:** Chronic obstructive pulmonary disease, DBH, FEV_1_, FEV_1_/FVC, Genome-wide association study, Spirometry

## Abstract

**Background:**

Pulmonary function decline is a major contributor to morbidity and mortality among smokers. Post bronchodilator FEV_1_ and FEV_1_/FVC ratio are considered the standard assessment of airflow obstruction. We performed a genome-wide association study (GWAS) in 9919 current and former smokers in the COPDGene study (6659 non-Hispanic Whites [NHW] and 3260 African Americans [AA]) to identify associations with spirometric measures (post-bronchodilator FEV_1_ and FEV_1_/FVC). We also conducted meta-analysis of FEV_1_ and FEV_1_/FVC GWAS in the COPDGene, ECLIPSE, and GenKOLS cohorts (total *n* = 13,532).

**Results:**

Among NHW in the COPDGene cohort, both measures of pulmonary function were significantly associated with SNPs at the 15q25 locus [containing *CHRNA3*/*5*, *AGPHD1*, *IREB2*, *CHRNB4*] (lowest *p*-value = 2.17 × 10^−11^), and FEV_1_/FVC was associated with a genomic region on chromosome 4 [upstream of *HHIP*] (lowest *p*-value = 5.94 × 10^−10^); both regions have been previously associated with COPD. For the meta-analysis, in addition to confirming associations to the regions near *CHRNA3/5* and *HHIP*, genome-wide significant associations were identified for FEV_1_ on chromosome 1 [*TGFB2*] (*p*-value = 8.99 × 10^−9^), 9 [*DBH*] (*p*-value = 9.69 × 10^−9^) and 19 [*CYP2A6/7*] (*p*-value = 3.49 × 10^−8^) and for FEV_1_/FVC on chromosome 1 [*TGFB2*] (*p*-value = 8.99 × 10^−9^), 4 [*FAM13A*] (*p*-value = 3.88 × 10^−12^), 11 [*MMP3/12*] (*p*-value = 3.29 × 10^−10^) and 14 [*RIN3*] (*p*-value = 5.64 × 10^−9^).

**Conclusions:**

In a large genome-wide association study of lung function in smokers, we found genome-wide significant associations at several previously described loci with lung function or COPD. We additionally identified a novel genome-wide significant locus with FEV_1_ on chromosome 9 [*DBH*] in a meta-analysis of three study populations.

**Electronic supplementary material:**

The online version of this article (doi:10.1186/s12863-015-0299-4) contains supplementary material, which is available to authorized users.

## Background

In the United States, chronic obstructive pulmonary disease (COPD) is the third leading cause of death [1]. The major environmental risk factor for COPD is cigarette smoking, but only a minority of smokers will develop COPD during their lifetime [2, 3]. COPD risk is most likely the cumulative result of genetic factors, environmental factors such as cigarette smoking, developmental factors, and gene-by-environment interactions [4].

A diagnosis of COPD is based on post bronchodilator spirometric measures of the forced expiratory volume in the first second (FEV_1_) and the forced vital capacity (FVC), the total volume of air expired after a maximal inhalation [5]. The ratio of FEV_1_/FVC is a widely used measure of airflow obstruction [3]. Understanding the genetics underlying these spirometric measurements may help increase our knowledge of the genetics of COPD.

Initial genome wide analyses of spirometric measures of pulmonary function using family-based linkage analyses identified broad genomic regions on chromosome 1, 2, 4, 8, and 18 [6]. Subsequent genome wide association studies in the Framingham cohort, a population based sample, identified the HHIP gene as a susceptibility locus for FEV_1_/FVC [7]. This Framingham cohort was combined with several other population based cohorts forming the CHARGE consortium of greater than 20,000 individuals. This sample, along with the SpiroMeta consortium, another population based sample of over 20,000 individuals, provided the sample for a series of meta-analyses; one used CHARGE as a discovery population with subsequent replication in SpiroMeta [8], a second used SpiroMeta as the discovery population with selected genotyping in an additional 32,000 individuals and a pooled meta analysis with the CHARGE consortium [9], and a third combined CHARGE and SpiroMeta in the discovery phase (*n* = 48,201) with SNP replication in an additional combined population based sample of 46,411 individuals [10]. The first two of these meta-analyses confirmed HHIP as a susceptibility locus for FEV_1_/FVC and identified multiple additional loci that were significantly associated with spriometric measures of pulmonary function. The third meta-analysis identified 16 new loci for pre bronchodilator pulmonary function in addition to 10 previously reported loci [10].

To examine the role of smoking on the genetic susceptibility to spirometric measures of pulmonary function, the CHARGE/SpiroMeta samples with additional European ancestry samples totalling more than 30,000 individuals were stratified by smoking status (ever versus never smokers) [11]. Among smokers, a novel signal on chromosome 15q25 (CHRNA5/A3/B4 was identified for airflow obstruction defined as pre bronchodialator FEV_1_ and FEV_1_/FVC below the lower limit of normal. This is the major genomic region for nicotine dependence and smoking exposure and related traits [12]. More recently a genome wide study of pulmonary function identified the CYP2U1 gene [involved in nicotine metabolism], however, this was not replicated in the CHARGE/SpiroMeta sample [13].

Given that smoking is the major environmental determinant of pulmonary function decline, we performed a GWAS of the full quantitative range of post bronchodilator pulmonary function in 9919 current and former smokers of the COPDGene study with complete data and genome wide genotyping. We hypothesized that we would identify novel genetic loci and replicate known genomic regions affecting pulmonary function by performing a GWAS of post bronchodilator spirometric measures in the COPDGene study, a multi-center observational study designed to identify genetic factors associated with risk of COPD. In addition, to insure the GWAS results are generalizable beyond a single study, we performed a meta-analysis of post bronchodilator FEV_1_ and FEV_1_/FVC ratio over three similar studies: the COPDGene, ECLIPSE, and GenKOLS studies. Characteristics of the 3 studies (COPDGene, ECLIPSE, and GenKOLS) are given in Table 1. We also assessed whether different genomic regions were associated with spirometric measures of pulmonary function separately among non-Hispanic white (NHW) and African-American (AA) COPDGene subjects.

**Table 1:** Characteristics of COPDGene, ECLIPSE, and GenKOLS Subjects included in genome-wide association analysis. For continuous variables, the mean is given first followed by the standard deviation

	COPDGene NHW	COPDGene AA	ECLIPSE	GenKOLS
Sample size (#COPD cases)	6659 (2812)	3260 (821)	1942 (1764)	1671 (863)
Gender (% male)	47.73 %	44.79 %	66.17 %	58.71 %
Age (years)	62.0 (8.9)	54.6 (7.2)	63.1 (7.6)	60.7 (11.1)
Height (cm)	169.7 (9.5)	171.0 (9.7)	169.7 (9.1)	170.8 (8.9)
BMI (kg/m2)	28.7 (6.0)	29.07 (6.7)	26.8 (5.5)	25.9 (4.6)
Pack-years	47.2 (26.0)	38.3 (21.6)	48.6 (27.7)	26.0 (17.4)
Current Smoking (%)	38.9 %	80.0 %	34.8 %	43.8 %
FEV1 (L)	2.20 (0.95)	2.30 (0.86)	1.50 (0.78)	2.38 (1.10)
FEV1 (% predicted)	74.0 (26.0)	82.0 (23.9)	53.1 (23.2)	72.2 (26.2)
FEV1/FVC	0.64 (0.17)	0.72 (0.14)	0.48 (0.15)	0.65 (0.17)

*Notes*: FEV_1_, FEV_1_ (% predicted), and FEV_1_/FVC are all based on post-bronchodilator spirometry

## Results

### COPDGene GWAS in Non-Hispanic Whites

For FEV_1_ among all NHW COPDGene participants, several SNPs at the 15q25 locus [near *CHRNA3, CHRNA5, CHRNB4, AGPHD1,* and *IREB2*] reached genome-wide significance. For FEV_1_/FVC among all NHW COPDGene subjects, several SNPs in the same region on chromosome 15 reached genome-wide significance. Tables 2 and 3 show *p*-values for the most significant SNPs in these regions. Additional file 1: Tables S5–S6 list all SNPs with a *p*-value less than 5 × 10^−6^.

**Table 2:** Genome-wide significant results for FEV_1_/FVC in the meta-analysis. The SNP with the lowest *p* value within each region or gene is listed ordered by chromosome number

SNP	Position (bp)	CHR	Nearest Gene	Coded Allele	COPDGene NHW			COPDGene AA			Meta-Analysis of COPDGene NHW, COPDGene AA, ECLIPSE and GenKOLS		
					Allele Freq	Beta	P	Allele Freq	Beta	P	Allele Freq	Beta	P
rs72738834	218623888	1	*TGFB2*	G/G/A	0.79	−0.01	0.00276	0.834	−0.017	5.34E-05	0.81	−0.013	6.51E-09
rs6837671	89873092	4	*FAM13A*	G/G/A	0.60	−0.01	5.18E-06	0.416	0.007	0.03196	0.535	0.013	5.45E-13
rs13141641	145506456	4	*LOC646576 (near HHIP)*	C/C/T	0.60	−0.02	5.72E-09	0.888	−0.015	0.00477	0.632	−0.018	9.52E-20
rs72981684	102724211	11	*MMP12*	T/T/T	0.88	−0.02	2.46E-07	0.968	−0.024	0.02783	0.114	0.019	3.92E-10
rs72981675	102721251	11	*MMP3*	T/T/T	0.88	−0.02	2.80E-07	0.968	−0.024	0.02842	0.114	0.019	3.65E-10
rs754388	93115410	14	*RIN3*	G/G/C	0.82	−0.02	3.17E-07	0.85	−0.01	0.00898	0.83	−0.014	5.54E-09
rs56077333	78899003	15	*CHRNA3*	A/A/A	0.65	−0.02	4.70E-10	0.816	0.019	6.02E-06	0.314	−0.018	9.55E-20
rs8042849	78817929	15	*AGPHD1*	C/C/T	0.56	0.02	2.71E-10	0.568	0.008	0.02729	0.561	0.015	2.81E-15
rs17486278	78867482	15	*CHRNA5*	C/C/A	0.63	0.02	2.28E-09	0.709	0.016	3.14E-06	0.65	0.017	4.48E-20
rs58365910	78849034	15	*PSMA4*	C/C/T	0.628	0.016	2.28E-09	0.747	0.011	0.00475	0.655	0.015	1.24E-15
rs17487223	78923987	15	*CHRNB4*	G/T/T	0.616	0.016	9.73E-09	0.882	0.013	0.00999	0.349	−0.016	3.28E-15
rs17484524	78772676	15	*IREB2*	G/G/A	0.639	0.015	1.86E-07	0.936	0.01	0.138	0.666	0.015	4.83E-13
rs12913260	79071095	15	*ADAMTS7*	A/A/A	0.59	0.02	1.60E-05	0.88	0.004	0.53	0.371	−0.015	4.82E-08
rs56113850	41353107	19	*CYP2A6*	T/C/T	0.60	−0.02	2.46E-05	0.562	0.01	0.01955	0.462	0.014	5.19E-09

The “Coded Allele” column refers to the reference allele where the first reference allele is for the COPDGene NHW cohort, the second reference allele is for the COPDGene AA cohort, and the third reference allele is for the meta-analysis of COPDGene NHW, COPDGene AA, ECLIPSE and GenKOLS. Note that FEV_1_/FVC was measured on the proportion scale (0–1) and not the percentage scale (0–100)

**Table 3:** Genome-wide significant results for FEV_1_ in the meta-analysis. The SNP with the lowest *p* value within each region or gene is listed ordered by chromosome number

SNP	Position (bp)	CHR	Gene/Nearest Gene	Coded Allele	COPDGene NHW			COPDGene AA			Meta-Analysis of COPDGene NHW, COPDGene AA, ECLIPSE and GenKOLS		
					Allele Freq	Beta	P	Allele Freq	Beta	P	Allele Freq	Beta	P
rs10863398	218588279	1	*TGFB2*	A/A/A	0.883	−0.055	0.00539	0.695	−0.07	4.94E-05	0.202	0.067	1.06E-08
rs138641402	145445779	4	*LOC646576 (near HHIP)*	T/T/A	0.641	−0.067	2.28E-06	0.919	−0.09	0.00479	0.668	−0.083	8.99E-15
rs6837671	89873092	4	*FAM13A*	G/G/A	0.595	0.057	1.11E-05	0.416	0.059	0.00024	0.537	0.064	2.89E-13
rs1108581	136505241	9	*DBH*	A/A/A	0.196	0.068	2.29E-05	0.438	0.045	0.00394	0.292	−0.058	8.72E-09
rs56077333	78899003	15	*CHRNA3*	A/A/A	0.649	0.089	9.49E-11	0.816	0.079	0.00014	0.318	−0.084	5.29E-18
rs8031948	78816057	15	*AGPHD1*	T/T/T	0.63	0.084	2.61E-10	0.838	0.05	0.02287	0.336	−0.075	2.86E-15
rs17486278	78867482	15	*CHRNA5*	C/C/A	0.632	0.085	1.44E-10	0.709	0.073	4.31E-05	0.647	0.079	3.48E-18
rs17487223	78923987	15	*CHRNB4*	T/T/T	0.616	0.081	1.82E-09	0.882	0.066	0.01205	0.353	−0.076	2.06E-14
rs2568494	78740964	15	*IREB2*	G/G/A	0.367	−0.076	7.25E-09	0.443	−0.028	0.07723	0.545	−0.062	1.66E-12
rs58365910	78849034	15	*PSMA4*	C/C/T	0.628	0.085	1.65E-10	0.747	0.045	0.01584	0.652	0.072	6.69E-15
rs56113850	41353107	19	*CYP2A6*	T/C/T	0.596	−0.067	0.00045	0.562	0.048	0.01985	0.458	0.067	1.51E-08

The “Coded Allele” column refers to the reference allele where the first reference allele is for the COPDGene NHW cohort, the second reference allele is for the COPDGene AA cohort, and the third reference allele is for the meta-analysis of COPDGene NHW, COPDGene AA, ECLIPSE and GenKOLS

### COPDGene GWAS in African Americans

For both FEV_1_ and FEV_1_/FVC among all AA COPDGene subjects, there were a few SNPs that were genome-wide significant, but these SNPs were all imputed, with low minor allele frequency (<5 %), and in a region with no other non-imputed SNPs. Therefore, we are not confident that these signals are valid associations. The top SNPs for these analyses with *p*-values less than 5 × 10^−6^ can be found in the Additional file 1: Tables S1 and S2.

### Results from the Meta-Analysis: COPDGene participants combined with the ECLIPSE and GenKOLS cohorts

For FEV_1_ and FEV_1_/FVC, most of the genome-wide significant results were again at the 15q25 locus, on chromosome 4 in *FAM13A*, and on chromosome 4 near *HHIP*. In addition, Table 2 shows other genome-wide significant findings for FEV_1_/FVC on chromosome 1 [*TGFB2*], 9 [*DBH*] and 19 [*CYP2A6, CYP2A7*]. Table 3 shows other genome-wide significant findings for FEV_1_ on chromosome 1 [*TGFB2*], 11 [*MMP3,MMP12*] and 14 [*RIN3*]. The top SNPs for these analyses with *p*-values less than 5 × 10^−6^ can be found in the Additional file 1: Tables S9 and S10.

### Case-only analyses among NHW in COPDGene, AA in COPDGene, and within the meta-analysis

In addition, we examined the genetic susceptibility to variation in these pulmonary function phenotypes in COPD cases only (GOLD stages 2–4) (2820 NHW in COPDGene, 821 AA in COPDGene, 1764 NHW in ECLIPSE, and 863 NHW in GenKOLS). While no SNPs reached genome-wide significance for either FEV_1_/FVC or FEV_1_ in the case-only analyses, Additional file 1: Tables S3–S4, S7-S8, and S11–S12 show the top SNPs for these analyses. Some of the regions that met genome-wide significance in the entire study population had at least nominal evidence for association in COPD cases only. In addition to the well-established COPD genetic loci near *HHIP, FAM13A*, and *CHRNA3/5*, the *MMP3/MMP12* and *DBH* regions had *P* < 0.001 evidence for association to lung function levels within COPD cases. Power may have been limited in the case-only analysis, but it is also possible that genetic determinants are more important for the presence/absence of COPD than for the severity of airflow obstruction within COPD cases.

### Comparison of Findings among AA and NHW

**Fig. 1:**
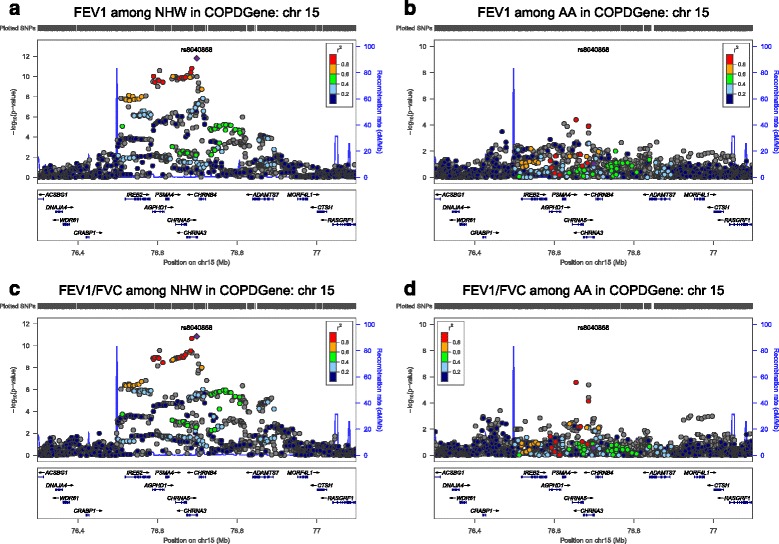
Comparison of chromosome 15 region [*CHRNA3/5, IREB2*] between NHW (**a** and **c**) and AA (**b** and **d**) in COPDGene for FEV_1_ (**a** and **b**), and FEV_1_/FVC (**c** and **d**). Note that there is a similar but narrower and less significant signal in AA

**Fig. 2:**
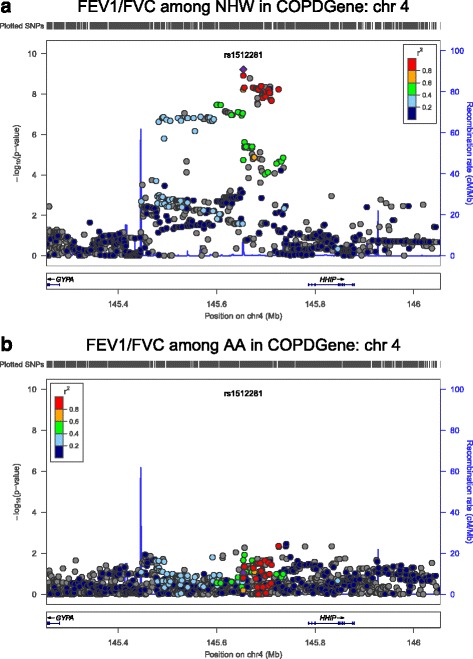
Comparison of chromosome 4 region [near *HHIP*] between NHW (**a**) and AA (**b**) for FEV_1_/FVC. Note that unlike for chromosome 15, there is not a similar signal in AA, although this could be due to the reduced statistical power in the smaller sample size of AA

Figure 1 shows how the *p*-values of the SNPs on chromosome 15 [*CHRNA3, CHRNA5, CHRNB4, AGPHD1,* and *IREB2*] compare among the NHW and AA COPDGene subjects for both spirometric measures of pulmonary function. While no single SNP reaches genome-wide significance in this region among the AA subjects, there is a region near *CHRNA3* with several SNPs that have *p*-values in the range of 5x10^−6^. It appears that the results among AA are similar to those in NHW but may not have the power to reach genome-wide significance due to the smaller sample size. Figure 2 shows qualitatively different association results in the NHW (highly associated) and AA (not associated) subjects for the region on chromosome 4 near *HHIP*, although reduced power in the AA subjects could contribute to reduced evidence for association.

### Comparison to previously published spirometry GWA studies

We considered genome-wide significant results from a previously published spirometry GWA analysis in general population samples, which combined the Cohorts for Heart and Aging Research in Genomic Epidemiology (CHARGE) and SpiroMeta studies [10]. Table 4 shows *p*-values of these loci from the CHARGE/SpiroMeta analyses in the COPDGene sub-populations analysed above. Except for *HHIP* and *FAM13A*, none of the other regions achieved genome-wide significance (*p*-value < 5 × 10^−8^) in the COPDGene cohort or in the meta-analysis of COPDGene, GenKOLS, and ECLIPSE. However, quite a few SNPs had a signal in the same direction as CHARGE/SpiroMeta and met a nominal levels of significance using Bonferroni correction (*p*-value < 0.0018 for the 28 regions tested) in the meta-analysis of these three study populations, including SNPs in or near AGER, *MFAP2, RARB, GSTCD, NPNT, SPATA9, ADAM19, THSD4,* and *CFDP1.*

**Table 4:** Comparison of the current GWA results with the novel genome wide results from the largest GWAS of pre bronchodilator FEV_1_ and FEV_1_/FVC ratio in the CHARGE/SpiroMeta consortium

chr	Gene	SNP/Position (bp)	Phenotype	CHARGE/SpiroMeta	COPDGene NHW	COPDGene AA	Meta-analysis: COPDGene, ECLIPSE, and GenKOLS
				Coded Allele/Beta/*P*-value	Coded Allele/Allele Frequency/Beta/*P*-value		
1	*MFAP2*	rs2284746	FEV_1_/FVC	G/-0.04/7.50e-16	G/0.476/0.007/0.012	G/0.807/0.005/0.256	C/0.545/0.007/1.4E-04
		17306675					
1	*TGFB2*	rs993925*	FEV_1_/FVC	T/0.034/1.16e-08	C/0.33/0.002/0.551	C/0.32/0.004/0.232	T/0..594/0.002/0.3655
		218860068					
2	TNS1	rs2571445	FEV_1_	G/0.047/9.83e-11	G/0.393/-0.027/0.03526	G/0/201/0.007/0.7201	A/0.645/-0.015/0.0996
		218683154					
2	*HDAC4*	rs12477314	FEV_1_/FVC	T/0.041/1.68e-08	T/0.8/-0.001/0.8196	T/0.963/-0.003/0.683	T/0.187/0.003/0.1802
		239877148					
3	*RARB*	rs1529672	FEV_1_/FVC	C/-0.048/3.97e-14	C/0.162/0.015/1.88E-05	C/0.20/0.005/0.2018	A/0.82/0.01/6.72E-06
		25520582					
3	MECOM	rs1344555	FEV_1_	T/-0.034/2.65e-08	T/0.809/0.017/0.293	T/0.799/-0.037/0.07322	T/0.194/-0.008/0.4903
		169300219					
4	*FAM13A*	rs2045517	FEV_1_/FVC	T/-0.047/2.0e-11	T/0.595/0.012/5.32E-06	T/0.343/0.005/0.1522	T/0.485/-0.012/5.66E-12
		89870964					
4	GSTCD	rs10516526	FEV_1_	G/0.108/4.75e-14	G/0.935/-0.117/6.16E-06	G/0.951/0.002/0.9647	A/0.694/-0.066/0.00029
		106688904					
4	NPNT	rs17331332	FEV_1_	G/-0.102/1.11e-12	A/0.933/-0.128/6.16E-06	A/0.979/-0.14/0.03813	A/0.064/0.093/3.19E-06
		106808107					
4	*HHIP*	rs1032296	FEV_1_/FVC	T/-0.05/3.42e-12	C/0.413/-0.011/1.82E-05	C/0.17/-0.01/0.023	T/0.59/-0.011/1.80E-08
		145434688	FEV_1_	T/-0.047/8.74e-11	A/0.414/-0.045/0.0004	A/0.17/-0.061/0.004	T/0.585/-0.048/2.27E-07
5	*SPATA9*	rs153916	FEV_1_/FVC	T/-0.031/2.12e-08	C/0.539/-0.008/0.002	C/0.57/-0.008/0.017	T/0.55/-0.008/3.06E-05
		95036700					
5	*HTR4*	rs11168048	FEV_1_/FVC	T/-0.047/5.97e-11	T/0.42/0.01/0.0102	T/0.23/-0.002/0.5213	T/0.425/-0.004/0.04823
		145479139	FEV_1_	T/-0.046/2.43e-10	T/0.42/0.03/0.008	T/0.23/0.01/0.548	T/0.428/-0.02/0.01452
5	*ADAM19*	rs11134779	FEV_1_/FVC	G/-0.042/6.01e-09	G/0.65/0.005/0.086	G/0.411/0.007/0.024	A/0.574/0.006/8.00E-04
		156936766					
6	*AGER*	rs2070600	FEV_1_/FVC	T/0.126/9.07e-15	A/0.043/0.035/6.22E-08	A/0.01/-0.003/0.849	T/0.31/0.026/2.24e-07
		32151443	FEV_1_	T/0.025/1.271e-12	C/0.043/0.121/0.0001	A/0.01/0.054/0.522	T/0.302/0.081/7.43e-04
6	*GPR126*	rs3817928	FEV_1_/FVC	G/0.059/2.27e-12	G/0.806/-0.003/0.311	G/0.802/-0.015/3e-04	A/0.807/-0.006/0.01394
		142750516					
6	*LOC153910*	rs262129	FEV_1_/FVC	G/0.056/2.91e-13	G/0.704/-0.006/0.043	G/0.19/-0.004/0.3078	A/0.59/-0.005/0.009
		142853144					
6	*ZKSCAN3*	rs6903823	FEV_1_	G/-0.037/2.18e-10	G/0.777/0.013/0.380	G/0.592/-0.049/0.00233	A/0.712/-0.013/0.193
		28322296					
6	*NCR3*	rs2857595	FEV_1_/FVC	G/0.037/2.28e-10	G/0.188/0/0.9309	G/0.423/-0.001/0.7777	A/0/-0.001/0.6174
		31568469					
6	*ARMC2*	rs2798641	FEV_1_/FVC	T/-0.041/8.35e-09	C/0.191/-0.009/0.005	C/0.053/-0.005/0.505	T/0.825/-0.008
		109268050					
							0.002
9	*PTCH1*	rs16909859	FEV_1_/FVC	G/0.08/7.45e-10	G/0.075/-0.004/0.432	G/0.295/-0.005/0.156	A/0.67/-0.004/0.1468
		98204792					
10	*CDC123*	rs7068966	FEV_1_/FVC	T/0.033/6.13e-13	C0.522/0.002/0.516	T/0.782/-0.008/0.04255	T/0.449/0.003/0.07659
		12277992					
10	*C10orf1*	rs11001819	FEV_1_	G/-0.029/2.98e-12	A/0.524/-0.009/0.493	A/0.661/0.012/0.4849	A/0.45/0.006/0.472
		78315224					
12	*LRP1*	rs11172113	FEV_1_/FVC	T/-0.032/1.24e-08	T/0.407/0.006/0.038	T/0.428/0.004/0.1701	T/0.415/-0.004/0.01385
		57527283					
12	*CCDC38*	rs1036429	FEV_1_/FVC	T/0.038/2.30e-11	C/0.21/0.006/0.050	C/0.162/0.004/0.399	T/0.809/0.004/0.06425
		96271428					
15	*THSD4*	rs12899618	FEV_1_/FVC	G/0.076/1.86e-15	G/0.152/-0.012/0.0007	G/0.12/-0.009/0.0507	A/0.854/-0.009/2.8e-04
		71645120					
16	*MMP15*	rs12447804	FEV_1_/FVC	T/-0.038/3.59e-08	T/0.79/-0.002/0.598	T/0.938/-0.001/0.911	T/0.191/0.002/0.528
		58075282					
16	*CFDP1*	rs2865531	FEV_1_/FVC	T/0.031/1.77e-11	T/0.592/-0.005/0.078	T/0.351/-0.006/0.05393	A/0.522/-0.005/0.0742
		75390316					
21	*KCNE2*	rs9978142	FEV_1_/FVC	T/-0.043/2.65e-08	T/0.849/0.002/0.558	T/0.799/0.002/0.530	A/0.83/0.001/0.677
		35652239					

The SNP with the lowest *p* value within each region or gene from the CHARGE/Spirometa consortium is listed ordered by chromosome number [10]. Quite a few SNPs met a nominal levels of significance using Bonferroni correction (*P* < 0.0018 for the 28 regions tested)

*In Table 4, while this SNP is not significant in our cohort and meta-analysis, rs12048582 in the TGFB2 gene was genome wide significant (*p*-value = 6.28E-09)

### A comparison of post and pre bronchodilator FEV_1_ and FEV_1_/FVC among NHW in the COPDGene cohort

Post bronchodilator pulmonary function provides the ability to separate individuals with reversible pulmonary function impairments, which is indicative of asthma from those individuals whose pulmonary function is not reversible with a bronchodilator. Thus, measuring post bronchodilator spirometry provides a phenotype that is more homogeneous with respect to the nature of the pulmonary function impairment. We hypothesized that a GWA of post bronchodilator FEV_1_ and FEV_1_/FVC would be similar or more powerful than a GWA of pre bronchodilator FEV_1_ and FEV_1_/FVC. To test this hypothesis, we performed a GWA of pre bronchodilator FEV_1_ and FEV_1_/FVC in the COPDGene cohort among NHW. The correlation between pre and post bronchodilator FEV_1_ is 0.95 and the correlation between pre and post bronchodilator FEV_1_/FVC is 0.98. Additional file 1: Tables S13 and S14 show the genome wide significant results for these analyses. The results for the GWA of pre bronchodilator FEV_1_ are similar to those of post bronchodilator FEV_1_ among NHW in the COPDGene cohort. 12 SNPs on chromosome 15 [*CHRNA3/5*] are significantly associated with post bronchodilator FEV_1_ where as 8 SNPs in this region are significantly associated with pre bronchodilator FEV_1_. For both pre and post bronchodilator FEV_1_/FVC, the same 8 SNPs on chromosome 15 [*CHRNA3/5*] and the same 4 SNPS on chromosome 4 [*HHIP*] are significantly associated with these measures. These comparisons suggest that previous GWAS [7–13] are not biased due to the inclusion of individuals with bronchodilator reversibility. There appears to be only a modest loss in signal in GWAS of pre bronchodilator FEV_1_ compared to post bronchodilator FEV_1_, and no apparent difference in the signal between pre and post bronchodilator FEV_1_/FVC ratio.

## Discussion

To the best of our knowledge, this is the first GWAS of post bronchodilator pulmonary function. These analyses were performed in a large cohort of current and former smokers with the full range of pulmonary function from normal values to severely impaired. We identified multiple loci that were genome wide significant for post bronchodialater FEV_1_ and FEV_1_/FVC in both the COPDGene cohort and in the combined meta-analyses.

The most significant association for both FEV_1_ and FEV_1_/FVC among NHW in the COPDGene cohort and in the combined meta analysis was on chromosome 15q25 [*CHRNA3*]. This region contains a cluster of nicotinic receptors that are associated with nicotine dependence, COPD case status, lower limit of normal for pre bronchodilator airway obstruction, lung cancer, and other smoking related traits [14–23]. A recent analysis by our group suggested this region may both directly and indirectly affect COPD affection status through nicotine dependence [24]. Other genes within this region in linkage disequilibrium also demonstrate significant associations with post bronchodialator FEV_1_ and FEV_1_/FVC ratio including *CHRNA5/B4, IREB2, AGPHD1*, and *ADAMTS7*.

Our results suggest common genetic susceptibility to post bronchodilator FEV_1_ and FEV_1_/FVC, pre bronchodilator measures of pulmonary function, and COPD affection status. We confirmed previous GWA association with *HHIP, TGFB2, FAM13A, MMP12, MMP3*, *CYPA7, CYP2A6,* and *RIN3* from previous studies with our results on post bronchodilator FEV1 and FEV1/FVC.

### Significant Spirometry Results associated with COPD Affection status in COPDGene

In this study, we found that multiple genetic determinants of COPD affection status were associated with spirometric measures of lung function in COPDGene. These results are not at all surprising, since COPD is defined by lung function thresholds. In the GWAS results of COPD and severe COPD affection status in COPDGene [25], there was a significant association with affection status and SNPs in the 15q25 region [*CHRNA3, CHRNA5, CHRNB4, AGPHD1,* and *IREB2*], *HHIP* on chromosome 4, *FAM13A* on chromosome 4, *TGFB2* on chromosome 1, *MMP3/ MMP12* on chromosome 11, and *RIN3* on chromosome 14. We found evidence for all of these regions as genetic determinants of spirometric measures in COPDGene as well.

The region on 15q25 contains the genes *CHRNA3, CHRNA5, CHRNB4, AGPHD1*, and *IREB2*, and numerous GWA have shown evidence of association of this region with COPD, emphysema, lung cancer, and smoking intensity [14–23]. Regions near *HHIP* [26–28], *FAM13A*, and *TGFB2* have been previously associated with lung function and COPD [8–10, 29]. In addition, SNPs in or near *AGER* have previously been reported in association with lung function and with emphysema [30].

The *RIN3* locus on chromosome 14 has not been previously associated with lung function, although SNPs in *RIN3* were associated with COPD affection status in the COPDGene study [25]. We identified an association near *RIN3* with FEV_1_/FVC in the COPDGene cohort. *RIN3*, a Rab5 GTPase binding protein, is expressed in many tissues, including the lung [31, 32].

### Significant spirometry GWA results not significantly associated with affection status in COPDGene

While not previously associated with lung function, *DBH* on chromosome 9 has been associated with smoking intensity [33]. Our finding represents the first evidence of association of this locus with lung function. Although the SNP identified in this study (rs1108581) does not cause amino acid residue changes in DBH, gene expression may be modified either directly or through other variant(s) in strong LD. This view is supported by evidence that a genetic variant (C-1021T or rs1611115), located upstream of the DBH translational start site, accounts for 51 % of the variation in plasma-DBH activity in NHW [33]. SNPs near *CYP2A7* and *CYP2A6* on chromosome 19 have been associated with lung cancer, cigarette smoking, and COPD [34, 35]. Notably, both of these loci were significant despite adjustment for cigarette smoking status.

### Novel nature of COPDGene study

The COPDGene study is novel in several ways. There are many subjects with severe and very severe COPD (GOLD spirometry grades 3–4). There were sufficient numbers of both AA and NHW subjects to allow reasonable power to detect a genetic association with quantitative spirometric measures in these stratified samples. COPDGene has carefully collected standardized spirometric measures and post-bronchodilator spirometry. In addition, all COPDGene subjects were former or current smokers.

### Potential limitations

The COPDGene cohort was ascertained based on smoking status and GOLD stage. Analysing secondary phenotypes in a case–control study can be biased due to this ascertainment condition. However, this is only an issue for SNPs associated with both the ascertainment condition and the secondary phenotype. Since our analysis focused on measures of pulmonary function (one of the primary ascertainment conditions) and adjusted for smoking status, our analysis should be robust against this sampling bias [36]. While COPDGene includes both AA and NHW, the sample size of AA subjects was considerably smaller and therefore had limited statistical power.

### Conclusions

The GWA of lung function measures in COPDGene identified a novel locus on chromosome 9/*DBH* among NHW as being associated with common spirometric measures, and replicated multiple previously reported genetic loci for lung function. Further research will be required to determine the functional genetic variants within these regions of association.

## Methods

### COPDGene study subjects

COPDGene is a multi-center study performed in the United States that has been described in detail previously [37]. The COPDGene study included 10,192 current and former smoking participants with at least 10 pack-years of cigarette smoking history and ages ranging from 45–81. Table 1 details characteristics of the COPDGene participants included in the genome-wide association analysis. We excluded subjects from the analysis with severe alpha-1 antitrypsin deficiency, genotyping failure, or spirometric tests which failed quality control review. This resulted in 9919 subjects (6659 NHW and 3260 AA). Among these subjects, there were 3641 COPD cases (2812 NHW and 821 AA) defined as having Global Initiative for Chronic Obstructive Lung Disease (GOLD) spirometry stages 2–4 with post-bronchodilator FEV1/FVC < 0.70 and FEV1 < 80 % of predicted values.

### Post bronchodilator spirometry measurements

Spirometry in COPDGene was performed using a standardized spirometer (EasyOne by ndd Medical Technologies, Inc, Andover, MA). Spirometry was performed at baseline and repeated approximately 20 min after two puffs (180 mcg) of albuterol administered through a spacer. The analyses in this manuscript focused on the post-bronchodilator spirometric values. Pulmonary function measurements were collected according to the American Thoracic Society/European Respiratory Society guidelines [38]. Methodology for spirometric measures have been described in detail previously [37].

### Genotyping, quality control and imputation

All COPDGene subjects were genotyped using the Illumina HumanOmniExpress by Illumina (San Diego, CA). Details of genotyping quality control have been previously described [25]. Imputation on the COPDGene cohorts was performed using MaCH and minimac [39, 40]. Prephasing and imputation were both performed using 30 rounds and 200 states, with regions divided into 1 megabase and 500 kb flanks. Reference panels for the NHW and AA subjects were the 1000 Genomes Phase I v3 European (EUR) and cosmopolitan reference panels, respectively [41]. Variants with an R-squared value of ≤ 0.3 were removed from further analysis. SNPs with minor allele frequency less than 1 % were excluded. Further details concerning genotyping, quality control, and imputation are posted on the COPDGene website (http://www.copdgene.org). All SNP genomic locations are based on the NCBI37/hg19 assembly.

### Meta-analysis study populations: ECLIPSE and GenKOLS

The Evaluation of COPD Longitudinally to Identify Predictive Surrogate Endpoints (ECLIPSE) was a longitudinal, prospective, observational study conducted at 46 clinical centers in 12 countries with genome-wide SNP data available from 1764 COPD cases and 178 current or ex-smoking controls [42]. The GenKOLS GWAS cohort consists of 863 COPD cases and 808 controls from Bergen, Norway. Genotyping methods and study descriptions for the GenKOLS and ECLIPSE cohorts have been described previously [43, 44]. We limited our analysis in both studies to current or ex-smokers of European descent.

### Statistical analyses

GWA analyses were performed in PLINK (v1.07) and were stratified by race [45]. Linear regression analyses of FEV_1_ and the ratio of FEV_1_/FVC were adjusted for age, gender, pack-years, height and genetic ancestry (as summarized by principal components) by including these covariates in the model. The primary analyses were performed on the whole cohort (including smoking controls with normal spirometry and GOLD stages 2 to 4 COPD in all cohorts, and additionally individuals with unclassified spirometry [FEV_1_ < 80 % predicted but FEV_1_/FVC ≥0.7], and GOLD stage 1 COPD with FEV_1_ ≥ 80 % predicted but FEV_1_/FVC < 0.7 in COPDGene). A secondary analysis was limited to only GOLD 2–4 cases.

A fixed effects meta-analysis was performed using METAL (v 2010-08-01) [46] for FEV_1_ and FEV_1_/FVC, adjusting for the same covariates mentioned above (age, gender, pack-years, height, genetic ancestry as summarized by principal components) for the COPDGene, ECLIPSE, and GenKOLS cohorts. Genome-wide significant associations were defined by *P* < 5 × 10^−8^. Suggestive associations were defined as 5 × 10^−7^ < *P* < 5 × 10^−8^.

### Ethics

The COPDGene, ECLIPSE, and GenKOLS studies were all approved by the respective clinical center institutional review boards. The COPDGene, ECLIPSE, and GenKOLS studies met IRB protocol approved by the NHLBI for human subjects research. For the COPDGene study and the meta-analysis study conducted in this manuscript, we have obtained IRB approval from the Colorado Multiple Institutional Review Board (COMIRB) at the University of Colorado, Colorado School of Public Health.

## Consent

We have obtained written informed consent from the subjects to participate in these studies. We have obtained written informed consent to publish from the participants of the COPDGene, ECLIPSE and GenKOLS studies and no individual patient data or individual clinical data is presented in this manuscript.

## Availability of data and materials

The datasets used in this paper can be found at http://www.ncbi.nlm.nih.gov/projects/gap/cgi-bin/study.cgi?study_id=phs000179.v1.p1

## Additional file

Additional file 1:
**The supplement for this manuscript contains the following information, tables, and figures.** The COPD Foundation funding and a list of the COPDGene and ECLIPSE investigators are given in the supplement. Tables S1–S12 list the top SNPs with a *p*-value <5E-06 for FEV_1_ and FEV_1_/FVC for all subjects and cases only among AA in the COPDGene study, NHW in the COPDGene study, and in the meta-analysis of the COPDGene, ECLIPSE and the GenKOLS studies. Tables S13 and S14 provide a comparison of genome-wide significant results for pre and post bronchodilator FEV_1_ and FEV_1_/FVC among NHW in the COPDGene study. Figures S1 and S2 are region plots for the genome-wide significant results for FEV_1_ and FEV_1_/FVC, respectively, in the meta-analysis. (DOC 15181 kb)

## Abbreviations

COPD: Chronic obstructive pulmonary disease
SNP: Single nucleotide polymorphism
MAF: Minor allele frequency
AA: African American
NHW: Non-Hispanic White
FEV_1_: Forced expiratory volume in the first second
FVC: Forced vital capacity, the total volume of air expired after a maximal inhalation
GWAS: Genome wide association study
