# Supplementary material for: A genome-wide association study identifies risk loci for spirometric measures among smokers of European and African ancestry
Source: BMC Genet. 2015 Dec 3;16:138. doi: 10.1186/s12863-015-0299-4 (PMC4668640; doi:10.1186/s12863-015-0299-4)
Supplement: Additional file 1: — The supplement for this manuscript contains the following information, tables, and figures. The COPD Foundation funding and a list of the COPDGene and ECLIPSE investigators are given in the supplement. Tables S1–S12 list the top SNPs with a p-value <5E-06 for FEV1 and FEV1/FVC for all subjects and cases only among AA in the COPDGene study, NHW in the COPDGene study, and in the meta-analysis of the COPDGene, ECLIPSE and the GenKOLS studies. Tables S13 and S14 provide a comparison of genome-wide significant results for pre and post bronchodilator FEV1 and FEV1/FVC among NHW in the COPDGene study. Figures S1 and S2 are region plots for the genome-wide significant results for FEV1 and FEV1/FVC, respectively, in the meta-analysis. (DOC 15181 kb) [file 12863_2015_299_MOESM1_ESM.doc]

**NIH Grant Support and Disclaimer**

This work was supported by National Heart, Lung and Blood Institute NHLBI R01 HL084323, P01 HL083069, P01 HL105339 and R01 HL089856 (E.K.S.); K08 HL097029 and R01 HL113264 (M.H.C.), and R01 HL089897 (J.D.C.). The content is solely the responsibility of the authors and does not necessarily represent the official views of the National Heart, Lung, And Blood Institute or the National Institutes of Health.

**COPD Foundation Funding**

The COPDGene project is also supported by the COPD Foundation through contributions made to an Industry Advisory Board comprised of AstraZeneca, Boehringer Ingelheim, Novartis, Pfizer, GlaxoSmithKline, Siemens and Sunovion.

**COPDGene Investigators – Core Units**

*Administrative Core*: James Crapo, MD (PI), Edwin Silverman, MD, PhD (PI), Barry Make, MD, Elizabeth Regan, MD, PhD, Rochelle Lantz, Lori Stepp, Sandra Melanson,

*Genetic Analysis Core*: Terri Beaty, PhD, Barbara Klanderman, PhD, Nan Laird, PhD, Christoph Lange, PhD, Michael Cho, MD, Stephanie Santorico, PhD, John Hokanson, MPH, PhD, Dawn DeMeo, MD, MPH, Nadia Hansel, MD, MPH, Craig Hersh, MD, MPH, Peter Castaldi, MD, MSc, Merry-Lynn McDonald, PhD, Jing Zhou, MD, PhD, Manuel Mattheissen, MD, PhD, Emily Wan, MD, Megan Hardin, MD, Jacqueline Hetmanski, MS, Margaret Parker, MS, Tanda Murray, MS

*Imaging Core*: David Lynch, MB, Joyce Schroeder, MD, John Newell, Jr., MD, John Reilly, MD, Harvey Coxson, PhD, Philip Judy, PhD, Eric Hoffman, PhD, George Washko, MD, Raul San Jose Estepar, PhD, James Ross, MSc, Mustafa Al Qaisi, MD, Jordan Zach, Alex Kluiber, Jered Sieren, Tanya Mann, Deanna Richert, Alexander McKenzie, Jaleh Akhavan, Douglas Stinson

*PFT QA Core, LDS Hospital, Salt Lake City, UT*: Robert Jensen, PhD

*Biological Repository*, *Johns Hopkins University, Baltimore, MD*: Homayoon Farzadegan, PhD, Stacey Meyerer, Shivam Chandan, Samantha Bragan

*Data Coordinating Center and Biostatistics*, *National Jewish Health, Denver, CO*: Douglas Everett, PhD, Andre Williams, PhD, Carla Wilson, MS, Anna Forssen, MS, Amber Powell, Joe Piccoli

*Epidemiology Core*, *University of Colorado School of Public Health, Denver, CO*: John Hokanson, MPH, PhD, Marci Sontag, PhD, Jennifer Black-Shinn, MPH, Gregory Kinney, MPH, PhDc, Sharon Lutz, MPH, PhD

**COPDGene Investigators – Clinical Centers**

*Ann Arbor VA:* Jeffrey Curtis, MD, Ella Kazerooni, MD

*Baylor College of Medicine, Houston, TX*: Nicola Hanania, MD, MS, Philip Alapat, MD, Venkata Bandi, MD, Kalpalatha Guntupalli, MD, Elizabeth Guy, MD, Antara Mallampalli, MD, Charles Trinh, MD, Mustafa Atik, MD, Hasan Al-Azzawi, MD, Marc Willis, DO, Susan Pinero, MD, Linda Fahr, MD, Arun Nachiappan, MD, Collin Bray, MD, L. Alexander Frigini, MD, Carlos Farinas, MD, David Katz, MD, Jose Freytes, MD, Anne Marie Marciel, MD

*Brigham and Women’s Hospital, Boston, MA*: Dawn DeMeo, MD, MPH, Craig Hersh, MD, MPH, George Washko, MD, Francine Jacobson, MD, MPH, Hiroto Hatabu, MD, PhD, Peter Clarke, MD, Ritu Gill, MD, Andetta Hunsaker, MD, Beatrice Trotman-Dickenson, MBBS, Rachna Madan, MD

*Columbia University, New York, NY*: R. Graham Barr, MD, DrPH, Byron Thomashow, MD, John Austin, MD, Belinda D’Souza, MD

*Duke University Medical Center, Durham, NC*: Neil MacIntyre, Jr., MD, Lacey Washington, MD, H Page McAdams, MD

*Fallon Clinic, Worcester, MA*: Richard Rosiello, MD, Timothy Bresnahan, MD, Joseph Bradley, MD, Sharon Kuong, MD, Steven Meller, MD, Suzanne Roland, MD

*Health Partners Research Foundation, Minneapolis, MN*: Charlene McEvoy, MD, MPH, Joseph Tashjian, MD

*Johns Hopkins University, Baltimore, MD*: Robert Wise, MD, Nadia Hansel, MD, MPH, Robert Brown, MD, Gregory Diette, MD, Karen Horton, MD

*Los Angeles Biomedical Research Institute at Harbor UCLA Medical Center, Los Angeles, CA*: Richard Casaburi, MD, Janos Porszasz, MD, PhD, Hans Fischer, MD, PhD, Matt Budoff, MD, Mehdi Rambod, MD

*Michael E. DeBakey VAMC, Houston*, TX: Amir Sharafkhaneh, MD, Charles Trinh, MD, Hirani Kamal, MD, Roham Darvishi, MD, Marc Willis, DO, Susan Pinero, MD, Linda Fahr, MD, Arun Nachiappan, MD, Collin Bray, MD, L. Alexander Frigini, MD, Carlos Farinas, MD, David Katz, MD, Jose Freytes, MD, Anne Marie Marciel, MD

*Minneapolis VA:* Dennis Niewoehner, MD, Quentin Anderson, MD, Kathryn Rice, MD, Audrey Caine, MD

*Morehouse School of Medicine, Atlanta, GA*: Marilyn Foreman, MD, MS, Gloria Westney, MD, MS, Eugene Berkowitz, MD, PhD

*National Jewish Health, Denver, CO*: Russell Bowler, MD, PhD, David Lynch, MB, Joyce Schroeder, MD, Valerie Hale, MD, John Armstrong, II, MD, Debra Dyer, MD, Jonathan Chung, MD, Christian Cox, MD

*Temple University, Philadelphia, PA*: Gerard Criner, MD, Victor Kim, MD, Nathaniel Marchetti, DO, Aditi Satti, MD, A. James Mamary, MD, Robert Steiner, MD, Chandra Dass, MD, Libby Cone, MD

*University of Alabama, Birmingham, AL:* William Bailey, MD, Mark Dransfield, MD, Michael Wells, MD, Surya Bhatt, MD, Hrudaya Nath, MD, Satinder Singh, MD

*University of California, San Diego, CA*: Joe Ramsdell, MD, Paul Friedman, MD

*University of Iowa, Iowa City, IA*: Alejandro Cornellas, MD, John Newell, Jr., MD, Edwin JR van Beek, MD, PhD

*University of Michigan, Ann Arbor, MI*: Fernando Martinez, MD, MeiLan Han, MD, Ella Kazerooni, MD

*University of Minnesota, Minneapolis, MN*: Christine Wendt, MD, Tadashi Allen, MD

*University of Pittsburgh, Pittsburgh, PA*: Frank Sciurba, MD, Joel Weissfeld, MD, MPH, Carl Fuhrman, MD, Jessica Bon, MD, Danielle Hooper, MD

*University of Texas Health Science Center at San Antonio, San Antonio, TX*: Antonio Anzueto, MD, Sandra Adams, MD, Carlos Orozco, MD, Mario Ruiz, MD, Amy Mumbower, MD, Ariel Kruger, MD, Carlos Restrepo, MD, Michael Lane, MD

**ECLIPSE Investigators** — *Bulgaria:* Y. Ivanov, Pleven; K. Kostov, Sofia. *Canada:* J. Bourbeau, Montreal; M. Fitzgerald, Vancouver, BC; P. Hernandez, Halifax, NS; K. Killian, Hamilton, ON; R. Levy, Vancouver, BC; F. Maltais, Montreal; D. O'Donnell, Kingston, ON. *Czech Republic:* J. Krepelka, Prague. *Denmark:* J. Vestbo, Hvidovre. *The Netherlands:* E. Wouters, Horn-Maastricht. *New Zealand:* D. Quinn, Wellington. *Norway:* P. Bakke, Bergen. *Slovenia:* M. Kosnik, Golnik. *Spain:* A. Agusti, J. Sauleda, P. de Mallorca. *Ukraine:* Y. Feschenko, V. Gavrisyuk, L. Yashina, Kiev; N. Monogarova, Donetsk. *United Kingdom:* P. Calverley, Liverpool; D. Lomas, Cambridge; W. MacNee, Edinburgh; D. Singh, Manchester; J. Wedzicha, London. *United States:* A. Anzueto, San Antonio, TX; S. Braman, Providence, RI; R. Casaburi, Torrance CA; B. Celli, Boston; G. Giessel, Richmond, VA; M. Gotfried, Phoenix, AZ; G. Greenwald, Rancho Mirage, CA; N. Hanania, Houston; D. Mahler, Lebanon, NH; B. Make, Denver; S. Rennard, Omaha, NE; C. Rochester, New Haven, CT; P. Scanlon, Rochester, N; D. Schuller, Omaha, NE; F. Sciurba, Pittsburgh; A. Sharafkhaneh, Houston; T. Siler, St. Charles, MO; E. Silverman, Boston; A. Wanner, Miami; R. Wise, Baltimore; R. ZuWallack, Hartford, CT.

**ECLIPSE Steering Committee**: H. Coxson (Canada), C. Crim (GlaxoSmithKline, USA), L. Edwards (GlaxoSmithKline, USA), D. Lomas (UK), W. MacNee (UK), E. Silverman (USA), R. Tal Singer (Co-chair, GlaxoSmithKline, USA), J. Vestbo (Co-chair, Denmark), J. Yates (GlaxoSmithKline, USA).

**ECLIPSE Scientific Committee**: A. Agusti (Spain), P. Calverley (UK), B. Celli (USA), C. Crim (GlaxoSmithKline, USA), B. Miller (GlaxoSmithKline, USA), W. MacNee (Chair, UK), S. Rennard (USA), R. Tal-Singer (GlaxoSmithKline, USA), E. Wouters (The Netherlands), J. Yates (GlaxoSmithKline, USA).

**Note:** For all of the supplemental tables, the meta- analysis is for COPDGene NHW, COPDGene AA, ECLIPSE, and GenKOLS.

**Table S1:** Top SNPs with a p-value < 5E-06 for FEV1/FVC among African Americans (AA) in COPDGene.

| SNP | Chr | Gene | Nearest Gene Up | Nearest Gene Down | COPDGene NHW  All Subjects | COPDGene NHW Cases Only | COPDGene AA  All Subjects | COPDGene AA Cases Only | | Meta-analysis  All Subjects | | Meta-analysis  Cases Only | |
| --- | --- | --- | --- | --- | --- | --- | --- | --- | --- | --- | --- | --- | --- |
|  |  |  |  |  | Coded Allele /Beta/P | Coded Allele /Beta/P | Coded Allele /Beta/P | Coded Allele / Beta/ P | | Coded Allele / Beta/ P | | Coded Allele / Beta/ P | |
| rs181927184 | 1 |  | [RUNX3 (227174)](http://genome.ucsc.edu/cgi-bin/hgTracks?hgHubConnect.destUrl=..%2Fcgi-bin%2FhgTracks&clade=mammal&org=Human&db=hg19&position=RUNX3) | [SYF2 (64822)](http://genome.ucsc.edu/cgi-bin/hgTracks?hgHubConnect.destUrl=..%2Fcgi-bin%2FhgTracks&clade=mammal&org=Human&db=hg19&position=SYF2) | NA | NA | A/0.998/0.304/7.06e-07 | A/0.996/0.196/0.00383 | | NA | | NA | |
| rs116540944 | 1 | [GRIK3](http://genome.ucsc.edu/cgi-bin/hgTracks?hgHubConnect.destUrl=..%2Fcgi-bin%2FhgTracks&clade=mammal&org=Human&db=hg19&position=GRIK3) |  |  | NA | NA | G/0.973/0.064/2.36e-06 | | G/0.965/0.054/0.01089 | | NA | | NA |
| rs76264265 | 1 | [GRIK3](http://genome.ucsc.edu/cgi-bin/hgTracks?hgHubConnect.destUrl=..%2Fcgi-bin%2FhgTracks&clade=mammal&org=Human&db=hg19&position=GRIK3) |  |  | NA | NA | T/0.972/0.062/2.93e-06 | | T/0.965/0.056/0.00741 | | NA | | NA |
| rs77653389 | 1 | [GRIK3](http://genome.ucsc.edu/cgi-bin/hgTracks?hgHubConnect.destUrl=..%2Fcgi-bin%2FhgTracks&clade=mammal&org=Human&db=hg19&position=GRIK3) |  |  | NA | NA | C/0.972/0.062/3.1e-06 | | C/0.965/0.055/0.00887 | | NA | | NA |
| rs183468661 | 1 | [RLF](http://genome.ucsc.edu/cgi-bin/hgTracks?hgHubConnect.destUrl=..%2Fcgi-bin%2FhgTracks&clade=mammal&org=Human&db=hg19&position=RLF) | [PPT1 (71700)](http://genome.ucsc.edu/cgi-bin/hgTracks?hgHubConnect.destUrl=..%2Fcgi-bin%2FhgTracks&clade=mammal&org=Human&db=hg19&position=PPT1) | [TMCO2 (78730)](http://genome.ucsc.edu/cgi-bin/hgTracks?hgHubConnect.destUrl=..%2Fcgi-bin%2FhgTracks&clade=mammal&org=Human&db=hg19&position=TMCO2) | NA | NA | T/0.999/0.388/1.6e-06 | | T/0.998/0.284/0.00113 | | NA | | NA |
| rs147410855 | 1 |  |  | [FGGY (24732)](http://genome.ucsc.edu/cgi-bin/hgTracks?hgHubConnect.destUrl=..%2Fcgi-bin%2FhgTracks&clade=mammal&org=Human&db=hg19&position=FGGY) | NA | NA | C/0.995/0.153/4.01e-07 | | C/0.991/0.077/0.04958 | | NA | | NA |
| rs192166173 | 1 |  | [PGM1 (36092)](http://genome.ucsc.edu/cgi-bin/hgTracks?hgHubConnect.destUrl=..%2Fcgi-bin%2FhgTracks&clade=mammal&org=Human&db=hg19&position=PGM1) | [ROR1 (77681)](http://genome.ucsc.edu/cgi-bin/hgTracks?hgHubConnect.destUrl=..%2Fcgi-bin%2FhgTracks&clade=mammal&org=Human&db=hg19&position=ROR1) | NA | NA | A/0.999/0.342/4.43e-06 | | A/0.998/0.184/0.01084 | | NA | | NA |
| rs138006855 | 1 |  |  |  | NA | NA | A/0.999/0.308/3.13e-06 | | A/0.997/0.179/0.00823 | | NA | | NA |
| rs181954514 | 1 | [FAM73A](http://genome.ucsc.edu/cgi-bin/hgTracks?hgHubConnect.destUrl=..%2Fcgi-bin%2FhgTracks&clade=mammal&org=Human&db=hg19&position=FAM73A) | [USP33 (27497)](http://genome.ucsc.edu/cgi-bin/hgTracks?hgHubConnect.destUrl=..%2Fcgi-bin%2FhgTracks&clade=mammal&org=Human&db=hg19&position=USP33) | [NEXN (101165)](http://genome.ucsc.edu/cgi-bin/hgTracks?hgHubConnect.destUrl=..%2Fcgi-bin%2FhgTracks&clade=mammal&org=Human&db=hg19&position=NEXN) | NA | NA | A/1/0.598/2.77e-07 | | A/0.999/0.389/0.00088 | | NA | | NA |
| rs114304737 | 1 |  | [COL24A1 (109275)](http://genome.ucsc.edu/cgi-bin/hgTracks?hgHubConnect.destUrl=..%2Fcgi-bin%2FhgTracks&clade=mammal&org=Human&db=hg19&position=COL24A1) | [ODF2L (81077)](http://genome.ucsc.edu/cgi-bin/hgTracks?hgHubConnect.destUrl=..%2Fcgi-bin%2FhgTracks&clade=mammal&org=Human&db=hg19&position=ODF2L) | A/0.98/-0.01/0.3064 | A/0.98/-0.022/0.08626 | A/0.997/0.159/1.21e-06 | | A/0.994/0.105/0.01193 | | A/0.02/0.001/0.887 | | A/0.02/0.013/0.1587 |
| rs138897663 | 1 |  |  |  | NA | NA | A/0.999/0.248/3.34e-06 | | A/0.997/0.144/0.01687 | | NA | | NA |
| rs150884133 | 1 |  |  |  | NA | NA | A/0.998/0.226/1.57e-06 | | A/0.996/0.13/0.01314 | | NA | | NA |
| rs137917644 | 1 |  |  |  | NA | NA | T/0.998/0.225/1.49e-06 | | T/0.996/0.129/0.01292 | | NA | | NA |
| rs187464554 | 1 |  |  |  | NA | NA | T/0.998/0.225/1.49e-06 | | T/0.996/0.129/0.01292 | | NA | | NA |
| rs187226391 | 1 |  |  |  | NA | NA | C/0.998/0.228/1.37e-06 | | C/0.996/0.131/0.01213 | | NA | | NA |
| rs149819415 | 1 |  |  |  | NA | NA | G/1/0.881/2.23e-06 | | G/0.999/0.482/0.00664 | | NA | | NA |
| rs74507156 | 1 |  |  |  | A/0.997/-0.03/0.3446 | A/0.997/-0.061/0.1336 | A/1/0.454/1.01e-06 | | A/0.999/0.311/0.00076 | | A/0.003/-0.005/0.8249 | | A/0.003/0.023/0.4355 |
| rs189354479 | 1 |  |  |  | G/0.999/-0.055/0.3569 | G/0.999/-0.088/0.3346 | G/1/0.615/6.83e-08 | | G/0.999/0.357/0.00077 | | T/0.999/0.053/0.2255 | | T/0.999/-0.056/0.3136 |
| rs192109529 | 1 |  | [SLC45A3 (29764)](http://genome.ucsc.edu/cgi-bin/hgTracks?hgHubConnect.destUrl=..%2Fcgi-bin%2FhgTracks&clade=mammal&org=Human&db=hg19&position=SLC45A3) | [NUCKS1 (2552)](http://genome.ucsc.edu/cgi-bin/hgTracks?hgHubConnect.destUrl=..%2Fcgi-bin%2FhgTracks&clade=mammal&org=Human&db=hg19&position=NUCKS1) | C/0.995/-0.017/0.4275 | C/0.996/-0.021/0.476 | C/0.999/0.371/1.58e-06 | | C/0.998/0.22/0.01255 | | C/0.007/-0.012/0.421 | | C/0.007/-0.003/0.8354 |
| rs192525030 | 1 |  |  |  | NA | NA | T/1/0.625/2.29e-06 | | T/0.999/0.346/0.00531 | | NA | | NA |
| rs182234674 | 1 | [PROX1-AS1](http://genome.ucsc.edu/cgi-bin/hgTracks?hgHubConnect.destUrl=..%2Fcgi-bin%2FhgTracks&clade=mammal&org=Human&db=hg19&position=PROX1-AS1) |  | [PROX1 (55542)](http://genome.ucsc.edu/cgi-bin/hgTracks?hgHubConnect.destUrl=..%2Fcgi-bin%2FhgTracks&clade=mammal&org=Human&db=hg19&position=PROX1) | NA | NA | T/0.998/0.273/4.87e-07 | | T/0.996/0.095/0.06917 | | NA | | NA |
| rs79131758 | 1 | [ESRRG](http://genome.ucsc.edu/cgi-bin/hgTracks?hgHubConnect.destUrl=..%2Fcgi-bin%2FhgTracks&clade=mammal&org=Human&db=hg19&position=ESRRG) |  |  | C/0.994/0.03/0.2267 | C/0.994/0.021/0.478 | C/0.998/0.251/3.96e-07 | | C/0.996/0.096/0.06 | | C/0.009/-0.028/0.04777 | | C/0.009/-0.012/0.4639 |
| rs138833465 | 1 |  | [RAB3GAP2 (139029)](http://genome.ucsc.edu/cgi-bin/hgTracks?hgHubConnect.destUrl=..%2Fcgi-bin%2FhgTracks&clade=mammal&org=Human&db=hg19&position=RAB3GAP2) | [MARK1 (116695)](http://genome.ucsc.edu/cgi-bin/hgTracks?hgHubConnect.destUrl=..%2Fcgi-bin%2FhgTracks&clade=mammal&org=Human&db=hg19&position=MARK1) | NA | NA | G/0.992/0.12/1.19e-06 | | G/0.987/0.086/0.01173 | | NA | | NA |
| rs181295069 | 1 |  | [LIN9 (14910)](http://genome.ucsc.edu/cgi-bin/hgTracks?hgHubConnect.destUrl=..%2Fcgi-bin%2FhgTracks&clade=mammal&org=Human&db=hg19&position=LIN9) | [PARP1 (35925)](http://genome.ucsc.edu/cgi-bin/hgTracks?hgHubConnect.destUrl=..%2Fcgi-bin%2FhgTracks&clade=mammal&org=Human&db=hg19&position=PARP1) | NA | NA | T/0.999/0.308/2.02e-06 | | T/0.997/0.159/0.01691 | | NA | | NA |
| rs139467600 | 1 | [SLC35F3](http://genome.ucsc.edu/cgi-bin/hgTracks?hgHubConnect.destUrl=..%2Fcgi-bin%2FhgTracks&clade=mammal&org=Human&db=hg19&position=SLC35F3) |  |  | NA | NA | T/0.998/0.235/6.78e-07 | | T/0.996/0.224/0.00074 | | NA | | NA |
| rs187730847 | 1 | [LYST](http://genome.ucsc.edu/cgi-bin/hgTracks?hgHubConnect.destUrl=..%2Fcgi-bin%2FhgTracks&clade=mammal&org=Human&db=hg19&position=LYST) | [MIR1537 (9513)](http://genome.ucsc.edu/cgi-bin/hgTracks?hgHubConnect.destUrl=..%2Fcgi-bin%2FhgTracks&clade=mammal&org=Human&db=hg19&position=MIR1537) | [NID1 (113258)](http://genome.ucsc.edu/cgi-bin/hgTracks?hgHubConnect.destUrl=..%2Fcgi-bin%2FhgTracks&clade=mammal&org=Human&db=hg19&position=NID1) | G/0.997/0.083/0.03834 | G/0.997/0.102/0.03316 | G/0.994/0.122/4.56e-06 | | G/0.99/0.104/0.00278 | | A/0.995/0.082/1.94e-05 | | A/0.996/0.051/0.1103 |
| rs150174091 | 1 | [HEATR1](http://genome.ucsc.edu/cgi-bin/hgTracks?hgHubConnect.destUrl=..%2Fcgi-bin%2FhgTracks&clade=mammal&org=Human&db=hg19&position=HEATR1) | [LGALS8 (31282)](http://genome.ucsc.edu/cgi-bin/hgTracks?hgHubConnect.destUrl=..%2Fcgi-bin%2FhgTracks&clade=mammal&org=Human&db=hg19&position=LGALS8) | [ACTN2 (102208)](http://genome.ucsc.edu/cgi-bin/hgTracks?hgHubConnect.destUrl=..%2Fcgi-bin%2FhgTracks&clade=mammal&org=Human&db=hg19&position=ACTN2) | NA | NA | G/0.999/0.262/4.88e-06 | | G/0.997/0.115/0.04289 | | NA | | NA |
| rs191185851 | 2 |  | [AK093525 (17332)](http://genome.ucsc.edu/cgi-bin/hgTracks?hgHubConnect.destUrl=..%2Fcgi-bin%2FhgTracks&clade=mammal&org=Human&db=hg19&position=AK093525) | [MYCNOS (203602)](http://genome.ucsc.edu/cgi-bin/hgTracks?hgHubConnect.destUrl=..%2Fcgi-bin%2FhgTracks&clade=mammal&org=Human&db=hg19&position=MYCNOS) | T/0.994/0.018/0.3617 | T/0.995/0.038/0.1658 | T/0.998/0.252/2.64e-07 | | T/0.996/0.115/0.02418 | | T/0.005/-0.034/0.03804 | | T/0.005/-0.001/0.944 |
| rs181043431 | 2 |  | [CIB4 (44965)](http://genome.ucsc.edu/cgi-bin/hgTracks?hgHubConnect.destUrl=..%2Fcgi-bin%2FhgTracks&clade=mammal&org=Human&db=hg19&position=CIB4) | [KCNK3 (6404)](http://genome.ucsc.edu/cgi-bin/hgTracks?hgHubConnect.destUrl=..%2Fcgi-bin%2FhgTracks&clade=mammal&org=Human&db=hg19&position=KCNK3) | NA | NA | T/0.999/0.439/3.94e-06 | | T/0.998/0.289/0.00174 | | NA | | NA |
| rs187943782 | 2 | [MAPRE3](http://genome.ucsc.edu/cgi-bin/hgTracks?hgHubConnect.destUrl=..%2Fcgi-bin%2FhgTracks&clade=mammal&org=Human&db=hg19&position=MAPRE3) | [DPYSL5 (32167)](http://genome.ucsc.edu/cgi-bin/hgTracks?hgHubConnect.destUrl=..%2Fcgi-bin%2FhgTracks&clade=mammal&org=Human&db=hg19&position=DPYSL5) | [AK125769 (29250)](http://genome.ucsc.edu/cgi-bin/hgTracks?hgHubConnect.destUrl=..%2Fcgi-bin%2FhgTracks&clade=mammal&org=Human&db=hg19&position=AK125769) | NA | NA | T/0.994/0.116/1.95e-06 | | T/0.99/0.071/0.02534 | | NA | | NA |
| rs185580150 | 2 | [GCKR](http://genome.ucsc.edu/cgi-bin/hgTracks?hgHubConnect.destUrl=..%2Fcgi-bin%2FhgTracks&clade=mammal&org=Human&db=hg19&position=GCKR) | [FNDC4 (12241)](http://genome.ucsc.edu/cgi-bin/hgTracks?hgHubConnect.destUrl=..%2Fcgi-bin%2FhgTracks&clade=mammal&org=Human&db=hg19&position=FNDC4) | [C2orf16 (69021)](http://genome.ucsc.edu/cgi-bin/hgTracks?hgHubConnect.destUrl=..%2Fcgi-bin%2FhgTracks&clade=mammal&org=Human&db=hg19&position=C2orf16) | G/0.991/0.007/0.679 | G/0.991/0.005/0.8057 | G/0.999/0.347/3.02e-06 | | G/0.998/0.259/0.00131 | | A/0.992/0.009/0.5127 | | A/0.992/-0.014/0.4388 |
| rs148738653 | 2 |  | [GCKR (21863)](http://genome.ucsc.edu/cgi-bin/hgTracks?hgHubConnect.destUrl=..%2Fcgi-bin%2FhgTracks&clade=mammal&org=Human&db=hg19&position=GCKR) | [C2orf16 (30975)](http://genome.ucsc.edu/cgi-bin/hgTracks?hgHubConnect.destUrl=..%2Fcgi-bin%2FhgTracks&clade=mammal&org=Human&db=hg19&position=C2orf16) | NA | NA | T/0.996/0.135/1.57e-06 | | T/0.993/0.096/0.00687 | | NA | | NA |
| rs187306222 | 2 | [C2orf16](http://genome.ucsc.edu/cgi-bin/hgTracks?hgHubConnect.destUrl=..%2Fcgi-bin%2FhgTracks&clade=mammal&org=Human&db=hg19&position=C2orf16) | [GCKR (55955)](http://genome.ucsc.edu/cgi-bin/hgTracks?hgHubConnect.destUrl=..%2Fcgi-bin%2FhgTracks&clade=mammal&org=Human&db=hg19&position=GCKR) | [ZNF512 (3387)](http://genome.ucsc.edu/cgi-bin/hgTracks?hgHubConnect.destUrl=..%2Fcgi-bin%2FhgTracks&clade=mammal&org=Human&db=hg19&position=ZNF512) | T/0.991/0.007/0.6586 | T/0.991/0.006/0.7913 | T/0.999/0.346/3.87e-06 | | T/0.998/0.26/0.00133 | | T/0.008/-0.01/0.4674 | | T/0.008/0.012/0.5081 |
| rs147620864 | 2 | [MSH2](http://genome.ucsc.edu/cgi-bin/hgTracks?hgHubConnect.destUrl=..%2Fcgi-bin%2FhgTracks&clade=mammal&org=Human&db=hg19&position=MSH2) | [EPCAM (72365)](http://genome.ucsc.edu/cgi-bin/hgTracks?hgHubConnect.destUrl=..%2Fcgi-bin%2FhgTracks&clade=mammal&org=Human&db=hg19&position=EPCAM) | [AK056077 (26627)](http://genome.ucsc.edu/cgi-bin/hgTracks?hgHubConnect.destUrl=..%2Fcgi-bin%2FhgTracks&clade=mammal&org=Human&db=hg19&position=AK056077) | NA | NA | G/0.998/0.315/4.53e-06 | | G/0.997/0.185/0.01326 | | NA | | NA |
| rs143359137 | 2 | [STON1-GTF2A1L](http://genome.ucsc.edu/cgi-bin/hgTracks?hgHubConnect.destUrl=..%2Fcgi-bin%2FhgTracks&clade=mammal&org=Human&db=hg19&position=STON1-GTF2A1L) | [STON1 (17178)](http://genome.ucsc.edu/cgi-bin/hgTracks?hgHubConnect.destUrl=..%2Fcgi-bin%2FhgTracks&clade=mammal&org=Human&db=hg19&position=STON1) | [GTF2A1L (2104)](http://genome.ucsc.edu/cgi-bin/hgTracks?hgHubConnect.destUrl=..%2Fcgi-bin%2FhgTracks&clade=mammal&org=Human&db=hg19&position=GTF2A1L) | T/0.997/0.054/0.1504 | T/0.997/0.147/0.00415 | T/0.999/0.464/4.61e-06 | | T/0.999/0.294/0.00517 | | T/0.002/-0.079/0.01048 | | T/0.002/-0.07/0.07899 |
| rs180692309 | 2 |  |  |  | NA | NA | T/0.999/0.568/3.63e-07 | | T/0.998/0.348/0.00114 | | NA | | NA |
| rs115249811 | 2 | [NRXN1](http://genome.ucsc.edu/cgi-bin/hgTracks?hgHubConnect.destUrl=..%2Fcgi-bin%2FhgTracks&clade=mammal&org=Human&db=hg19&position=NRXN1) |  |  | C/1/-0.089/0.4017 | C/1/-0.203/0.09554 | C/1/0.834/2.11e-07 | | C/0.999/0.484/0.00168 | | T/0.999/0.086/0.05246 | | T/0.998/0.027/0.5605 |
| rs185508190 | 2 | [NRXN1](http://genome.ucsc.edu/cgi-bin/hgTracks?hgHubConnect.destUrl=..%2Fcgi-bin%2FhgTracks&clade=mammal&org=Human&db=hg19&position=NRXN1) |  |  | A/1/-0.023/0.8145 | A/0.999/-0.048/0.6642 | A/1/0.729/3.48e-06 | | A/0.999/0.406/0.00541 | | A/0.002/-0.077/0.06938 | | A/0.002/-0.033/0.4624 |
| rs7573615 | 2 |  |  |  | T/0.843/0.009/0.01802 | T/0.837/0.009/0.05204 | T/0.542/-0.014/4.67e-06 | | T/0.571/-0.013/0.02765 | | T/0.296/0.004/0.06415 | | T/0.159/-0.006/0.07776 |
| rs180765220 | 2 |  | [EFEMP1 (5262)](http://genome.ucsc.edu/cgi-bin/hgTracks?hgHubConnect.destUrl=..%2Fcgi-bin%2FhgTracks&clade=mammal&org=Human&db=hg19&position=EFEMP1) | [BC043355 (22693)](http://genome.ucsc.edu/cgi-bin/hgTracks?hgHubConnect.destUrl=..%2Fcgi-bin%2FhgTracks&clade=mammal&org=Human&db=hg19&position=BC043355) | NA | NA | T/0.996/0.168/4.85e-06 | | T/0.993/0.104/0.03087 | | NA | | NA |
| rs190486114 | 2 | [BC043355](http://genome.ucsc.edu/cgi-bin/hgTracks?hgHubConnect.destUrl=..%2Fcgi-bin%2FhgTracks&clade=mammal&org=Human&db=hg19&position=BC043355) |  | [MIR217 (27172)](http://genome.ucsc.edu/cgi-bin/hgTracks?hgHubConnect.destUrl=..%2Fcgi-bin%2FhgTracks&clade=mammal&org=Human&db=hg19&position=MIR217) | NA | NA | A/0.996/0.184/1.19e-06 | | A/0.993/0.104/0.03105 | | NA | | NA |
| rs186819721 | 2 | [BC043355](http://genome.ucsc.edu/cgi-bin/hgTracks?hgHubConnect.destUrl=..%2Fcgi-bin%2FhgTracks&clade=mammal&org=Human&db=hg19&position=BC043355) |  | [MIR217 (12949)](http://genome.ucsc.edu/cgi-bin/hgTracks?hgHubConnect.destUrl=..%2Fcgi-bin%2FhgTracks&clade=mammal&org=Human&db=hg19&position=MIR217) | NA | NA | A/0.996/0.186/8.65e-07 | | A/0.993/0.104/0.03207 | | NA | | NA |
| rs138402956 | 2 | [BC043355](http://genome.ucsc.edu/cgi-bin/hgTracks?hgHubConnect.destUrl=..%2Fcgi-bin%2FhgTracks&clade=mammal&org=Human&db=hg19&position=BC043355) |  | [MIR217 (12301)](http://genome.ucsc.edu/cgi-bin/hgTracks?hgHubConnect.destUrl=..%2Fcgi-bin%2FhgTracks&clade=mammal&org=Human&db=hg19&position=MIR217) | NA | NA | C/0.996/0.168/1.05e-06 | | C/0.991/0.073/0.08417 | | NA | | NA |
| rs182751789 | 2 | [BC043355](http://genome.ucsc.edu/cgi-bin/hgTracks?hgHubConnect.destUrl=..%2Fcgi-bin%2FhgTracks&clade=mammal&org=Human&db=hg19&position=BC043355) | [MIR216A (1479)](http://genome.ucsc.edu/cgi-bin/hgTracks?hgHubConnect.destUrl=..%2Fcgi-bin%2FhgTracks&clade=mammal&org=Human&db=hg19&position=MIR216A) | [MIR216B (10175)](http://genome.ucsc.edu/cgi-bin/hgTracks?hgHubConnect.destUrl=..%2Fcgi-bin%2FhgTracks&clade=mammal&org=Human&db=hg19&position=MIR216B) | NA | NA | C/0.997/0.181/3.78e-06 | | C/0.993/0.097/0.05156 | | NA | | NA |
| rs138258488 | 2 |  |  |  | G/0.992/-0.003/0.8905 | G/0.992/0.002/0.9416 | G/0.998/0.169/4e-06 | | G/0.995/0.154/0.00115 | | A/0.993/0.024/0.1167 | | A/0.992/-0.008/0.679 |
| rs75598744 | 2 |  |  |  | C/0.992/-0.002/0.9096 | C/0.992/0.004/0.897 | C/0.997/0.168/3.88e-06 | | C/0.995/0.155/0.00106 | | C/0.007/-0.024/0.1112 | | C/0.008/0.007/0.7314 |
| rs181660893 | 2 |  |  |  | A/0.992/-0.002/0.9101 | A/0.992/0.003/0.9032 | A/0.997/0.17/3.4e-06 | | A/0.995/0.155/0.00105 | | A/0.007/-0.024/0.1151 | | A/0.008/0.007/0.7179 |
| rs78797065 | 2 |  |  |  | C/0.992/0.001/0.9756 | C/0.992/0.004/0.8988 | C/0.997/0.176/1.48e-06 | | C/0.995/0.156/0.00089 | | A/0.993/0.026/0.09125 | | A/0.992/-0.008/0.6948 |
| rs191356286 | 2 |  |  |  | C/0.992/0.008/0.7069 | C/0.992/0.001/0.9592 | C/0.997/0.174/7.84e-07 | | C/0.994/0.127/0.00335 | | A/0.993/0.03/0.04745 | | A/0.992/-0.004/0.8411 |
| rs187133462 | 2 |  |  |  | A/0.999/0.022/0.6836 | A/0.999/0.171/0.04123 | A/1/0.578/4e-07 | | A/0.999/0.304/0.00411 | | NA | | A/0.001/-0.116/0.09652 |
| rs191899678 | 2 |  |  |  | NA | NA | T/0.987/0.069/4.68e-06 | | T/0.981/0.063/0.00589 | | NA | | NA |
| rs17011851 | 2 | [CNTNAP5](http://genome.ucsc.edu/cgi-bin/hgTracks?hgHubConnect.destUrl=..%2Fcgi-bin%2FhgTracks&clade=mammal&org=Human&db=hg19&position=CNTNAP5) |  |  | A/0.967/0.005/0.4842 | A/0.967/0.004/0.6817 | A/0.974/0.049/1.67e-06 | | A/0.965/0.058/0.00029 | | A/0.03/-0.015/0.00469 | | A/0.03/0.002/0.8337 |
| rs1454139 | 2 | [CNTNAP5](http://genome.ucsc.edu/cgi-bin/hgTracks?hgHubConnect.destUrl=..%2Fcgi-bin%2FhgTracks&clade=mammal&org=Human&db=hg19&position=CNTNAP5) |  |  | G/0.967/0.005/0.484 | G/0.967/0.004/0.6817 | G/0.974/0.049/1.66e-06 | | G/0.965/0.058/0.00029 | | A/0.97/0.015/0.00473 | | A/0.97/-0.002/0.8337 |
| rs191947856 | 2 | [CNTNAP5](http://genome.ucsc.edu/cgi-bin/hgTracks?hgHubConnect.destUrl=..%2Fcgi-bin%2FhgTracks&clade=mammal&org=Human&db=hg19&position=CNTNAP5) |  |  | NA | NA | A/0.996/0.162/2.26e-06 | | A/0.994/0.143/0.00154 | | NA | | NA |
| rs17011859 | 2 | [CNTNAP5](http://genome.ucsc.edu/cgi-bin/hgTracks?hgHubConnect.destUrl=..%2Fcgi-bin%2FhgTracks&clade=mammal&org=Human&db=hg19&position=CNTNAP5) |  |  | G/0.967/0.005/0.4835 | G/0.967/0.004/0.6817 | G/0.974/0.05/1.65e-06 | | G/0.965/0.058/0.00029 | | A/0.97/0.015/0.00465 | | A/0.97/-0.002/0.8337 |
| rs17011865 | 2 | [CNTNAP5](http://genome.ucsc.edu/cgi-bin/hgTracks?hgHubConnect.destUrl=..%2Fcgi-bin%2FhgTracks&clade=mammal&org=Human&db=hg19&position=CNTNAP5) |  |  | A/0.967/0.005/0.4831 | A/0.967/0.004/0.6817 | A/0.974/0.05/1.65e-06 | | A/0.965/0.058/0.00029 | | A/0.03/-0.015/0.00462 | | A/0.03/0.002/0.8352 |
| rs66820110 | 2 | [CNTNAP5](http://genome.ucsc.edu/cgi-bin/hgTracks?hgHubConnect.destUrl=..%2Fcgi-bin%2FhgTracks&clade=mammal&org=Human&db=hg19&position=CNTNAP5) |  |  | C/0.967/0.005/0.4828 | C/0.967/0.004/0.6814 | C/0.974/0.05/1.64e-06 | | C/0.965/0.058/0.00029 | | C/0.03/-0.015/0.00462 | | C/0.03/0.002/0.8352 |
| rs1454137 | 2 | [CNTNAP5](http://genome.ucsc.edu/cgi-bin/hgTracks?hgHubConnect.destUrl=..%2Fcgi-bin%2FhgTracks&clade=mammal&org=Human&db=hg19&position=CNTNAP5) |  |  | A/0.967/0.005/0.4828 | A/0.967/0.004/0.6817 | A/0.974/0.05/1.64e-06 | | A/0.965/0.058/0.00029 | | A/0.03/-0.015/0.00465 | | A/0.03/0.002/0.8352 |
| rs186609563 | 2 | [CNTNAP5](http://genome.ucsc.edu/cgi-bin/hgTracks?hgHubConnect.destUrl=..%2Fcgi-bin%2FhgTracks&clade=mammal&org=Human&db=hg19&position=CNTNAP5) |  |  | NA | NA | G/0.994/0.138/3.97e-06 | | G/0.991/0.097/0.01782 | | NA | | NA |
| rs71420856 | 2 |  |  |  | T/0.994/0.035/0.1588 | T/0.993/-0.03/0.2844 | T/0.999/0.326/2.42e-06 | | T/0.997/0.19/0.0062 | | A/0.995/0.039/0.04362 | | A/0.994/-0.021/0.3554 |
| rs78170588 | 2 | [MGAT5](http://genome.ucsc.edu/cgi-bin/hgTracks?hgHubConnect.destUrl=..%2Fcgi-bin%2FhgTracks&clade=mammal&org=Human&db=hg19&position=MGAT5) |  | [TMEM163 (5058)](http://genome.ucsc.edu/cgi-bin/hgTracks?hgHubConnect.destUrl=..%2Fcgi-bin%2FhgTracks&clade=mammal&org=Human&db=hg19&position=TMEM163) | NA | NA | A/0.993/0.096/2.72e-06 | | A/0.988/0.067/0.01863 | | NA | | NA |
| rs116518699 | 2 |  | [AK126351 (50157)](http://genome.ucsc.edu/cgi-bin/hgTracks?hgHubConnect.destUrl=..%2Fcgi-bin%2FhgTracks&clade=mammal&org=Human&db=hg19&position=AK126351) | [DAPL1 (10157)](http://genome.ucsc.edu/cgi-bin/hgTracks?hgHubConnect.destUrl=..%2Fcgi-bin%2FhgTracks&clade=mammal&org=Human&db=hg19&position=DAPL1) | T/1/-0.054/0.443 | T/1/-0.009/0.945 | T/0.988/0.072/2.66e-06 | | T/0.98/0.052/0.0149 | | NA | | T/0.001/0.002/0.9676 |
| rs145710351 | 2 |  | [DAPL1 (4749)](http://genome.ucsc.edu/cgi-bin/hgTracks?hgHubConnect.destUrl=..%2Fcgi-bin%2FhgTracks&clade=mammal&org=Human&db=hg19&position=DAPL1) | [TANC1 (147900)](http://genome.ucsc.edu/cgi-bin/hgTracks?hgHubConnect.destUrl=..%2Fcgi-bin%2FhgTracks&clade=mammal&org=Human&db=hg19&position=TANC1) | NA | NA | A/0.99/0.076/3.74e-06 | | A/0.982/0.066/0.00369 | | NA | | NA |
| rs148444376 | 2 |  | [DAPL1 (16969)](http://genome.ucsc.edu/cgi-bin/hgTracks?hgHubConnect.destUrl=..%2Fcgi-bin%2FhgTracks&clade=mammal&org=Human&db=hg19&position=DAPL1) | [TANC1 (135680)](http://genome.ucsc.edu/cgi-bin/hgTracks?hgHubConnect.destUrl=..%2Fcgi-bin%2FhgTracks&clade=mammal&org=Human&db=hg19&position=TANC1) | NA | NA | C/0.989/0.076/3.52e-06 | | C/0.982/0.066/0.00367 | | NA | | NA |
| rs148169454 | 2 |  | [DAPL1 (56561)](http://genome.ucsc.edu/cgi-bin/hgTracks?hgHubConnect.destUrl=..%2Fcgi-bin%2FhgTracks&clade=mammal&org=Human&db=hg19&position=DAPL1) | [TANC1 (96088)](http://genome.ucsc.edu/cgi-bin/hgTracks?hgHubConnect.destUrl=..%2Fcgi-bin%2FhgTracks&clade=mammal&org=Human&db=hg19&position=TANC1) | NA | NA | A/0.989/0.087/1.77e-06 | | A/0.983/0.072/0.00527 | | NA | | NA |
| rs184118729 | 2 |  | [DAPL1 (83041)](http://genome.ucsc.edu/cgi-bin/hgTracks?hgHubConnect.destUrl=..%2Fcgi-bin%2FhgTracks&clade=mammal&org=Human&db=hg19&position=DAPL1) | [TANC1 (69608)](http://genome.ucsc.edu/cgi-bin/hgTracks?hgHubConnect.destUrl=..%2Fcgi-bin%2FhgTracks&clade=mammal&org=Human&db=hg19&position=TANC1) | NA | NA | A/0.993/0.118/1.73e-07 | | A/0.989/0.12/0.00014 | | NA | | NA |
| rs115038670 | 2 | [KCNH7](http://genome.ucsc.edu/cgi-bin/hgTracks?hgHubConnect.destUrl=..%2Fcgi-bin%2FhgTracks&clade=mammal&org=Human&db=hg19&position=KCNH7) |  |  | NA | NA | T/0.992/0.118/4.07e-06 | | T/0.987/0.053/0.1302 | | NA | | NA |
| rs142206003 | 2 | [GULP1](http://genome.ucsc.edu/cgi-bin/hgTracks?hgHubConnect.destUrl=..%2Fcgi-bin%2FhgTracks&clade=mammal&org=Human&db=hg19&position=GULP1) |  |  | NA | NA | A/0.996/0.152/3.55e-06 | | A/0.993/0.06/0.1195 | | NA | | NA |
| rs147910279 | 2 | [GULP1](http://genome.ucsc.edu/cgi-bin/hgTracks?hgHubConnect.destUrl=..%2Fcgi-bin%2FhgTracks&clade=mammal&org=Human&db=hg19&position=GULP1) |  |  | NA | NA | T/0.997/0.151/3.82e-06 | | T/0.993/0.06/0.1207 | | NA | | NA |
| rs183770498 | 2 | [PARD3B](http://genome.ucsc.edu/cgi-bin/hgTracks?hgHubConnect.destUrl=..%2Fcgi-bin%2FhgTracks&clade=mammal&org=Human&db=hg19&position=PARD3B) |  |  | T/0.976/-0.005/0.6638 | T/0.977/0.003/0.8584 | T/0.967/-0.052/1.92e-07 | | T/0.979/-0.052/0.02924 | | A/0.971/-0.026/0.00016 | | A/0.976/0.006/0.5691 |
| rs141034416 | 2 | [DIRC3](http://genome.ucsc.edu/cgi-bin/hgTracks?hgHubConnect.destUrl=..%2Fcgi-bin%2FhgTracks&clade=mammal&org=Human&db=hg19&position=DIRC3) |  |  | NA | NA | T/0.998/0.181/1.5e-06 | | T/0.995/0.108/0.00993 | | NA | | NA |
| rs181284109 | 2 | [USP37](http://genome.ucsc.edu/cgi-bin/hgTracks?hgHubConnect.destUrl=..%2Fcgi-bin%2FhgTracks&clade=mammal&org=Human&db=hg19&position=USP37) | [VIL1 (31464)](http://genome.ucsc.edu/cgi-bin/hgTracks?hgHubConnect.destUrl=..%2Fcgi-bin%2FhgTracks&clade=mammal&org=Human&db=hg19&position=VIL1) | [RQCD1 (87965)](http://genome.ucsc.edu/cgi-bin/hgTracks?hgHubConnect.destUrl=..%2Fcgi-bin%2FhgTracks&clade=mammal&org=Human&db=hg19&position=RQCD1) | NA | NA | G/0.999/0.356/3.46e-06 | | G/0.998/0.121/0.1087 | | NA | | NA |
| rs189985091 | 2 |  |  |  | NA | NA | G/0.996/0.137/1.92e-06 | | G/0.991/0.083/0.01419 | | NA | | NA |
| rs141521978 | 2 | [SP100](http://genome.ucsc.edu/cgi-bin/hgTracks?hgHubConnect.destUrl=..%2Fcgi-bin%2FhgTracks&clade=mammal&org=Human&db=hg19&position=SP100) | [SP140L (43932)](http://genome.ucsc.edu/cgi-bin/hgTracks?hgHubConnect.destUrl=..%2Fcgi-bin%2FhgTracks&clade=mammal&org=Human&db=hg19&position=SP140L) |  | NA | NA | T/0.996/0.168/1.38e-06 | | T/0.993/0.072/0.07782 | | NA | | NA |
| rs184963741 | 2 | [SP100](http://genome.ucsc.edu/cgi-bin/hgTracks?hgHubConnect.destUrl=..%2Fcgi-bin%2FhgTracks&clade=mammal&org=Human&db=hg19&position=SP100) | [SP140L (65041)](http://genome.ucsc.edu/cgi-bin/hgTracks?hgHubConnect.destUrl=..%2Fcgi-bin%2FhgTracks&clade=mammal&org=Human&db=hg19&position=SP140L) | [LOC151475 (222149)](http://genome.ucsc.edu/cgi-bin/hgTracks?hgHubConnect.destUrl=..%2Fcgi-bin%2FhgTracks&clade=mammal&org=Human&db=hg19&position=LOC151475) | NA | NA | C/0.997/0.173/6.42e-07 | | C/0.993/0.075/0.06224 | | NA | | NA |
| rs144060362 | 2 |  | [CXCR7 (92728)](http://genome.ucsc.edu/cgi-bin/hgTracks?hgHubConnect.destUrl=..%2Fcgi-bin%2FhgTracks&clade=mammal&org=Human&db=hg19&position=CXCR7) |  | NA | NA | T/0.999/0.265/2.03e-06 | | T/0.997/0.139/0.01713 | | NA | | NA |
| rs150343078 | 3 | [CNTN4](http://genome.ucsc.edu/cgi-bin/hgTracks?hgHubConnect.destUrl=..%2Fcgi-bin%2FhgTracks&clade=mammal&org=Human&db=hg19&position=CNTN4) |  | [IL5RA (234834)](http://genome.ucsc.edu/cgi-bin/hgTracks?hgHubConnect.destUrl=..%2Fcgi-bin%2FhgTracks&clade=mammal&org=Human&db=hg19&position=IL5RA) | NA | NA | T/0.999/0.44/2.96e-07 | | T/0.998/0.301/0.00021 | | NA | | NA |
| rs148064379 | 3 |  |  |  | NA | NA | G/0.999/0.368/4.64e-08 | | G/0.997/0.191/0.00409 | | NA | | NA |
| rs147463808 | 3 |  |  |  | NA | NA | T/0.998/0.305/7.4e-08 | | T/0.996/0.188/0.00148 | | NA | | NA |
| rs143915539 | 3 | [C3orf24](http://genome.ucsc.edu/cgi-bin/hgTracks?hgHubConnect.destUrl=..%2Fcgi-bin%2FhgTracks&clade=mammal&org=Human&db=hg19&position=C3orf24) | [FANCD2 (45113)](http://genome.ucsc.edu/cgi-bin/hgTracks?hgHubConnect.destUrl=..%2Fcgi-bin%2FhgTracks&clade=mammal&org=Human&db=hg19&position=FANCD2) | [BRK1 (28973)](http://genome.ucsc.edu/cgi-bin/hgTracks?hgHubConnect.destUrl=..%2Fcgi-bin%2FhgTracks&clade=mammal&org=Human&db=hg19&position=BRK1) | NA | NA | T/0.986/0.075/3.34e-06 | | T/0.977/0.058/0.01323 | | NA | | NA |
| rs116554817 | 3 |  | [NUP210 (75307)](http://genome.ucsc.edu/cgi-bin/hgTracks?hgHubConnect.destUrl=..%2Fcgi-bin%2FhgTracks&clade=mammal&org=Human&db=hg19&position=NUP210) | [HDAC11 (49722)](http://genome.ucsc.edu/cgi-bin/hgTracks?hgHubConnect.destUrl=..%2Fcgi-bin%2FhgTracks&clade=mammal&org=Human&db=hg19&position=HDAC11) | NA | NA | A/0.998/0.246/3.56e-06 | | A/0.996/0.13/0.03739 | | NA | | NA |
| rs189158807 | 3 |  | [NUP210 (76624)](http://genome.ucsc.edu/cgi-bin/hgTracks?hgHubConnect.destUrl=..%2Fcgi-bin%2FhgTracks&clade=mammal&org=Human&db=hg19&position=NUP210) | [HDAC11 (48405)](http://genome.ucsc.edu/cgi-bin/hgTracks?hgHubConnect.destUrl=..%2Fcgi-bin%2FhgTracks&clade=mammal&org=Human&db=hg19&position=HDAC11) | NA | NA | T/0.998/0.246/3.58e-06 | | T/0.996/0.13/0.03749 | | NA | | NA |
| rs116126197 | 3 |  | [NUP210 (84840)](http://genome.ucsc.edu/cgi-bin/hgTracks?hgHubConnect.destUrl=..%2Fcgi-bin%2FhgTracks&clade=mammal&org=Human&db=hg19&position=NUP210) | [HDAC11 (40189)](http://genome.ucsc.edu/cgi-bin/hgTracks?hgHubConnect.destUrl=..%2Fcgi-bin%2FhgTracks&clade=mammal&org=Human&db=hg19&position=HDAC11) | NA | NA | A/0.997/0.246/4.03e-06 | | A/0.995/0.131/0.03668 | | NA | | NA |
| rs150765348 | 3 | [ZNF385D](http://genome.ucsc.edu/cgi-bin/hgTracks?hgHubConnect.destUrl=..%2Fcgi-bin%2FhgTracks&clade=mammal&org=Human&db=hg19&position=ZNF385D) |  |  | C/0.998/-0.038/0.4166 | C/0.998/0.002/0.9775 | C/1/0.426/2.15e-06 | | C/0.999/0.244/0.00373 | | NA | | T/0.998/0.07/0.1055 |
| rs181391108 | 3 |  |  |  | C/0.999/-0.084/0.1633 | C/1/-0.034/0.7529 | C/1/0.425/2.77e-06 | | C/0.999/0.24/0.00436 | | NA | | T/0.999/0.097/0.1233 |
| rs145287362 | 3 |  | [MIR548AC (106788)](http://genome.ucsc.edu/cgi-bin/hgTracks?hgHubConnect.destUrl=..%2Fcgi-bin%2FhgTracks&clade=mammal&org=Human&db=hg19&position=MIR548AC) | [UBE2E1 (91713)](http://genome.ucsc.edu/cgi-bin/hgTracks?hgHubConnect.destUrl=..%2Fcgi-bin%2FhgTracks&clade=mammal&org=Human&db=hg19&position=UBE2E1) | G/0.97/0.004/0.6842 | G/0.971/0.02/0.1148 | G/0.995/0.149/2.78e-08 | | G/0.99/0.085/0.01304 | | A/0.973/0.012/0.1114 | | A/0.972/0.004/0.6839 |
| rs187206368 | 3 |  |  | [RBMS3 (241540)](http://genome.ucsc.edu/cgi-bin/hgTracks?hgHubConnect.destUrl=..%2Fcgi-bin%2FhgTracks&clade=mammal&org=Human&db=hg19&position=RBMS3) | A/0.995/0.004/0.8759 | A/0.996/0.082/0.04104 | A/0.997/0.199/1.12e-06 | | A/0.994/0.155/0.00328 | | A/0.004/-0.054/0.0063 | | A/0.005/-0.046/0.07487 |
| rs115082929 | 3 |  | [ZNF621 (217869)](http://genome.ucsc.edu/cgi-bin/hgTracks?hgHubConnect.destUrl=..%2Fcgi-bin%2FhgTracks&clade=mammal&org=Human&db=hg19&position=ZNF621) |  | NA | NA | G/0.996/0.143/2.75e-06 | | G/0.992/0.147/0.00043 | | NA | | NA |
| rs116837984 | 3 | [ULK4](http://genome.ucsc.edu/cgi-bin/hgTracks?hgHubConnect.destUrl=..%2Fcgi-bin%2FhgTracks&clade=mammal&org=Human&db=hg19&position=ULK4) | [CTNNB1 (113408)](http://genome.ucsc.edu/cgi-bin/hgTracks?hgHubConnect.destUrl=..%2Fcgi-bin%2FhgTracks&clade=mammal&org=Human&db=hg19&position=CTNNB1) |  | NA | NA | A/0.994/0.121/2.45e-07 | | A/0.991/0.122/0.00016 | | NA | | NA |
| rs188003336 | 3 | [ULK4](http://genome.ucsc.edu/cgi-bin/hgTracks?hgHubConnect.destUrl=..%2Fcgi-bin%2FhgTracks&clade=mammal&org=Human&db=hg19&position=ULK4) | [CTNNB1 (155692)](http://genome.ucsc.edu/cgi-bin/hgTracks?hgHubConnect.destUrl=..%2Fcgi-bin%2FhgTracks&clade=mammal&org=Human&db=hg19&position=CTNNB1) |  | NA | NA | G/0.998/0.296/9.66e-08 | | G/0.996/0.135/0.01725 | | NA | | NA |
| rs148560677 | 3 | [ULK4](http://genome.ucsc.edu/cgi-bin/hgTracks?hgHubConnect.destUrl=..%2Fcgi-bin%2FhgTracks&clade=mammal&org=Human&db=hg19&position=ULK4) | [CTNNB1 (164651)](http://genome.ucsc.edu/cgi-bin/hgTracks?hgHubConnect.destUrl=..%2Fcgi-bin%2FhgTracks&clade=mammal&org=Human&db=hg19&position=CTNNB1) |  | NA | NA | C/0.998/0.289/1.33e-07 | | C/0.996/0.134/0.0176 | | NA | | NA |
| rs139606157 | 3 | [ULK4](http://genome.ucsc.edu/cgi-bin/hgTracks?hgHubConnect.destUrl=..%2Fcgi-bin%2FhgTracks&clade=mammal&org=Human&db=hg19&position=ULK4) |  |  | NA | NA | A/0.992/0.1/2.72e-06 | | A/0.989/0.106/0.00063 | | NA | | NA |
| rs190548655 | 3 |  | [TMEM158 (120337)](http://genome.ucsc.edu/cgi-bin/hgTracks?hgHubConnect.destUrl=..%2Fcgi-bin%2FhgTracks&clade=mammal&org=Human&db=hg19&position=TMEM158) | [LARS2 (41923)](http://genome.ucsc.edu/cgi-bin/hgTracks?hgHubConnect.destUrl=..%2Fcgi-bin%2FhgTracks&clade=mammal&org=Human&db=hg19&position=LARS2) | T/0.994/0.017/0.4872 | T/0.994/0.002/0.9474 | T/0.999/0.239/4.38e-06 | | T/0.997/0.172/0.0052 | | T/0.007/-0.038/0.01412 | | T/0.008/-0.01/0.573 |
| rs74498795 | 3 | [PRSS46](http://genome.ucsc.edu/cgi-bin/hgTracks?hgHubConnect.destUrl=..%2Fcgi-bin%2FhgTracks&clade=mammal&org=Human&db=hg19&position=PRSS46) | [PRSS50 (867)](http://genome.ucsc.edu/cgi-bin/hgTracks?hgHubConnect.destUrl=..%2Fcgi-bin%2FhgTracks&clade=mammal&org=Human&db=hg19&position=PRSS50) | [PRSS45 (7328)](http://genome.ucsc.edu/cgi-bin/hgTracks?hgHubConnect.destUrl=..%2Fcgi-bin%2FhgTracks&clade=mammal&org=Human&db=hg19&position=PRSS45) | NA | NA | G/0.999/0.224/3.12e-07 | | G/0.997/0.2/0.00013 | | NA | | NA |
| rs76770574 | 3 |  | [MYL3 (2945)](http://genome.ucsc.edu/cgi-bin/hgTracks?hgHubConnect.destUrl=..%2Fcgi-bin%2FhgTracks&clade=mammal&org=Human&db=hg19&position=MYL3) | [PTH1R (11317)](http://genome.ucsc.edu/cgi-bin/hgTracks?hgHubConnect.destUrl=..%2Fcgi-bin%2FhgTracks&clade=mammal&org=Human&db=hg19&position=PTH1R) | NA | NA | T/0.998/0.237/1.15e-07 | | T/0.997/0.202/0.00012 | | NA | | NA |
| rs144261207 | 3 |  | [MYL3 (5620)](http://genome.ucsc.edu/cgi-bin/hgTracks?hgHubConnect.destUrl=..%2Fcgi-bin%2FhgTracks&clade=mammal&org=Human&db=hg19&position=MYL3) | [PTH1R (8642)](http://genome.ucsc.edu/cgi-bin/hgTracks?hgHubConnect.destUrl=..%2Fcgi-bin%2FhgTracks&clade=mammal&org=Human&db=hg19&position=PTH1R) | A/0.994/-0.016/0.4431 | A/0.994/-0.053/0.04964 | A/0.997/0.179/1.55e-06 | | A/0.995/0.192/4.63e-05 | | A/0.005/-0.028/0.06209 | | A/0.005/0.011/0.55 |
| rs61734084 | 3 | [NBEAL2](http://genome.ucsc.edu/cgi-bin/hgTracks?hgHubConnect.destUrl=..%2Fcgi-bin%2FhgTracks&clade=mammal&org=Human&db=hg19&position=NBEAL2) | [CCDC12 (11960)](http://genome.ucsc.edu/cgi-bin/hgTracks?hgHubConnect.destUrl=..%2Fcgi-bin%2FhgTracks&clade=mammal&org=Human&db=hg19&position=CCDC12) | [NRADDP (22801)](http://genome.ucsc.edu/cgi-bin/hgTracks?hgHubConnect.destUrl=..%2Fcgi-bin%2FhgTracks&clade=mammal&org=Human&db=hg19&position=NRADDP) | NA | NA | G/0.998/0.224/4.56e-06 | | G/0.996/0.164/0.00451 | | NA | | NA |
| rs114496665 | 3 | [NBEAL2](http://genome.ucsc.edu/cgi-bin/hgTracks?hgHubConnect.destUrl=..%2Fcgi-bin%2FhgTracks&clade=mammal&org=Human&db=hg19&position=NBEAL2) | [CCDC12 (30705)](http://genome.ucsc.edu/cgi-bin/hgTracks?hgHubConnect.destUrl=..%2Fcgi-bin%2FhgTracks&clade=mammal&org=Human&db=hg19&position=CCDC12) | [NRADDP (4056)](http://genome.ucsc.edu/cgi-bin/hgTracks?hgHubConnect.destUrl=..%2Fcgi-bin%2FhgTracks&clade=mammal&org=Human&db=hg19&position=NRADDP) | NA | NA | C/0.999/0.246/2.19e-06 | | C/0.997/0.179/0.00256 | | NA | | NA |
| rs151074357 | 3 | [SETD2](http://genome.ucsc.edu/cgi-bin/hgTracks?hgHubConnect.destUrl=..%2Fcgi-bin%2FhgTracks&clade=mammal&org=Human&db=hg19&position=SETD2) | [NRADDP (21511)](http://genome.ucsc.edu/cgi-bin/hgTracks?hgHubConnect.destUrl=..%2Fcgi-bin%2FhgTracks&clade=mammal&org=Human&db=hg19&position=NRADDP) | [FLJ39534 (129391)](http://genome.ucsc.edu/cgi-bin/hgTracks?hgHubConnect.destUrl=..%2Fcgi-bin%2FhgTracks&clade=mammal&org=Human&db=hg19&position=FLJ39534) | NA | NA | C/0.999/0.24/3.32e-06 | | C/0.997/0.178/0.00269 | | NA | | NA |
| rs193058367 | 3 |  | [CACNA2D3 (16553)](http://genome.ucsc.edu/cgi-bin/hgTracks?hgHubConnect.destUrl=..%2Fcgi-bin%2FhgTracks&clade=mammal&org=Human&db=hg19&position=CACNA2D3) |  | NA | NA | A/0.998/0.199/1.84e-06 | | A/0.997/0.213/5.78e-05 | | NA | | NA |
| rs73075135 | 3 | [ERC2](http://genome.ucsc.edu/cgi-bin/hgTracks?hgHubConnect.destUrl=..%2Fcgi-bin%2FhgTracks&clade=mammal&org=Human&db=hg19&position=ERC2) |  |  | NA | NA | T/1/0.671/2.79e-07 | | T/0.999/0.34/0.00529 | | NA | | T/0.001/-0.1/0.2116 |
| rs191663403 | 3 |  |  |  | NA | NA | C/0.999/0.425/2.99e-07 | | C/0.998/0.43/4.85e-06 | | NA | | NA |
| rs182544594 | 3 |  |  |  | NA | NA | G/0.996/0.15/5.6e-07 | | G/0.991/0.064/0.0687 | | NA | | NA |
| rs182242981 | 3 |  |  |  | NA | NA | T/0.996/0.149/4.81e-07 | | T/0.991/0.065/0.06255 | | NA | | NA |
| rs188500549 | 3 | [MAGI1](http://genome.ucsc.edu/cgi-bin/hgTracks?hgHubConnect.destUrl=..%2Fcgi-bin%2FhgTracks&clade=mammal&org=Human&db=hg19&position=MAGI1) |  |  | A/0.995/-0.009/0.7381 | A/0.996/0.083/0.03048 | A/1/0.573/1.02e-06 | | A/0.999/0.41/0.00055 | | NA | | A/0.004/-0.068/0.04874 |
| rs146646693 | 3 |  | [FAM19A1 (117777)](http://genome.ucsc.edu/cgi-bin/hgTracks?hgHubConnect.destUrl=..%2Fcgi-bin%2FhgTracks&clade=mammal&org=Human&db=hg19&position=FAM19A1) |  | NA | NA | T/0.999/0.303/2.18e-06 | | T/0.997/0.176/0.01747 | | NA | | NA |
| rs143902882 | 3 | [ROBO2](http://genome.ucsc.edu/cgi-bin/hgTracks?hgHubConnect.destUrl=..%2Fcgi-bin%2FhgTracks&clade=mammal&org=Human&db=hg19&position=ROBO2) |  |  | NA | NA | T/0.993/0.106/2.64e-06 | | T/0.987/0.072/0.0121 | | NA | | NA |
| rs142772618 | 3 | [ROBO2](http://genome.ucsc.edu/cgi-bin/hgTracks?hgHubConnect.destUrl=..%2Fcgi-bin%2FhgTracks&clade=mammal&org=Human&db=hg19&position=ROBO2) |  |  | NA | NA | C/0.992/0.102/3.17e-06 | | C/0.986/0.072/0.0106 | | NA | | NA |
| rs192784693 | 3 |  |  |  | NA | NA | T/1/0.542/4.07e-06 | | T/0.999/0.435/0.00078 | | NA | | NA |
| rs145583199 | 3 | [CLDND1](http://genome.ucsc.edu/cgi-bin/hgTracks?hgHubConnect.destUrl=..%2Fcgi-bin%2FhgTracks&clade=mammal&org=Human&db=hg19&position=CLDND1) | [OR5K2 (19711)](http://genome.ucsc.edu/cgi-bin/hgTracks?hgHubConnect.destUrl=..%2Fcgi-bin%2FhgTracks&clade=mammal&org=Human&db=hg19&position=OR5K2) | [GPR15 (13691)](http://genome.ucsc.edu/cgi-bin/hgTracks?hgHubConnect.destUrl=..%2Fcgi-bin%2FhgTracks&clade=mammal&org=Human&db=hg19&position=GPR15) | NA | NA | C/0.999/0.41/2.94e-07 | | C/0.998/0.248/0.00222 | | NA | | NA |
| rs1585775 | 3 | [ZPLD1](http://genome.ucsc.edu/cgi-bin/hgTracks?hgHubConnect.destUrl=..%2Fcgi-bin%2FhgTracks&clade=mammal&org=Human&db=hg19&position=ZPLD1) |  |  | T/0.131/0.002/0.6697 | T/0.131/0.002/0.6386 | T/0.022/-0.052/1.19e-06 | | T/0.022/-0.058/0.00054 | | T/0.353/0.003/0.3973 | | T/0.496/-0.003/0.4118 |
| rs115371810 | 3 |  |  | [GAP43 (8186)](http://genome.ucsc.edu/cgi-bin/hgTracks?hgHubConnect.destUrl=..%2Fcgi-bin%2FhgTracks&clade=mammal&org=Human&db=hg19&position=GAP43) | NA | NA | G/1/0.484/2.83e-06 | | G/0.999/0.291/0.0024 | | NA | | NA |
| rs78274021 | 3 |  |  | [GAP43 (6631)](http://genome.ucsc.edu/cgi-bin/hgTracks?hgHubConnect.destUrl=..%2Fcgi-bin%2FhgTracks&clade=mammal&org=Human&db=hg19&position=GAP43) | NA | NA | T/0.998/0.27/1.74e-06 | | T/0.997/0.246/0.00029 | | NA | | NA |
| rs192210795 | 3 | [KALRN](http://genome.ucsc.edu/cgi-bin/hgTracks?hgHubConnect.destUrl=..%2Fcgi-bin%2FhgTracks&clade=mammal&org=Human&db=hg19&position=KALRN) | [DKFZp434B1222 (209130)](http://genome.ucsc.edu/cgi-bin/hgTracks?hgHubConnect.destUrl=..%2Fcgi-bin%2FhgTracks&clade=mammal&org=Human&db=hg19&position=DKFZp434B1222) |  | NA | NA | T/1/0.542/1.2e-06 | | T/0.999/0.288/0.00618 | | NA | | NA |
| rs143410069 | 3 | [KALRN](http://genome.ucsc.edu/cgi-bin/hgTracks?hgHubConnect.destUrl=..%2Fcgi-bin%2FhgTracks&clade=mammal&org=Human&db=hg19&position=KALRN) |  | [UMPS (176049)](http://genome.ucsc.edu/cgi-bin/hgTracks?hgHubConnect.destUrl=..%2Fcgi-bin%2FhgTracks&clade=mammal&org=Human&db=hg19&position=UMPS) | NA | NA | C/0.999/0.342/4.39e-07 | | C/0.998/0.178/0.00549 | | NA | | NA |
| rs151058966 | 3 | [KALRN](http://genome.ucsc.edu/cgi-bin/hgTracks?hgHubConnect.destUrl=..%2Fcgi-bin%2FhgTracks&clade=mammal&org=Human&db=hg19&position=KALRN) |  | [UMPS (173525)](http://genome.ucsc.edu/cgi-bin/hgTracks?hgHubConnect.destUrl=..%2Fcgi-bin%2FhgTracks&clade=mammal&org=Human&db=hg19&position=UMPS) | NA | NA | G/0.999/0.341/4.5e-07 | | G/0.998/0.178/0.00548 | | NA | | NA |
| rs150425940 | 3 |  | [GP9 (20936)](http://genome.ucsc.edu/cgi-bin/hgTracks?hgHubConnect.destUrl=..%2Fcgi-bin%2FhgTracks&clade=mammal&org=Human&db=hg19&position=GP9) | [ISY1-RAB43 (4222)](http://genome.ucsc.edu/cgi-bin/hgTracks?hgHubConnect.destUrl=..%2Fcgi-bin%2FhgTracks&clade=mammal&org=Human&db=hg19&position=ISY1-RAB43) | NA | NA | A/0.988/0.078/2.09e-06 | | A/0.982/0.084/0.00029 | | NA | | NA |
| rs140798517 | 3 | [RAB6B](http://genome.ucsc.edu/cgi-bin/hgTracks?hgHubConnect.destUrl=..%2Fcgi-bin%2FhgTracks&clade=mammal&org=Human&db=hg19&position=RAB6B) | [SRPRB (56792)](http://genome.ucsc.edu/cgi-bin/hgTracks?hgHubConnect.destUrl=..%2Fcgi-bin%2FhgTracks&clade=mammal&org=Human&db=hg19&position=SRPRB) | [C3orf36 (49861)](http://genome.ucsc.edu/cgi-bin/hgTracks?hgHubConnect.destUrl=..%2Fcgi-bin%2FhgTracks&clade=mammal&org=Human&db=hg19&position=C3orf36) | C/0.985/0.003/0.7644 | C/0.985/0.009/0.5596 | C/0.996/0.188/3.28e-06 | | C/0.994/0.192/0.00047 | | T/0.985/0.018/0.04124 | | T/0.984/0.004/0.6542 |
| rs143087368 | 3 |  | [RAB6B (14248)](http://genome.ucsc.edu/cgi-bin/hgTracks?hgHubConnect.destUrl=..%2Fcgi-bin%2FhgTracks&clade=mammal&org=Human&db=hg19&position=RAB6B) | [C3orf36 (18050)](http://genome.ucsc.edu/cgi-bin/hgTracks?hgHubConnect.destUrl=..%2Fcgi-bin%2FhgTracks&clade=mammal&org=Human&db=hg19&position=C3orf36) | G/0.987/0/0.9775 | G/0.987/-0.005/0.7458 | G/0.996/0.187/4.13e-06 | | G/0.994/0.191/0.00049 | | C/0.987/0.018/0.049 | | C/0.985/-0.001/0.9095 |
| rs112448655 | 3 | [CLSTN2](http://genome.ucsc.edu/cgi-bin/hgTracks?hgHubConnect.destUrl=..%2Fcgi-bin%2FhgTracks&clade=mammal&org=Human&db=hg19&position=CLSTN2) |  |  | A/0.99/0.006/0.6353 | A/0.99/0.025/0.1616 | A/0.997/0.163/3.12e-07 | | A/0.994/0.081/0.03283 | | A/0.009/-0.034/0.00124 | | A/0.011/-0.018/0.1364 |
| rs138465513 | 3 |  |  | [IQCJ (52245)](http://genome.ucsc.edu/cgi-bin/hgTracks?hgHubConnect.destUrl=..%2Fcgi-bin%2FhgTracks&clade=mammal&org=Human&db=hg19&position=IQCJ) | G/0.999/-0.029/0.6097 | G/0.999/-0.096/0.2202 | G/1/0.914/2.86e-06 | | G/0.999/0.514/0.00558 | | NA | | T/0.999/0/0.9991 |
| rs138869894 | 3 | [IQCJ](http://genome.ucsc.edu/cgi-bin/hgTracks?hgHubConnect.destUrl=..%2Fcgi-bin%2FhgTracks&clade=mammal&org=Human&db=hg19&position=IQCJ) |  | [SCHIP1 (146094)](http://genome.ucsc.edu/cgi-bin/hgTracks?hgHubConnect.destUrl=..%2Fcgi-bin%2FhgTracks&clade=mammal&org=Human&db=hg19&position=SCHIP1) | A/0.996/-0.031/0.2057 | A/0.996/-0.01/0.7926 | A/1/0.576/1.43e-06 | | A/0.999/0.333/0.00774 | | A/0.004/-0.007/0.707 | | A/0.004/-0.01/0.6531 |
| rs144152585 | 3 | [IQCJ](http://genome.ucsc.edu/cgi-bin/hgTracks?hgHubConnect.destUrl=..%2Fcgi-bin%2FhgTracks&clade=mammal&org=Human&db=hg19&position=IQCJ) |  | [SCHIP1 (78205)](http://genome.ucsc.edu/cgi-bin/hgTracks?hgHubConnect.destUrl=..%2Fcgi-bin%2FhgTracks&clade=mammal&org=Human&db=hg19&position=SCHIP1) | A/0.998/-0.042/0.2165 | A/0.998/-0.069/0.2099 | A/1/0.616/2.6e-06 | | A/0.999/0.369/0.00484 | | A/0.002/0/0.9882 | | A/0.002/0.013/0.7423 |
| rs141332792 | 3 | [IQCJ-SCHIP1](http://genome.ucsc.edu/cgi-bin/hgTracks?hgHubConnect.destUrl=..%2Fcgi-bin%2FhgTracks&clade=mammal&org=Human&db=hg19&position=IQCJ-SCHIP1) | [MIR3919 (11767)](http://genome.ucsc.edu/cgi-bin/hgTracks?hgHubConnect.destUrl=..%2Fcgi-bin%2FhgTracks&clade=mammal&org=Human&db=hg19&position=MIR3919) |  | G/0.997/-0.044/0.2077 | G/0.998/-0.069/0.2132 | G/1/0.631/2.2e-06 | | G/0.999/0.381/0.00425 | | NA | | A/0.998/-0.014/0.7355 |
| rs191765304 | 3 |  | [TRIM59 (8743)](http://genome.ucsc.edu/cgi-bin/hgTracks?hgHubConnect.destUrl=..%2Fcgi-bin%2FhgTracks&clade=mammal&org=Human&db=hg19&position=TRIM59) | [KPNA4 (36413)](http://genome.ucsc.edu/cgi-bin/hgTracks?hgHubConnect.destUrl=..%2Fcgi-bin%2FhgTracks&clade=mammal&org=Human&db=hg19&position=KPNA4) | NA | NA | T/1/0.634/2.68e-06 | | T/0.999/0.305/0.01535 | | NA | | NA |
| rs188694422 | 3 |  | [TRIM59 (8891)](http://genome.ucsc.edu/cgi-bin/hgTracks?hgHubConnect.destUrl=..%2Fcgi-bin%2FhgTracks&clade=mammal&org=Human&db=hg19&position=TRIM59) | [KPNA4 (36265)](http://genome.ucsc.edu/cgi-bin/hgTracks?hgHubConnect.destUrl=..%2Fcgi-bin%2FhgTracks&clade=mammal&org=Human&db=hg19&position=KPNA4) | NA | NA | A/1/0.633/2.67e-06 | | A/0.999/0.304/0.01538 | | NA | | NA |
| rs190230151 | 3 | [KPNA4](http://genome.ucsc.edu/cgi-bin/hgTracks?hgHubConnect.destUrl=..%2Fcgi-bin%2FhgTracks&clade=mammal&org=Human&db=hg19&position=KPNA4) | [SCARNA7 (13682)](http://genome.ucsc.edu/cgi-bin/hgTracks?hgHubConnect.destUrl=..%2Fcgi-bin%2FhgTracks&clade=mammal&org=Human&db=hg19&position=SCARNA7) | [BC125159 (38750)](http://genome.ucsc.edu/cgi-bin/hgTracks?hgHubConnect.destUrl=..%2Fcgi-bin%2FhgTracks&clade=mammal&org=Human&db=hg19&position=BC125159) | NA | NA | G/1/0.571/2.97e-06 | | G/0.999/0.277/0.01501 | | NA | | NA |
| rs190431552 | 3 |  | [ARL14 (23111)](http://genome.ucsc.edu/cgi-bin/hgTracks?hgHubConnect.destUrl=..%2Fcgi-bin%2FhgTracks&clade=mammal&org=Human&db=hg19&position=ARL14) | [PPM1L (54649)](http://genome.ucsc.edu/cgi-bin/hgTracks?hgHubConnect.destUrl=..%2Fcgi-bin%2FhgTracks&clade=mammal&org=Human&db=hg19&position=PPM1L) | NA | NA | G/1/0.457/2.52e-06 | | G/0.999/0.222/0.01396 | | NA | | NA |
| rs146844429 | 3 |  | [ARL14 (75520)](http://genome.ucsc.edu/cgi-bin/hgTracks?hgHubConnect.destUrl=..%2Fcgi-bin%2FhgTracks&clade=mammal&org=Human&db=hg19&position=ARL14) | [PPM1L (2240)](http://genome.ucsc.edu/cgi-bin/hgTracks?hgHubConnect.destUrl=..%2Fcgi-bin%2FhgTracks&clade=mammal&org=Human&db=hg19&position=PPM1L) | NA | NA | G/0.999/0.468/3.72e-07 | | G/0.998/0.212/0.01368 | | NA | | NA |
| rs186451686 | 3 | [PPM1L](http://genome.ucsc.edu/cgi-bin/hgTracks?hgHubConnect.destUrl=..%2Fcgi-bin%2FhgTracks&clade=mammal&org=Human&db=hg19&position=PPM1L) |  | [AK055323 (239084)](http://genome.ucsc.edu/cgi-bin/hgTracks?hgHubConnect.destUrl=..%2Fcgi-bin%2FhgTracks&clade=mammal&org=Human&db=hg19&position=AK055323) | NA | NA | A/1/0.454/2.32e-06 | | A/0.998/0.22/0.0139 | | NA | | NA |
| rs149794616 | 3 | [PPM1L](http://genome.ucsc.edu/cgi-bin/hgTracks?hgHubConnect.destUrl=..%2Fcgi-bin%2FhgTracks&clade=mammal&org=Human&db=hg19&position=PPM1L) |  | [AK055323 (237576)](http://genome.ucsc.edu/cgi-bin/hgTracks?hgHubConnect.destUrl=..%2Fcgi-bin%2FhgTracks&clade=mammal&org=Human&db=hg19&position=AK055323) | NA | NA | C/0.999/0.45/1.08e-06 | | C/0.998/0.208/0.01571 | | NA | | NA |
| rs188531091 | 3 | [PPM1L](http://genome.ucsc.edu/cgi-bin/hgTracks?hgHubConnect.destUrl=..%2Fcgi-bin%2FhgTracks&clade=mammal&org=Human&db=hg19&position=PPM1L) |  | [AK055323 (230124)](http://genome.ucsc.edu/cgi-bin/hgTracks?hgHubConnect.destUrl=..%2Fcgi-bin%2FhgTracks&clade=mammal&org=Human&db=hg19&position=AK055323) | NA | NA | G/1/0.454/2.33e-06 | | G/0.998/0.22/0.01395 | | NA | | NA |
| rs182750324 | 3 | [PPM1L](http://genome.ucsc.edu/cgi-bin/hgTracks?hgHubConnect.destUrl=..%2Fcgi-bin%2FhgTracks&clade=mammal&org=Human&db=hg19&position=PPM1L) |  | [AK055323 (190782)](http://genome.ucsc.edu/cgi-bin/hgTracks?hgHubConnect.destUrl=..%2Fcgi-bin%2FhgTracks&clade=mammal&org=Human&db=hg19&position=AK055323) | NA | NA | A/1/0.448/2.92e-06 | | A/0.999/0.218/0.01439 | | NA | | NA |
| rs145882615 | 3 | [PPM1L](http://genome.ucsc.edu/cgi-bin/hgTracks?hgHubConnect.destUrl=..%2Fcgi-bin%2FhgTracks&clade=mammal&org=Human&db=hg19&position=PPM1L) |  | [AK055323 (75653)](http://genome.ucsc.edu/cgi-bin/hgTracks?hgHubConnect.destUrl=..%2Fcgi-bin%2FhgTracks&clade=mammal&org=Human&db=hg19&position=AK055323) | NA | NA | G/1/0.435/4.09e-06 | | G/0.998/0.217/0.01429 | | NA | | NA |
| rs191390172 | 3 | [PPM1L](http://genome.ucsc.edu/cgi-bin/hgTracks?hgHubConnect.destUrl=..%2Fcgi-bin%2FhgTracks&clade=mammal&org=Human&db=hg19&position=PPM1L) |  | [AK055323 (27057)](http://genome.ucsc.edu/cgi-bin/hgTracks?hgHubConnect.destUrl=..%2Fcgi-bin%2FhgTracks&clade=mammal&org=Human&db=hg19&position=AK055323) | NA | NA | C/1/0.435/4.05e-06 | | C/0.998/0.216/0.01455 | | NA | | NA |
| rs192294496 | 3 |  | [B3GALNT1 (8414)](http://genome.ucsc.edu/cgi-bin/hgTracks?hgHubConnect.destUrl=..%2Fcgi-bin%2FhgTracks&clade=mammal&org=Human&db=hg19&position=B3GALNT1) | [NMD3 (107524)](http://genome.ucsc.edu/cgi-bin/hgTracks?hgHubConnect.destUrl=..%2Fcgi-bin%2FhgTracks&clade=mammal&org=Human&db=hg19&position=NMD3) | NA | NA | T/1/0.449/3.01e-06 | | T/0.999/0.218/0.01488 | | NA | | NA |
| rs187303806 | 3 |  | [B3GALNT1 (58315)](http://genome.ucsc.edu/cgi-bin/hgTracks?hgHubConnect.destUrl=..%2Fcgi-bin%2FhgTracks&clade=mammal&org=Human&db=hg19&position=B3GALNT1) | [NMD3 (57623)](http://genome.ucsc.edu/cgi-bin/hgTracks?hgHubConnect.destUrl=..%2Fcgi-bin%2FhgTracks&clade=mammal&org=Human&db=hg19&position=NMD3) | NA | NA | C/1/0.432/4.72e-06 | | C/0.998/0.214/0.01501 | | NA | | NA |
| rs140014224 | 3 | [NMD3](http://genome.ucsc.edu/cgi-bin/hgTracks?hgHubConnect.destUrl=..%2Fcgi-bin%2FhgTracks&clade=mammal&org=Human&db=hg19&position=NMD3) | [B3GALNT1 (133614)](http://genome.ucsc.edu/cgi-bin/hgTracks?hgHubConnect.destUrl=..%2Fcgi-bin%2FhgTracks&clade=mammal&org=Human&db=hg19&position=B3GALNT1) | [SPTSSB (105805)](http://genome.ucsc.edu/cgi-bin/hgTracks?hgHubConnect.destUrl=..%2Fcgi-bin%2FhgTracks&clade=mammal&org=Human&db=hg19&position=SPTSSB) | NA | NA | G/1/0.436/4.33e-06 | | G/0.999/0.216/0.01514 | | NA | | NA |
| rs142560558 | 3 |  | [NMD3 (69887)](http://genome.ucsc.edu/cgi-bin/hgTracks?hgHubConnect.destUrl=..%2Fcgi-bin%2FhgTracks&clade=mammal&org=Human&db=hg19&position=NMD3) | [SPTSSB (21372)](http://genome.ucsc.edu/cgi-bin/hgTracks?hgHubConnect.destUrl=..%2Fcgi-bin%2FhgTracks&clade=mammal&org=Human&db=hg19&position=SPTSSB) | NA | NA | G/1/0.44/3.49e-06 | | G/0.998/0.218/0.01477 | | NA | | NA |
| rs192376484 | 3 |  | [NMD3 (84495)](http://genome.ucsc.edu/cgi-bin/hgTracks?hgHubConnect.destUrl=..%2Fcgi-bin%2FhgTracks&clade=mammal&org=Human&db=hg19&position=NMD3) | [SPTSSB (6764)](http://genome.ucsc.edu/cgi-bin/hgTracks?hgHubConnect.destUrl=..%2Fcgi-bin%2FhgTracks&clade=mammal&org=Human&db=hg19&position=SPTSSB) | NA | NA | A/1/0.445/1.92e-06 | | A/0.998/0.211/0.01532 | | NA | | NA |
| rs190957092 | 3 |  |  |  | C/0.997/-0.061/0.129 | C/0.997/-0.093/0.07955 | C/0.999/0.425/2.32e-07 | | C/0.998/0.113/0.1482 | | T/0.997/0.014/0.627 | | T/0.997/-0.027/0.4085 |
| rs147508746 | 3 |  | [DQ571917 (211542)](http://genome.ucsc.edu/cgi-bin/hgTracks?hgHubConnect.destUrl=..%2Fcgi-bin%2FhgTracks&clade=mammal&org=Human&db=hg19&position=DQ571917) |  | C/0.983/0.018/0.1223 | C/0.983/0.049/0.00163 | C/0.997/0.165/4.66e-07 | | C/0.992/0.054/0.1481 | | A/0.984/0.024/0.01057 | | A/0.983/0.023/0.04036 |
| rs142255501 | 3 |  | [DQ571917 (214612)](http://genome.ucsc.edu/cgi-bin/hgTracks?hgHubConnect.destUrl=..%2Fcgi-bin%2FhgTracks&clade=mammal&org=Human&db=hg19&position=DQ571917) |  | A/0.983/0.018/0.1227 | A/0.983/0.049/0.00166 | A/0.997/0.164/4.48e-07 | | A/0.992/0.054/0.1529 | | A/0.016/-0.024/0.01074 | | A/0.017/-0.023/0.0409 |
| rs114946066 | 3 | [TRA2B](http://genome.ucsc.edu/cgi-bin/hgTracks?hgHubConnect.destUrl=..%2Fcgi-bin%2FhgTracks&clade=mammal&org=Human&db=hg19&position=TRA2B) | [IGF2BP2 (110270)](http://genome.ucsc.edu/cgi-bin/hgTracks?hgHubConnect.destUrl=..%2Fcgi-bin%2FhgTracks&clade=mammal&org=Human&db=hg19&position=IGF2BP2) | [LOC344887 (24660)](http://genome.ucsc.edu/cgi-bin/hgTracks?hgHubConnect.destUrl=..%2Fcgi-bin%2FhgTracks&clade=mammal&org=Human&db=hg19&position=LOC344887) | C/0.976/-0.006/0.6425 | C/0.978/0.011/0.5462 | C/0.993/0.139/6.33e-07 | | C/0.988/0.067/0.03672 | | T/0.979/0.017/0.07579 | | T/0.977/0.003/0.7788 |
| rs138641425 | 3 | [FGF12](http://genome.ucsc.edu/cgi-bin/hgTracks?hgHubConnect.destUrl=..%2Fcgi-bin%2FhgTracks&clade=mammal&org=Human&db=hg19&position=FGF12) |  |  | NA | NA | A/0.999/0.389/1.98e-06 | | A/0.997/0.188/0.01912 | | NA | | NA |
| rs149667740 | 3 | [FGF12](http://genome.ucsc.edu/cgi-bin/hgTracks?hgHubConnect.destUrl=..%2Fcgi-bin%2FhgTracks&clade=mammal&org=Human&db=hg19&position=FGF12) |  |  | NA | NA | G/0.999/0.362/1.54e-06 | | G/0.997/0.168/0.02291 | | NA | | NA |
| rs189326995 | 3 | [FGF12](http://genome.ucsc.edu/cgi-bin/hgTracks?hgHubConnect.destUrl=..%2Fcgi-bin%2FhgTracks&clade=mammal&org=Human&db=hg19&position=FGF12) |  |  | NA | NA | C/0.999/0.443/7.99e-07 | | C/0.998/0.256/0.00296 | | NA | | NA |
| rs180919520 | 4 |  | [FAM193A (5073)](http://genome.ucsc.edu/cgi-bin/hgTracks?hgHubConnect.destUrl=..%2Fcgi-bin%2FhgTracks&clade=mammal&org=Human&db=hg19&position=FAM193A) | [TNIP2 (4011)](http://genome.ucsc.edu/cgi-bin/hgTracks?hgHubConnect.destUrl=..%2Fcgi-bin%2FhgTracks&clade=mammal&org=Human&db=hg19&position=TNIP2) | NA | NA | C/0.997/0.218/4.92e-06 | | C/0.994/0.105/0.06069 | | NA | | NA |
| rs190569495 | 4 | [LOC100507266](http://genome.ucsc.edu/cgi-bin/hgTracks?hgHubConnect.destUrl=..%2Fcgi-bin%2FhgTracks&clade=mammal&org=Human&db=hg19&position=LOC100507266) | [STX18 (113767)](http://genome.ucsc.edu/cgi-bin/hgTracks?hgHubConnect.destUrl=..%2Fcgi-bin%2FhgTracks&clade=mammal&org=Human&db=hg19&position=STX18) | [AK056081 (105989)](http://genome.ucsc.edu/cgi-bin/hgTracks?hgHubConnect.destUrl=..%2Fcgi-bin%2FhgTracks&clade=mammal&org=Human&db=hg19&position=AK056081) | T/0.99/-0.006/0.7014 | T/0.992/0.003/0.9115 | T/0.999/0.296/2.29e-06 | | T/0.997/0.168/0.01032 | | T/0.01/-0.02/0.1015 | | T/0.011/-0.009/0.5373 |
| rs191540052 | 4 |  | [MSX1 (21454)](http://genome.ucsc.edu/cgi-bin/hgTracks?hgHubConnect.destUrl=..%2Fcgi-bin%2FhgTracks&clade=mammal&org=Human&db=hg19&position=MSX1) | [CYTL1 (129199)](http://genome.ucsc.edu/cgi-bin/hgTracks?hgHubConnect.destUrl=..%2Fcgi-bin%2FhgTracks&clade=mammal&org=Human&db=hg19&position=CYTL1) | NA | NA | A/0.999/0.271/4.37e-06 | | A/0.997/0.193/0.00166 | | NA | | NA |
| rs76225479 | 4 |  | [MSX1 (39686)](http://genome.ucsc.edu/cgi-bin/hgTracks?hgHubConnect.destUrl=..%2Fcgi-bin%2FhgTracks&clade=mammal&org=Human&db=hg19&position=MSX1) | [CYTL1 (110967)](http://genome.ucsc.edu/cgi-bin/hgTracks?hgHubConnect.destUrl=..%2Fcgi-bin%2FhgTracks&clade=mammal&org=Human&db=hg19&position=CYTL1) | NA | NA | G/0.997/0.224/3.72e-07 | | G/0.994/0.115/0.01781 | | NA | | A/0.98/0.009/0.6591 |
| rs188006861 | 4 | [AFAP1](http://genome.ucsc.edu/cgi-bin/hgTracks?hgHubConnect.destUrl=..%2Fcgi-bin%2FhgTracks&clade=mammal&org=Human&db=hg19&position=AFAP1) | [AFAP1-AS1 (70189)](http://genome.ucsc.edu/cgi-bin/hgTracks?hgHubConnect.destUrl=..%2Fcgi-bin%2FhgTracks&clade=mammal&org=Human&db=hg19&position=AFAP1-AS1) | [ABLIM2 (116193)](http://genome.ucsc.edu/cgi-bin/hgTracks?hgHubConnect.destUrl=..%2Fcgi-bin%2FhgTracks&clade=mammal&org=Human&db=hg19&position=ABLIM2) | NA | NA | A/0.991/0.096/9.41e-07 | | A/0.984/0.076/0.00426 | | NA | | NA |
| rs150450027 | 4 | [LOC152742](http://genome.ucsc.edu/cgi-bin/hgTracks?hgHubConnect.destUrl=..%2Fcgi-bin%2FhgTracks&clade=mammal&org=Human&db=hg19&position=LOC152742) |  |  | NA | NA | T/1/0.424/1.81e-07 | | T/0.998/0.23/0.00222 | | NA | | NA |
| rs181995593 | 4 | [KCNIP4](http://genome.ucsc.edu/cgi-bin/hgTracks?hgHubConnect.destUrl=..%2Fcgi-bin%2FhgTracks&clade=mammal&org=Human&db=hg19&position=KCNIP4) |  |  | NA | NA | C/0.998/0.224/4.3e-06 | | C/0.997/0.141/0.01083 | | NA | | NA |
| rs138355804 | 4 |  |  |  | T/0.989/-0.007/0.633 | T/0.989/-0.027/0.1719 | T/0.998/0.291/4.57e-06 | | T/0.997/0.199/0.00573 | | T/0.011/-0.006/0.6198 | | T/0.012/0.015/0.237 |
| rs184665953 | 4 |  | [GABRA2 (52938)](http://genome.ucsc.edu/cgi-bin/hgTracks?hgHubConnect.destUrl=..%2Fcgi-bin%2FhgTracks&clade=mammal&org=Human&db=hg19&position=GABRA2) |  | G/0.995/0.013/0.5648 | G/0.995/-0.012/0.6689 | G/0.999/0.281/3.43e-06 | | G/0.997/0.118/0.0357 | | T/0.995/0.045/0.00414 | | T/0.994/0.028/0.09792 |
| rs193113980 | 4 | [CHIC2](http://genome.ucsc.edu/cgi-bin/hgTracks?hgHubConnect.destUrl=..%2Fcgi-bin%2FhgTracks&clade=mammal&org=Human&db=hg19&position=CHIC2) |  | [GSX2 (76864)](http://genome.ucsc.edu/cgi-bin/hgTracks?hgHubConnect.destUrl=..%2Fcgi-bin%2FhgTracks&clade=mammal&org=Human&db=hg19&position=GSX2) | NA | NA | T/0.998/0.297/1.83e-06 | | T/0.996/0.168/0.00689 | | NA | | NA |
| rs182823009 | 4 | [PDGFRA](http://genome.ucsc.edu/cgi-bin/hgTracks?hgHubConnect.destUrl=..%2Fcgi-bin%2FhgTracks&clade=mammal&org=Human&db=hg19&position=PDGFRA) | [CHIC2 (9127)](http://genome.ucsc.edu/cgi-bin/hgTracks?hgHubConnect.destUrl=..%2Fcgi-bin%2FhgTracks&clade=mammal&org=Human&db=hg19&position=CHIC2) |  | NA | NA | C/0.999/0.332/2.74e-06 | | C/0.997/0.178/0.00852 | | NA | | NA |
| rs192046469 | 4 | [PDGFRA](http://genome.ucsc.edu/cgi-bin/hgTracks?hgHubConnect.destUrl=..%2Fcgi-bin%2FhgTracks&clade=mammal&org=Human&db=hg19&position=PDGFRA) | [CHIC2 (13928)](http://genome.ucsc.edu/cgi-bin/hgTracks?hgHubConnect.destUrl=..%2Fcgi-bin%2FhgTracks&clade=mammal&org=Human&db=hg19&position=CHIC2) |  | NA | NA | T/0.999/0.336/1.12e-06 | | T/0.997/0.178/0.00713 | | NA | | NA |
| rs190972371 | 4 | [SPINK2](http://genome.ucsc.edu/cgi-bin/hgTracks?hgHubConnect.destUrl=..%2Fcgi-bin%2FhgTracks&clade=mammal&org=Human&db=hg19&position=SPINK2) | [HOPX (157722)](http://genome.ucsc.edu/cgi-bin/hgTracks?hgHubConnect.destUrl=..%2Fcgi-bin%2FhgTracks&clade=mammal&org=Human&db=hg19&position=HOPX) | [REST (93631)](http://genome.ucsc.edu/cgi-bin/hgTracks?hgHubConnect.destUrl=..%2Fcgi-bin%2FhgTracks&clade=mammal&org=Human&db=hg19&position=REST) | NA | NA | C/0.998/0.22/1.31e-06 | | C/0.996/0.149/0.00356 | | NA | | NA |
| rs190057637 | 4 |  |  |  | T/0.996/0.036/0.2404 | T/0.996/-0.002/0.9616 | T/0.999/0.426/4.39e-06 | | T/0.998/0.229/0.00985 | | T/0.004/-0.041/0.08087 | | T/0.004/-0.002/0.9553 |
| rs145688504 | 4 | [GRID2](http://genome.ucsc.edu/cgi-bin/hgTracks?hgHubConnect.destUrl=..%2Fcgi-bin%2FhgTracks&clade=mammal&org=Human&db=hg19&position=GRID2) |  |  | NA | NA | T/0.999/0.412/4.8e-06 | | T/0.998/0.206/0.0176 | | NA | | NA |
| rs117802898 | 4 | [GRID2](http://genome.ucsc.edu/cgi-bin/hgTracks?hgHubConnect.destUrl=..%2Fcgi-bin%2FhgTracks&clade=mammal&org=Human&db=hg19&position=GRID2) |  |  | NA | NA | C/0.998/0.181/3.05e-06 | | C/0.994/0.122/0.00515 | | NA | | NA |
| rs143324395 | 4 | [GRID2](http://genome.ucsc.edu/cgi-bin/hgTracks?hgHubConnect.destUrl=..%2Fcgi-bin%2FhgTracks&clade=mammal&org=Human&db=hg19&position=GRID2) |  |  | NA | NA | G/0.997/0.18/2.6e-06 | | G/0.994/0.121/0.00522 | | NA | | NA |
| rs117932668 | 4 | [GRID2](http://genome.ucsc.edu/cgi-bin/hgTracks?hgHubConnect.destUrl=..%2Fcgi-bin%2FhgTracks&clade=mammal&org=Human&db=hg19&position=GRID2) |  |  | NA | NA | A/0.997/0.177/3.74e-06 | | A/0.994/0.121/0.0053 | | NA | | NA |
| rs72674100 | 4 |  |  |  | A/0.918/0.001/0.8926 | A/0.915/-0.003/0.6047 | A/0.943/0.035/1.69e-06 | | A/0.928/0.023/0.05898 | | A/0.076/-0.006/0.06902 | | A/0.083/0.005/0.2324 |
| rs11934983 | 4 |  |  |  | G/0.528/0.001/0.7767 | G/0.526/-0.002/0.5399 | C/0.692/0.016/3.12e-06 | | C/0.663/0.026/6.04e-05 | | C/0.473/-0.003/0.09533 | | C/0.53/-0.002/0.3124 |
| rs144910173 | 4 |  |  |  | A/0.502/0/0.8715 | A/0.501/-0.002/0.643 | C/0.7/0.017/4.79e-06 | | C/0.674/0.025/0.00023 | | A/0.545/0.003/0.13 | | A/0.496/0.002/0.4584 |
| rs960450 | 4 |  |  |  | C/0.465/-0.001/0.7754 | C/0.465/0.002/0.6035 | T/0.318/-0.016/2.41e-06 | | T/0.318/-0.025/8.56e-05 | | T/0.453/0.003/0.1028 | | T/0.493/0.002/0.3731 |
| rs139367725 | 4 | [C4orf37](http://genome.ucsc.edu/cgi-bin/hgTracks?hgHubConnect.destUrl=..%2Fcgi-bin%2FhgTracks&clade=mammal&org=Human&db=hg19&position=C4orf37) |  |  | G/0.528/0.001/0.8116 | G/0.526/-0.002/0.5241 | C/0.69/0.017/1.41e-06 | | C/0.659/0.027/2.97e-05 | | C/0.473/-0.003/0.06981 | | C/0.53/-0.002/0.3248 |
| rs12642926 | 4 | [C4orf37](http://genome.ucsc.edu/cgi-bin/hgTracks?hgHubConnect.destUrl=..%2Fcgi-bin%2FhgTracks&clade=mammal&org=Human&db=hg19&position=C4orf37) |  |  | G/0.53/0.001/0.7869 | G/0.528/-0.002/0.5376 | A/0.69/0.017/1.28e-06 | | A/0.66/0.026/5.34e-05 | | A/0.474/-0.003/0.07976 | | A/0.531/-0.002/0.311 |
| rs28766878 | 4 | [C4orf37](http://genome.ucsc.edu/cgi-bin/hgTracks?hgHubConnect.destUrl=..%2Fcgi-bin%2FhgTracks&clade=mammal&org=Human&db=hg19&position=C4orf37) |  |  | T/0.474/0.001/0.8213 | T/0.477/-0.002/0.6148 | C/0.688/0.017/2.99e-06 | | C/0.662/0.025/0.00018 | | T/0.562/0.003/0.127 | | T/0.518/0.001/0.5981 |
| rs72889099 | 4 | [C4orf37](http://genome.ucsc.edu/cgi-bin/hgTracks?hgHubConnect.destUrl=..%2Fcgi-bin%2FhgTracks&clade=mammal&org=Human&db=hg19&position=C4orf37) |  |  | G/0.993/0.022/0.1737 | G/0.992/0.028/0.1539 | G/0.559/-0.015/4.82e-06 | | G/0.586/-0.019/0.00291 | | A/0.586/-0.013/6.87e-05 | | A/0.993/0.009/0.5451 |
| rs1911792 | 4 | [C4orf37](http://genome.ucsc.edu/cgi-bin/hgTracks?hgHubConnect.destUrl=..%2Fcgi-bin%2FhgTracks&clade=mammal&org=Human&db=hg19&position=C4orf37) |  |  | A/0.531/0/0.8999 | A/0.53/-0.002/0.5133 | G/0.678/0.016/4.7e-06 | | G/0.654/0.025/0.00012 | | A/0.522/0.003/0.0855 | | A/0.466/0.002/0.4168 |
| rs72889102 | 4 | [C4orf37](http://genome.ucsc.edu/cgi-bin/hgTracks?hgHubConnect.destUrl=..%2Fcgi-bin%2FhgTracks&clade=mammal&org=Human&db=hg19&position=C4orf37) |  |  | G/0.993/0.022/0.174 | G/0.992/0.028/0.1541 | G/0.559/-0.015/4.81e-06 | | G/0.587/-0.019/0.0029 | | C/0.586/-0.013/6.87e-05 | | C/0.993/0.009/0.5451 |
| rs1532993 | 4 | [C4orf37](http://genome.ucsc.edu/cgi-bin/hgTracks?hgHubConnect.destUrl=..%2Fcgi-bin%2FhgTracks&clade=mammal&org=Human&db=hg19&position=C4orf37) |  |  | T/0.587/0/0.9195 | T/0.586/-0.003/0.383 | C/0.668/0.016/3.47e-06 | | C/0.644/0.023/0.00024 | | T/0.48/0.004/0.04617 | | T/0.411/0.003/0.2651 |
| rs1508459 | 4 | [C4orf37](http://genome.ucsc.edu/cgi-bin/hgTracks?hgHubConnect.destUrl=..%2Fcgi-bin%2FhgTracks&clade=mammal&org=Human&db=hg19&position=C4orf37) |  |  | G/0.993/0.022/0.1741 | G/0.992/0.028/0.1541 | G/0.559/-0.015/4.82e-06 | | G/0.587/-0.019/0.0029 | | A/0.586/-0.013/6.87e-05 | | A/0.993/0.009/0.5451 |
| rs1567287 | 4 | [C4orf37](http://genome.ucsc.edu/cgi-bin/hgTracks?hgHubConnect.destUrl=..%2Fcgi-bin%2FhgTracks&clade=mammal&org=Human&db=hg19&position=C4orf37) |  |  | C/0.581/-0.001/0.7777 | C/0.581/-0.004/0.3225 | T/0.67/0.016/2.9e-06 | | T/0.645/0.024/0.00022 | | T/0.516/-0.004/0.04097 | | T/0.584/-0.003/0.2534 |
| rs17026828 | 4 | [C4orf37](http://genome.ucsc.edu/cgi-bin/hgTracks?hgHubConnect.destUrl=..%2Fcgi-bin%2FhgTracks&clade=mammal&org=Human&db=hg19&position=C4orf37) |  |  | A/0.993/0.022/0.1741 | A/0.992/0.028/0.1541 | A/0.562/-0.016/4.07e-06 | | A/0.59/-0.02/0.00174 | | A/0.411/0.013/5.4e-05 | | A/0.007/-0.009/0.5451 |
| rs61456001 | 4 | [C4orf37](http://genome.ucsc.edu/cgi-bin/hgTracks?hgHubConnect.destUrl=..%2Fcgi-bin%2FhgTracks&clade=mammal&org=Human&db=hg19&position=C4orf37) |  |  | G/0.993/0.022/0.1742 | G/0.992/0.028/0.1541 | G/0.559/-0.015/4.83e-06 | | G/0.587/-0.019/0.0029 | | A/0.586/-0.013/6.87e-05 | | A/0.993/0.009/0.5451 |
| rs56880249 | 4 | [C4orf37](http://genome.ucsc.edu/cgi-bin/hgTracks?hgHubConnect.destUrl=..%2Fcgi-bin%2FhgTracks&clade=mammal&org=Human&db=hg19&position=C4orf37) |  |  | C/0.993/0.022/0.1794 | C/0.992/0.028/0.1549 | C/0.56/-0.015/4.99e-06 | | C/0.587/-0.019/0.00272 | | A/0.587/-0.013/6.76e-05 | | A/0.993/0.008/0.5475 |
| rs61154600 | 4 | [C4orf37](http://genome.ucsc.edu/cgi-bin/hgTracks?hgHubConnect.destUrl=..%2Fcgi-bin%2FhgTracks&clade=mammal&org=Human&db=hg19&position=C4orf37) |  |  | T/0.993/0.022/0.1795 | T/0.992/0.028/0.155 | T/0.56/-0.015/4.98e-06 | | T/0.587/-0.019/0.00271 | | T/0.413/0.013/6.77e-05 | | T/0.007/-0.008/0.5475 |
| rs58276078 | 4 | [C4orf37](http://genome.ucsc.edu/cgi-bin/hgTracks?hgHubConnect.destUrl=..%2Fcgi-bin%2FhgTracks&clade=mammal&org=Human&db=hg19&position=C4orf37) |  |  | C/0.993/0.022/0.1795 | C/0.992/0.028/0.155 | C/0.56/-0.015/4.98e-06 | | C/0.587/-0.019/0.00271 | | T/0.587/-0.013/6.77e-05 | | T/0.993/0.008/0.5475 |
| rs7662978 | 4 | [C4orf37](http://genome.ucsc.edu/cgi-bin/hgTracks?hgHubConnect.destUrl=..%2Fcgi-bin%2FhgTracks&clade=mammal&org=Human&db=hg19&position=C4orf37) |  |  | C/0.987/0.025/0.06669 | C/0.985/0.024/0.1556 | C/0.558/-0.015/4.72e-06 | | C/0.586/-0.019/0.00248 | | T/0.595/-0.013/8.86e-05 | | T/0.986/0.003/0.8246 |
| rs72890850 | 4 | [C4orf37](http://genome.ucsc.edu/cgi-bin/hgTracks?hgHubConnect.destUrl=..%2Fcgi-bin%2FhgTracks&clade=mammal&org=Human&db=hg19&position=C4orf37) |  |  | G/0.988/0.017/0.1653 | G/0.987/0.01/0.5156 | G/0.57/-0.017/1.2e-06 | | G/0.6/-0.02/0.00166 | | A/0.616/-0.013/2.81e-05 | | A/0.987/-0.001/0.9178 |
| rs72890852 | 4 | [C4orf37](http://genome.ucsc.edu/cgi-bin/hgTracks?hgHubConnect.destUrl=..%2Fcgi-bin%2FhgTracks&clade=mammal&org=Human&db=hg19&position=C4orf37) |  |  | T/0.993/0.019/0.2247 | T/0.992/0.028/0.1583 | T/0.565/-0.016/2.3e-06 | | T/0.595/-0.019/0.00314 | | T/0.41/0.014/1.87e-05 | | T/0.007/-0.008/0.5513 |
| rs76142193 | 4 | [C4orf37](http://genome.ucsc.edu/cgi-bin/hgTracks?hgHubConnect.destUrl=..%2Fcgi-bin%2FhgTracks&clade=mammal&org=Human&db=hg19&position=C4orf37) |  |  | A/0.988/0.016/0.1801 | A/0.987/0.01/0.5172 | A/0.564/-0.016/2.96e-06 | | A/0.593/-0.019/0.00291 | | A/0.391/0.013/4e-05 | | A/0.013/0.001/0.9235 |
| rs78681061 | 4 | [C4orf37](http://genome.ucsc.edu/cgi-bin/hgTracks?hgHubConnect.destUrl=..%2Fcgi-bin%2FhgTracks&clade=mammal&org=Human&db=hg19&position=C4orf37) |  |  | A/0.99/0.02/0.1952 | A/0.989/0.025/0.2023 | A/0.624/-0.016/4.52e-06 | | A/0.648/-0.018/0.00687 | | A/0.352/0.013/5.8e-05 | | A/0.01/-0.01/0.486 |
| rs7676966 | 4 | [C4orf37](http://genome.ucsc.edu/cgi-bin/hgTracks?hgHubConnect.destUrl=..%2Fcgi-bin%2FhgTracks&clade=mammal&org=Human&db=hg19&position=C4orf37) |  |  | C/0.987/0.015/0.2206 | C/0.986/0.011/0.4721 | C/0.562/-0.015/4.96e-06 | | C/0.59/-0.018/0.0036 | | T/0.607/-0.012/7.46e-05 | | T/0.986/-0.001/0.9579 |
| rs55901496 | 4 | [C4orf37](http://genome.ucsc.edu/cgi-bin/hgTracks?hgHubConnect.destUrl=..%2Fcgi-bin%2FhgTracks&clade=mammal&org=Human&db=hg19&position=C4orf37) |  |  | G/0.993/0.019/0.235 | G/0.992/0.028/0.1587 | G/0.563/-0.015/3.3e-06 | | G/0.592/-0.018/0.00391 | | A/0.588/-0.013/3.5e-05 | | A/0.993/0.008/0.5513 |
| rs17026847 | 4 | [C4orf37](http://genome.ucsc.edu/cgi-bin/hgTracks?hgHubConnect.destUrl=..%2Fcgi-bin%2FhgTracks&clade=mammal&org=Human&db=hg19&position=C4orf37) |  |  | T/0.988/0.016/0.1865 | T/0.987/0.01/0.5175 | T/0.562/-0.015/4.54e-06 | | T/0.59/-0.018/0.00372 | | T/0.393/0.012/8.11e-05 | | T/0.013/0.001/0.9252 |
| rs17026850 | 4 | [C4orf37](http://genome.ucsc.edu/cgi-bin/hgTracks?hgHubConnect.destUrl=..%2Fcgi-bin%2FhgTracks&clade=mammal&org=Human&db=hg19&position=C4orf37) |  |  | C/0.993/0.019/0.2352 | C/0.992/0.028/0.1587 | C/0.568/-0.016/2.4e-06 | | C/0.596/-0.018/0.00388 | | T/0.593/-0.014/2.09e-05 | | T/0.993/0.008/0.5513 |
| rs2136082 | 4 | [C4orf37](http://genome.ucsc.edu/cgi-bin/hgTracks?hgHubConnect.destUrl=..%2Fcgi-bin%2FhgTracks&clade=mammal&org=Human&db=hg19&position=C4orf37) |  |  | T/0.993/0.019/0.2353 | T/0.992/0.028/0.1587 | T/0.625/-0.016/3.57e-06 | | T/0.649/-0.018/0.00746 | | T/0.368/0.014/1.95e-05 | | T/0.26/-0.003/0.837 |
| rs2078362 | 4 | [C4orf37](http://genome.ucsc.edu/cgi-bin/hgTracks?hgHubConnect.destUrl=..%2Fcgi-bin%2FhgTracks&clade=mammal&org=Human&db=hg19&position=C4orf37) |  |  | C/0.993/0.019/0.2356 | C/0.992/0.028/0.1588 | C/0.563/-0.015/3.3e-06 | | C/0.592/-0.018/0.00392 | | A/0.588/-0.013/3.49e-05 | | A/0.993/0.008/0.5513 |
| rs60217712 | 4 | [C4orf37](http://genome.ucsc.edu/cgi-bin/hgTracks?hgHubConnect.destUrl=..%2Fcgi-bin%2FhgTracks&clade=mammal&org=Human&db=hg19&position=C4orf37) |  |  | T/0.993/0.019/0.2382 | T/0.992/0.028/0.1589 | T/0.563/-0.015/3.28e-06 | | T/0.592/-0.018/0.00392 | | A/0.588/-0.013/3.47e-05 | | A/0.993/0.008/0.5513 |
| rs142026888 | 4 | [C4orf37](http://genome.ucsc.edu/cgi-bin/hgTracks?hgHubConnect.destUrl=..%2Fcgi-bin%2FhgTracks&clade=mammal&org=Human&db=hg19&position=C4orf37) |  |  | C/0.984/-0.002/0.8926 | C/0.985/0.028/0.08015 | C/0.598/-0.016/1.65e-06 | | C/0.624/-0.02/0.00192 | | T/0.641/-0.015/3.68e-06 | | T/0.985/0.001/0.9513 |
| rs148460633 | 4 | [C4orf37](http://genome.ucsc.edu/cgi-bin/hgTracks?hgHubConnect.destUrl=..%2Fcgi-bin%2FhgTracks&clade=mammal&org=Human&db=hg19&position=C4orf37) |  |  | G/0.993/0.019/0.239 | G/0.992/0.028/0.1589 | G/0.63/-0.017/1.42e-06 | | G/0.655/-0.019/0.00394 | | A/0.653/-0.014/1.3e-05 | | A/0.993/0.008/0.5513 |
| rs144250695 | 4 | [C4orf37](http://genome.ucsc.edu/cgi-bin/hgTracks?hgHubConnect.destUrl=..%2Fcgi-bin%2FhgTracks&clade=mammal&org=Human&db=hg19&position=C4orf37) |  |  | G/0.993/0.018/0.2451 | G/0.992/0.028/0.1592 | G/0.567/-0.015/3.12e-06 | | G/0.596/-0.017/0.00454 | | A/0.592/-0.013/3.05e-05 | | A/0.993/0.008/0.557 |
| rs147576484 | 4 | [C4orf37](http://genome.ucsc.edu/cgi-bin/hgTracks?hgHubConnect.destUrl=..%2Fcgi-bin%2FhgTracks&clade=mammal&org=Human&db=hg19&position=C4orf37) |  |  | T/0.993/0.018/0.2452 | T/0.992/0.028/0.1592 | T/0.625/-0.016/3.59e-06 | | T/0.65/-0.017/0.00862 | | T/0.352/0.014/3.17e-05 | | T/0.007/-0.008/0.557 |
| rs59901031 | 4 | [C4orf37](http://genome.ucsc.edu/cgi-bin/hgTracks?hgHubConnect.destUrl=..%2Fcgi-bin%2FhgTracks&clade=mammal&org=Human&db=hg19&position=C4orf37) |  |  | G/0.988/0.016/0.1928 | G/0.987/0.01/0.5178 | G/0.566/-0.015/4.24e-06 | | G/0.593/-0.018/0.00252 | | A/0.61/-0.012/7.97e-05 | | A/0.987/-0.001/0.9242 |
| rs1012333 | 4 | [C4orf37](http://genome.ucsc.edu/cgi-bin/hgTracks?hgHubConnect.destUrl=..%2Fcgi-bin%2FhgTracks&clade=mammal&org=Human&db=hg19&position=C4orf37) |  |  | C/0.993/0.018/0.2458 | C/0.992/0.028/0.1593 | C/0.624/-0.016/3.26e-06 | | C/0.649/-0.017/0.00779 | | T/0.648/-0.014/3.17e-05 | | T/0.993/0.008/0.557 |
| rs59602504 | 4 | [C4orf37](http://genome.ucsc.edu/cgi-bin/hgTracks?hgHubConnect.destUrl=..%2Fcgi-bin%2FhgTracks&clade=mammal&org=Human&db=hg19&position=C4orf37) |  |  | G/0.988/0.016/0.193 | G/0.987/0.01/0.5176 | G/0.562/-0.015/4.38e-06 | | G/0.59/-0.018/0.00393 | | A/0.607/-0.012/8.98e-05 | | A/0.987/-0.001/0.9242 |
| rs59810062 | 4 | [C4orf37](http://genome.ucsc.edu/cgi-bin/hgTracks?hgHubConnect.destUrl=..%2Fcgi-bin%2FhgTracks&clade=mammal&org=Human&db=hg19&position=C4orf37) |  |  | A/0.993/0.018/0.2461 | A/0.992/0.028/0.1593 | A/0.563/-0.015/3.09e-06 | | A/0.592/-0.018/0.00409 | | A/0.411/0.013/3.47e-05 | | A/0.007/-0.008/0.5577 |
| rs72890876 | 4 | [C4orf37](http://genome.ucsc.edu/cgi-bin/hgTracks?hgHubConnect.destUrl=..%2Fcgi-bin%2FhgTracks&clade=mammal&org=Human&db=hg19&position=C4orf37) |  |  | C/0.993/0.018/0.2467 | C/0.992/0.028/0.1593 | C/0.624/-0.016/3.23e-06 | | C/0.649/-0.017/0.00781 | | A/0.648/-0.014/3.17e-05 | | A/0.993/0.008/0.5577 |
| rs56945629 | 4 | [C4orf37](http://genome.ucsc.edu/cgi-bin/hgTracks?hgHubConnect.destUrl=..%2Fcgi-bin%2FhgTracks&clade=mammal&org=Human&db=hg19&position=C4orf37) |  |  | T/0.988/0.016/0.1937 | T/0.987/0.01/0.5175 | T/0.562/-0.015/4.4e-06 | | T/0.59/-0.018/0.00396 | | T/0.393/0.012/8.9e-05 | | T/0.013/0.001/0.9242 |
| rs59606962 | 4 | [C4orf37](http://genome.ucsc.edu/cgi-bin/hgTracks?hgHubConnect.destUrl=..%2Fcgi-bin%2FhgTracks&clade=mammal&org=Human&db=hg19&position=C4orf37) |  |  | T/0.988/0.016/0.1937 | T/0.987/0.01/0.5175 | T/0.562/-0.015/4.4e-06 | | T/0.59/-0.018/0.00396 | | T/0.393/0.012/8.9e-05 | | T/0.013/0.001/0.9242 |
| rs72890889 | 4 | [C4orf37](http://genome.ucsc.edu/cgi-bin/hgTracks?hgHubConnect.destUrl=..%2Fcgi-bin%2FhgTracks&clade=mammal&org=Human&db=hg19&position=C4orf37) |  |  | A/0.988/0.016/0.1938 | A/0.987/0.01/0.5174 | A/0.562/-0.015/4.28e-06 | | A/0.591/-0.018/0.00394 | | A/0.393/0.012/8.9e-05 | | A/0.013/0.001/0.9242 |
| rs72892812 | 4 | [C4orf37](http://genome.ucsc.edu/cgi-bin/hgTracks?hgHubConnect.destUrl=..%2Fcgi-bin%2FhgTracks&clade=mammal&org=Human&db=hg19&position=C4orf37) |  |  | C/0.988/0.015/0.2066 | C/0.987/0.01/0.5389 | C/0.618/-0.015/4.24e-06 | | C/0.647/-0.017/0.00867 | | C/0.343/0.013/5.3e-05 | | C/0.013/0.001/0.902 |
| rs59172540 | 4 | [C4orf37](http://genome.ucsc.edu/cgi-bin/hgTracks?hgHubConnect.destUrl=..%2Fcgi-bin%2FhgTracks&clade=mammal&org=Human&db=hg19&position=C4orf37) |  |  | G/0.988/0.015/0.2093 | G/0.987/0.009/0.5436 | G/0.614/-0.015/4.59e-06 | | G/0.643/-0.017/0.00702 | | A/0.653/-0.012/5.94e-05 | | A/0.987/-0.001/0.8985 |
| rs58864230 | 4 | [C4orf37](http://genome.ucsc.edu/cgi-bin/hgTracks?hgHubConnect.destUrl=..%2Fcgi-bin%2FhgTracks&clade=mammal&org=Human&db=hg19&position=C4orf37) |  |  | T/0.988/0.015/0.2104 | T/0.987/0.009/0.5455 | T/0.614/-0.015/4.69e-06 | | T/0.643/-0.017/0.00706 | | T/0.347/0.012/5.94e-05 | | T/0.013/0.001/0.8985 |
| rs60804385 | 4 | [C4orf37](http://genome.ucsc.edu/cgi-bin/hgTracks?hgHubConnect.destUrl=..%2Fcgi-bin%2FhgTracks&clade=mammal&org=Human&db=hg19&position=C4orf37) |  |  | C/0.988/0.015/0.2266 | C/0.987/0.009/0.5728 | C/0.614/-0.015/4.55e-06 | | C/0.643/-0.017/0.00711 | | T/0.653/-0.013/5.63e-05 | | T/0.987/-0.002/0.8742 |
| rs57750317 | 4 | [C4orf37](http://genome.ucsc.edu/cgi-bin/hgTracks?hgHubConnect.destUrl=..%2Fcgi-bin%2FhgTracks&clade=mammal&org=Human&db=hg19&position=C4orf37) |  |  | G/0.988/0.015/0.2283 | G/0.987/0.009/0.5757 | G/0.614/-0.015/4.53e-06 | | G/0.643/-0.017/0.00711 | | C/0.653/-0.013/5.63e-05 | | C/0.987/-0.002/0.8742 |
| rs17026855 | 4 | [C4orf37](http://genome.ucsc.edu/cgi-bin/hgTracks?hgHubConnect.destUrl=..%2Fcgi-bin%2FhgTracks&clade=mammal&org=Human&db=hg19&position=C4orf37) |  |  | A/0.993/0.017/0.2779 | A/0.992/0.027/0.1795 | A/0.618/-0.016/2.36e-06 | | A/0.648/-0.017/0.00893 | | A/0.359/0.014/1.9e-05 | | A/0.007/-0.008/0.59 |
| rs58111058 | 4 | [C4orf37](http://genome.ucsc.edu/cgi-bin/hgTracks?hgHubConnect.destUrl=..%2Fcgi-bin%2FhgTracks&clade=mammal&org=Human&db=hg19&position=C4orf37) |  |  | G/0.988/0.025/0.08759 | G/0.987/0.03/0.1035 | G/0.622/-0.015/4.07e-06 | | G/0.651/-0.017/0.00897 | | T/0.648/-0.013/7.02e-05 | | T/0.987/0.014/0.2801 |
| rs61012000 | 4 | [C4orf37](http://genome.ucsc.edu/cgi-bin/hgTracks?hgHubConnect.destUrl=..%2Fcgi-bin%2FhgTracks&clade=mammal&org=Human&db=hg19&position=C4orf37) |  |  | T/0.993/0.016/0.3091 | T/0.992/0.025/0.2094 | T/0.622/-0.015/4.31e-06 | | T/0.651/-0.016/0.01357 | | T/0.357/0.013/3.39e-05 | | T/0.007/-0.007/0.635 |
| rs58724568 | 4 | [C4orf37](http://genome.ucsc.edu/cgi-bin/hgTracks?hgHubConnect.destUrl=..%2Fcgi-bin%2FhgTracks&clade=mammal&org=Human&db=hg19&position=C4orf37) |  |  | A/0.993/0.016/0.324 | A/0.992/0.025/0.2169 | A/0.618/-0.015/3.76e-06 | | A/0.648/-0.015/0.01595 | | A/0.36/0.013/2.87e-05 | | A/0.007/-0.007/0.6479 |
| rs72877580 | 4 | [C4orf37](http://genome.ucsc.edu/cgi-bin/hgTracks?hgHubConnect.destUrl=..%2Fcgi-bin%2FhgTracks&clade=mammal&org=Human&db=hg19&position=C4orf37) |  |  | G/0.993/0.016/0.3251 | G/0.992/0.025/0.2175 | G/0.653/-0.017/8.06e-07 | | G/0.685/-0.018/0.00852 | | A/0.675/-0.015/1.13e-05 | | A/0.993/0.006/0.6533 |
| rs7658560 | 4 | [C4orf37](http://genome.ucsc.edu/cgi-bin/hgTracks?hgHubConnect.destUrl=..%2Fcgi-bin%2FhgTracks&clade=mammal&org=Human&db=hg19&position=C4orf37) |  |  | T/0.993/0.016/0.3288 | T/0.992/0.025/0.2194 | T/0.626/-0.015/4.94e-06 | | T/0.656/-0.016/0.01466 | | T/0.352/0.013/2.85e-05 | | T/0.007/-0.006/0.6576 |
| rs7658798 | 4 | [C4orf37](http://genome.ucsc.edu/cgi-bin/hgTracks?hgHubConnect.destUrl=..%2Fcgi-bin%2FhgTracks&clade=mammal&org=Human&db=hg19&position=C4orf37) |  |  | G/0.993/0.016/0.329 | G/0.992/0.025/0.2186 | G/0.626/-0.015/4.95e-06 | | G/0.656/-0.016/0.01469 | | A/0.648/-0.013/2.85e-05 | | A/0.993/0.006/0.6551 |
| rs61095879 | 4 | [AK094561](http://genome.ucsc.edu/cgi-bin/hgTracks?hgHubConnect.destUrl=..%2Fcgi-bin%2FhgTracks&clade=mammal&org=Human&db=hg19&position=AK094561) | [CXXC4 (163043)](http://genome.ucsc.edu/cgi-bin/hgTracks?hgHubConnect.destUrl=..%2Fcgi-bin%2FhgTracks&clade=mammal&org=Human&db=hg19&position=CXXC4) |  | NA | NA | G/0.992/0.09/2.16e-06 | | G/0.984/0.041/0.09855 | | NA | | NA |
| rs73839333 | 4 |  | [AK094561 (50757)](http://genome.ucsc.edu/cgi-bin/hgTracks?hgHubConnect.destUrl=..%2Fcgi-bin%2FhgTracks&clade=mammal&org=Human&db=hg19&position=AK094561) |  | NA | NA | A/0.991/0.09/1.5e-06 | | A/0.984/0.044/0.07493 | | NA | | NA |
| rs73839334 | 4 |  | [AK094561 (54193)](http://genome.ucsc.edu/cgi-bin/hgTracks?hgHubConnect.destUrl=..%2Fcgi-bin%2FhgTracks&clade=mammal&org=Human&db=hg19&position=AK094561) |  | NA | NA | A/0.991/0.09/1.54e-06 | | A/0.984/0.044/0.07572 | | NA | | NA |
| rs181310918 | 4 |  |  |  | NA | NA | C/0.99/0.082/3.41e-06 | | C/0.982/0.043/0.07948 | | NA | | NA |
| rs77285130 | 4 |  |  |  | NA | NA | T/0.994/0.108/6.41e-07 | | T/0.988/0.054/0.05387 | | NA | | NA |
| rs78736651 | 4 |  |  |  | A/1/0.161/0.06719 | A/1/0.158/0.1028 | A/0.988/0.079/2.67e-06 | | A/0.98/0.038/0.1043 | | A/0.01/-0.065/2.81e-05 | | A/0.001/-0.038/0.3832 |
| rs73836173 | 4 |  |  |  | NA | NA | A/0.993/0.102/6.47e-07 | | A/0.986/0.048/0.06791 | | NA | | NA |
| rs73836179 | 4 |  |  |  | G/0.997/0.012/0.6993 | G/0.997/0.054/0.1883 | G/0.988/0.074/3.15e-06 | | G/0.979/0.035/0.1139 | | A/0.99/0.058/9.86e-06 | | A/0.997/0.043/0.1048 |
| rs73838632 | 4 |  |  |  | NA | NA | G/0.988/0.073/3.97e-06 | | G/0.979/0.034/0.1262 | | NA | | NA |
| rs73838634 | 4 |  |  |  | NA | NA | G/0.987/0.072/3.4e-06 | | G/0.978/0.03/0.1595 | | NA | | NA |
| rs56404102 | 4 |  |  |  | NA | NA | C/0.987/0.072/3.35e-06 | | C/0.978/0.03/0.1609 | | NA | | NA |
| rs73838636 | 4 |  |  |  | NA | NA | A/0.988/0.073/2.62e-06 | | A/0.979/0.03/0.1708 | | NA | | NA |
| rs73838638 | 4 |  |  |  | NA | NA | G/0.994/0.104/1.17e-06 | | G/0.988/0.05/0.07421 | | NA | | NA |
| rs73838643 | 4 |  |  | [TET2 (244472)](http://genome.ucsc.edu/cgi-bin/hgTracks?hgHubConnect.destUrl=..%2Fcgi-bin%2FhgTracks&clade=mammal&org=Human&db=hg19&position=TET2) | NA | NA | T/0.988/0.073/2.52e-06 | | T/0.979/0.029/0.1735 | | NA | | NA |
| rs73838644 | 4 |  |  | [TET2 (241897)](http://genome.ucsc.edu/cgi-bin/hgTracks?hgHubConnect.destUrl=..%2Fcgi-bin%2FhgTracks&clade=mammal&org=Human&db=hg19&position=TET2) | NA | NA | A/0.988/0.073/2.52e-06 | | A/0.979/0.029/0.1728 | | NA | | NA |
| rs192264222 | 4 |  |  | [TET2 (241581)](http://genome.ucsc.edu/cgi-bin/hgTracks?hgHubConnect.destUrl=..%2Fcgi-bin%2FhgTracks&clade=mammal&org=Human&db=hg19&position=TET2) | NA | NA | T/0.988/0.073/2.51e-06 | | T/0.979/0.029/0.1727 | | NA | | NA |
| rs73838646 | 4 |  |  | [TET2 (232533)](http://genome.ucsc.edu/cgi-bin/hgTracks?hgHubConnect.destUrl=..%2Fcgi-bin%2FhgTracks&clade=mammal&org=Human&db=hg19&position=TET2) | NA | NA | C/0.988/0.073/2.56e-06 | | C/0.979/0.03/0.1625 | | NA | | NA |
| rs147515761 | 4 |  |  | [TET2 (223112)](http://genome.ucsc.edu/cgi-bin/hgTracks?hgHubConnect.destUrl=..%2Fcgi-bin%2FhgTracks&clade=mammal&org=Human&db=hg19&position=TET2) | NA | NA | C/0.987/0.074/2.05e-06 | | C/0.978/0.031/0.1529 | | NA | | NA |
| rs61068760 | 4 |  |  | [TET2 (215155)](http://genome.ucsc.edu/cgi-bin/hgTracks?hgHubConnect.destUrl=..%2Fcgi-bin%2FhgTracks&clade=mammal&org=Human&db=hg19&position=TET2) | NA | NA | G/0.987/0.079/9.46e-07 | | G/0.978/0.035/0.1119 | | NA | | NA |
| rs76187983 | 4 |  |  | [TET2 (213909)](http://genome.ucsc.edu/cgi-bin/hgTracks?hgHubConnect.destUrl=..%2Fcgi-bin%2FhgTracks&clade=mammal&org=Human&db=hg19&position=TET2) | NA | NA | G/0.987/0.079/9.39e-07 | | G/0.978/0.035/0.1114 | | NA | | NA |
| rs73838651 | 4 |  |  | [TET2 (199116)](http://genome.ucsc.edu/cgi-bin/hgTracks?hgHubConnect.destUrl=..%2Fcgi-bin%2FhgTracks&clade=mammal&org=Human&db=hg19&position=TET2) | NA | NA | C/0.989/0.087/4.32e-07 | | C/0.981/0.04/0.08746 | | NA | | NA |
| rs73838652 | 4 |  |  | [TET2 (195581)](http://genome.ucsc.edu/cgi-bin/hgTracks?hgHubConnect.destUrl=..%2Fcgi-bin%2FhgTracks&clade=mammal&org=Human&db=hg19&position=TET2) | NA | NA | A/0.989/0.088/3.87e-07 | | A/0.981/0.041/0.08608 | | NA | | NA |
| rs73838653 | 4 |  |  | [TET2 (195306)](http://genome.ucsc.edu/cgi-bin/hgTracks?hgHubConnect.destUrl=..%2Fcgi-bin%2FhgTracks&clade=mammal&org=Human&db=hg19&position=TET2) | NA | NA | A/0.989/0.088/3.88e-07 | | A/0.981/0.041/0.0858 | | NA | | NA |
| rs56235007 | 4 |  |  | [TET2 (193248)](http://genome.ucsc.edu/cgi-bin/hgTracks?hgHubConnect.destUrl=..%2Fcgi-bin%2FhgTracks&clade=mammal&org=Human&db=hg19&position=TET2) | NA | NA | C/0.989/0.088/3.87e-07 | | C/0.981/0.041/0.08507 | | NA | | NA |
| rs73838654 | 4 |  |  | [TET2 (192487)](http://genome.ucsc.edu/cgi-bin/hgTracks?hgHubConnect.destUrl=..%2Fcgi-bin%2FhgTracks&clade=mammal&org=Human&db=hg19&position=TET2) | NA | NA | C/0.989/0.088/3.86e-07 | | C/0.981/0.041/0.08463 | | NA | | NA |
| rs73838655 | 4 |  |  | [TET2 (192445)](http://genome.ucsc.edu/cgi-bin/hgTracks?hgHubConnect.destUrl=..%2Fcgi-bin%2FhgTracks&clade=mammal&org=Human&db=hg19&position=TET2) | NA | NA | C/0.989/0.088/3.86e-07 | | C/0.981/0.041/0.08463 | | NA | | NA |
| rs74341662 | 4 |  |  | [TET2 (187837)](http://genome.ucsc.edu/cgi-bin/hgTracks?hgHubConnect.destUrl=..%2Fcgi-bin%2FhgTracks&clade=mammal&org=Human&db=hg19&position=TET2) | NA | NA | G/0.988/0.086/4.08e-07 | | G/0.979/0.046/0.04803 | | NA | | NA |
| rs77556290 | 4 |  |  | [TET2 (186281)](http://genome.ucsc.edu/cgi-bin/hgTracks?hgHubConnect.destUrl=..%2Fcgi-bin%2FhgTracks&clade=mammal&org=Human&db=hg19&position=TET2) | A/0.996/0.005/0.8187 | A/0.996/0.047/0.1098 | A/0.986/0.074/3.79e-06 | | A/0.978/0.041/0.07245 | | A/0.01/-0.045/0.00019 | | A/0.004/-0.058/0.00856 |
| rs73838658 | 4 |  |  | [TET2 (179723)](http://genome.ucsc.edu/cgi-bin/hgTracks?hgHubConnect.destUrl=..%2Fcgi-bin%2FhgTracks&clade=mammal&org=Human&db=hg19&position=TET2) | NA | NA | T/0.988/0.086/3.75e-07 | | T/0.979/0.046/0.04725 | | NA | | NA |
| rs73838071 | 4 | [TET2](http://genome.ucsc.edu/cgi-bin/hgTracks?hgHubConnect.destUrl=..%2Fcgi-bin%2FhgTracks&clade=mammal&org=Human&db=hg19&position=TET2) |  | [PPA2 (93256)](http://genome.ucsc.edu/cgi-bin/hgTracks?hgHubConnect.destUrl=..%2Fcgi-bin%2FhgTracks&clade=mammal&org=Human&db=hg19&position=PPA2) | NA | NA | A/0.997/0.154/2.72e-06 | | A/0.994/0.095/0.02121 | | NA | | NA |
| rs186822321 | 4 |  |  |  | NA | NA | A/0.999/0.424/2.44e-06 | | A/0.998/0.267/0.00154 | | NA | | A/0.004/0.039/0.4643 |
| rs185438855 | 4 |  |  |  | G/0.998/0.004/0.9163 | G/0.998/-0.025/0.6176 | G/0.999/0.372/2.23e-06 | | G/0.998/0.213/0.01134 | | A/0.998/0.054/0.08216 | | A/0.998/0.001/0.9783 |
| rs186699656 | 4 | [LOC100505545](http://genome.ucsc.edu/cgi-bin/hgTracks?hgHubConnect.destUrl=..%2Fcgi-bin%2FhgTracks&clade=mammal&org=Human&db=hg19&position=LOC100505545) |  | [LSM6 (55891)](http://genome.ucsc.edu/cgi-bin/hgTracks?hgHubConnect.destUrl=..%2Fcgi-bin%2FhgTracks&clade=mammal&org=Human&db=hg19&position=LSM6) | NA | NA | T/0.999/0.239/1.15e-06 | | T/0.996/0.139/0.00633 | | NA | | NA |
| rs141644443 | 4 |  | [TTC29 (109690)](http://genome.ucsc.edu/cgi-bin/hgTracks?hgHubConnect.destUrl=..%2Fcgi-bin%2FhgTracks&clade=mammal&org=Human&db=hg19&position=TTC29) |  | NA | NA | G/1/0.559/3.47e-08 | | G/0.999/0.338/0.00033 | | NA | | NA |
| rs192691827 | 4 |  | [CTSO (4651)](http://genome.ucsc.edu/cgi-bin/hgTracks?hgHubConnect.destUrl=..%2Fcgi-bin%2FhgTracks&clade=mammal&org=Human&db=hg19&position=CTSO) |  | NA | NA | G/0.992/0.09/3.15e-06 | | G/0.988/0.08/0.00544 | | NA | | NA |
| rs145368269 | 4 |  | [CTSO (12141)](http://genome.ucsc.edu/cgi-bin/hgTracks?hgHubConnect.destUrl=..%2Fcgi-bin%2FhgTracks&clade=mammal&org=Human&db=hg19&position=CTSO) |  | NA | NA | C/0.993/0.092/2.57e-06 | | C/0.989/0.079/0.00615 | | NA | | NA |
| rs183470003 | 4 | [MARCH1](http://genome.ucsc.edu/cgi-bin/hgTracks?hgHubConnect.destUrl=..%2Fcgi-bin%2FhgTracks&clade=mammal&org=Human&db=hg19&position=MARCH1) |  |  | T/0.991/0.002/0.8633 | T/0.991/-0.009/0.6539 | T/0.999/0.231/3.94e-06 | | T/0.996/0.118/0.01961 | | T/0.008/-0.012/0.32 | | T/0.009/0.005/0.6951 |
| rs2859815 | 4 |  |  | [TLL1 (121573)](http://genome.ucsc.edu/cgi-bin/hgTracks?hgHubConnect.destUrl=..%2Fcgi-bin%2FhgTracks&clade=mammal&org=Human&db=hg19&position=TLL1) | C/0.876/0.001/0.8114 | C/0.882/0.006/0.3024 | C/0.826/0.021/4.96e-06 | | C/0.795/0.01/0.1943 | | C/0.139/-0.01/0.00032 | | C/0.12/-0.007/0.05155 |
| rs185609610 | 4 | [AK094945](http://genome.ucsc.edu/cgi-bin/hgTracks?hgHubConnect.destUrl=..%2Fcgi-bin%2FhgTracks&clade=mammal&org=Human&db=hg19&position=AK094945) | [AGA (85903)](http://genome.ucsc.edu/cgi-bin/hgTracks?hgHubConnect.destUrl=..%2Fcgi-bin%2FhgTracks&clade=mammal&org=Human&db=hg19&position=AGA) |  | NA | NA | G/0.999/0.331/4.59e-06 | | G/0.998/0.142/0.03309 | | NA | | NA |
| rs145613323 | 5 |  | [BC032469 (10954)](http://genome.ucsc.edu/cgi-bin/hgTracks?hgHubConnect.destUrl=..%2Fcgi-bin%2FhgTracks&clade=mammal&org=Human&db=hg19&position=BC032469) | [SLC6A19 (12035)](http://genome.ucsc.edu/cgi-bin/hgTracks?hgHubConnect.destUrl=..%2Fcgi-bin%2FhgTracks&clade=mammal&org=Human&db=hg19&position=SLC6A19) | NA | NA | A/0.999/0.396/4.54e-06 | | A/0.998/0.167/0.04136 | | NA | | NA |
| rs186953828 | 5 |  |  | [MIR4454 (72975)](http://genome.ucsc.edu/cgi-bin/hgTracks?hgHubConnect.destUrl=..%2Fcgi-bin%2FhgTracks&clade=mammal&org=Human&db=hg19&position=MIR4454) | A/0.789/-0.002/0.5954 | A/0.79/-0.002/0.6112 | A/0.729/-0.018/4.83e-06 | | A/0.758/-0.024/0.00076 | | A/0.232/0.008/0.00047 | | A/0.208/0.002/0.4497 |
| rs76550486 | 5 | [HCN1](http://genome.ucsc.edu/cgi-bin/hgTracks?hgHubConnect.destUrl=..%2Fcgi-bin%2FhgTracks&clade=mammal&org=Human&db=hg19&position=HCN1) |  |  | NA | NA | A/0.998/0.31/4.4e-06 | | A/0.997/0.235/0.00151 | | NA | | NA |
| rs140065777 | 5 |  |  |  | NA | NA | T/0.999/0.312/4.28e-06 | | T/0.998/0.238/0.0015 | | NA | | NA |
| rs180976407 | 5 |  |  |  | NA | NA | G/0.999/0.319/3.77e-06 | | G/0.998/0.238/0.0017 | | NA | | NA |
| rs191414751 | 5 |  |  |  | NA | NA | C/0.999/0.32/3.98e-06 | | C/0.998/0.241/0.00159 | | NA | | NA |
| rs184606651 | 5 |  |  |  | NA | NA | G/0.999/0.32/4.06e-06 | | G/0.998/0.241/0.0016 | | NA | | NA |
| rs192419686 | 5 |  |  |  | NA | NA | T/0.999/0.321/3.96e-06 | | T/0.998/0.242/0.00161 | | NA | | NA |
| rs184759447 | 5 |  |  |  | NA | NA | T/0.999/0.324/3.79e-06 | | T/0.998/0.242/0.00172 | | NA | | NA |
| rs189178057 | 5 |  |  |  | NA | NA | C/0.999/0.331/3.39e-06 | | C/0.998/0.247/0.00165 | | NA | | NA |
| rs186947746 | 5 |  | [DEPDC1B (36285)](http://genome.ucsc.edu/cgi-bin/hgTracks?hgHubConnect.destUrl=..%2Fcgi-bin%2FhgTracks&clade=mammal&org=Human&db=hg19&position=DEPDC1B) | [ELOVL7 (15337)](http://genome.ucsc.edu/cgi-bin/hgTracks?hgHubConnect.destUrl=..%2Fcgi-bin%2FhgTracks&clade=mammal&org=Human&db=hg19&position=ELOVL7) | NA | NA | A/1/0.498/7.97e-07 | | A/0.999/0.327/0.00051 | | NA | | NA |
| rs185898158 | 5 |  | [DEPDC1B (46504)](http://genome.ucsc.edu/cgi-bin/hgTracks?hgHubConnect.destUrl=..%2Fcgi-bin%2FhgTracks&clade=mammal&org=Human&db=hg19&position=DEPDC1B) | [ELOVL7 (5118)](http://genome.ucsc.edu/cgi-bin/hgTracks?hgHubConnect.destUrl=..%2Fcgi-bin%2FhgTracks&clade=mammal&org=Human&db=hg19&position=ELOVL7) | NA | NA | A/1/0.5/7.91e-07 | | A/0.999/0.328/0.00051 | | NA | | NA |
| rs146002062 | 5 | [MAST4](http://genome.ucsc.edu/cgi-bin/hgTracks?hgHubConnect.destUrl=..%2Fcgi-bin%2FhgTracks&clade=mammal&org=Human&db=hg19&position=MAST4) |  | [BC080587 (175518)](http://genome.ucsc.edu/cgi-bin/hgTracks?hgHubConnect.destUrl=..%2Fcgi-bin%2FhgTracks&clade=mammal&org=Human&db=hg19&position=BC080587) | G/0.985/-0.019/0.1629 | G/0.985/-0.033/0.06728 | G/0.991/0.12/1.23e-06 | | G/0.986/0.09/0.00544 | | A/0.986/0.015/0.1405 | | A/0.985/-0.008/0.5371 |
| rs115559601 | 5 |  | [CD180 (26541)](http://genome.ucsc.edu/cgi-bin/hgTracks?hgHubConnect.destUrl=..%2Fcgi-bin%2FhgTracks&clade=mammal&org=Human&db=hg19&position=CD180) |  | NA | NA | T/0.993/0.102/1.68e-06 | | T/0.986/0.045/0.1137 | | NA | | NA |
| rs188526878 | 5 |  | [F2R (12787)](http://genome.ucsc.edu/cgi-bin/hgTracks?hgHubConnect.destUrl=..%2Fcgi-bin%2FhgTracks&clade=mammal&org=Human&db=hg19&position=F2R) | [F2RL1 (70450)](http://genome.ucsc.edu/cgi-bin/hgTracks?hgHubConnect.destUrl=..%2Fcgi-bin%2FhgTracks&clade=mammal&org=Human&db=hg19&position=F2RL1) | NA | NA | T/1/0.69/1.6e-06 | | T/0.999/0.346/0.00938 | | NA | | NA |
| rs185237819 | 5 |  | [LHFPL2 (106925)](http://genome.ucsc.edu/cgi-bin/hgTracks?hgHubConnect.destUrl=..%2Fcgi-bin%2FhgTracks&clade=mammal&org=Human&db=hg19&position=LHFPL2) | [ARSB (28126)](http://genome.ucsc.edu/cgi-bin/hgTracks?hgHubConnect.destUrl=..%2Fcgi-bin%2FhgTracks&clade=mammal&org=Human&db=hg19&position=ARSB) | G/0.998/0.017/0.7092 | G/0.998/0.019/0.7379 | G/1/0.631/1.75e-06 | | G/0.999/0.356/0.00358 | | A/0.998/0.029/0.3579 | | A/0.997/-0.019/0.5751 |
| rs145317423 | 5 | [EDIL3](http://genome.ucsc.edu/cgi-bin/hgTracks?hgHubConnect.destUrl=..%2Fcgi-bin%2FhgTracks&clade=mammal&org=Human&db=hg19&position=EDIL3) |  |  | NA | NA | C/0.984/0.066/2.23e-06 | | C/0.973/0.021/0.2757 | | NA | | NA |
| rs138833221 | 5 | [KIAA0825](http://genome.ucsc.edu/cgi-bin/hgTracks?hgHubConnect.destUrl=..%2Fcgi-bin%2FhgTracks&clade=mammal&org=Human&db=hg19&position=KIAA0825) | [FAM172A (248895)](http://genome.ucsc.edu/cgi-bin/hgTracks?hgHubConnect.destUrl=..%2Fcgi-bin%2FhgTracks&clade=mammal&org=Human&db=hg19&position=FAM172A) |  | A/0.994/-0.028/0.1477 | A/0.994/-0.018/0.4901 | A/0.998/0.217/1.01e-06 | | A/0.996/0.128/0.01272 | | A/0.006/-0.01/0.4593 | | A/0.006/0.007/0.656 |
| rs186147679 | 5 | [KIAA0825](http://genome.ucsc.edu/cgi-bin/hgTracks?hgHubConnect.destUrl=..%2Fcgi-bin%2FhgTracks&clade=mammal&org=Human&db=hg19&position=KIAA0825) |  | [ANKRD32 (245268)](http://genome.ucsc.edu/cgi-bin/hgTracks?hgHubConnect.destUrl=..%2Fcgi-bin%2FhgTracks&clade=mammal&org=Human&db=hg19&position=ANKRD32) | A/0.994/-0.03/0.1193 | A/0.994/-0.021/0.4351 | A/0.998/0.221/8.61e-07 | | A/0.996/0.132/0.0112 | | A/0.006/-0.01/0.4854 | | A/0.006/0.006/0.7311 |
| rs192514502 | 5 | [MCTP1](http://genome.ucsc.edu/cgi-bin/hgTracks?hgHubConnect.destUrl=..%2Fcgi-bin%2FhgTracks&clade=mammal&org=Human&db=hg19&position=MCTP1) | [ANKRD32 (243842)](http://genome.ucsc.edu/cgi-bin/hgTracks?hgHubConnect.destUrl=..%2Fcgi-bin%2FhgTracks&clade=mammal&org=Human&db=hg19&position=ANKRD32) |  | T/1/0.177/0.08566 | T/1/0.156/0.09006 | T/0.999/0.476/3.15e-06 | | T/0.998/0.264/0.00687 | | A/1/0.253/4.56e-05 | | A/1/0.105/0.1433 |
| rs138043549 | 5 |  | [ST8SIA4 (154374)](http://genome.ucsc.edu/cgi-bin/hgTracks?hgHubConnect.destUrl=..%2Fcgi-bin%2FhgTracks&clade=mammal&org=Human&db=hg19&position=ST8SIA4) |  | NA | NA | G/0.976/0.051/4.42e-06 | | G/0.967/0.058/8e-04 | | NA | | NA |
| rs73777486 | 5 |  | [ST8SIA4 (162447)](http://genome.ucsc.edu/cgi-bin/hgTracks?hgHubConnect.destUrl=..%2Fcgi-bin%2FhgTracks&clade=mammal&org=Human&db=hg19&position=ST8SIA4) |  | NA | NA | G/0.976/0.052/4.19e-06 | | G/0.967/0.059/8e-04 | | NA | | NA |
| rs76933135 | 5 |  | [ST8SIA4 (217957)](http://genome.ucsc.edu/cgi-bin/hgTracks?hgHubConnect.destUrl=..%2Fcgi-bin%2FhgTracks&clade=mammal&org=Human&db=hg19&position=ST8SIA4) |  | NA | NA | T/0.975/0.05/4.67e-06 | | T/0.966/0.054/0.00135 | | NA | | NA |
| rs73777909 | 5 |  |  |  | NA | NA | C/0.976/0.051/4.24e-06 | | C/0.967/0.057/0.001 | | NA | | NA |
| rs77905828 | 5 |  |  |  | NA | NA | A/0.978/0.054/2.07e-06 | | A/0.969/0.062/0.00036 | | NA | | NA |
| rs11956923 | 5 |  |  |  | C/0.972/-0.009/0.2574 | C/0.974/0.004/0.7013 | C/0.723/0.016/4.08e-06 | | C/0.693/0.018/0.00434 | | A/0.777/0.012/2e-04 | | A/0.975/-0.001/0.914 |
| rs73780158 | 5 |  |  |  | NA | NA | G/0.98/0.062/4.74e-07 | | G/0.97/0.067/3e-04 | | NA | | NA |
| rs184897315 | 5 |  |  |  | NA | NA | G/1/0.855/3.54e-07 | | G/0.999/0.475/0.00222 | | NA | | NA |
| rs144137862 | 5 |  |  | [HINT1 (53228)](http://genome.ucsc.edu/cgi-bin/hgTracks?hgHubConnect.destUrl=..%2Fcgi-bin%2FhgTracks&clade=mammal&org=Human&db=hg19&position=HINT1) | NA | NA | G/0.998/0.255/1.9e-06 | | G/0.996/0.165/0.01224 | | NA | | NA |
| rs143404520 | 5 | [SPOCK1](http://genome.ucsc.edu/cgi-bin/hgTracks?hgHubConnect.destUrl=..%2Fcgi-bin%2FhgTracks&clade=mammal&org=Human&db=hg19&position=SPOCK1) |  |  | NA | NA | T/0.999/0.258/4.28e-06 | | T/0.997/0.194/0.00163 | | NA | | NA |
| rs115854050 | 5 | [CD74](http://genome.ucsc.edu/cgi-bin/hgTracks?hgHubConnect.destUrl=..%2Fcgi-bin%2FhgTracks&clade=mammal&org=Human&db=hg19&position=CD74) | [TCOF1 (4448)](http://genome.ucsc.edu/cgi-bin/hgTracks?hgHubConnect.destUrl=..%2Fcgi-bin%2FhgTracks&clade=mammal&org=Human&db=hg19&position=TCOF1) | [RPS14 (39472)](http://genome.ucsc.edu/cgi-bin/hgTracks?hgHubConnect.destUrl=..%2Fcgi-bin%2FhgTracks&clade=mammal&org=Human&db=hg19&position=RPS14) | NA | NA | A/0.996/0.119/3.71e-06 | | A/0.992/0.056/0.1185 | | NA | | NA |
| rs187460213 | 5 |  | [DCTN4 (11178)](http://genome.ucsc.edu/cgi-bin/hgTracks?hgHubConnect.destUrl=..%2Fcgi-bin%2FhgTracks&clade=mammal&org=Human&db=hg19&position=DCTN4) | [C5orf62 (7672)](http://genome.ucsc.edu/cgi-bin/hgTracks?hgHubConnect.destUrl=..%2Fcgi-bin%2FhgTracks&clade=mammal&org=Human&db=hg19&position=C5orf62) | G/0.999/0.034/0.6224 | G/0.999/0.086/0.3415 | G/0.998/0.237/3.71e-06 | | G/0.996/0.127/0.01552 | | NA | | A/0.999/0.086/0.3414 |
| rs187769864 | 5 |  | [FAT2 (63808)](http://genome.ucsc.edu/cgi-bin/hgTracks?hgHubConnect.destUrl=..%2Fcgi-bin%2FhgTracks&clade=mammal&org=Human&db=hg19&position=FAT2) | [BC034636 (19526)](http://genome.ucsc.edu/cgi-bin/hgTracks?hgHubConnect.destUrl=..%2Fcgi-bin%2FhgTracks&clade=mammal&org=Human&db=hg19&position=BC034636) | NA | NA | A/1/0.613/2.29e-06 | | A/0.999/0.347/0.00447 | | NA | | NA |
| rs182791062 | 5 |  | [GLRA1 (14725)](http://genome.ucsc.edu/cgi-bin/hgTracks?hgHubConnect.destUrl=..%2Fcgi-bin%2FhgTracks&clade=mammal&org=Human&db=hg19&position=GLRA1) |  | NA | NA | G/1/0.506/4.09e-06 | | G/0.999/0.303/0.0057 | | NA | | NA |
| rs191199264 | 5 | [AK001582](http://genome.ucsc.edu/cgi-bin/hgTracks?hgHubConnect.destUrl=..%2Fcgi-bin%2FhgTracks&clade=mammal&org=Human&db=hg19&position=AK001582) | [GLRA1 (96303)](http://genome.ucsc.edu/cgi-bin/hgTracks?hgHubConnect.destUrl=..%2Fcgi-bin%2FhgTracks&clade=mammal&org=Human&db=hg19&position=GLRA1) |  | NA | NA | A/1/0.492/4.32e-06 | | A/0.999/0.296/0.00599 | | NA | | NA |
| rs73326661 | 5 |  |  |  | NA | NA | A/0.981/0.085/1.1e-08 | | A/0.972/0.076/0.00049 | | NA | | NA |
| rs142001454 | 5 |  |  |  | NA | NA | C/0.99/0.107/1.01e-07 | | C/0.983/0.068/0.01242 | | NA | | NA |
| rs73803919 | 5 | [ODZ2](http://genome.ucsc.edu/cgi-bin/hgTracks?hgHubConnect.destUrl=..%2Fcgi-bin%2FhgTracks&clade=mammal&org=Human&db=hg19&position=ODZ2) |  |  | NA | NA | A/0.994/0.171/7.29e-09 | | A/0.99/0.153/0.00012 | | NA | | NA |
| rs187885921 | 6 |  | [FAM50B (42764)](http://genome.ucsc.edu/cgi-bin/hgTracks?hgHubConnect.destUrl=..%2Fcgi-bin%2FhgTracks&clade=mammal&org=Human&db=hg19&position=FAM50B) | [PRPF4B (127253)](http://genome.ucsc.edu/cgi-bin/hgTracks?hgHubConnect.destUrl=..%2Fcgi-bin%2FhgTracks&clade=mammal&org=Human&db=hg19&position=PRPF4B) | NA | NA | C/0.999/0.366/3.6e-06 | | C/0.998/0.237/0.01087 | | NA | | NA |
| rs189431351 | 6 | [MRDS1](http://genome.ucsc.edu/cgi-bin/hgTracks?hgHubConnect.destUrl=..%2Fcgi-bin%2FhgTracks&clade=mammal&org=Human&db=hg19&position=MRDS1) | [OFCC1 (217591)](http://genome.ucsc.edu/cgi-bin/hgTracks?hgHubConnect.destUrl=..%2Fcgi-bin%2FhgTracks&clade=mammal&org=Human&db=hg19&position=OFCC1) | [TFAP2A (201492)](http://genome.ucsc.edu/cgi-bin/hgTracks?hgHubConnect.destUrl=..%2Fcgi-bin%2FhgTracks&clade=mammal&org=Human&db=hg19&position=TFAP2A) | NA | NA | T/0.999/0.359/1.17e-06 | | T/0.997/0.172/0.0197 | | NA | | NA |
| rs190801949 | 6 |  | [MRDS1 (1819)](http://genome.ucsc.edu/cgi-bin/hgTracks?hgHubConnect.destUrl=..%2Fcgi-bin%2FhgTracks&clade=mammal&org=Human&db=hg19&position=MRDS1) | [TFAP2A (183255)](http://genome.ucsc.edu/cgi-bin/hgTracks?hgHubConnect.destUrl=..%2Fcgi-bin%2FhgTracks&clade=mammal&org=Human&db=hg19&position=TFAP2A) | NA | NA | G/0.998/0.286/3.89e-06 | | G/0.996/0.155/0.01527 | | NA | | NA |
| rs185569610 | 6 |  | [C6orf105 (6080)](http://genome.ucsc.edu/cgi-bin/hgTracks?hgHubConnect.destUrl=..%2Fcgi-bin%2FhgTracks&clade=mammal&org=Human&db=hg19&position=C6orf105) |  | NA | NA | C/0.999/0.359/3.23e-06 | | C/0.998/0.236/0.00232 | | NA | | NA |
| rs186361153 | 6 | [FAM65B](http://genome.ucsc.edu/cgi-bin/hgTracks?hgHubConnect.destUrl=..%2Fcgi-bin%2FhgTracks&clade=mammal&org=Human&db=hg19&position=FAM65B) | [GMNN (206037)](http://genome.ucsc.edu/cgi-bin/hgTracks?hgHubConnect.destUrl=..%2Fcgi-bin%2FhgTracks&clade=mammal&org=Human&db=hg19&position=GMNN) | [CMAHP (88932)](http://genome.ucsc.edu/cgi-bin/hgTracks?hgHubConnect.destUrl=..%2Fcgi-bin%2FhgTracks&clade=mammal&org=Human&db=hg19&position=CMAHP) | G/0.999/-0.021/0.6752 | G/0.999/0.002/0.9783 | G/1/0.489/8.55e-07 | | G/0.999/0.268/0.00447 | | A/0.999/0.058/0.1185 | | A/0.999/-0.013/0.7755 |
| rs190027738 | 6 | [ZFAND3](http://genome.ucsc.edu/cgi-bin/hgTracks?hgHubConnect.destUrl=..%2Fcgi-bin%2FhgTracks&clade=mammal&org=Human&db=hg19&position=ZFAND3) |  | [BTBD9 (17484)](http://genome.ucsc.edu/cgi-bin/hgTracks?hgHubConnect.destUrl=..%2Fcgi-bin%2FhgTracks&clade=mammal&org=Human&db=hg19&position=BTBD9) | NA | NA | A/0.997/0.216/4.07e-06 | | A/0.996/0.225/0.00024 | | NA | | NA |
| rs77928651 | 6 | [BTBD9](http://genome.ucsc.edu/cgi-bin/hgTracks?hgHubConnect.destUrl=..%2Fcgi-bin%2FhgTracks&clade=mammal&org=Human&db=hg19&position=BTBD9) | [AX747659 (102365)](http://genome.ucsc.edu/cgi-bin/hgTracks?hgHubConnect.destUrl=..%2Fcgi-bin%2FhgTracks&clade=mammal&org=Human&db=hg19&position=AX747659) |  | NA | NA | A/0.999/0.254/1.35e-06 | | A/0.997/0.216/0.00039 | | NA | | NA |
| rs139632137 | 6 | [BTBD9](http://genome.ucsc.edu/cgi-bin/hgTracks?hgHubConnect.destUrl=..%2Fcgi-bin%2FhgTracks&clade=mammal&org=Human&db=hg19&position=BTBD9) | [AX747659 (104809)](http://genome.ucsc.edu/cgi-bin/hgTracks?hgHubConnect.destUrl=..%2Fcgi-bin%2FhgTracks&clade=mammal&org=Human&db=hg19&position=AX747659) |  | NA | NA | A/0.999/0.283/2.11e-06 | | A/0.997/0.246/0.00044 | | NA | | NA |
| rs185692311 | 6 |  |  | [UNC5CL (220244)](http://genome.ucsc.edu/cgi-bin/hgTracks?hgHubConnect.destUrl=..%2Fcgi-bin%2FhgTracks&clade=mammal&org=Human&db=hg19&position=UNC5CL) | NA | NA | C/0.999/0.535/5.34e-07 | | C/0.999/0.314/0.00339 | | NA | | NA |
| rs188657011 | 6 | [SUPT3H](http://genome.ucsc.edu/cgi-bin/hgTracks?hgHubConnect.destUrl=..%2Fcgi-bin%2FhgTracks&clade=mammal&org=Human&db=hg19&position=SUPT3H) |  |  | A/0.999/0.043/0.4566 | A/0.998/-0.001/0.9865 | A/0.999/0.367/1.56e-06 | | A/0.998/0.189/0.009 | | NA | | A/0.002/0.001/0.986 |
| rs181741409 | 6 | [SUPT3H](http://genome.ucsc.edu/cgi-bin/hgTracks?hgHubConnect.destUrl=..%2Fcgi-bin%2FhgTracks&clade=mammal&org=Human&db=hg19&position=SUPT3H) |  | [MIR586 (7624)](http://genome.ucsc.edu/cgi-bin/hgTracks?hgHubConnect.destUrl=..%2Fcgi-bin%2FhgTracks&clade=mammal&org=Human&db=hg19&position=MIR586) | NA | NA | A/0.999/0.462/2.8e-06 | | A/0.998/0.26/0.00735 | | NA | | NA |
| rs183433534 | 6 | [SLC25A27](http://genome.ucsc.edu/cgi-bin/hgTracks?hgHubConnect.destUrl=..%2Fcgi-bin%2FhgTracks&clade=mammal&org=Human&db=hg19&position=SLC25A27) | [CYP39A1 (3456)](http://genome.ucsc.edu/cgi-bin/hgTracks?hgHubConnect.destUrl=..%2Fcgi-bin%2FhgTracks&clade=mammal&org=Human&db=hg19&position=CYP39A1) | [TDRD6 (31632)](http://genome.ucsc.edu/cgi-bin/hgTracks?hgHubConnect.destUrl=..%2Fcgi-bin%2FhgTracks&clade=mammal&org=Human&db=hg19&position=TDRD6) | A/0.994/0.006/0.8189 | A/0.995/0/0.9972 | A/0.996/0.214/3.38e-06 | | A/0.994/0.144/0.01004 | | NA | | A/0.005/-0.01/0.6926 |
| rs149476743 | 6 | [PKHD1](http://genome.ucsc.edu/cgi-bin/hgTracks?hgHubConnect.destUrl=..%2Fcgi-bin%2FhgTracks&clade=mammal&org=Human&db=hg19&position=PKHD1) |  |  | NA | NA | T/0.995/0.143/1.37e-07 | | T/0.991/0.108/0.00262 | | NA | | NA |
| rs150576801 | 6 | [PKHD1](http://genome.ucsc.edu/cgi-bin/hgTracks?hgHubConnect.destUrl=..%2Fcgi-bin%2FhgTracks&clade=mammal&org=Human&db=hg19&position=PKHD1) |  |  | NA | NA | T/0.995/0.139/1.74e-07 | | T/0.992/0.129/0.00043 | | NA | | NA |
| rs146895361 | 6 | [PKHD1](http://genome.ucsc.edu/cgi-bin/hgTracks?hgHubConnect.destUrl=..%2Fcgi-bin%2FhgTracks&clade=mammal&org=Human&db=hg19&position=PKHD1) |  |  | NA | NA | A/0.995/0.15/6.05e-08 | | A/0.992/0.128/0.00057 | | NA | | NA |
| rs182332476 | 6 |  | [ELOVL5 (56578)](http://genome.ucsc.edu/cgi-bin/hgTracks?hgHubConnect.destUrl=..%2Fcgi-bin%2FhgTracks&clade=mammal&org=Human&db=hg19&position=ELOVL5) | [GCLC (91584)](http://genome.ucsc.edu/cgi-bin/hgTracks?hgHubConnect.destUrl=..%2Fcgi-bin%2FhgTracks&clade=mammal&org=Human&db=hg19&position=GCLC) | NA | NA | G/0.999/0.283/2.64e-06 | | G/0.998/0.301/9.88e-06 | | NA | | NA |
| rs190738582 | 6 |  |  |  | NA | NA | T/0.998/0.323/7.94e-08 | | T/0.996/0.21/0.00267 | | NA | | NA |
| rs181238686 | 6 |  |  |  | NA | NA | A/0.998/0.325/7.83e-08 | | A/0.996/0.212/0.00268 | | NA | | NA |
| rs144046596 | 6 |  | [RFX6 (79274)](http://genome.ucsc.edu/cgi-bin/hgTracks?hgHubConnect.destUrl=..%2Fcgi-bin%2FhgTracks&clade=mammal&org=Human&db=hg19&position=RFX6) |  | NA | NA | T/0.985/0.08/5.64e-07 | | T/0.975/0.042/0.06304 | | NA | | NA |
| rs56804048 | 6 |  |  |  | NA | NA | C/0.99/0.089/7.57e-07 | | C/0.981/0.036/0.134 | | NA | | NA |
| rs56392554 | 6 |  |  |  | NA | NA | G/0.989/0.08/3.22e-06 | | G/0.98/0.026/0.2578 | | NA | | NA |
| rs192087058 | 6 | [THEMIS](http://genome.ucsc.edu/cgi-bin/hgTracks?hgHubConnect.destUrl=..%2Fcgi-bin%2FhgTracks&clade=mammal&org=Human&db=hg19&position=THEMIS) |  | [PTPRK (78117)](http://genome.ucsc.edu/cgi-bin/hgTracks?hgHubConnect.destUrl=..%2Fcgi-bin%2FhgTracks&clade=mammal&org=Human&db=hg19&position=PTPRK) | A/0.99/0.006/0.6917 | A/0.989/-0.006/0.7559 | A/0.998/0.211/1.52e-06 | | A/0.996/0.172/0.00121 | | A/0.012/-0.021/0.04547 | | A/0.013/-0.005/0.6558 |
| rs151181520 | 6 | [THEMIS](http://genome.ucsc.edu/cgi-bin/hgTracks?hgHubConnect.destUrl=..%2Fcgi-bin%2FhgTracks&clade=mammal&org=Human&db=hg19&position=THEMIS) |  | [PTPRK (65756)](http://genome.ucsc.edu/cgi-bin/hgTracks?hgHubConnect.destUrl=..%2Fcgi-bin%2FhgTracks&clade=mammal&org=Human&db=hg19&position=PTPRK) | T/0.986/0/0.9749 | T/0.985/-0.011/0.5479 | T/0.998/0.199/4.67e-06 | | T/0.996/0.168/0.00154 | | T/0.016/-0.016/0.109 | | T/0.018/-0.003/0.8088 |
| rs145481885 | 6 |  | [TMEM200A (91261)](http://genome.ucsc.edu/cgi-bin/hgTracks?hgHubConnect.destUrl=..%2Fcgi-bin%2FhgTracks&clade=mammal&org=Human&db=hg19&position=TMEM200A) |  | NA | NA | G/0.991/0.081/2.73e-06 | | G/0.984/0.043/0.0703 | | NA | | NA |
| rs184091137 | 6 |  | [CITED2 (82045)](http://genome.ucsc.edu/cgi-bin/hgTracks?hgHubConnect.destUrl=..%2Fcgi-bin%2FhgTracks&clade=mammal&org=Human&db=hg19&position=CITED2) |  | T/1/0.126/0.09342 | T/0.999/-0.001/0.9922 | T/1/0.705/1.03e-06 | | T/0.999/0.456/0.00061 | | A/0.999/0.143/0.00252 | | A/0.999/0.033/0.4786 |
| rs150612539 | 6 | [PLAGL1](http://genome.ucsc.edu/cgi-bin/hgTracks?hgHubConnect.destUrl=..%2Fcgi-bin%2FhgTracks&clade=mammal&org=Human&db=hg19&position=PLAGL1) | [C6orf94 (14440)](http://genome.ucsc.edu/cgi-bin/hgTracks?hgHubConnect.destUrl=..%2Fcgi-bin%2FhgTracks&clade=mammal&org=Human&db=hg19&position=C6orf94) | [HYMAI (50110)](http://genome.ucsc.edu/cgi-bin/hgTracks?hgHubConnect.destUrl=..%2Fcgi-bin%2FhgTracks&clade=mammal&org=Human&db=hg19&position=HYMAI) | C/0.994/-0.051/0.00662 | C/0.996/-0.007/0.8215 | C/1/0.441/1.53e-06 | | C/0.999/0.281/0.001 | | T/0.995/-0.034/0.04354 | | T/0.997/-0.039/0.09494 |
| rs150029151 | 6 | [PLAGL1](http://genome.ucsc.edu/cgi-bin/hgTracks?hgHubConnect.destUrl=..%2Fcgi-bin%2FhgTracks&clade=mammal&org=Human&db=hg19&position=PLAGL1) | [HYMAI (16326)](http://genome.ucsc.edu/cgi-bin/hgTracks?hgHubConnect.destUrl=..%2Fcgi-bin%2FhgTracks&clade=mammal&org=Human&db=hg19&position=HYMAI) | [SF3B5 (69824)](http://genome.ucsc.edu/cgi-bin/hgTracks?hgHubConnect.destUrl=..%2Fcgi-bin%2FhgTracks&clade=mammal&org=Human&db=hg19&position=SF3B5) | A/0.994/-0.049/0.00926 | A/0.996/-0.002/0.9587 | A/1/0.432/1.68e-06 | | A/0.999/0.275/0.00104 | | A/0.005/0.033/0.04642 | | A/0.003/0.038/0.1148 |
| rs192748541 | 6 | [PLAGL1](http://genome.ucsc.edu/cgi-bin/hgTracks?hgHubConnect.destUrl=..%2Fcgi-bin%2FhgTracks&clade=mammal&org=Human&db=hg19&position=PLAGL1) | [HYMAI (43826)](http://genome.ucsc.edu/cgi-bin/hgTracks?hgHubConnect.destUrl=..%2Fcgi-bin%2FhgTracks&clade=mammal&org=Human&db=hg19&position=HYMAI) | [SF3B5 (42324)](http://genome.ucsc.edu/cgi-bin/hgTracks?hgHubConnect.destUrl=..%2Fcgi-bin%2FhgTracks&clade=mammal&org=Human&db=hg19&position=SF3B5) | A/0.994/-0.049/0.00833 | A/0.996/-0.003/0.9334 | A/1/0.434/1.49e-06 | | A/0.999/0.275/0.00102 | | A/0.005/0.034/0.04373 | | A/0.003/0.038/0.1138 |
| rs140594067 | 6 | [PARK2](http://genome.ucsc.edu/cgi-bin/hgTracks?hgHubConnect.destUrl=..%2Fcgi-bin%2FhgTracks&clade=mammal&org=Human&db=hg19&position=PARK2) |  |  | NA | NA | A/0.998/0.219/3.29e-07 | | A/0.994/0.097/0.0254 | | NA | | NA |
| rs146977322 | 6 | [PARK2](http://genome.ucsc.edu/cgi-bin/hgTracks?hgHubConnect.destUrl=..%2Fcgi-bin%2FhgTracks&clade=mammal&org=Human&db=hg19&position=PARK2) |  |  | NA | NA | A/0.998/0.216/6.33e-07 | | A/0.995/0.093/0.0336 | | NA | | NA |
| rs17438732 | 6 | [PARK2](http://genome.ucsc.edu/cgi-bin/hgTracks?hgHubConnect.destUrl=..%2Fcgi-bin%2FhgTracks&clade=mammal&org=Human&db=hg19&position=PARK2) |  |  | C/0.545/0.002/0.5322 | C/0.544/-0.001/0.8657 | G/0.682/0.016/2.49e-06 | | G/0.644/0.014/0.02124 | | C/0.517/0.003/0.1081 | | C/0.461/-0.002/0.5415 |
| rs60338266 | 6 | [PARK2](http://genome.ucsc.edu/cgi-bin/hgTracks?hgHubConnect.destUrl=..%2Fcgi-bin%2FhgTracks&clade=mammal&org=Human&db=hg19&position=PARK2) |  |  | G/0.546/0.002/0.5762 | G/0.546/-0.001/0.8626 | A/0.695/0.016/4.93e-06 | | A/0.659/0.015/0.0121 | | A/0.478/-0.003/0.07728 | | A/0.542/0.001/0.5909 |
| rs56681491 | 6 | [PARK2](http://genome.ucsc.edu/cgi-bin/hgTracks?hgHubConnect.destUrl=..%2Fcgi-bin%2FhgTracks&clade=mammal&org=Human&db=hg19&position=PARK2) |  |  | A/0.546/0.002/0.5764 | A/0.546/-0.001/0.863 | G/0.695/0.016/4.92e-06 | | G/0.659/0.015/0.01217 | | A/0.522/0.003/0.07728 | | A/0.458/-0.001/0.6018 |
| rs13191310 | 6 | [PARK2](http://genome.ucsc.edu/cgi-bin/hgTracks?hgHubConnect.destUrl=..%2Fcgi-bin%2FhgTracks&clade=mammal&org=Human&db=hg19&position=PARK2) |  |  | C/0.454/-0.002/0.4598 | C/0.454/0/0.9951 | T/0.304/-0.016/4.16e-06 | | T/0.304/-0.015/0.01355 | | T/0.456/0.003/0.1171 | | T/0.501/-0.002/0.4726 |
| rs151274007 | 6 | [PACRG](http://genome.ucsc.edu/cgi-bin/hgTracks?hgHubConnect.destUrl=..%2Fcgi-bin%2FhgTracks&clade=mammal&org=Human&db=hg19&position=PACRG) | [PARK2 (29912)](http://genome.ucsc.edu/cgi-bin/hgTracks?hgHubConnect.destUrl=..%2Fcgi-bin%2FhgTracks&clade=mammal&org=Human&db=hg19&position=PARK2) |  | NA | NA | C/1/0.671/4.21e-06 | | C/0.999/0.432/0.00167 | | NA | | NA |
| rs143655133 | 6 | [PACRG](http://genome.ucsc.edu/cgi-bin/hgTracks?hgHubConnect.destUrl=..%2Fcgi-bin%2FhgTracks&clade=mammal&org=Human&db=hg19&position=PACRG) | [PARK2 (44555)](http://genome.ucsc.edu/cgi-bin/hgTracks?hgHubConnect.destUrl=..%2Fcgi-bin%2FhgTracks&clade=mammal&org=Human&db=hg19&position=PARK2) |  | NA | NA | A/1/0.667/4.09e-06 | | A/0.999/0.433/0.00166 | | NA | | NA |
| rs866743 | 6 |  |  | [AF086258 (146946)](http://genome.ucsc.edu/cgi-bin/hgTracks?hgHubConnect.destUrl=..%2Fcgi-bin%2FhgTracks&clade=mammal&org=Human&db=hg19&position=AF086258) | C/0.373/0.004/0.1606 | C/0.373/0.001/0.737 | T/0.383/-0.015/2.74e-06 | | T/0.383/-0.021/0.00045 | | T/0.556/0.007/5.29e-05 | | T/0.494/0.002/0.4768 |
| rs893239 | 7 | [LFNG](http://genome.ucsc.edu/cgi-bin/hgTracks?hgHubConnect.destUrl=..%2Fcgi-bin%2FhgTracks&clade=mammal&org=Human&db=hg19&position=LFNG) | [BC034268 (77783)](http://genome.ucsc.edu/cgi-bin/hgTracks?hgHubConnect.destUrl=..%2Fcgi-bin%2FhgTracks&clade=mammal&org=Human&db=hg19&position=BC034268) | [MIR4648 (1439)](http://genome.ucsc.edu/cgi-bin/hgTracks?hgHubConnect.destUrl=..%2Fcgi-bin%2FhgTracks&clade=mammal&org=Human&db=hg19&position=MIR4648) | C/0.737/-0.002/0.6832 | C/0.735/-0.002/0.7551 | C/0.523/-0.023/1.3e-06 | | C/0.546/-0.027/0.00331 | | A/0.678/-0.008/0.00151 | | A/0.743/-0.002/0.5538 |
| rs115605431 | 7 |  | [BC038729 (7361)](http://genome.ucsc.edu/cgi-bin/hgTracks?hgHubConnect.destUrl=..%2Fcgi-bin%2FhgTracks&clade=mammal&org=Human&db=hg19&position=BC038729) |  | NA | NA | T/0.999/0.417/2.02e-07 | | T/0.998/0.223/0.00423 | | NA | | NA |
| rs75890985 | 7 |  | [AX746880 (63260)](http://genome.ucsc.edu/cgi-bin/hgTracks?hgHubConnect.destUrl=..%2Fcgi-bin%2FhgTracks&clade=mammal&org=Human&db=hg19&position=AX746880) |  | C/0.853/-0.002/0.6178 | C/0.859/0.004/0.4602 | C/0.954/0.039/1.46e-06 | | C/0.941/0.022/0.09463 | | T/0.866/0.004/0.2089 | | T/0.856/0/0.8911 |
| rs111751834 | 7 |  | [AX746880 (64084)](http://genome.ucsc.edu/cgi-bin/hgTracks?hgHubConnect.destUrl=..%2Fcgi-bin%2FhgTracks&clade=mammal&org=Human&db=hg19&position=AX746880) |  | G/0.853/-0.002/0.6201 | G/0.859/0.004/0.4586 | G/0.954/0.039/1.55e-06 | | G/0.94/0.022/0.09512 | | A/0.866/0.004/0.2009 | | A/0.856/0/0.8911 |
| rs80328607 | 7 |  | [AX746880 (64271)](http://genome.ucsc.edu/cgi-bin/hgTracks?hgHubConnect.destUrl=..%2Fcgi-bin%2FhgTracks&clade=mammal&org=Human&db=hg19&position=AX746880) |  | T/0.853/-0.002/0.6207 | T/0.859/0.004/0.4581 | T/0.954/0.039/1.57e-06 | | T/0.94/0.022/0.09535 | | T/0.134/-0.004/0.2009 | | T/0.144/0/0.8911 |
| rs117814736 | 7 |  |  | [TMEM106B (70090)](http://genome.ucsc.edu/cgi-bin/hgTracks?hgHubConnect.destUrl=..%2Fcgi-bin%2FhgTracks&clade=mammal&org=Human&db=hg19&position=TMEM106B) | C/0.995/-0.02/0.4226 | C/0.995/-0.024/0.4465 | C/0.998/0.268/2.15e-06 | | C/0.997/0.161/0.00998 | | T/0.995/0.027/0.1669 | | T/0.995/-0.021/0.3913 |
| rs183820509 | 7 |  |  | [TMEM106B (70026)](http://genome.ucsc.edu/cgi-bin/hgTracks?hgHubConnect.destUrl=..%2Fcgi-bin%2FhgTracks&clade=mammal&org=Human&db=hg19&position=TMEM106B) | A/0.995/-0.02/0.4226 | A/0.995/-0.024/0.4465 | A/0.998/0.268/2.15e-06 | | A/0.997/0.161/0.00998 | | A/0.005/-0.027/0.1667 | | A/0.005/0.021/0.3917 |
| rs141958207 | 7 |  |  | [TMEM106B (69347)](http://genome.ucsc.edu/cgi-bin/hgTracks?hgHubConnect.destUrl=..%2Fcgi-bin%2FhgTracks&clade=mammal&org=Human&db=hg19&position=TMEM106B) | T/0.995/-0.02/0.4223 | T/0.995/-0.024/0.4465 | T/0.998/0.268/2.16e-06 | | T/0.997/0.161/0.01002 | | T/0.005/-0.027/0.1666 | | T/0.005/0.021/0.3902 |
| rs1541424 | 7 |  | [LOC441204 (18827)](http://genome.ucsc.edu/cgi-bin/hgTracks?hgHubConnect.destUrl=..%2Fcgi-bin%2FhgTracks&clade=mammal&org=Human&db=hg19&position=LOC441204) | [KIAA0087 (15318)](http://genome.ucsc.edu/cgi-bin/hgTracks?hgHubConnect.destUrl=..%2Fcgi-bin%2FhgTracks&clade=mammal&org=Human&db=hg19&position=KIAA0087) | G/0.26/0/0.9728 | G/0.26/-0.003/0.4152 | G/0.161/-0.02/4.87e-06 | | G/0.161/-0.014/0.06062 | | A/0.764/-0.006/0.00886 | | A/0.739/-0.001/0.6784 |
| rs878361 | 7 |  | [LOC441204 (19274)](http://genome.ucsc.edu/cgi-bin/hgTracks?hgHubConnect.destUrl=..%2Fcgi-bin%2FhgTracks&clade=mammal&org=Human&db=hg19&position=LOC441204) | [KIAA0087 (14871)](http://genome.ucsc.edu/cgi-bin/hgTracks?hgHubConnect.destUrl=..%2Fcgi-bin%2FhgTracks&clade=mammal&org=Human&db=hg19&position=KIAA0087) | C/0.744/0/0.9613 | C/0.74/0.004/0.3386 | C/0.84/0.02/3.26e-06 | | C/0.814/0.017/0.0201 | | A/0.766/0.005/0.01113 | | A/0.741/0.001/0.6639 |
| rs2391283 | 7 |  | [LOC441204 (31665)](http://genome.ucsc.edu/cgi-bin/hgTracks?hgHubConnect.destUrl=..%2Fcgi-bin%2FhgTracks&clade=mammal&org=Human&db=hg19&position=LOC441204) | [KIAA0087 (2480)](http://genome.ucsc.edu/cgi-bin/hgTracks?hgHubConnect.destUrl=..%2Fcgi-bin%2FhgTracks&clade=mammal&org=Human&db=hg19&position=KIAA0087) | A/0.255/0/0.959 | A/0.255/-0.003/0.4051 | A/0.156/-0.021/9.26e-07 | | A/0.156/-0.015/0.03783 | | A/0.371/0.006/0.00698 | | A/0.516/0.001/0.686 |
| rs12700745 | 7 |  | [LOC441204 (31941)](http://genome.ucsc.edu/cgi-bin/hgTracks?hgHubConnect.destUrl=..%2Fcgi-bin%2FhgTracks&clade=mammal&org=Human&db=hg19&position=LOC441204) | [KIAA0087 (2204)](http://genome.ucsc.edu/cgi-bin/hgTracks?hgHubConnect.destUrl=..%2Fcgi-bin%2FhgTracks&clade=mammal&org=Human&db=hg19&position=KIAA0087) | G/0.74/0/0.9818 | G/0.737/0.004/0.367 | G/0.805/0.019/1.76e-06 | | G/0.772/0.01/0.1491 | | C/0.757/0.006/0.0052 | | C/0.74/0.001/0.645 |
| rs730954 | 7 | [KIAA0087](http://genome.ucsc.edu/cgi-bin/hgTracks?hgHubConnect.destUrl=..%2Fcgi-bin%2FhgTracks&clade=mammal&org=Human&db=hg19&position=KIAA0087) | [LOC441204 (36072)](http://genome.ucsc.edu/cgi-bin/hgTracks?hgHubConnect.destUrl=..%2Fcgi-bin%2FhgTracks&clade=mammal&org=Human&db=hg19&position=LOC441204) | [C7orf71 (102823)](http://genome.ucsc.edu/cgi-bin/hgTracks?hgHubConnect.destUrl=..%2Fcgi-bin%2FhgTracks&clade=mammal&org=Human&db=hg19&position=C7orf71) | A/0.735/0/0.8738 | A/0.733/0.004/0.2682 | A/0.832/0.02/2.48e-06 | | A/0.803/0.012/0.1103 | | A/0.241/-0.005/0.01178 | | A/0.264/-0.002/0.5561 |
| rs2391285 | 7 |  | [KIAA0087 (3352)](http://genome.ucsc.edu/cgi-bin/hgTracks?hgHubConnect.destUrl=..%2Fcgi-bin%2FhgTracks&clade=mammal&org=Human&db=hg19&position=KIAA0087) | [C7orf71 (95693)](http://genome.ucsc.edu/cgi-bin/hgTracks?hgHubConnect.destUrl=..%2Fcgi-bin%2FhgTracks&clade=mammal&org=Human&db=hg19&position=C7orf71) | A/0.281/0.002/0.5363 | A/0.281/-0.002/0.6298 | A/0.162/-0.02/3.12e-06 | | A/0.162/-0.013/0.07056 | | A/0.378/0.004/0.07895 | | A/0.513/0/0.9843 |
| rs181328644 | 7 | [AAA1](http://genome.ucsc.edu/cgi-bin/hgTracks?hgHubConnect.destUrl=..%2Fcgi-bin%2FhgTracks&clade=mammal&org=Human&db=hg19&position=AAA1) |  | [DPY19L1 (61666)](http://genome.ucsc.edu/cgi-bin/hgTracks?hgHubConnect.destUrl=..%2Fcgi-bin%2FhgTracks&clade=mammal&org=Human&db=hg19&position=DPY19L1) | C/0.995/0.016/0.4337 | C/0.994/-0.004/0.861 | C/0.999/0.348/2.96e-06 | | C/0.998/0.129/0.06385 | | T/0.995/0.036/0.01744 | | T/0.994/0.016/0.3551 |
| rs150347869 | 7 |  |  |  | A/0.997/0.071/0.01524 | A/0.996/0.04/0.2353 | A/1/0.483/2.96e-06 | | A/0.999/0.258/0.00844 | | A/0.004/-0.068/0.00168 | | A/0.004/-0.013/0.5555 |
| rs144872818 | 7 | [VWC2](http://genome.ucsc.edu/cgi-bin/hgTracks?hgHubConnect.destUrl=..%2Fcgi-bin%2FhgTracks&clade=mammal&org=Human&db=hg19&position=VWC2) |  | [ZPBP (82828)](http://genome.ucsc.edu/cgi-bin/hgTracks?hgHubConnect.destUrl=..%2Fcgi-bin%2FhgTracks&clade=mammal&org=Human&db=hg19&position=ZPBP) | T/0.978/-0.026/0.05304 | T/0.98/0.005/0.7817 | T/0.996/0.186/2.72e-06 | | T/0.993/0.102/0.03437 | | T/0.02/-0.002/0.8542 | | T/0.02/-0.025/0.05586 |
| rs142422697 | 7 |  |  | [ZPBP (11732)](http://genome.ucsc.edu/cgi-bin/hgTracks?hgHubConnect.destUrl=..%2Fcgi-bin%2FhgTracks&clade=mammal&org=Human&db=hg19&position=ZPBP) | C/0.978/-0.026/0.04703 | C/0.981/0.007/0.7323 | C/0.996/0.189/1.22e-06 | | C/0.992/0.097/0.03757 | | T/0.98/0.003/0.7723 | | T/0.981/0.026/0.04641 |
| rs114659987 | 7 | [COBL](http://genome.ucsc.edu/cgi-bin/hgTracks?hgHubConnect.destUrl=..%2Fcgi-bin%2FhgTracks&clade=mammal&org=Human&db=hg19&position=COBL) | [GRB10 (244699)](http://genome.ucsc.edu/cgi-bin/hgTracks?hgHubConnect.destUrl=..%2Fcgi-bin%2FhgTracks&clade=mammal&org=Human&db=hg19&position=GRB10) |  | NA | NA | A/0.999/0.452/4.72e-08 | | A/0.998/0.265/0.00139 | | NA | | NA |
| rs192739715 | 7 |  |  |  | C/0.994/0.004/0.8478 | C/0.995/0.013/0.6284 | C/0.998/0.181/3.44e-06 | | C/0.995/0.104/0.02057 | | A/0.995/0.019/0.1877 | | A/0.995/-0.006/0.765 |
| rs117375698 | 7 |  |  |  | T/0.997/0.012/0.7889 | T/0.997/-0.017/0.7647 | T/0.999/0.397/2.9e-07 | | T/0.998/0.302/0.0014 | | T/0.003/-0.051/0.07679 | | T/0.004/0.031/0.3708 |
| rs182033693 | 7 | [WBSCR17](http://genome.ucsc.edu/cgi-bin/hgTracks?hgHubConnect.destUrl=..%2Fcgi-bin%2FhgTracks&clade=mammal&org=Human&db=hg19&position=WBSCR17) |  | [MIR3914-1 (150230)](http://genome.ucsc.edu/cgi-bin/hgTracks?hgHubConnect.destUrl=..%2Fcgi-bin%2FhgTracks&clade=mammal&org=Human&db=hg19&position=MIR3914-1) | NA | NA | C/1/0.54/4.96e-06 | | C/0.999/0.401/0.00124 | | NA | | NA |
| rs183832949 | 7 | [MYL10](http://genome.ucsc.edu/cgi-bin/hgTracks?hgHubConnect.destUrl=..%2Fcgi-bin%2FhgTracks&clade=mammal&org=Human&db=hg19&position=MYL10) | [BC039094 (51040)](http://genome.ucsc.edu/cgi-bin/hgTracks?hgHubConnect.destUrl=..%2Fcgi-bin%2FhgTracks&clade=mammal&org=Human&db=hg19&position=BC039094) | [CUX1 (195857)](http://genome.ucsc.edu/cgi-bin/hgTracks?hgHubConnect.destUrl=..%2Fcgi-bin%2FhgTracks&clade=mammal&org=Human&db=hg19&position=CUX1) | A/0.997/0.004/0.93 | A/0.997/0.031/0.6388 | A/0.999/0.49/3.79e-06 | | A/0.999/0.299/0.00466 | | A/0.003/-0.057/0.08159 | | A/0.003/-0.033/0.4441 |
| rs188593748 | 7 | [PNPLA8](http://genome.ucsc.edu/cgi-bin/hgTracks?hgHubConnect.destUrl=..%2Fcgi-bin%2FhgTracks&clade=mammal&org=Human&db=hg19&position=PNPLA8) |  | [THAP5 (45448)](http://genome.ucsc.edu/cgi-bin/hgTracks?hgHubConnect.destUrl=..%2Fcgi-bin%2FhgTracks&clade=mammal&org=Human&db=hg19&position=THAP5) | NA | NA | A/0.999/0.451/8.05e-07 | | A/0.998/0.265/0.00263 | | NA | | NA |
| rs183464040 | 7 |  |  |  | NA | NA | G/0.999/0.622/2.03e-08 | | G/0.998/0.352/0.00076 | | NA | | NA |
| rs191729664 | 7 |  |  |  | NA | NA | T/0.999/0.671/2.51e-09 | | T/0.998/0.373/0.00043 | | NA | | NA |
| rs186074592 | 7 |  | [CHCHD3 (88443)](http://genome.ucsc.edu/cgi-bin/hgTracks?hgHubConnect.destUrl=..%2Fcgi-bin%2FhgTracks&clade=mammal&org=Human&db=hg19&position=CHCHD3) | [EXOC4 (82551)](http://genome.ucsc.edu/cgi-bin/hgTracks?hgHubConnect.destUrl=..%2Fcgi-bin%2FhgTracks&clade=mammal&org=Human&db=hg19&position=EXOC4) | NA | NA | T/1/0.784/3.94e-06 | | T/0.999/0.414/0.00926 | | NA | | NA |
| rs185746565 | 7 | [NUP205](http://genome.ucsc.edu/cgi-bin/hgTracks?hgHubConnect.destUrl=..%2Fcgi-bin%2FhgTracks&clade=mammal&org=Human&db=hg19&position=NUP205) | [CNOT4 (65174)](http://genome.ucsc.edu/cgi-bin/hgTracks?hgHubConnect.destUrl=..%2Fcgi-bin%2FhgTracks&clade=mammal&org=Human&db=hg19&position=CNOT4) | [C7orf73 (87171)](http://genome.ucsc.edu/cgi-bin/hgTracks?hgHubConnect.destUrl=..%2Fcgi-bin%2FhgTracks&clade=mammal&org=Human&db=hg19&position=C7orf73) | NA | NA | C/1/0.554/4.56e-06 | | C/0.999/0.302/0.00884 | | NA | | C/0.001/-0.019/0.828 |
| rs185205268 | 7 | [ARHGEF5](http://genome.ucsc.edu/cgi-bin/hgTracks?hgHubConnect.destUrl=..%2Fcgi-bin%2FhgTracks&clade=mammal&org=Human&db=hg19&position=ARHGEF5) | [CBRC7TM_40 (22885)](http://genome.ucsc.edu/cgi-bin/hgTracks?hgHubConnect.destUrl=..%2Fcgi-bin%2FhgTracks&clade=mammal&org=Human&db=hg19&position=CBRC7TM_40) | [NOBOX (19215)](http://genome.ucsc.edu/cgi-bin/hgTracks?hgHubConnect.destUrl=..%2Fcgi-bin%2FhgTracks&clade=mammal&org=Human&db=hg19&position=NOBOX) | NA | NA | G/0.998/0.236/4e-06 | | G/0.996/0.084/0.09928 | | NA | | NA |
| rs114174633 | 7 | [GALNTL5](http://genome.ucsc.edu/cgi-bin/hgTracks?hgHubConnect.destUrl=..%2Fcgi-bin%2FhgTracks&clade=mammal&org=Human&db=hg19&position=GALNTL5) | [LOC100505483 (129881)](http://genome.ucsc.edu/cgi-bin/hgTracks?hgHubConnect.destUrl=..%2Fcgi-bin%2FhgTracks&clade=mammal&org=Human&db=hg19&position=LOC100505483) | [GALNT11 (16588)](http://genome.ucsc.edu/cgi-bin/hgTracks?hgHubConnect.destUrl=..%2Fcgi-bin%2FhgTracks&clade=mammal&org=Human&db=hg19&position=GALNT11) | NA | NA | A/1/0.592/6.18e-08 | | A/0.999/0.375/0.00021 | | NA | | NA |
| rs141892389 | 7 | [GALNTL5](http://genome.ucsc.edu/cgi-bin/hgTracks?hgHubConnect.destUrl=..%2Fcgi-bin%2FhgTracks&clade=mammal&org=Human&db=hg19&position=GALNTL5) | [LOC100505483 (140403)](http://genome.ucsc.edu/cgi-bin/hgTracks?hgHubConnect.destUrl=..%2Fcgi-bin%2FhgTracks&clade=mammal&org=Human&db=hg19&position=LOC100505483) | [GALNT11 (6066)](http://genome.ucsc.edu/cgi-bin/hgTracks?hgHubConnect.destUrl=..%2Fcgi-bin%2FhgTracks&clade=mammal&org=Human&db=hg19&position=GALNT11) | NA | NA | A/0.999/0.495/1.05e-06 | | A/0.999/0.369/0.00024 | | NA | | NA |
| rs188061594 | 7 | [GALNT11](http://genome.ucsc.edu/cgi-bin/hgTracks?hgHubConnect.destUrl=..%2Fcgi-bin%2FhgTracks&clade=mammal&org=Human&db=hg19&position=GALNT11) | [GALNTL5 (12198)](http://genome.ucsc.edu/cgi-bin/hgTracks?hgHubConnect.destUrl=..%2Fcgi-bin%2FhgTracks&clade=mammal&org=Human&db=hg19&position=GALNTL5) | [MLL3 (102792)](http://genome.ucsc.edu/cgi-bin/hgTracks?hgHubConnect.destUrl=..%2Fcgi-bin%2FhgTracks&clade=mammal&org=Human&db=hg19&position=MLL3) | NA | NA | T/0.999/0.418/1.87e-06 | | T/0.998/0.352/0.00024 | | NA | | NA |
| rs147962389 | 7 | [GALNT11](http://genome.ucsc.edu/cgi-bin/hgTracks?hgHubConnect.destUrl=..%2Fcgi-bin%2FhgTracks&clade=mammal&org=Human&db=hg19&position=GALNT11) | [GALNTL5 (13872)](http://genome.ucsc.edu/cgi-bin/hgTracks?hgHubConnect.destUrl=..%2Fcgi-bin%2FhgTracks&clade=mammal&org=Human&db=hg19&position=GALNTL5) | [MLL3 (101118)](http://genome.ucsc.edu/cgi-bin/hgTracks?hgHubConnect.destUrl=..%2Fcgi-bin%2FhgTracks&clade=mammal&org=Human&db=hg19&position=MLL3) | NA | NA | A/0.999/0.413/2.2e-06 | | A/0.998/0.35/0.00025 | | NA | | NA |
| rs189909051 | 7 | [GALNT11](http://genome.ucsc.edu/cgi-bin/hgTracks?hgHubConnect.destUrl=..%2Fcgi-bin%2FhgTracks&clade=mammal&org=Human&db=hg19&position=GALNT11) | [GALNTL5 (14784)](http://genome.ucsc.edu/cgi-bin/hgTracks?hgHubConnect.destUrl=..%2Fcgi-bin%2FhgTracks&clade=mammal&org=Human&db=hg19&position=GALNTL5) | [MLL3 (100206)](http://genome.ucsc.edu/cgi-bin/hgTracks?hgHubConnect.destUrl=..%2Fcgi-bin%2FhgTracks&clade=mammal&org=Human&db=hg19&position=MLL3) | NA | NA | T/0.999/0.41/2.34e-06 | | T/0.998/0.35/0.00025 | | NA | | NA |
| rs149774937 | 7 | [GALNT11](http://genome.ucsc.edu/cgi-bin/hgTracks?hgHubConnect.destUrl=..%2Fcgi-bin%2FhgTracks&clade=mammal&org=Human&db=hg19&position=GALNT11) | [GALNTL5 (20001)](http://genome.ucsc.edu/cgi-bin/hgTracks?hgHubConnect.destUrl=..%2Fcgi-bin%2FhgTracks&clade=mammal&org=Human&db=hg19&position=GALNTL5) | [MLL3 (94989)](http://genome.ucsc.edu/cgi-bin/hgTracks?hgHubConnect.destUrl=..%2Fcgi-bin%2FhgTracks&clade=mammal&org=Human&db=hg19&position=MLL3) | NA | NA | C/0.999/0.401/2.97e-06 | | C/0.998/0.347/0.00026 | | NA | | NA |
| rs149619169 | 7 | [GALNT11](http://genome.ucsc.edu/cgi-bin/hgTracks?hgHubConnect.destUrl=..%2Fcgi-bin%2FhgTracks&clade=mammal&org=Human&db=hg19&position=GALNT11) | [GALNTL5 (22033)](http://genome.ucsc.edu/cgi-bin/hgTracks?hgHubConnect.destUrl=..%2Fcgi-bin%2FhgTracks&clade=mammal&org=Human&db=hg19&position=GALNTL5) | [MLL3 (92957)](http://genome.ucsc.edu/cgi-bin/hgTracks?hgHubConnect.destUrl=..%2Fcgi-bin%2FhgTracks&clade=mammal&org=Human&db=hg19&position=MLL3) | NA | NA | C/0.999/0.398/3.28e-06 | | C/0.998/0.346/0.00027 | | NA | | NA |
| rs148376973 | 7 | [GALNT11](http://genome.ucsc.edu/cgi-bin/hgTracks?hgHubConnect.destUrl=..%2Fcgi-bin%2FhgTracks&clade=mammal&org=Human&db=hg19&position=GALNT11) | [GALNTL5 (22251)](http://genome.ucsc.edu/cgi-bin/hgTracks?hgHubConnect.destUrl=..%2Fcgi-bin%2FhgTracks&clade=mammal&org=Human&db=hg19&position=GALNTL5) | [MLL3 (92739)](http://genome.ucsc.edu/cgi-bin/hgTracks?hgHubConnect.destUrl=..%2Fcgi-bin%2FhgTracks&clade=mammal&org=Human&db=hg19&position=MLL3) | NA | NA | T/0.999/0.397/3.29e-06 | | T/0.998/0.346/0.00027 | | NA | | NA |
| rs183747217 | 7 | [GALNT11](http://genome.ucsc.edu/cgi-bin/hgTracks?hgHubConnect.destUrl=..%2Fcgi-bin%2FhgTracks&clade=mammal&org=Human&db=hg19&position=GALNT11) | [GALNTL5 (23792)](http://genome.ucsc.edu/cgi-bin/hgTracks?hgHubConnect.destUrl=..%2Fcgi-bin%2FhgTracks&clade=mammal&org=Human&db=hg19&position=GALNTL5) | [MLL3 (91198)](http://genome.ucsc.edu/cgi-bin/hgTracks?hgHubConnect.destUrl=..%2Fcgi-bin%2FhgTracks&clade=mammal&org=Human&db=hg19&position=MLL3) | NA | NA | C/0.999/0.394/3.61e-06 | | C/0.998/0.346/0.00027 | | NA | | NA |
| rs114004118 | 7 | [GALNT11](http://genome.ucsc.edu/cgi-bin/hgTracks?hgHubConnect.destUrl=..%2Fcgi-bin%2FhgTracks&clade=mammal&org=Human&db=hg19&position=GALNT11) | [GALNTL5 (25083)](http://genome.ucsc.edu/cgi-bin/hgTracks?hgHubConnect.destUrl=..%2Fcgi-bin%2FhgTracks&clade=mammal&org=Human&db=hg19&position=GALNTL5) | [MLL3 (89907)](http://genome.ucsc.edu/cgi-bin/hgTracks?hgHubConnect.destUrl=..%2Fcgi-bin%2FhgTracks&clade=mammal&org=Human&db=hg19&position=MLL3) | NA | NA | A/0.999/0.392/3.93e-06 | | A/0.998/0.345/0.00028 | | NA | | NA |
| rs150256815 | 7 | [GALNT11](http://genome.ucsc.edu/cgi-bin/hgTracks?hgHubConnect.destUrl=..%2Fcgi-bin%2FhgTracks&clade=mammal&org=Human&db=hg19&position=GALNT11) | [GALNTL5 (26774)](http://genome.ucsc.edu/cgi-bin/hgTracks?hgHubConnect.destUrl=..%2Fcgi-bin%2FhgTracks&clade=mammal&org=Human&db=hg19&position=GALNTL5) | [MLL3 (88216)](http://genome.ucsc.edu/cgi-bin/hgTracks?hgHubConnect.destUrl=..%2Fcgi-bin%2FhgTracks&clade=mammal&org=Human&db=hg19&position=MLL3) | NA | NA | G/0.999/0.39/4.13e-06 | | G/0.998/0.345/0.00028 | | NA | | NA |
| rs139557951 | 7 | [GALNT11](http://genome.ucsc.edu/cgi-bin/hgTracks?hgHubConnect.destUrl=..%2Fcgi-bin%2FhgTracks&clade=mammal&org=Human&db=hg19&position=GALNT11) | [GALNTL5 (31455)](http://genome.ucsc.edu/cgi-bin/hgTracks?hgHubConnect.destUrl=..%2Fcgi-bin%2FhgTracks&clade=mammal&org=Human&db=hg19&position=GALNTL5) | [MLL3 (83535)](http://genome.ucsc.edu/cgi-bin/hgTracks?hgHubConnect.destUrl=..%2Fcgi-bin%2FhgTracks&clade=mammal&org=Human&db=hg19&position=MLL3) | NA | NA | C/0.999/0.384/4.82e-06 | | C/0.998/0.343/0.00029 | | NA | | NA |
| rs149069622 | 7 | [GALNT11](http://genome.ucsc.edu/cgi-bin/hgTracks?hgHubConnect.destUrl=..%2Fcgi-bin%2FhgTracks&clade=mammal&org=Human&db=hg19&position=GALNT11) | [GALNTL5 (32240)](http://genome.ucsc.edu/cgi-bin/hgTracks?hgHubConnect.destUrl=..%2Fcgi-bin%2FhgTracks&clade=mammal&org=Human&db=hg19&position=GALNTL5) | [MLL3 (82750)](http://genome.ucsc.edu/cgi-bin/hgTracks?hgHubConnect.destUrl=..%2Fcgi-bin%2FhgTracks&clade=mammal&org=Human&db=hg19&position=MLL3) | NA | NA | G/0.999/0.383/4.96e-06 | | G/0.998/0.343/0.00029 | | NA | | NA |
| rs182302106 | 8 | [DLGAP2](http://genome.ucsc.edu/cgi-bin/hgTracks?hgHubConnect.destUrl=..%2Fcgi-bin%2FhgTracks&clade=mammal&org=Human&db=hg19&position=DLGAP2) |  |  | NA | NA | G/0.999/0.45/1.94e-06 | | G/0.998/0.332/0.00202 | | NA | | NA |
| rs192316701 | 8 | [DLGAP2](http://genome.ucsc.edu/cgi-bin/hgTracks?hgHubConnect.destUrl=..%2Fcgi-bin%2FhgTracks&clade=mammal&org=Human&db=hg19&position=DLGAP2) |  |  | NA | NA | C/0.997/0.163/4.81e-06 | | C/0.995/0.116/0.0073 | | NA | | NA |
| rs191935895 | 8 |  |  |  | NA | NA | A/0.999/0.334/2.68e-06 | | A/0.997/0.064/0.375 | | NA | | NA |
| rs141088253 | 8 |  |  |  | NA | NA | G/0.999/0.384/1.33e-06 | | G/0.997/0.074/0.3592 | | NA | | NA |
| rs150432113 | 8 | [MCPH1](http://genome.ucsc.edu/cgi-bin/hgTracks?hgHubConnect.destUrl=..%2Fcgi-bin%2FhgTracks&clade=mammal&org=Human&db=hg19&position=MCPH1) | [ANGPT2 (43001)](http://genome.ucsc.edu/cgi-bin/hgTracks?hgHubConnect.destUrl=..%2Fcgi-bin%2FhgTracks&clade=mammal&org=Human&db=hg19&position=ANGPT2) | [AGPAT5 (102092)](http://genome.ucsc.edu/cgi-bin/hgTracks?hgHubConnect.destUrl=..%2Fcgi-bin%2FhgTracks&clade=mammal&org=Human&db=hg19&position=AGPAT5) | NA | NA | G/0.999/0.355/2.5e-06 | | G/0.998/0.161/0.02339 | | NA | | NA |
| rs186046079 | 8 | [MCPH1](http://genome.ucsc.edu/cgi-bin/hgTracks?hgHubConnect.destUrl=..%2Fcgi-bin%2FhgTracks&clade=mammal&org=Human&db=hg19&position=MCPH1) | [ANGPT2 (47930)](http://genome.ucsc.edu/cgi-bin/hgTracks?hgHubConnect.destUrl=..%2Fcgi-bin%2FhgTracks&clade=mammal&org=Human&db=hg19&position=ANGPT2) | [AGPAT5 (97163)](http://genome.ucsc.edu/cgi-bin/hgTracks?hgHubConnect.destUrl=..%2Fcgi-bin%2FhgTracks&clade=mammal&org=Human&db=hg19&position=AGPAT5) | NA | NA | T/0.999/0.332/3.96e-07 | | T/0.997/0.178/0.00487 | | NA | | NA |
| rs185206099 | 8 | [MCPH1](http://genome.ucsc.edu/cgi-bin/hgTracks?hgHubConnect.destUrl=..%2Fcgi-bin%2FhgTracks&clade=mammal&org=Human&db=hg19&position=MCPH1) | [ANGPT2 (56514)](http://genome.ucsc.edu/cgi-bin/hgTracks?hgHubConnect.destUrl=..%2Fcgi-bin%2FhgTracks&clade=mammal&org=Human&db=hg19&position=ANGPT2) | [AGPAT5 (88579)](http://genome.ucsc.edu/cgi-bin/hgTracks?hgHubConnect.destUrl=..%2Fcgi-bin%2FhgTracks&clade=mammal&org=Human&db=hg19&position=AGPAT5) | NA | NA | C/0.999/0.377/4.76e-09 | | C/0.997/0.182/0.00245 | | NA | | C/0.005/0.021/0.572 |
| rs138094162 | 8 | [MCPH1](http://genome.ucsc.edu/cgi-bin/hgTracks?hgHubConnect.destUrl=..%2Fcgi-bin%2FhgTracks&clade=mammal&org=Human&db=hg19&position=MCPH1) | [ANGPT2 (68784)](http://genome.ucsc.edu/cgi-bin/hgTracks?hgHubConnect.destUrl=..%2Fcgi-bin%2FhgTracks&clade=mammal&org=Human&db=hg19&position=ANGPT2) | [AGPAT5 (76309)](http://genome.ucsc.edu/cgi-bin/hgTracks?hgHubConnect.destUrl=..%2Fcgi-bin%2FhgTracks&clade=mammal&org=Human&db=hg19&position=AGPAT5) | NA | NA | G/0.999/0.287/6.29e-07 | | G/0.997/0.168/0.00412 | | NA | | NA |
| rs188858915 | 8 | [MCPH1](http://genome.ucsc.edu/cgi-bin/hgTracks?hgHubConnect.destUrl=..%2Fcgi-bin%2FhgTracks&clade=mammal&org=Human&db=hg19&position=MCPH1) | [ANGPT2 (74049)](http://genome.ucsc.edu/cgi-bin/hgTracks?hgHubConnect.destUrl=..%2Fcgi-bin%2FhgTracks&clade=mammal&org=Human&db=hg19&position=ANGPT2) | [AGPAT5 (71044)](http://genome.ucsc.edu/cgi-bin/hgTracks?hgHubConnect.destUrl=..%2Fcgi-bin%2FhgTracks&clade=mammal&org=Human&db=hg19&position=AGPAT5) | NA | NA | C/0.999/0.267/2.25e-06 | | C/0.997/0.169/0.00407 | | NA | | NA |
| rs141395649 | 8 | [MCPH1](http://genome.ucsc.edu/cgi-bin/hgTracks?hgHubConnect.destUrl=..%2Fcgi-bin%2FhgTracks&clade=mammal&org=Human&db=hg19&position=MCPH1) | [ANGPT2 (77906)](http://genome.ucsc.edu/cgi-bin/hgTracks?hgHubConnect.destUrl=..%2Fcgi-bin%2FhgTracks&clade=mammal&org=Human&db=hg19&position=ANGPT2) | [AGPAT5 (67187)](http://genome.ucsc.edu/cgi-bin/hgTracks?hgHubConnect.destUrl=..%2Fcgi-bin%2FhgTracks&clade=mammal&org=Human&db=hg19&position=AGPAT5) | NA | NA | C/0.999/0.27/3.15e-06 | | C/0.997/0.178/0.00347 | | NA | | NA |
| rs143335239 | 8 | [MCPH1](http://genome.ucsc.edu/cgi-bin/hgTracks?hgHubConnect.destUrl=..%2Fcgi-bin%2FhgTracks&clade=mammal&org=Human&db=hg19&position=MCPH1) | [ANGPT2 (80025)](http://genome.ucsc.edu/cgi-bin/hgTracks?hgHubConnect.destUrl=..%2Fcgi-bin%2FhgTracks&clade=mammal&org=Human&db=hg19&position=ANGPT2) | [AGPAT5 (65068)](http://genome.ucsc.edu/cgi-bin/hgTracks?hgHubConnect.destUrl=..%2Fcgi-bin%2FhgTracks&clade=mammal&org=Human&db=hg19&position=AGPAT5) | NA | NA | A/0.999/0.271/3.19e-06 | | A/0.997/0.179/0.00344 | | NA | | NA |
| rs150335055 | 8 |  | [LOC157273 (51674)](http://genome.ucsc.edu/cgi-bin/hgTracks?hgHubConnect.destUrl=..%2Fcgi-bin%2FhgTracks&clade=mammal&org=Human&db=hg19&position=LOC157273) | [TNKS (169180)](http://genome.ucsc.edu/cgi-bin/hgTracks?hgHubConnect.destUrl=..%2Fcgi-bin%2FhgTracks&clade=mammal&org=Human&db=hg19&position=TNKS) | T/0.993/0.019/0.2786 | T/0.992/0.028/0.1978 | T/0.999/0.214/2.25e-06 | | T/0.996/0.098/0.04433 | | A/0.994/0.049/0.00021 | | A/0.992/0.033/0.02042 |
| rs142487520 | 8 |  | [LOC157273 (81786)](http://genome.ucsc.edu/cgi-bin/hgTracks?hgHubConnect.destUrl=..%2Fcgi-bin%2FhgTracks&clade=mammal&org=Human&db=hg19&position=LOC157273) | [TNKS (139068)](http://genome.ucsc.edu/cgi-bin/hgTracks?hgHubConnect.destUrl=..%2Fcgi-bin%2FhgTracks&clade=mammal&org=Human&db=hg19&position=TNKS) | C/0.99/0.008/0.6055 | C/0.989/0.02/0.2784 | C/0.998/0.204/1.49e-06 | | C/0.996/0.089/0.05041 | | T/0.991/0.035/0.00256 | | T/0.989/0.025/0.0542 |
| rs144424757 | 8 |  | [KIAA1456 (42509)](http://genome.ucsc.edu/cgi-bin/hgTracks?hgHubConnect.destUrl=..%2Fcgi-bin%2FhgTracks&clade=mammal&org=Human&db=hg19&position=KIAA1456) | [DLC1 (11078)](http://genome.ucsc.edu/cgi-bin/hgTracks?hgHubConnect.destUrl=..%2Fcgi-bin%2FhgTracks&clade=mammal&org=Human&db=hg19&position=DLC1) | NA | NA | G/0.998/0.234/1.71e-06 | | G/0.996/0.173/0.00168 | | NA | | NA |
| rs144222594 | 8 |  | [KIAA1456 (44220)](http://genome.ucsc.edu/cgi-bin/hgTracks?hgHubConnect.destUrl=..%2Fcgi-bin%2FhgTracks&clade=mammal&org=Human&db=hg19&position=KIAA1456) | [DLC1 (9367)](http://genome.ucsc.edu/cgi-bin/hgTracks?hgHubConnect.destUrl=..%2Fcgi-bin%2FhgTracks&clade=mammal&org=Human&db=hg19&position=DLC1) | NA | NA | A/0.998/0.234/1.73e-06 | | A/0.996/0.173/0.00168 | | NA | | NA |
| rs147410223 | 8 |  | [FP15737 (74403)](http://genome.ucsc.edu/cgi-bin/hgTracks?hgHubConnect.destUrl=..%2Fcgi-bin%2FhgTracks&clade=mammal&org=Human&db=hg19&position=FP15737) | [NKX3-1 (28826)](http://genome.ucsc.edu/cgi-bin/hgTracks?hgHubConnect.destUrl=..%2Fcgi-bin%2FhgTracks&clade=mammal&org=Human&db=hg19&position=NKX3-1) | G/0.726/0/0.9514 | G/0.723/-0.001/0.7882 | G/0.783/0.021/7.22e-07 | | G/0.766/0.036/2.34e-06 | | A/0.743/0.004/0.05377 | | A/0.733/-0.003/0.2917 |
| rs147851892 | 8 |  | [ADAM28 (40743)](http://genome.ucsc.edu/cgi-bin/hgTracks?hgHubConnect.destUrl=..%2Fcgi-bin%2FhgTracks&clade=mammal&org=Human&db=hg19&position=ADAM28) | [ADAMDEC1 (7442)](http://genome.ucsc.edu/cgi-bin/hgTracks?hgHubConnect.destUrl=..%2Fcgi-bin%2FhgTracks&clade=mammal&org=Human&db=hg19&position=ADAMDEC1) | NA | NA | G/0.998/0.236/1.86e-07 | | G/0.996/0.167/0.00058 | | NA | | NA |
| rs113414285 | 8 |  | [ADAM7 (10780)](http://genome.ucsc.edu/cgi-bin/hgTracks?hgHubConnect.destUrl=..%2Fcgi-bin%2FhgTracks&clade=mammal&org=Human&db=hg19&position=ADAM7) |  | NA | NA | C/0.998/0.217/5.59e-07 | | C/0.996/0.128/0.00384 | | NA | | NA |
| rs111754512 | 8 |  | [ADAM7 (16165)](http://genome.ucsc.edu/cgi-bin/hgTracks?hgHubConnect.destUrl=..%2Fcgi-bin%2FhgTracks&clade=mammal&org=Human&db=hg19&position=ADAM7) |  | NA | NA | T/0.999/0.217/5.76e-07 | | T/0.996/0.128/0.00396 | | NA | | NA |
| rs187162159 | 8 | [DOCK5](http://genome.ucsc.edu/cgi-bin/hgTracks?hgHubConnect.destUrl=..%2Fcgi-bin%2FhgTracks&clade=mammal&org=Human&db=hg19&position=DOCK5) |  | [PPP2R2A (118468)](http://genome.ucsc.edu/cgi-bin/hgTracks?hgHubConnect.destUrl=..%2Fcgi-bin%2FhgTracks&clade=mammal&org=Human&db=hg19&position=PPP2R2A) | NA | NA | C/0.995/0.166/1.99e-07 | | C/0.992/0.092/0.02902 | | NA | | NA |
| rs182115318 | 8 | [DOCK5](http://genome.ucsc.edu/cgi-bin/hgTracks?hgHubConnect.destUrl=..%2Fcgi-bin%2FhgTracks&clade=mammal&org=Human&db=hg19&position=DOCK5) |  | [PPP2R2A (101351)](http://genome.ucsc.edu/cgi-bin/hgTracks?hgHubConnect.destUrl=..%2Fcgi-bin%2FhgTracks&clade=mammal&org=Human&db=hg19&position=PPP2R2A) | NA | NA | T/0.995/0.149/1.08e-06 | | T/0.992/0.087/0.03142 | | NA | | NA |
| rs182387400 | 8 | [DOCK5](http://genome.ucsc.edu/cgi-bin/hgTracks?hgHubConnect.destUrl=..%2Fcgi-bin%2FhgTracks&clade=mammal&org=Human&db=hg19&position=DOCK5) |  | [PPP2R2A (100366)](http://genome.ucsc.edu/cgi-bin/hgTracks?hgHubConnect.destUrl=..%2Fcgi-bin%2FhgTracks&clade=mammal&org=Human&db=hg19&position=PPP2R2A) | NA | NA | T/0.994/0.158/3.34e-08 | | T/0.99/0.105/0.00516 | | NA | | NA |
| rs147913210 | 8 | [DOCK5](http://genome.ucsc.edu/cgi-bin/hgTracks?hgHubConnect.destUrl=..%2Fcgi-bin%2FhgTracks&clade=mammal&org=Human&db=hg19&position=DOCK5) |  | [PPP2R2A (99841)](http://genome.ucsc.edu/cgi-bin/hgTracks?hgHubConnect.destUrl=..%2Fcgi-bin%2FhgTracks&clade=mammal&org=Human&db=hg19&position=PPP2R2A) | NA | NA | G/0.994/0.158/3.44e-08 | | G/0.99/0.105/0.00519 | | NA | | NA |
| rs150281976 | 8 | [DOCK5](http://genome.ucsc.edu/cgi-bin/hgTracks?hgHubConnect.destUrl=..%2Fcgi-bin%2FhgTracks&clade=mammal&org=Human&db=hg19&position=DOCK5) |  | [PPP2R2A (98615)](http://genome.ucsc.edu/cgi-bin/hgTracks?hgHubConnect.destUrl=..%2Fcgi-bin%2FhgTracks&clade=mammal&org=Human&db=hg19&position=PPP2R2A) | NA | NA | T/0.995/0.139/3.47e-06 | | T/0.991/0.086/0.03189 | | NA | | NA |
| rs190951784 | 8 | [DOCK5](http://genome.ucsc.edu/cgi-bin/hgTracks?hgHubConnect.destUrl=..%2Fcgi-bin%2FhgTracks&clade=mammal&org=Human&db=hg19&position=DOCK5) |  | [PPP2R2A (98551)](http://genome.ucsc.edu/cgi-bin/hgTracks?hgHubConnect.destUrl=..%2Fcgi-bin%2FhgTracks&clade=mammal&org=Human&db=hg19&position=PPP2R2A) | NA | NA | A/0.995/0.145/1.53e-06 | | A/0.991/0.085/0.03263 | | NA | | NA |
| rs183529902 | 8 |  | [AK092313 (226405)](http://genome.ucsc.edu/cgi-bin/hgTracks?hgHubConnect.destUrl=..%2Fcgi-bin%2FhgTracks&clade=mammal&org=Human&db=hg19&position=AK092313) |  | NA | NA | C/0.998/0.26/5e-06 | | C/0.996/0.145/0.02113 | | NA | | NA |
| rs72657954 | 8 | [LOC100132891](http://genome.ucsc.edu/cgi-bin/hgTracks?hgHubConnect.destUrl=..%2Fcgi-bin%2FhgTracks&clade=mammal&org=Human&db=hg19&position=LOC100132891) |  |  | A/0.994/-0.012/0.5905 | A/0.995/-0.011/0.7221 | A/0.999/0.288/2.15e-07 | | A/0.996/0.115/0.02839 | | A/0.006/-0.002/0.8811 | | A/0.005/0.013/0.5701 |
| rs72657970 | 8 | [LOC100132891](http://genome.ucsc.edu/cgi-bin/hgTracks?hgHubConnect.destUrl=..%2Fcgi-bin%2FhgTracks&clade=mammal&org=Human&db=hg19&position=LOC100132891) |  |  | A/0.994/-0.014/0.5331 | A/0.995/-0.014/0.6438 | A/0.999/0.288/1.65e-07 | | A/0.996/0.11/0.03174 | | A/0.006/-0.001/0.957 | | A/0.005/0.018/0.4074 |
| rs183191361 | 8 |  | [CRISPLD1 (136454)](http://genome.ucsc.edu/cgi-bin/hgTracks?hgHubConnect.destUrl=..%2Fcgi-bin%2FhgTracks&clade=mammal&org=Human&db=hg19&position=CRISPLD1) | [BC062758 (52413)](http://genome.ucsc.edu/cgi-bin/hgTracks?hgHubConnect.destUrl=..%2Fcgi-bin%2FhgTracks&clade=mammal&org=Human&db=hg19&position=BC062758) | NA | NA | T/0.999/0.444/6.21e-07 | | T/0.998/0.259/0.00299 | | NA | | NA |
| rs192994403 | 8 |  |  | [HNF4G (108849)](http://genome.ucsc.edu/cgi-bin/hgTracks?hgHubConnect.destUrl=..%2Fcgi-bin%2FhgTracks&clade=mammal&org=Human&db=hg19&position=HNF4G) | NA | NA | C/0.999/0.441/1.71e-07 | | C/0.998/0.251/0.00242 | | NA | | NA |
| rs114258913 | 8 |  |  |  | NA | NA | G/0.982/-0.074/2.98e-06 | | G/0.99/-0.072/0.09804 | | NA | | NA |
| rs56010636 | 8 |  |  |  | A/0.989/-0.041/0.05832 | A/0.99/0.013/0.7105 | A/0.998/0.287/5.07e-07 | | A/0.996/0.176/0.00837 | | NA | | A/0.01/-0.013/0.7106 |
| rs118061259 | 8 |  | [ABRA (45606)](http://genome.ucsc.edu/cgi-bin/hgTracks?hgHubConnect.destUrl=..%2Fcgi-bin%2FhgTracks&clade=mammal&org=Human&db=hg19&position=ABRA) |  | NA | NA | G/0.997/0.157/4.05e-06 | | G/0.994/0.098/0.01999 | | NA | | NA |
| rs187899339 | 8 |  | [KCNV1 (131029)](http://genome.ucsc.edu/cgi-bin/hgTracks?hgHubConnect.destUrl=..%2Fcgi-bin%2FhgTracks&clade=mammal&org=Human&db=hg19&position=KCNV1) |  | G/0.994/-0.026/0.2239 | G/0.994/-0.041/0.1601 | G/0.999/0.361/2.1e-06 | | G/0.998/0.212/0.00449 | | A/0.994/0.004/0.8077 | | A/0.994/-0.009/0.6781 |
| rs17189216 | 8 |  | [NOV (12254)](http://genome.ucsc.edu/cgi-bin/hgTracks?hgHubConnect.destUrl=..%2Fcgi-bin%2FhgTracks&clade=mammal&org=Human&db=hg19&position=NOV) | [ENPP2 (120384)](http://genome.ucsc.edu/cgi-bin/hgTracks?hgHubConnect.destUrl=..%2Fcgi-bin%2FhgTracks&clade=mammal&org=Human&db=hg19&position=ENPP2) | G/0.891/0/0.9681 | G/0.89/0.001/0.8745 | G/0.968/0.063/1.84e-07 | | G/0.955/0.044/0.01218 | | A/0.894/0.004/0.3351 | | A/0.888/-0.001/0.8695 |
| rs1585518 | 8 |  | [NOV (38560)](http://genome.ucsc.edu/cgi-bin/hgTracks?hgHubConnect.destUrl=..%2Fcgi-bin%2FhgTracks&clade=mammal&org=Human&db=hg19&position=NOV) | [ENPP2 (94078)](http://genome.ucsc.edu/cgi-bin/hgTracks?hgHubConnect.destUrl=..%2Fcgi-bin%2FhgTracks&clade=mammal&org=Human&db=hg19&position=ENPP2) | G/0.89/0.002/0.6816 | G/0.888/0.003/0.6157 | G/0.964/0.061/1.94e-07 | | G/0.952/0.047/0.00667 | | C/0.894/0.005/0.1384 | | C/0.887/0.001/0.8678 |
| rs62531470 | 9 | [KANK1](http://genome.ucsc.edu/cgi-bin/hgTracks?hgHubConnect.destUrl=..%2Fcgi-bin%2FhgTracks&clade=mammal&org=Human&db=hg19&position=KANK1) | [DOCK8 (227378)](http://genome.ucsc.edu/cgi-bin/hgTracks?hgHubConnect.destUrl=..%2Fcgi-bin%2FhgTracks&clade=mammal&org=Human&db=hg19&position=DOCK8) |  | T/0.995/0.02/0.445 | T/0.995/0.07/0.05315 | T/0.999/0.356/2.3e-06 | | T/0.997/0.197/0.01578 | | NA | | T/0.005/-0.04/0.1567 |
| rs143639284 | 9 |  | [JAK2 (12775)](http://genome.ucsc.edu/cgi-bin/hgTracks?hgHubConnect.destUrl=..%2Fcgi-bin%2FhgTracks&clade=mammal&org=Human&db=hg19&position=JAK2) | [INSL6 (22904)](http://genome.ucsc.edu/cgi-bin/hgTracks?hgHubConnect.destUrl=..%2Fcgi-bin%2FhgTracks&clade=mammal&org=Human&db=hg19&position=INSL6) | NA | NA | A/0.999/0.468/9.06e-07 | | A/0.998/0.249/0.00562 | | NA | | NA |
| rs144087410 | 9 | [INSL6](http://genome.ucsc.edu/cgi-bin/hgTracks?hgHubConnect.destUrl=..%2Fcgi-bin%2FhgTracks&clade=mammal&org=Human&db=hg19&position=INSL6) | [JAK2 (57398)](http://genome.ucsc.edu/cgi-bin/hgTracks?hgHubConnect.destUrl=..%2Fcgi-bin%2FhgTracks&clade=mammal&org=Human&db=hg19&position=JAK2) | [INSL4 (45837)](http://genome.ucsc.edu/cgi-bin/hgTracks?hgHubConnect.destUrl=..%2Fcgi-bin%2FhgTracks&clade=mammal&org=Human&db=hg19&position=INSL4) | T/0.992/0.006/0.7927 | T/0.991/0.004/0.8934 | T/0.999/0.397/2.39e-07 | | T/0.997/0.215/0.00397 | | NA | | A/0.992/0.003/0.8963 |
| rs185095244 | 9 | [PTPRD](http://genome.ucsc.edu/cgi-bin/hgTracks?hgHubConnect.destUrl=..%2Fcgi-bin%2FhgTracks&clade=mammal&org=Human&db=hg19&position=PTPRD) |  |  | A/0.997/-0.02/0.5014 | A/0.998/-0.034/0.4164 | A/1/0.389/1.9e-06 | | A/0.998/0.289/0.00063 | | A/0.002/-0.026/0.3087 | | A/0.002/0.012/0.7201 |
| rs142944815 | 9 | [PTPRD](http://genome.ucsc.edu/cgi-bin/hgTracks?hgHubConnect.destUrl=..%2Fcgi-bin%2FhgTracks&clade=mammal&org=Human&db=hg19&position=PTPRD) |  |  | G/0.997/-0.02/0.5037 | G/0.998/-0.035/0.4133 | G/1/0.39/1.93e-06 | | G/0.998/0.289/0.00064 | | T/0.998/0.026/0.3213 | | T/0.998/-0.015/0.658 |
| rs145068833 | 9 | [PTPRD](http://genome.ucsc.edu/cgi-bin/hgTracks?hgHubConnect.destUrl=..%2Fcgi-bin%2FhgTracks&clade=mammal&org=Human&db=hg19&position=PTPRD) |  |  | NA | NA | T/0.989/0.076/4.14e-06 | | T/0.98/0.025/0.2693 | | NA | | NA |
| rs79030472 | 9 |  |  |  | NA | NA | A/0.99/0.109/3.7e-07 | | A/0.982/0.039/0.1526 | | NA | | NA |
| rs117660864 | 9 |  |  |  | NA | NA | G/0.997/0.199/1.87e-06 | | G/0.994/0.079/0.08267 | | NA | | NA |
| rs183456655 | 9 |  |  |  | NA | NA | G/0.997/0.199/1.98e-06 | | G/0.994/0.079/0.08286 | | NA | | NA |
| rs191623547 | 9 |  |  |  | NA | NA | C/0.997/0.199/1.83e-06 | | C/0.994/0.084/0.06518 | | NA | | NA |
| rs183029213 | 9 | [JA374875](http://genome.ucsc.edu/cgi-bin/hgTracks?hgHubConnect.destUrl=..%2Fcgi-bin%2FhgTracks&clade=mammal&org=Human&db=hg19&position=JA374875) |  |  | NA | NA | C/0.997/0.209/7.56e-07 | | C/0.994/0.087/0.05713 | | NA | | NA |
| rs184680785 | 9 |  | [JA374875 (10490)](http://genome.ucsc.edu/cgi-bin/hgTracks?hgHubConnect.destUrl=..%2Fcgi-bin%2FhgTracks&clade=mammal&org=Human&db=hg19&position=JA374875) |  | NA | NA | C/0.997/0.17/2.81e-06 | | C/0.994/0.072/0.0992 | | NA | | NA |
| rs191531070 | 9 |  | [JA374875 (19700)](http://genome.ucsc.edu/cgi-bin/hgTracks?hgHubConnect.destUrl=..%2Fcgi-bin%2FhgTracks&clade=mammal&org=Human&db=hg19&position=JA374875) |  | NA | NA | T/0.998/0.225/6.1e-07 | | T/0.995/0.095/0.04859 | | NA | | NA |
| rs181672678 | 9 |  | [JA374875 (25419)](http://genome.ucsc.edu/cgi-bin/hgTracks?hgHubConnect.destUrl=..%2Fcgi-bin%2FhgTracks&clade=mammal&org=Human&db=hg19&position=JA374875) |  | NA | NA | G/0.998/0.225/6.2e-07 | | G/0.995/0.095/0.04858 | | NA | | NA |
| rs187930576 | 9 |  | [JA374875 (26611)](http://genome.ucsc.edu/cgi-bin/hgTracks?hgHubConnect.destUrl=..%2Fcgi-bin%2FhgTracks&clade=mammal&org=Human&db=hg19&position=JA374875) |  | NA | NA | C/0.998/0.226/6.13e-07 | | C/0.995/0.095/0.04848 | | NA | | NA |
| rs188120168 | 9 |  | [JA374875 (42364)](http://genome.ucsc.edu/cgi-bin/hgTracks?hgHubConnect.destUrl=..%2Fcgi-bin%2FhgTracks&clade=mammal&org=Human&db=hg19&position=JA374875) |  | NA | NA | C/0.998/0.23/9.63e-07 | | C/0.996/0.1/0.04308 | | NA | | NA |
| rs183397371 | 9 |  | [JA374875 (59247)](http://genome.ucsc.edu/cgi-bin/hgTracks?hgHubConnect.destUrl=..%2Fcgi-bin%2FhgTracks&clade=mammal&org=Human&db=hg19&position=JA374875) |  | NA | NA | C/0.998/0.231/1.41e-06 | | C/0.996/0.102/0.04218 | | NA | | NA |
| rs148340644 | 9 |  |  |  | T/0.992/-0.004/0.8663 | T/0.992/-0.038/0.1661 | T/1/0.543/3.85e-06 | | T/0.999/0.29/0.01205 | | NA | | T/0.008/0.028/0.2499 |
| rs78589086 | 9 |  |  |  | NA | NA | C/0.999/0.355/1.95e-06 | | C/0.998/0.236/0.00167 | | NA | | NA |
| rs189970609 | 9 |  |  |  | NA | NA | C/0.999/0.372/3.14e-07 | | C/0.998/0.262/0.00026 | | NA | | NA |
| rs139088314 | 9 |  |  |  | NA | NA | T/0.999/0.366/3.38e-07 | | T/0.998/0.259/0.00026 | | NA | | NA |
| rs138746541 | 9 |  |  |  | NA | NA | A/0.999/0.365/3.45e-07 | | A/0.998/0.258/0.00026 | | NA | | NA |
| rs139528199 | 9 |  |  |  | NA | NA | C/0.999/0.362/3.58e-07 | | C/0.998/0.257/0.00026 | | NA | | NA |
| rs149450639 | 9 |  |  |  | NA | NA | C/0.999/0.348/4.7e-07 | | C/0.998/0.254/0.00026 | | NA | | NA |
| rs80324951 | 9 |  |  |  | NA | NA | C/0.999/0.346/5.25e-07 | | C/0.998/0.252/0.00027 | | NA | | NA |
| rs147368481 | 9 |  |  |  | NA | NA | A/0.999/0.344/5.86e-07 | | A/0.998/0.251/0.00028 | | NA | | NA |
| rs76695314 | 9 |  |  |  | NA | NA | T/0.999/0.343/6.07e-07 | | T/0.998/0.251/0.00028 | | NA | | NA |
| rs149406381 | 9 |  |  |  | NA | NA | A/0.998/0.278/3.91e-06 | | A/0.997/0.167/0.00487 | | NA | | NA |
| rs17785414 | 9 |  |  |  | NA | NA | A/0.999/0.337/8e-07 | | A/0.997/0.249/3e-04 | | NA | | NA |
| rs17785420 | 9 |  |  |  | NA | NA | G/0.999/0.334/9.16e-07 | | G/0.997/0.247/0.00031 | | NA | | NA |
| rs139048350 | 9 |  |  |  | NA | NA | A/0.999/0.331/9.28e-07 | | A/0.997/0.246/0.00031 | | NA | | NA |
| rs138549889 | 9 |  |  |  | NA | NA | T/0.999/0.331/9.47e-07 | | T/0.997/0.246/0.00031 | | NA | | NA |
| rs145444918 | 9 |  |  |  | NA | NA | A/0.999/0.328/9.56e-07 | | A/0.997/0.245/0.00031 | | NA | | NA |
| rs184557780 | 9 |  |  |  | NA | NA | T/0.999/0.32/9.57e-07 | | T/0.997/0.241/3e-04 | | NA | | NA |
| rs75214736 | 9 |  |  |  | NA | NA | C/0.999/0.32/9.76e-07 | | C/0.997/0.24/0.00031 | | NA | | NA |
| rs151101851 | 9 |  |  |  | NA | NA | G/0.999/0.32/9.87e-07 | | G/0.997/0.24/0.00032 | | NA | | NA |
| rs151321188 | 9 |  |  |  | NA | NA | G/0.999/0.32/9.93e-07 | | G/0.997/0.24/0.00032 | | NA | | NA |
| rs143976741 | 9 |  |  |  | NA | NA | G/0.999/0.32/1.15e-06 | | G/0.997/0.24/0.00034 | | NA | | NA |
| rs74838144 | 9 |  |  |  | NA | NA | A/0.999/0.32/1.49e-06 | | A/0.997/0.239/0.00037 | | NA | | NA |
| rs184676284 | 9 |  |  |  | NA | NA | A/0.999/0.32/1.48e-06 | | A/0.997/0.239/0.00037 | | NA | | NA |
| rs141510725 | 9 |  |  |  | NA | NA | G/0.999/0.32/1.47e-06 | | G/0.997/0.239/0.00037 | | NA | | NA |
| rs138792676 | 9 |  |  |  | NA | NA | A/0.999/0.32/1.47e-06 | | A/0.997/0.239/0.00037 | | NA | | NA |
| rs145505122 | 9 |  |  |  | NA | NA | C/0.999/0.314/2.33e-06 | | C/0.997/0.239/0.00037 | | NA | | NA |
| rs148861184 | 9 |  |  |  | NA | NA | A/0.999/0.314/2.3e-06 | | A/0.997/0.239/0.00037 | | NA | | NA |
| rs77213524 | 9 |  |  |  | NA | NA | T/0.999/0.314/2.29e-06 | | T/0.997/0.239/0.00037 | | NA | | NA |
| rs191378958 | 9 |  |  |  | NA | NA | G/0.999/0.402/1.63e-06 | | G/0.998/0.278/0.00059 | | NA | | NA |
| rs143838614 | 9 |  |  |  | NA | NA | T/0.987/0.071/3.34e-06 | | T/0.982/0.059/0.01319 | | NA | | NA |
| rs115855507 | 9 |  |  |  | NA | NA | A/0.945/0.033/4.23e-06 | | A/0.923/0.013/0.2624 | | NA | | NA |
| rs116107739 | 9 |  |  |  | NA | NA | A/0.945/0.034/4.08e-06 | | A/0.923/0.012/0.2963 | | NA | | NA |
| rs114785263 | 9 |  |  |  | NA | NA | T/0.945/0.034/4.07e-06 | | T/0.923/0.012/0.2965 | | NA | | NA |
| rs59775286 | 9 |  |  |  | NA | NA | T/0.945/0.034/3.89e-06 | | T/0.923/0.012/0.2972 | | NA | | NA |
| rs58710288 | 9 |  |  |  | NA | NA | C/0.945/0.034/4.06e-06 | | C/0.923/0.012/0.2994 | | NA | | NA |
| rs115081025 | 9 |  |  |  | NA | NA | A/0.945/0.033/4.06e-06 | | A/0.923/0.011/0.3228 | | NA | | NA |
| rs184991195 | 9 |  |  |  | NA | NA | T/0.988/0.08/6.45e-07 | | T/0.981/0.052/0.03334 | | NA | | NA |
| rs149727148 | 9 |  |  |  | NA | NA | C/0.984/0.075/2.64e-06 | | C/0.979/0.052/0.03237 | | NA | | NA |
| rs188470542 | 9 |  | [TLE4 (62450)](http://genome.ucsc.edu/cgi-bin/hgTracks?hgHubConnect.destUrl=..%2Fcgi-bin%2FhgTracks&clade=mammal&org=Human&db=hg19&position=TLE4) | [DQ575560 (74791)](http://genome.ucsc.edu/cgi-bin/hgTracks?hgHubConnect.destUrl=..%2Fcgi-bin%2FhgTracks&clade=mammal&org=Human&db=hg19&position=DQ575560) | NA | NA | T/1/0.772/7.12e-07 | | T/0.999/0.447/0.00195 | | NA | | NA |
| rs184522131 | 9 | [DAPK1](http://genome.ucsc.edu/cgi-bin/hgTracks?hgHubConnect.destUrl=..%2Fcgi-bin%2FhgTracks&clade=mammal&org=Human&db=hg19&position=DAPK1) |  | [CTSL1 (163452)](http://genome.ucsc.edu/cgi-bin/hgTracks?hgHubConnect.destUrl=..%2Fcgi-bin%2FhgTracks&clade=mammal&org=Human&db=hg19&position=CTSL1) | NA | NA | G/1/0.513/3.78e-06 | | G/0.998/0.205/0.0498 | | NA | | A/0.997/-0.023/0.6961 |
| rs138411828 | 9 |  |  |  | NA | NA | C/1/0.684/4.85e-06 | | C/0.999/0.441/0.00161 | | NA | | NA |
| rs113752012 | 9 |  | [AK311445 (48677)](http://genome.ucsc.edu/cgi-bin/hgTracks?hgHubConnect.destUrl=..%2Fcgi-bin%2FhgTracks&clade=mammal&org=Human&db=hg19&position=AK311445) |  | NA | NA | A/0.999/0.448/3.19e-06 | | A/0.998/0.226/0.02331 | | NA | | NA |
| rs186915809 | 9 | [PALM2](http://genome.ucsc.edu/cgi-bin/hgTracks?hgHubConnect.destUrl=..%2Fcgi-bin%2FhgTracks&clade=mammal&org=Human&db=hg19&position=PALM2) | [PTPN3 (242267)](http://genome.ucsc.edu/cgi-bin/hgTracks?hgHubConnect.destUrl=..%2Fcgi-bin%2FhgTracks&clade=mammal&org=Human&db=hg19&position=PTPN3) | [AKAP2 (74578)](http://genome.ucsc.edu/cgi-bin/hgTracks?hgHubConnect.destUrl=..%2Fcgi-bin%2FhgTracks&clade=mammal&org=Human&db=hg19&position=AKAP2) | NA | NA | A/1/1.227/3.42e-06 | | A/1/0.717/0.00342 | | NA | | NA |
| rs185011186 | 9 | [HSDL2](http://genome.ucsc.edu/cgi-bin/hgTracks?hgHubConnect.destUrl=..%2Fcgi-bin%2FhgTracks&clade=mammal&org=Human&db=hg19&position=HSDL2) | [MIR3134 (49787)](http://genome.ucsc.edu/cgi-bin/hgTracks?hgHubConnect.destUrl=..%2Fcgi-bin%2FhgTracks&clade=mammal&org=Human&db=hg19&position=MIR3134) | [BC047074 (16494)](http://genome.ucsc.edu/cgi-bin/hgTracks?hgHubConnect.destUrl=..%2Fcgi-bin%2FhgTracks&clade=mammal&org=Human&db=hg19&position=BC047074) | NA | NA | A/0.999/0.302/1.85e-06 | | A/0.997/0.206/0.00145 | | NA | | NA |
| rs117000167 | 9 | [BC039180](http://genome.ucsc.edu/cgi-bin/hgTracks?hgHubConnect.destUrl=..%2Fcgi-bin%2FhgTracks&clade=mammal&org=Human&db=hg19&position=BC039180) | [DAB2IP (133889)](http://genome.ucsc.edu/cgi-bin/hgTracks?hgHubConnect.destUrl=..%2Fcgi-bin%2FhgTracks&clade=mammal&org=Human&db=hg19&position=DAB2IP) | [TTLL11 (57466)](http://genome.ucsc.edu/cgi-bin/hgTracks?hgHubConnect.destUrl=..%2Fcgi-bin%2FhgTracks&clade=mammal&org=Human&db=hg19&position=TTLL11) | G/0.996/-0.044/0.0732 | G/0.997/-0.05/0.2196 | G/1/0.568/1.51e-06 | | G/0.999/0.325/0.00323 | | C/0.996/-0.028/0.1651 | | C/0.997/-0.047/0.08419 |
| rs192256335 | 9 | [RALGPS1](http://genome.ucsc.edu/cgi-bin/hgTracks?hgHubConnect.destUrl=..%2Fcgi-bin%2FhgTracks&clade=mammal&org=Human&db=hg19&position=RALGPS1) | [ZBTB34 (77664)](http://genome.ucsc.edu/cgi-bin/hgTracks?hgHubConnect.destUrl=..%2Fcgi-bin%2FhgTracks&clade=mammal&org=Human&db=hg19&position=ZBTB34) |  | NA | NA | G/1/0.604/2.51e-06 | | G/0.999/0.404/0.0015 | | NA | | NA |
| rs187979858 | 9 | [SARDH](http://genome.ucsc.edu/cgi-bin/hgTracks?hgHubConnect.destUrl=..%2Fcgi-bin%2FhgTracks&clade=mammal&org=Human&db=hg19&position=SARDH) | [DBH (75632)](http://genome.ucsc.edu/cgi-bin/hgTracks?hgHubConnect.destUrl=..%2Fcgi-bin%2FhgTracks&clade=mammal&org=Human&db=hg19&position=DBH) | [AK123314 (26291)](http://genome.ucsc.edu/cgi-bin/hgTracks?hgHubConnect.destUrl=..%2Fcgi-bin%2FhgTracks&clade=mammal&org=Human&db=hg19&position=AK123314) | A/0.992/-0.003/0.8645 | A/0.991/-0.007/0.7461 | A/0.998/0.2/3.91e-06 | | A/0.996/0.129/0.01711 | | A/0.008/-0.018/0.1881 | | A/0.008/0.005/0.7573 |
| rs186821245 | 9 |  | [SARDH (958)](http://genome.ucsc.edu/cgi-bin/hgTracks?hgHubConnect.destUrl=..%2Fcgi-bin%2FhgTracks&clade=mammal&org=Human&db=hg19&position=SARDH) | [AK123314 (20354)](http://genome.ucsc.edu/cgi-bin/hgTracks?hgHubConnect.destUrl=..%2Fcgi-bin%2FhgTracks&clade=mammal&org=Human&db=hg19&position=AK123314) | C/0.991/-0.001/0.9659 | C/0.991/-0.008/0.7149 | C/0.997/0.204/2.56e-06 | | C/0.996/0.128/0.01813 | | C/0.008/-0.02/0.1376 | | C/0.009/0.004/0.7869 |
| rs142965926 | 9 |  | [SARDH (13051)](http://genome.ucsc.edu/cgi-bin/hgTracks?hgHubConnect.destUrl=..%2Fcgi-bin%2FhgTracks&clade=mammal&org=Human&db=hg19&position=SARDH) | [AK123314 (8261)](http://genome.ucsc.edu/cgi-bin/hgTracks?hgHubConnect.destUrl=..%2Fcgi-bin%2FhgTracks&clade=mammal&org=Human&db=hg19&position=AK123314) | G/0.99/0.024/0.1456 | G/0.989/0.007/0.7141 | G/0.996/0.189/1.18e-06 | | G/0.994/0.126/0.01071 | | C/0.991/0.036/0.00444 | | C/0.99/0.006/0.6805 |
| rs138071473 | 10 | [PRPF18](http://genome.ucsc.edu/cgi-bin/hgTracks?hgHubConnect.destUrl=..%2Fcgi-bin%2FhgTracks&clade=mammal&org=Human&db=hg19&position=PRPF18) | [BEND7 (171448)](http://genome.ucsc.edu/cgi-bin/hgTracks?hgHubConnect.destUrl=..%2Fcgi-bin%2FhgTracks&clade=mammal&org=Human&db=hg19&position=BEND7) | [AK055017 (7414)](http://genome.ucsc.edu/cgi-bin/hgTracks?hgHubConnect.destUrl=..%2Fcgi-bin%2FhgTracks&clade=mammal&org=Human&db=hg19&position=AK055017) | T/0.999/-0.088/0.2443 | T/1/0.083/0.5418 | T/1/0.698/4.83e-06 | | T/1/0.447/0.00149 | | NA | | T/0/-0.083/0.5417 |
| rs192665745 | 10 |  | [GAD2 (33890)](http://genome.ucsc.edu/cgi-bin/hgTracks?hgHubConnect.destUrl=..%2Fcgi-bin%2FhgTracks&clade=mammal&org=Human&db=hg19&position=GAD2) | [APBB1IP (99884)](http://genome.ucsc.edu/cgi-bin/hgTracks?hgHubConnect.destUrl=..%2Fcgi-bin%2FhgTracks&clade=mammal&org=Human&db=hg19&position=APBB1IP) | NA | NA | G/1/0.672/4.29e-06 | | G/0.999/0.392/0.00405 | | NA | | NA |
| rs183323229 | 10 | [C10orf68](http://genome.ucsc.edu/cgi-bin/hgTracks?hgHubConnect.destUrl=..%2Fcgi-bin%2FhgTracks&clade=mammal&org=Human&db=hg19&position=C10orf68) |  | [ITGB1 (21030)](http://genome.ucsc.edu/cgi-bin/hgTracks?hgHubConnect.destUrl=..%2Fcgi-bin%2FhgTracks&clade=mammal&org=Human&db=hg19&position=ITGB1) | NA | NA | G/0.996/0.177/4.48e-06 | | G/0.992/0.046/0.3198 | | NA | | NA |
| rs185671464 | 10 |  |  |  | NA | NA | C/0.999/0.326/2.37e-06 | | C/0.998/0.345/2.59e-05 | | NA | | NA |
| rs182344828 | 10 |  |  |  | NA | NA | G/1/0.645/2.91e-07 | | G/0.999/0.512/1e-04 | | NA | | NA |
| rs139479025 | 10 | [AIFM2](http://genome.ucsc.edu/cgi-bin/hgTracks?hgHubConnect.destUrl=..%2Fcgi-bin%2FhgTracks&clade=mammal&org=Human&db=hg19&position=AIFM2) | [H2AFY2 (8261)](http://genome.ucsc.edu/cgi-bin/hgTracks?hgHubConnect.destUrl=..%2Fcgi-bin%2FhgTracks&clade=mammal&org=Human&db=hg19&position=H2AFY2) | [TYSND1 (17431)](http://genome.ucsc.edu/cgi-bin/hgTracks?hgHubConnect.destUrl=..%2Fcgi-bin%2FhgTracks&clade=mammal&org=Human&db=hg19&position=TYSND1) | NA | NA | C/0.999/0.374/1.33e-06 | | C/0.998/0.266/9e-04 | | NA | | NA |
| rs144473309 | 10 |  | [ZNF503-AS1 (7725)](http://genome.ucsc.edu/cgi-bin/hgTracks?hgHubConnect.destUrl=..%2Fcgi-bin%2FhgTracks&clade=mammal&org=Human&db=hg19&position=ZNF503-AS1) | [ZNF503 (28441)](http://genome.ucsc.edu/cgi-bin/hgTracks?hgHubConnect.destUrl=..%2Fcgi-bin%2FhgTracks&clade=mammal&org=Human&db=hg19&position=ZNF503) | A/0.995/-0.004/0.8669 | A/0.994/-0.006/0.8376 | A/0.982/0.069/2.09e-06 | | A/0.973/0.049/0.01895 | | A/0.013/-0.042/0.00026 | | A/0.005/0/0.995 |
| rs1025561 | 10 |  | [LOC100132987 (76572)](http://genome.ucsc.edu/cgi-bin/hgTracks?hgHubConnect.destUrl=..%2Fcgi-bin%2FhgTracks&clade=mammal&org=Human&db=hg19&position=LOC100132987) |  | A/0.842/0.002/0.4846 | A/0.841/0.003/0.5435 | A/0.696/0.019/5.62e-07 | | A/0.666/0.016/0.01552 | | A/0.369/-0.008/0.00021 | | A/0.512/-0.002/0.5724 |
| rs2593149 | 10 |  | [LOC100132987 (79155)](http://genome.ucsc.edu/cgi-bin/hgTracks?hgHubConnect.destUrl=..%2Fcgi-bin%2FhgTracks&clade=mammal&org=Human&db=hg19&position=LOC100132987) |  | T/0.84/0.002/0.5556 | T/0.84/0.002/0.5903 | T/0.694/0.018/6.01e-07 | | T/0.665/0.016/0.01489 | | T/0.214/-0.008/2e-04 | | T/0.161/-0.002/0.4683 |
| rs1434432 | 10 |  | [LOC100132987 (85738)](http://genome.ucsc.edu/cgi-bin/hgTracks?hgHubConnect.destUrl=..%2Fcgi-bin%2FhgTracks&clade=mammal&org=Human&db=hg19&position=LOC100132987) |  | C/0.832/0/0.9458 | C/0.834/0.002/0.6713 | C/0.628/0.017/9.02e-07 | | C/0.599/0.016/0.01363 | | T/0.749/0.007/0.00071 | | T/0.831/0.002/0.4867 |
| rs182554582 | 10 |  | [GRID1 (68709)](http://genome.ucsc.edu/cgi-bin/hgTracks?hgHubConnect.destUrl=..%2Fcgi-bin%2FhgTracks&clade=mammal&org=Human&db=hg19&position=GRID1) | [WAPAL (53)](http://genome.ucsc.edu/cgi-bin/hgTracks?hgHubConnect.destUrl=..%2Fcgi-bin%2FhgTracks&clade=mammal&org=Human&db=hg19&position=WAPAL) | NA | NA | A/0.999/0.433/8.28e-07 | | A/0.998/0.206/0.01403 | | NA | | NA |
| rs184737911 | 10 | [LDB3](http://genome.ucsc.edu/cgi-bin/hgTracks?hgHubConnect.destUrl=..%2Fcgi-bin%2FhgTracks&clade=mammal&org=Human&db=hg19&position=LDB3) | [OPN4 (4687)](http://genome.ucsc.edu/cgi-bin/hgTracks?hgHubConnect.destUrl=..%2Fcgi-bin%2FhgTracks&clade=mammal&org=Human&db=hg19&position=OPN4) | [AX747977 (60856)](http://genome.ucsc.edu/cgi-bin/hgTracks?hgHubConnect.destUrl=..%2Fcgi-bin%2FhgTracks&clade=mammal&org=Human&db=hg19&position=AX747977) | NA | NA | G/0.999/0.249/3.41e-06 | | G/0.996/0.105/0.04781 | | NA | | NA |
| rs140682412 | 10 |  | [MINPP1 (19488)](http://genome.ucsc.edu/cgi-bin/hgTracks?hgHubConnect.destUrl=..%2Fcgi-bin%2FhgTracks&clade=mammal&org=Human&db=hg19&position=MINPP1) | [PAPSS2 (86769)](http://genome.ucsc.edu/cgi-bin/hgTracks?hgHubConnect.destUrl=..%2Fcgi-bin%2FhgTracks&clade=mammal&org=Human&db=hg19&position=PAPSS2) | NA | NA | C/1/0.436/1.93e-06 | | C/0.999/0.237/0.00522 | | NA | | NA |
| rs142973438 | 10 |  | [CEP55 (27150)](http://genome.ucsc.edu/cgi-bin/hgTracks?hgHubConnect.destUrl=..%2Fcgi-bin%2FhgTracks&clade=mammal&org=Human&db=hg19&position=CEP55) | [O3FAR1 (10422)](http://genome.ucsc.edu/cgi-bin/hgTracks?hgHubConnect.destUrl=..%2Fcgi-bin%2FhgTracks&clade=mammal&org=Human&db=hg19&position=O3FAR1) | NA | NA | A/0.977/0.049/3.47e-06 | | A/0.969/0.055/0.00104 | | NA | | NA |
| rs143988467 | 10 |  | [CEP55 (28545)](http://genome.ucsc.edu/cgi-bin/hgTracks?hgHubConnect.destUrl=..%2Fcgi-bin%2FhgTracks&clade=mammal&org=Human&db=hg19&position=CEP55) | [O3FAR1 (9027)](http://genome.ucsc.edu/cgi-bin/hgTracks?hgHubConnect.destUrl=..%2Fcgi-bin%2FhgTracks&clade=mammal&org=Human&db=hg19&position=O3FAR1) | NA | NA | G/0.977/0.049/3.45e-06 | | G/0.969/0.055/0.00105 | | NA | | NA |
| rs79105493 | 10 |  | [FRA10AC1 (12441)](http://genome.ucsc.edu/cgi-bin/hgTracks?hgHubConnect.destUrl=..%2Fcgi-bin%2FhgTracks&clade=mammal&org=Human&db=hg19&position=FRA10AC1) | [LGI1 (42211)](http://genome.ucsc.edu/cgi-bin/hgTracks?hgHubConnect.destUrl=..%2Fcgi-bin%2FhgTracks&clade=mammal&org=Human&db=hg19&position=LGI1) | NA | NA | C/0.998/0.254/3.45e-06 | | C/0.996/0.206/0.00263 | | NA | | NA |
| rs186856805 | 10 | [UBTD1](http://genome.ucsc.edu/cgi-bin/hgTracks?hgHubConnect.destUrl=..%2Fcgi-bin%2FhgTracks&clade=mammal&org=Human&db=hg19&position=UBTD1) | [MMS19 (13491)](http://genome.ucsc.edu/cgi-bin/hgTracks?hgHubConnect.destUrl=..%2Fcgi-bin%2FhgTracks&clade=mammal&org=Human&db=hg19&position=MMS19) | [ANKRD2 (60251)](http://genome.ucsc.edu/cgi-bin/hgTracks?hgHubConnect.destUrl=..%2Fcgi-bin%2FhgTracks&clade=mammal&org=Human&db=hg19&position=ANKRD2) | NA | NA | C/1/0.679/1.96e-06 | | C/0.999/0.469/0.00081 | | NA | | NA |
| rs190045766 | 10 |  | [C10orf46 (129128)](http://genome.ucsc.edu/cgi-bin/hgTracks?hgHubConnect.destUrl=..%2Fcgi-bin%2FhgTracks&clade=mammal&org=Human&db=hg19&position=C10orf46) | [NANOS1 (145341)](http://genome.ucsc.edu/cgi-bin/hgTracks?hgHubConnect.destUrl=..%2Fcgi-bin%2FhgTracks&clade=mammal&org=Human&db=hg19&position=NANOS1) | NA | NA | T/0.999/0.429/3.15e-06 | | T/0.998/0.246/0.00481 | | NA | | NA |
| rs141497497 | 10 |  |  |  | NA | NA | T/0.998/0.269/9.49e-08 | | T/0.995/0.177/0.00092 | | NA | | NA |
| rs112302542 | 10 |  |  |  | NA | NA | G/0.997/0.267/2.43e-08 | | G/0.994/0.174/0.00062 | | NA | | NA |
| rs187797607 | 10 |  |  |  | NA | NA | A/0.999/0.368/4.03e-06 | | A/0.998/0.208/0.01429 | | NA | | NA |
| rs112905267 | 10 |  |  |  | A/0.993/-0.001/0.9664 | A/0.993/0.012/0.7508 | A/0.998/0.3/1.34e-09 | | A/0.995/0.168/0.00103 | | NA | | A/0.007/-0.012/0.7516 |
| rs190671939 | 10 | [GPR123](http://genome.ucsc.edu/cgi-bin/hgTracks?hgHubConnect.destUrl=..%2Fcgi-bin%2FhgTracks&clade=mammal&org=Human&db=hg19&position=GPR123) | [AK125849 (120129)](http://genome.ucsc.edu/cgi-bin/hgTracks?hgHubConnect.destUrl=..%2Fcgi-bin%2FhgTracks&clade=mammal&org=Human&db=hg19&position=AK125849) | [KNDC1 (63983)](http://genome.ucsc.edu/cgi-bin/hgTracks?hgHubConnect.destUrl=..%2Fcgi-bin%2FhgTracks&clade=mammal&org=Human&db=hg19&position=KNDC1) | NA | NA | T/1/0.539/3.03e-07 | | T/0.999/0.279/0.00433 | | NA | | NA |
| rs185636671 | 10 |  | [C10orf125 (1265)](http://genome.ucsc.edu/cgi-bin/hgTracks?hgHubConnect.destUrl=..%2Fcgi-bin%2FhgTracks&clade=mammal&org=Human&db=hg19&position=C10orf125) | [ECHS1 (3192)](http://genome.ucsc.edu/cgi-bin/hgTracks?hgHubConnect.destUrl=..%2Fcgi-bin%2FhgTracks&clade=mammal&org=Human&db=hg19&position=ECHS1) | NA | NA | A/0.999/0.453/2.34e-06 | | A/0.998/0.246/0.00738 | | NA | | NA |
| rs188724679 | 10 | [MTG1](http://genome.ucsc.edu/cgi-bin/hgTracks?hgHubConnect.destUrl=..%2Fcgi-bin%2FhgTracks&clade=mammal&org=Human&db=hg19&position=MTG1) | [PAOX (19657)](http://genome.ucsc.edu/cgi-bin/hgTracks?hgHubConnect.destUrl=..%2Fcgi-bin%2FhgTracks&clade=mammal&org=Human&db=hg19&position=PAOX) | [SPRN (9314)](http://genome.ucsc.edu/cgi-bin/hgTracks?hgHubConnect.destUrl=..%2Fcgi-bin%2FhgTracks&clade=mammal&org=Human&db=hg19&position=SPRN) | NA | NA | C/0.999/0.43/3.91e-06 | | C/0.998/0.296/0.00244 | | NA | | NA |
| rs114639778 | 11 | [OTOG](http://genome.ucsc.edu/cgi-bin/hgTracks?hgHubConnect.destUrl=..%2Fcgi-bin%2FhgTracks&clade=mammal&org=Human&db=hg19&position=OTOG) | [USH1C (52768)](http://genome.ucsc.edu/cgi-bin/hgTracks?hgHubConnect.destUrl=..%2Fcgi-bin%2FhgTracks&clade=mammal&org=Human&db=hg19&position=USH1C) | [MYOD1 (122378)](http://genome.ucsc.edu/cgi-bin/hgTracks?hgHubConnect.destUrl=..%2Fcgi-bin%2FhgTracks&clade=mammal&org=Human&db=hg19&position=MYOD1) | NA | NA | G/0.998/0.257/2.17e-06 | | G/0.996/0.133/0.0126 | | NA | | NA |
| rs188867939 | 11 |  | [SLC17A6 (103168)](http://genome.ucsc.edu/cgi-bin/hgTracks?hgHubConnect.destUrl=..%2Fcgi-bin%2FhgTracks&clade=mammal&org=Human&db=hg19&position=SLC17A6) | [FANCF (139864)](http://genome.ucsc.edu/cgi-bin/hgTracks?hgHubConnect.destUrl=..%2Fcgi-bin%2FhgTracks&clade=mammal&org=Human&db=hg19&position=FANCF) | NA | NA | G/0.995/0.159/3.49e-06 | | G/0.992/0.074/0.07724 | | NA | | NA |
| rs146109676 | 11 | [PAMR1](http://genome.ucsc.edu/cgi-bin/hgTracks?hgHubConnect.destUrl=..%2Fcgi-bin%2FhgTracks&clade=mammal&org=Human&db=hg19&position=PAMR1) | [SLC1A2 (11766)](http://genome.ucsc.edu/cgi-bin/hgTracks?hgHubConnect.destUrl=..%2Fcgi-bin%2FhgTracks&clade=mammal&org=Human&db=hg19&position=SLC1A2) | [FJX1 (186358)](http://genome.ucsc.edu/cgi-bin/hgTracks?hgHubConnect.destUrl=..%2Fcgi-bin%2FhgTracks&clade=mammal&org=Human&db=hg19&position=FJX1) | NA | NA | C/1/0.425/3.59e-06 | | C/0.999/0.294/0.00121 | | NA | | NA |
| rs184852128 | 11 | [LRRC4C](http://genome.ucsc.edu/cgi-bin/hgTracks?hgHubConnect.destUrl=..%2Fcgi-bin%2FhgTracks&clade=mammal&org=Human&db=hg19&position=LRRC4C) |  |  | NA | NA | T/1/0.432/1.03e-06 | | T/0.999/0.235/0.00422 | | NA | | NA |
| rs144572046 | 11 |  | [LOC221122 (59553)](http://genome.ucsc.edu/cgi-bin/hgTracks?hgHubConnect.destUrl=..%2Fcgi-bin%2FhgTracks&clade=mammal&org=Human&db=hg19&position=LOC221122) | [PRDM11 (56432)](http://genome.ucsc.edu/cgi-bin/hgTracks?hgHubConnect.destUrl=..%2Fcgi-bin%2FhgTracks&clade=mammal&org=Human&db=hg19&position=PRDM11) | NA | NA | T/0.978/0.054/3.76e-06 | | T/0.97/0.078/2.54e-05 | | NA | | NA |
| rs137877371 | 11 |  | [MS4A1 (2795)](http://genome.ucsc.edu/cgi-bin/hgTracks?hgHubConnect.destUrl=..%2Fcgi-bin%2FhgTracks&clade=mammal&org=Human&db=hg19&position=MS4A1) | [MS4A12 (19230)](http://genome.ucsc.edu/cgi-bin/hgTracks?hgHubConnect.destUrl=..%2Fcgi-bin%2FhgTracks&clade=mammal&org=Human&db=hg19&position=MS4A12) | NA | NA | T/0.96/0.044/3.05e-06 | | T/0.948/0.05/0.00087 | | NA | | NA |
| rs77902874 | 11 |  | [MS4A1 (13120)](http://genome.ucsc.edu/cgi-bin/hgTracks?hgHubConnect.destUrl=..%2Fcgi-bin%2FhgTracks&clade=mammal&org=Human&db=hg19&position=MS4A1) | [MS4A12 (8905)](http://genome.ucsc.edu/cgi-bin/hgTracks?hgHubConnect.destUrl=..%2Fcgi-bin%2FhgTracks&clade=mammal&org=Human&db=hg19&position=MS4A12) | NA | NA | A/0.957/0.043/1.19e-06 | | A/0.943/0.04/0.00339 | | NA | | NA |
| rs149746592 | 11 |  | [AHNAK (11375)](http://genome.ucsc.edu/cgi-bin/hgTracks?hgHubConnect.destUrl=..%2Fcgi-bin%2FhgTracks&clade=mammal&org=Human&db=hg19&position=AHNAK) | [EEF1G (1365)](http://genome.ucsc.edu/cgi-bin/hgTracks?hgHubConnect.destUrl=..%2Fcgi-bin%2FhgTracks&clade=mammal&org=Human&db=hg19&position=EEF1G) | A/0.996/-0.016/0.6834 | A/0.996/-0.064/0.1811 | A/0.976/0.05/4.43e-06 | | A/0.967/0.048/0.00531 | | NA | | A/0.004/0.064/0.1815 |
| rs146799327 | 11 | [TUT1](http://genome.ucsc.edu/cgi-bin/hgTracks?hgHubConnect.destUrl=..%2Fcgi-bin%2FhgTracks&clade=mammal&org=Human&db=hg19&position=TUT1) | [EEF1G (4630)](http://genome.ucsc.edu/cgi-bin/hgTracks?hgHubConnect.destUrl=..%2Fcgi-bin%2FhgTracks&clade=mammal&org=Human&db=hg19&position=EEF1G) | [MTA2 (13643)](http://genome.ucsc.edu/cgi-bin/hgTracks?hgHubConnect.destUrl=..%2Fcgi-bin%2FhgTracks&clade=mammal&org=Human&db=hg19&position=MTA2) | NA | NA | T/0.996/0.155/4.86e-06 | | T/0.993/0.056/0.2063 | | NA | | NA |
| rs188954228 | 11 | [ATG2A](http://genome.ucsc.edu/cgi-bin/hgTracks?hgHubConnect.destUrl=..%2Fcgi-bin%2FhgTracks&clade=mammal&org=Human&db=hg19&position=ATG2A) | [AB429224 (22004)](http://genome.ucsc.edu/cgi-bin/hgTracks?hgHubConnect.destUrl=..%2Fcgi-bin%2FhgTracks&clade=mammal&org=Human&db=hg19&position=AB429224) | [PPP2R5B (9217)](http://genome.ucsc.edu/cgi-bin/hgTracks?hgHubConnect.destUrl=..%2Fcgi-bin%2FhgTracks&clade=mammal&org=Human&db=hg19&position=PPP2R5B) | NA | NA | C/0.998/0.263/3.1e-06 | | C/0.996/0.192/0.00816 | | NA | | NA |
| rs77988765 | 11 |  | [FADD (4077)](http://genome.ucsc.edu/cgi-bin/hgTracks?hgHubConnect.destUrl=..%2Fcgi-bin%2FhgTracks&clade=mammal&org=Human&db=hg19&position=FADD) | [PPFIA1 (59220)](http://genome.ucsc.edu/cgi-bin/hgTracks?hgHubConnect.destUrl=..%2Fcgi-bin%2FhgTracks&clade=mammal&org=Human&db=hg19&position=PPFIA1) | G/0.87/0.004/0.2909 | G/0.866/0.001/0.8677 | G/0.973/0.047/4.04e-06 | | G/0.965/0.05/0.00191 | | A/0.88/0.006/0.03885 | | A/0.87/0/0.9528 |
| rs150267141 | 11 |  | [FADD (34791)](http://genome.ucsc.edu/cgi-bin/hgTracks?hgHubConnect.destUrl=..%2Fcgi-bin%2FhgTracks&clade=mammal&org=Human&db=hg19&position=FADD) | [PPFIA1 (28506)](http://genome.ucsc.edu/cgi-bin/hgTracks?hgHubConnect.destUrl=..%2Fcgi-bin%2FhgTracks&clade=mammal&org=Human&db=hg19&position=PPFIA1) | T/0.956/0.016/0.09185 | T/0.953/0.008/0.5242 | T/0.992/0.134/3.46e-06 | | T/0.988/0.092/0.02336 | | T/0.041/-0.018/0.01557 | | T/0.045/0/0.9962 |
| rs118084537 | 11 |  | [FADD (37198)](http://genome.ucsc.edu/cgi-bin/hgTracks?hgHubConnect.destUrl=..%2Fcgi-bin%2FhgTracks&clade=mammal&org=Human&db=hg19&position=FADD) | [PPFIA1 (26099)](http://genome.ucsc.edu/cgi-bin/hgTracks?hgHubConnect.destUrl=..%2Fcgi-bin%2FhgTracks&clade=mammal&org=Human&db=hg19&position=PPFIA1) | T/0.94/0.013/0.1048 | T/0.938/0.004/0.7319 | T/0.989/0.124/5.56e-08 | | T/0.984/0.069/0.02842 | | T/0.055/-0.017/0.00698 | | T/0.06/0/0.9499 |
| rs180910263 | 11 |  | [CTTN (28355)](http://genome.ucsc.edu/cgi-bin/hgTracks?hgHubConnect.destUrl=..%2Fcgi-bin%2FhgTracks&clade=mammal&org=Human&db=hg19&position=CTTN) | [SHANK2 (2915)](http://genome.ucsc.edu/cgi-bin/hgTracks?hgHubConnect.destUrl=..%2Fcgi-bin%2FhgTracks&clade=mammal&org=Human&db=hg19&position=SHANK2) | G/0.997/0.023/0.5772 | G/0.997/0.057/0.2538 | G/1/0.625/1.05e-06 | | G/0.999/0.311/0.00954 | | NA | | C/0.997/0.057/0.2536 |
| rs143737902 | 11 | [AK128563](http://genome.ucsc.edu/cgi-bin/hgTracks?hgHubConnect.destUrl=..%2Fcgi-bin%2FhgTracks&clade=mammal&org=Human&db=hg19&position=AK128563) | [CTTN (37733)](http://genome.ucsc.edu/cgi-bin/hgTracks?hgHubConnect.destUrl=..%2Fcgi-bin%2FhgTracks&clade=mammal&org=Human&db=hg19&position=CTTN) | [SHANK2 (15847)](http://genome.ucsc.edu/cgi-bin/hgTracks?hgHubConnect.destUrl=..%2Fcgi-bin%2FhgTracks&clade=mammal&org=Human&db=hg19&position=SHANK2) | G/0.998/0.042/0.3594 | G/0.998/0.072/0.174 | G/1/0.617/1.61e-06 | | G/0.999/0.31/0.01 | | NA | | C/0.998/0.072/0.1733 |
| rs117767473 | 11 | [BC127192](http://genome.ucsc.edu/cgi-bin/hgTracks?hgHubConnect.destUrl=..%2Fcgi-bin%2FhgTracks&clade=mammal&org=Human&db=hg19&position=BC127192) | [AK128563 (5318)](http://genome.ucsc.edu/cgi-bin/hgTracks?hgHubConnect.destUrl=..%2Fcgi-bin%2FhgTracks&clade=mammal&org=Human&db=hg19&position=AK128563) | [SHANK2 (9677)](http://genome.ucsc.edu/cgi-bin/hgTracks?hgHubConnect.destUrl=..%2Fcgi-bin%2FhgTracks&clade=mammal&org=Human&db=hg19&position=SHANK2) | A/0.996/0.062/0.1047 | A/0.996/0.072/0.1017 | A/0.999/0.474/3.04e-06 | | A/0.998/0.239/0.01402 | | NA | | A/0.004/-0.055/0.1435 |
| rs144903619 | 11 | [BC127192](http://genome.ucsc.edu/cgi-bin/hgTracks?hgHubConnect.destUrl=..%2Fcgi-bin%2FhgTracks&clade=mammal&org=Human&db=hg19&position=BC127192) | [AK128563 (24334)](http://genome.ucsc.edu/cgi-bin/hgTracks?hgHubConnect.destUrl=..%2Fcgi-bin%2FhgTracks&clade=mammal&org=Human&db=hg19&position=AK128563) | [BC131767 (103798)](http://genome.ucsc.edu/cgi-bin/hgTracks?hgHubConnect.destUrl=..%2Fcgi-bin%2FhgTracks&clade=mammal&org=Human&db=hg19&position=BC131767) | T/0.998/0.042/0.3701 | T/0.998/0.078/0.149 | T/1/0.621/1.56e-06 | | T/0.999/0.313/0.00992 | | NA | | T/0.002/-0.078/0.1488 |
| rs181309176 | 11 |  | [CLPB (199324)](http://genome.ucsc.edu/cgi-bin/hgTracks?hgHubConnect.destUrl=..%2Fcgi-bin%2FhgTracks&clade=mammal&org=Human&db=hg19&position=CLPB) | [AL832797 (62731)](http://genome.ucsc.edu/cgi-bin/hgTracks?hgHubConnect.destUrl=..%2Fcgi-bin%2FhgTracks&clade=mammal&org=Human&db=hg19&position=AL832797) | NA | NA | T/0.999/0.282/2.69e-06 | | T/0.997/0.124/0.02775 | | NA | | NA |
| rs115148171 | 11 |  | [GUCY2E (8767)](http://genome.ucsc.edu/cgi-bin/hgTracks?hgHubConnect.destUrl=..%2Fcgi-bin%2FhgTracks&clade=mammal&org=Human&db=hg19&position=GUCY2E) | [TSKU (52684)](http://genome.ucsc.edu/cgi-bin/hgTracks?hgHubConnect.destUrl=..%2Fcgi-bin%2FhgTracks&clade=mammal&org=Human&db=hg19&position=TSKU) | NA | NA | C/0.999/0.359/2e-06 | | C/0.998/0.226/0.00568 | | NA | | NA |
| rs138394018 | 11 | [MYO7A](http://genome.ucsc.edu/cgi-bin/hgTracks?hgHubConnect.destUrl=..%2Fcgi-bin%2FhgTracks&clade=mammal&org=Human&db=hg19&position=MYO7A) | [CAPN5 (82919)](http://genome.ucsc.edu/cgi-bin/hgTracks?hgHubConnect.destUrl=..%2Fcgi-bin%2FhgTracks&clade=mammal&org=Human&db=hg19&position=CAPN5) | [GDPD4 (7485)](http://genome.ucsc.edu/cgi-bin/hgTracks?hgHubConnect.destUrl=..%2Fcgi-bin%2FhgTracks&clade=mammal&org=Human&db=hg19&position=GDPD4) | NA | NA | G/0.997/0.162/4.25e-06 | | G/0.995/0.12/0.01593 | | NA | | NA |
| rs150923551 | 11 |  | [GDPD4 (7477)](http://genome.ucsc.edu/cgi-bin/hgTracks?hgHubConnect.destUrl=..%2Fcgi-bin%2FhgTracks&clade=mammal&org=Human&db=hg19&position=GDPD4) | [PAK1 (27119)](http://genome.ucsc.edu/cgi-bin/hgTracks?hgHubConnect.destUrl=..%2Fcgi-bin%2FhgTracks&clade=mammal&org=Human&db=hg19&position=PAK1) | NA | NA | A/0.997/0.165/1.82e-06 | | A/0.994/0.113/0.01635 | | NA | | NA |
| rs181563674 | 11 |  | [GDPD4 (14370)](http://genome.ucsc.edu/cgi-bin/hgTracks?hgHubConnect.destUrl=..%2Fcgi-bin%2FhgTracks&clade=mammal&org=Human&db=hg19&position=GDPD4) | [PAK1 (20226)](http://genome.ucsc.edu/cgi-bin/hgTracks?hgHubConnect.destUrl=..%2Fcgi-bin%2FhgTracks&clade=mammal&org=Human&db=hg19&position=PAK1) | NA | NA | T/0.997/0.165/1.8e-06 | | T/0.994/0.112/0.01635 | | NA | | NA |
| rs146103351 | 11 | [PAK1](http://genome.ucsc.edu/cgi-bin/hgTracks?hgHubConnect.destUrl=..%2Fcgi-bin%2FhgTracks&clade=mammal&org=Human&db=hg19&position=PAK1) | [GDPD4 (35488)](http://genome.ucsc.edu/cgi-bin/hgTracks?hgHubConnect.destUrl=..%2Fcgi-bin%2FhgTracks&clade=mammal&org=Human&db=hg19&position=GDPD4) |  | NA | NA | A/0.996/0.167/9.99e-07 | | A/0.994/0.112/0.01496 | | NA | | NA |
| rs139530741 | 11 |  | [PAK1 (196534)](http://genome.ucsc.edu/cgi-bin/hgTracks?hgHubConnect.destUrl=..%2Fcgi-bin%2FhgTracks&clade=mammal&org=Human&db=hg19&position=PAK1) | [AQP11 (33839)](http://genome.ucsc.edu/cgi-bin/hgTracks?hgHubConnect.destUrl=..%2Fcgi-bin%2FhgTracks&clade=mammal&org=Human&db=hg19&position=AQP11) | NA | NA | G/0.993/0.111/1.24e-06 | | G/0.989/0.108/0.00083 | | NA | | NA |
| rs144029093 | 11 |  |  |  | T/0.995/-0.025/0.2356 | T/0.996/-0.05/0.09453 | T/0.999/0.406/7.76e-08 | | T/0.998/0.305/0.00015 | | T/0.005/-0.008/0.6347 | | T/0.005/0.017/0.3883 |
| rs150829342 | 11 | [DLG2](http://genome.ucsc.edu/cgi-bin/hgTracks?hgHubConnect.destUrl=..%2Fcgi-bin%2FhgTracks&clade=mammal&org=Human&db=hg19&position=DLG2) |  |  | G/0.991/-0.018/0.2264 | G/0.991/-0.004/0.8471 | G/0.998/0.2/5.11e-08 | | G/0.994/0.11/0.00548 | | A/0.991/0.005/0.6708 | | A/0.991/-0.007/0.5961 |
| rs189780858 | 11 | [DLG2](http://genome.ucsc.edu/cgi-bin/hgTracks?hgHubConnect.destUrl=..%2Fcgi-bin%2FhgTracks&clade=mammal&org=Human&db=hg19&position=DLG2) |  |  | T/0.989/-0.011/0.4276 | T/0.989/0.001/0.9582 | T/0.998/0.192/5.72e-08 | | T/0.994/0.103/0.00638 | | T/0.01/-0.004/0.6599 | | T/0.01/0.004/0.7723 |
| rs192772225 | 11 | [DLG2](http://genome.ucsc.edu/cgi-bin/hgTracks?hgHubConnect.destUrl=..%2Fcgi-bin%2FhgTracks&clade=mammal&org=Human&db=hg19&position=DLG2) |  |  | A/0.998/-0.064/0.1068 | A/0.998/-0.046/0.3556 | A/1/0.47/8.61e-08 | | A/0.998/0.23/0.00534 | | A/0.002/-0.01/0.7533 | | A/0.002/0.031/0.413 |
| rs149504921 | 11 | [HP11113](http://genome.ucsc.edu/cgi-bin/hgTracks?hgHubConnect.destUrl=..%2Fcgi-bin%2FhgTracks&clade=mammal&org=Human&db=hg19&position=HP11113) |  |  | NA | NA | C/0.992/0.097/4.51e-06 | | C/0.986/0.064/0.02296 | | NA | | NA |
| rs142535958 | 11 |  | [KIAA1377 (4550)](http://genome.ucsc.edu/cgi-bin/hgTracks?hgHubConnect.destUrl=..%2Fcgi-bin%2FhgTracks&clade=mammal&org=Human&db=hg19&position=KIAA1377) | [C11orf70 (41825)](http://genome.ucsc.edu/cgi-bin/hgTracks?hgHubConnect.destUrl=..%2Fcgi-bin%2FhgTracks&clade=mammal&org=Human&db=hg19&position=C11orf70) | NA | NA | A/0.999/0.43/2.48e-06 | | A/0.998/0.218/0.01345 | | NA | | NA |
| rs188240612 | 11 | [C11orf70](http://genome.ucsc.edu/cgi-bin/hgTracks?hgHubConnect.destUrl=..%2Fcgi-bin%2FhgTracks&clade=mammal&org=Human&db=hg19&position=C11orf70) | [KIAA1377 (72701)](http://genome.ucsc.edu/cgi-bin/hgTracks?hgHubConnect.destUrl=..%2Fcgi-bin%2FhgTracks&clade=mammal&org=Human&db=hg19&position=KIAA1377) | [BC040646 (34122)](http://genome.ucsc.edu/cgi-bin/hgTracks?hgHubConnect.destUrl=..%2Fcgi-bin%2FhgTracks&clade=mammal&org=Human&db=hg19&position=BC040646) | NA | NA | A/0.999/0.487/8.9e-07 | | A/0.998/0.246/0.00833 | | NA | | NA |
| rs189693121 | 11 | [BUD13](http://genome.ucsc.edu/cgi-bin/hgTracks?hgHubConnect.destUrl=..%2Fcgi-bin%2FhgTracks&clade=mammal&org=Human&db=hg19&position=BUD13) |  | [ZNF259 (25180)](http://genome.ucsc.edu/cgi-bin/hgTracks?hgHubConnect.destUrl=..%2Fcgi-bin%2FhgTracks&clade=mammal&org=Human&db=hg19&position=ZNF259) | NA | NA | A/0.999/0.441/1.59e-06 | | A/0.998/0.236/0.0097 | | NA | | NA |
| rs181290688 | 11 | [BUD13](http://genome.ucsc.edu/cgi-bin/hgTracks?hgHubConnect.destUrl=..%2Fcgi-bin%2FhgTracks&clade=mammal&org=Human&db=hg19&position=BUD13) |  | [ZNF259 (25179)](http://genome.ucsc.edu/cgi-bin/hgTracks?hgHubConnect.destUrl=..%2Fcgi-bin%2FhgTracks&clade=mammal&org=Human&db=hg19&position=ZNF259) | NA | NA | A/0.999/0.441/1.6e-06 | | A/0.998/0.236/0.00972 | | NA | | NA |
| rs7941140 | 11 |  | [SIK3 (27768)](http://genome.ucsc.edu/cgi-bin/hgTracks?hgHubConnect.destUrl=..%2Fcgi-bin%2FhgTracks&clade=mammal&org=Human&db=hg19&position=SIK3) | [AB231710 (10500)](http://genome.ucsc.edu/cgi-bin/hgTracks?hgHubConnect.destUrl=..%2Fcgi-bin%2FhgTracks&clade=mammal&org=Human&db=hg19&position=AB231710) | NA | NA | T/0.909/0.027/8.77e-07 | | T/0.884/0.014/0.1247 | | NA | | NA |
| rs188193075 | 11 |  | [SLC37A4 (507)](http://genome.ucsc.edu/cgi-bin/hgTracks?hgHubConnect.destUrl=..%2Fcgi-bin%2FhgTracks&clade=mammal&org=Human&db=hg19&position=SLC37A4) | [HYOU1 (12772)](http://genome.ucsc.edu/cgi-bin/hgTracks?hgHubConnect.destUrl=..%2Fcgi-bin%2FhgTracks&clade=mammal&org=Human&db=hg19&position=HYOU1) | NA | NA | G/0.998/0.228/2.53e-06 | | G/0.996/0.122/0.02163 | | NA | | NA |
| rs150556575 | 11 |  | [SLC37A4 (622)](http://genome.ucsc.edu/cgi-bin/hgTracks?hgHubConnect.destUrl=..%2Fcgi-bin%2FhgTracks&clade=mammal&org=Human&db=hg19&position=SLC37A4) | [HYOU1 (12657)](http://genome.ucsc.edu/cgi-bin/hgTracks?hgHubConnect.destUrl=..%2Fcgi-bin%2FhgTracks&clade=mammal&org=Human&db=hg19&position=HYOU1) | NA | NA | G/0.998/0.228/2.55e-06 | | G/0.996/0.122/0.02165 | | NA | | NA |
| rs185836767 | 11 |  | [SLC37A4 (11438)](http://genome.ucsc.edu/cgi-bin/hgTracks?hgHubConnect.destUrl=..%2Fcgi-bin%2FhgTracks&clade=mammal&org=Human&db=hg19&position=SLC37A4) | [HYOU1 (1841)](http://genome.ucsc.edu/cgi-bin/hgTracks?hgHubConnect.destUrl=..%2Fcgi-bin%2FhgTracks&clade=mammal&org=Human&db=hg19&position=HYOU1) | NA | NA | C/0.998/0.226/2.39e-06 | | C/0.996/0.12/0.02117 | | NA | | NA |
| rs147134271 | 11 | [HYOU1](http://genome.ucsc.edu/cgi-bin/hgTracks?hgHubConnect.destUrl=..%2Fcgi-bin%2FhgTracks&clade=mammal&org=Human&db=hg19&position=HYOU1) | [SLC37A4 (22523)](http://genome.ucsc.edu/cgi-bin/hgTracks?hgHubConnect.destUrl=..%2Fcgi-bin%2FhgTracks&clade=mammal&org=Human&db=hg19&position=SLC37A4) | [VPS11 (14353)](http://genome.ucsc.edu/cgi-bin/hgTracks?hgHubConnect.destUrl=..%2Fcgi-bin%2FhgTracks&clade=mammal&org=Human&db=hg19&position=VPS11) | NA | NA | G/0.998/0.231/1.2e-06 | | G/0.995/0.122/0.01899 | | NA | | NA |
| rs183974900 | 11 |  | [MIR100HG (31147)](http://genome.ucsc.edu/cgi-bin/hgTracks?hgHubConnect.destUrl=..%2Fcgi-bin%2FhgTracks&clade=mammal&org=Human&db=hg19&position=MIR100HG) |  | G/0.998/0.039/0.4201 | G/0.998/0.019/0.6924 | G/0.999/0.398/1.14e-06 | | G/0.999/0.232/0.00651 | | A/0.998/0.086/0.00659 | | A/0.998/0.017/0.5985 |
| rs145803288 | 11 |  |  | [UBASH3B (35738)](http://genome.ucsc.edu/cgi-bin/hgTracks?hgHubConnect.destUrl=..%2Fcgi-bin%2FhgTracks&clade=mammal&org=Human&db=hg19&position=UBASH3B) | T/0.999/0.034/0.408 | T/0.998/-0.003/0.9486 | T/1/0.348/2.09e-06 | | T/0.999/0.213/0.01001 | | T/0.002/-0.078/0.00543 | | T/0.002/-0.01/0.7447 |
| rs190169131 | 11 |  |  |  | G/0.99/-0.004/0.8473 | G/0.99/-0.049/0.06662 | G/0.998/0.315/1.3e-06 | | G/0.997/0.195/0.00416 | | A/0.991/0.025/0.1242 | | A/0.99/-0.001/0.9716 |
| rs187327935 | 11 |  |  |  | T/0.989/0.009/0.6067 | T/0.989/0.009/0.6938 | T/0.998/0.306/3.78e-08 | | T/0.996/0.165/0.01147 | | T/0.01/-0.031/0.03822 | | T/0.01/-0.013/0.4911 |
| rs191110399 | 11 |  |  |  | G/0.989/0.009/0.5956 | G/0.989/0.01/0.6668 | G/0.998/0.3/5.43e-08 | | G/0.996/0.166/0.01126 | | T/0.99/0.03/0.04135 | | T/0.99/0.013/0.5008 |
| rs141955301 | 12 | [CACNA1C](http://genome.ucsc.edu/cgi-bin/hgTracks?hgHubConnect.destUrl=..%2Fcgi-bin%2FhgTracks&clade=mammal&org=Human&db=hg19&position=CACNA1C) | [AK308652 (128875)](http://genome.ucsc.edu/cgi-bin/hgTracks?hgHubConnect.destUrl=..%2Fcgi-bin%2FhgTracks&clade=mammal&org=Human&db=hg19&position=AK308652) |  | NA | NA | T/1/0.631/4.83e-06 | | T/0.999/0.405/0.00233 | | NA | | NA |
| rs191413231 | 12 |  |  |  | A/0.993/0.048/0.01035 | A/0.993/0.057/0.01457 | A/0.999/0.299/1.89e-06 | | A/0.997/0.228/0.00153 | | A/0.006/-0.058/6.28e-05 | | A/0.007/-0.052/0.00168 |
| rs146978549 | 12 |  |  |  | NA | NA | G/0.999/0.423/2.99e-06 | | G/0.998/0.307/0.00888 | | NA | | NA |
| rs184061709 | 12 | [ST8SIA1](http://genome.ucsc.edu/cgi-bin/hgTracks?hgHubConnect.destUrl=..%2Fcgi-bin%2FhgTracks&clade=mammal&org=Human&db=hg19&position=ST8SIA1) |  | [KIAA0528 (139239)](http://genome.ucsc.edu/cgi-bin/hgTracks?hgHubConnect.destUrl=..%2Fcgi-bin%2FhgTracks&clade=mammal&org=Human&db=hg19&position=KIAA0528) | NA | NA | G/1/0.38/2.87e-06 | | G/0.998/0.182/0.01614 | | NA | | NA |
| rs182272664 | 12 | [ST8SIA1](http://genome.ucsc.edu/cgi-bin/hgTracks?hgHubConnect.destUrl=..%2Fcgi-bin%2FhgTracks&clade=mammal&org=Human&db=hg19&position=ST8SIA1) |  | [KIAA0528 (136577)](http://genome.ucsc.edu/cgi-bin/hgTracks?hgHubConnect.destUrl=..%2Fcgi-bin%2FhgTracks&clade=mammal&org=Human&db=hg19&position=KIAA0528) | NA | NA | A/0.999/0.383/2.18e-06 | | A/0.998/0.182/0.01569 | | NA | | NA |
| rs139337462 | 12 |  | [TMTC1 (190129)](http://genome.ucsc.edu/cgi-bin/hgTracks?hgHubConnect.destUrl=..%2Fcgi-bin%2FhgTracks&clade=mammal&org=Human&db=hg19&position=TMTC1) |  | NA | NA | G/0.999/0.296/4.52e-06 | | G/0.997/0.08/0.2228 | | NA | | NA |
| rs138037897 | 12 |  | [TMTC1 (191327)](http://genome.ucsc.edu/cgi-bin/hgTracks?hgHubConnect.destUrl=..%2Fcgi-bin%2FhgTracks&clade=mammal&org=Human&db=hg19&position=TMTC1) |  | NA | NA | A/0.999/0.298/4.5e-06 | | A/0.997/0.08/0.2221 | | NA | | NA |
| rs143053231 | 12 | [ITGA7](http://genome.ucsc.edu/cgi-bin/hgTracks?hgHubConnect.destUrl=..%2Fcgi-bin%2FhgTracks&clade=mammal&org=Human&db=hg19&position=ITGA7) | [METTL7B (12638)](http://genome.ucsc.edu/cgi-bin/hgTracks?hgHubConnect.destUrl=..%2Fcgi-bin%2FhgTracks&clade=mammal&org=Human&db=hg19&position=METTL7B) | [BLOC1S1 (18785)](http://genome.ucsc.edu/cgi-bin/hgTracks?hgHubConnect.destUrl=..%2Fcgi-bin%2FhgTracks&clade=mammal&org=Human&db=hg19&position=BLOC1S1) | NA | NA | G/1/0.415/1e-06 | | G/0.998/0.23/0.0042 | | NA | | NA |
| rs11174267 | 12 | [FAM19A2](http://genome.ucsc.edu/cgi-bin/hgTracks?hgHubConnect.destUrl=..%2Fcgi-bin%2FhgTracks&clade=mammal&org=Human&db=hg19&position=FAM19A2) |  |  | A/0.381/0.001/0.6022 | A/0.381/0.001/0.7378 | A/0.139/-0.025/6.14e-08 | | A/0.139/-0.016/0.04372 | | A/0.366/0.004/0.02847 | | A/0.413/0.001/0.5794 |
| rs12815303 | 12 | [FAM19A2](http://genome.ucsc.edu/cgi-bin/hgTracks?hgHubConnect.destUrl=..%2Fcgi-bin%2FhgTracks&clade=mammal&org=Human&db=hg19&position=FAM19A2) |  |  | G/0.618/-0.002/0.5815 | G/0.614/-0.001/0.7471 | G/0.86/0.024/7.9e-08 | | G/0.826/0.016/0.04684 | | A/0.593/0.004/0.03045 | | A/0.486/0.001/0.5579 |
| rs12817007 | 12 | [FAM19A2](http://genome.ucsc.edu/cgi-bin/hgTracks?hgHubConnect.destUrl=..%2Fcgi-bin%2FhgTracks&clade=mammal&org=Human&db=hg19&position=FAM19A2) |  |  | C/0.618/-0.002/0.5805 | C/0.614/-0.001/0.7476 | C/0.86/0.024/8.45e-08 | | C/0.826/0.016/0.04759 | | T/0.661/0.004/0.03067 | | T/0.616/0.001/0.5848 |
| rs12817011 | 12 | [FAM19A2](http://genome.ucsc.edu/cgi-bin/hgTracks?hgHubConnect.destUrl=..%2Fcgi-bin%2FhgTracks&clade=mammal&org=Human&db=hg19&position=FAM19A2) |  |  | C/0.618/-0.002/0.5796 | C/0.614/-0.001/0.7482 | C/0.86/0.024/8.44e-08 | | C/0.826/0.016/0.04783 | | T/0.661/0.004/0.03067 | | T/0.616/0.001/0.5848 |
| rs2289936 | 12 | [WIF1](http://genome.ucsc.edu/cgi-bin/hgTracks?hgHubConnect.destUrl=..%2Fcgi-bin%2FhgTracks&clade=mammal&org=Human&db=hg19&position=WIF1) | [FLJ41278 (100165)](http://genome.ucsc.edu/cgi-bin/hgTracks?hgHubConnect.destUrl=..%2Fcgi-bin%2FhgTracks&clade=mammal&org=Human&db=hg19&position=FLJ41278) | [LEMD3 (91883)](http://genome.ucsc.edu/cgi-bin/hgTracks?hgHubConnect.destUrl=..%2Fcgi-bin%2FhgTracks&clade=mammal&org=Human&db=hg19&position=LEMD3) | G/0.941/-0.002/0.7779 | G/0.94/-0.002/0.824 | G/0.97/0.047/4.17e-06 | | G/0.957/0.01/0.5364 | | A/0.942/0.009/0.028 | | A/0.934/0.003/0.507 |
| rs182408739 | 12 | [MSRB3](http://genome.ucsc.edu/cgi-bin/hgTracks?hgHubConnect.destUrl=..%2Fcgi-bin%2FhgTracks&clade=mammal&org=Human&db=hg19&position=MSRB3) | [LEMD3 (84508)](http://genome.ucsc.edu/cgi-bin/hgTracks?hgHubConnect.destUrl=..%2Fcgi-bin%2FhgTracks&clade=mammal&org=Human&db=hg19&position=LEMD3) |  | NA | NA | C/0.999/0.299/1.23e-06 | | C/0.997/0.181/0.00318 | | NA | | NA |
| rs143566192 | 12 |  | [BC043551 (42667)](http://genome.ucsc.edu/cgi-bin/hgTracks?hgHubConnect.destUrl=..%2Fcgi-bin%2FhgTracks&clade=mammal&org=Human&db=hg19&position=BC043551) | [RPSAP52 (158042)](http://genome.ucsc.edu/cgi-bin/hgTracks?hgHubConnect.destUrl=..%2Fcgi-bin%2FhgTracks&clade=mammal&org=Human&db=hg19&position=RPSAP52) | NA | NA | G/0.999/0.242/4.12e-06 | | G/0.997/0.185/0.00161 | | NA | | NA |
| rs11175944 | 12 | [AK128707](http://genome.ucsc.edu/cgi-bin/hgTracks?hgHubConnect.destUrl=..%2Fcgi-bin%2FhgTracks&clade=mammal&org=Human&db=hg19&position=AK128707) | [HMGA2 (5097)](http://genome.ucsc.edu/cgi-bin/hgTracks?hgHubConnect.destUrl=..%2Fcgi-bin%2FhgTracks&clade=mammal&org=Human&db=hg19&position=HMGA2) |  | G/0.073/0/0.9896 | G/0.073/-0.001/0.8923 | C/0.312/-0.016/4.91e-06 | | C/0.312/-0.017/0.00648 | | C/0.663/0.01/8.56e-05 | | C/0.488/-0.002/0.6765 |
| rs74910874 | 12 | [GRIP1](http://genome.ucsc.edu/cgi-bin/hgTracks?hgHubConnect.destUrl=..%2Fcgi-bin%2FhgTracks&clade=mammal&org=Human&db=hg19&position=GRIP1) |  |  | T/0.994/0.001/0.9794 | T/0.994/0.046/0.1224 | T/0.999/0.393/3.8e-09 | | T/0.997/0.246/0.00011 | | A/0.994/0.017/0.3415 | | A/0.994/0.03/0.1925 |
| rs147616082 | 12 | [GRIP1](http://genome.ucsc.edu/cgi-bin/hgTracks?hgHubConnect.destUrl=..%2Fcgi-bin%2FhgTracks&clade=mammal&org=Human&db=hg19&position=GRIP1) |  |  | A/0.997/-0.004/0.8799 | A/0.998/0.098/0.02354 | A/1/0.764/1.04e-06 | | A/0.999/0.503/5e-04 | | A/0.003/-0.008/0.7401 | | A/0.002/-0.088/0.02317 |
| rs184699315 | 12 | [TSPAN8](http://genome.ucsc.edu/cgi-bin/hgTracks?hgHubConnect.destUrl=..%2Fcgi-bin%2FhgTracks&clade=mammal&org=Human&db=hg19&position=TSPAN8) |  | [LGR5 (183033)](http://genome.ucsc.edu/cgi-bin/hgTracks?hgHubConnect.destUrl=..%2Fcgi-bin%2FhgTracks&clade=mammal&org=Human&db=hg19&position=LGR5) | T/0.999/0.043/0.5162 | T/0.999/-0.029/0.7049 | T/0.999/0.478/4.01e-06 | | T/0.998/0.246/0.01317 | | NA | | T/0.001/0.026/0.7271 |
| rs186586221 | 12 | [KCNC2](http://genome.ucsc.edu/cgi-bin/hgTracks?hgHubConnect.destUrl=..%2Fcgi-bin%2FhgTracks&clade=mammal&org=Human&db=hg19&position=KCNC2) |  | [AK093193 (54196)](http://genome.ucsc.edu/cgi-bin/hgTracks?hgHubConnect.destUrl=..%2Fcgi-bin%2FhgTracks&clade=mammal&org=Human&db=hg19&position=AK093193) | NA | NA | C/0.999/0.332/1.38e-06 | | C/0.998/0.184/0.008 | | NA | | NA |
| rs140785266 | 12 |  |  | [RASSF9 (2407)](http://genome.ucsc.edu/cgi-bin/hgTracks?hgHubConnect.destUrl=..%2Fcgi-bin%2FhgTracks&clade=mammal&org=Human&db=hg19&position=RASSF9) | G/0.997/-0.006/0.8277 | G/0.996/-0.025/0.4222 | G/1/0.412/3.97e-06 | | G/0.999/0.246/0.0038 | | A/0.995/-0.006/0.7248 | | A/0.994/-0.034/0.05555 |
| rs79843055 | 12 |  | [NTS (7689)](http://genome.ucsc.edu/cgi-bin/hgTracks?hgHubConnect.destUrl=..%2Fcgi-bin%2FhgTracks&clade=mammal&org=Human&db=hg19&position=NTS) | [MGAT4C (88577)](http://genome.ucsc.edu/cgi-bin/hgTracks?hgHubConnect.destUrl=..%2Fcgi-bin%2FhgTracks&clade=mammal&org=Human&db=hg19&position=MGAT4C) | C/0.996/-0.009/0.6987 | C/0.996/-0.03/0.2721 | C/1/0.425/4.15e-07 | | C/0.998/0.235/0.003 | | A/0.995/-0.006/0.7043 | | A/0.994/-0.035/0.03891 |
| rs147510300 | 12 |  | [NTS (41778)](http://genome.ucsc.edu/cgi-bin/hgTracks?hgHubConnect.destUrl=..%2Fcgi-bin%2FhgTracks&clade=mammal&org=Human&db=hg19&position=NTS) | [MGAT4C (54488)](http://genome.ucsc.edu/cgi-bin/hgTracks?hgHubConnect.destUrl=..%2Fcgi-bin%2FhgTracks&clade=mammal&org=Human&db=hg19&position=MGAT4C) | NA | NA | G/1/0.498/8.42e-07 | | G/0.999/0.268/0.00437 | | NA | | NA |
| rs150696478 | 12 |  | [NTS (44618)](http://genome.ucsc.edu/cgi-bin/hgTracks?hgHubConnect.destUrl=..%2Fcgi-bin%2FhgTracks&clade=mammal&org=Human&db=hg19&position=NTS) | [MGAT4C (51648)](http://genome.ucsc.edu/cgi-bin/hgTracks?hgHubConnect.destUrl=..%2Fcgi-bin%2FhgTracks&clade=mammal&org=Human&db=hg19&position=MGAT4C) | NA | NA | A/1/0.498/8.47e-07 | | A/0.999/0.268/0.00438 | | NA | | NA |
| rs76177788 | 12 | [MGAT4C](http://genome.ucsc.edu/cgi-bin/hgTracks?hgHubConnect.destUrl=..%2Fcgi-bin%2FhgTracks&clade=mammal&org=Human&db=hg19&position=MGAT4C) |  |  | T/0.997/-0.006/0.8127 | T/0.996/-0.036/0.2096 | T/1/0.49/4e-07 | | T/0.999/0.292/0.0019 | | T/0.004/0.005/0.7973 | | T/0.005/0.037/0.05014 |
| rs188585019 | 12 | [MGAT4C](http://genome.ucsc.edu/cgi-bin/hgTracks?hgHubConnect.destUrl=..%2Fcgi-bin%2FhgTracks&clade=mammal&org=Human&db=hg19&position=MGAT4C) |  |  | A/0.997/-0.006/0.8133 | A/0.996/-0.036/0.2093 | A/0.999/0.49/6.97e-07 | | A/0.998/0.299/0.002 | | A/0.004/0.005/0.8008 | | A/0.005/0.037/0.05423 |
| rs187003284 | 12 | [MGAT4C](http://genome.ucsc.edu/cgi-bin/hgTracks?hgHubConnect.destUrl=..%2Fcgi-bin%2FhgTracks&clade=mammal&org=Human&db=hg19&position=MGAT4C) |  |  | T/0.997/-0.007/0.7995 | T/0.996/-0.039/0.2029 | T/1/0.545/7.27e-07 | | T/0.999/0.338/0.00188 | | A/0.996/-0.004/0.8235 | | A/0.996/-0.038/0.06626 |
| rs140444490 | 12 | [MGAT4C](http://genome.ucsc.edu/cgi-bin/hgTracks?hgHubConnect.destUrl=..%2Fcgi-bin%2FhgTracks&clade=mammal&org=Human&db=hg19&position=MGAT4C) |  |  | C/0.997/-0.013/0.7015 | C/0.996/-0.053/0.1596 | C/0.999/0.431/1e-06 | | C/0.998/0.311/0.00123 | | T/0.997/0.016/0.5434 | | T/0.997/-0.044/0.12 |
| rs142054194 | 12 | [MGAT4C](http://genome.ucsc.edu/cgi-bin/hgTracks?hgHubConnect.destUrl=..%2Fcgi-bin%2FhgTracks&clade=mammal&org=Human&db=hg19&position=MGAT4C) |  |  | T/0.997/-0.013/0.7006 | T/0.996/-0.053/0.1591 | T/0.999/0.43/9.82e-07 | | T/0.998/0.311/0.00122 | | T/0.003/-0.016/0.5361 | | T/0.003/0.045/0.1198 |
| rs183244340 | 12 | [MGAT4C](http://genome.ucsc.edu/cgi-bin/hgTracks?hgHubConnect.destUrl=..%2Fcgi-bin%2FhgTracks&clade=mammal&org=Human&db=hg19&position=MGAT4C) |  |  | T/0.998/-0.02/0.6163 | T/0.997/-0.066/0.1259 | T/0.999/0.366/4.62e-07 | | T/0.998/0.276/0.00064 | | NA | | A/0.997/-0.066/0.1259 |
| rs187035304 | 12 | [MGAT4C](http://genome.ucsc.edu/cgi-bin/hgTracks?hgHubConnect.destUrl=..%2Fcgi-bin%2FhgTracks&clade=mammal&org=Human&db=hg19&position=MGAT4C) |  |  | T/0.998/-0.027/0.5247 | T/0.998/-0.076/0.09835 | T/0.999/0.318/6.61e-07 | | T/0.998/0.252/0.00053 | | NA | | T/0.002/0.076/0.09865 |
| rs187039407 | 12 |  |  |  | T/0.998/-0.024/0.555 | T/0.997/-0.07/0.1136 | T/0.999/0.296/6.39e-07 | | T/0.998/0.234/0.00061 | | NA | | T/0.003/0.07/0.1134 |
| rs144148493 | 12 |  |  |  | G/0.998/-0.023/0.5664 | G/0.998/-0.07/0.1167 | G/0.999/0.295/6.54e-07 | | G/0.998/0.232/0.00067 | | NA | | A/0.998/-0.07/0.1167 |
| rs77628219 | 12 |  |  |  | G/0.998/-0.028/0.4293 | G/0.997/-0.063/0.118 | G/0.999/0.277/1.19e-06 | | G/0.998/0.22/0.00093 | | A/0.998/0.032/0.2434 | | A/0.998/-0.06/0.07436 |
| rs187607716 | 12 |  | [KERA (35386)](http://genome.ucsc.edu/cgi-bin/hgTracks?hgHubConnect.destUrl=..%2Fcgi-bin%2FhgTracks&clade=mammal&org=Human&db=hg19&position=KERA) | [LUM (9714)](http://genome.ucsc.edu/cgi-bin/hgTracks?hgHubConnect.destUrl=..%2Fcgi-bin%2FhgTracks&clade=mammal&org=Human&db=hg19&position=LUM) | NA | NA | G/1/0.451/1.39e-06 | | G/0.999/0.264/0.00247 | | NA | | NA |
| rs190262953 | 12 |  |  |  | NA | NA | T/1/0.427/2.52e-06 | | T/0.999/0.284/0.00176 | | NA | | NA |
| rs182057829 | 12 |  | [NEDD1 (64858)](http://genome.ucsc.edu/cgi-bin/hgTracks?hgHubConnect.destUrl=..%2Fcgi-bin%2FhgTracks&clade=mammal&org=Human&db=hg19&position=NEDD1) |  | G/0.999/0.134/0.00771 | G/0.998/0.08/0.1454 | G/1/0.557/4.59e-06 | | G/0.999/0.345/0.00298 | | A/0.999/0.125/0.00305 | | A/0.998/0.05/0.3207 |
| rs118152513 | 12 | [ANO4](http://genome.ucsc.edu/cgi-bin/hgTracks?hgHubConnect.destUrl=..%2Fcgi-bin%2FhgTracks&clade=mammal&org=Human&db=hg19&position=ANO4) | [GAS2L3 (165567)](http://genome.ucsc.edu/cgi-bin/hgTracks?hgHubConnect.destUrl=..%2Fcgi-bin%2FhgTracks&clade=mammal&org=Human&db=hg19&position=GAS2L3) |  | C/0.996/0.012/0.6424 | C/0.996/-0.054/0.08801 | C/0.999/0.349/8.56e-07 | | C/0.998/0.187/0.00604 | | T/0.996/0.033/0.0726 | | T/0.995/-0.033/0.101 |
| rs183825572 | 12 | [ANO4](http://genome.ucsc.edu/cgi-bin/hgTracks?hgHubConnect.destUrl=..%2Fcgi-bin%2FhgTracks&clade=mammal&org=Human&db=hg19&position=ANO4) | [GAS2L3 (246160)](http://genome.ucsc.edu/cgi-bin/hgTracks?hgHubConnect.destUrl=..%2Fcgi-bin%2FhgTracks&clade=mammal&org=Human&db=hg19&position=GAS2L3) |  | A/0.996/0.018/0.4817 | A/0.995/-0.046/0.134 | A/1/0.379/8.69e-07 | | A/0.998/0.191/0.00726 | | A/0.005/-0.035/0.04849 | | A/0.005/0.028/0.1533 |
| rs188024766 | 12 | [ANO4](http://genome.ucsc.edu/cgi-bin/hgTracks?hgHubConnect.destUrl=..%2Fcgi-bin%2FhgTracks&clade=mammal&org=Human&db=hg19&position=ANO4) |  |  | G/0.996/0.025/0.3097 | G/0.995/-0.038/0.2071 | G/1/0.377/1.04e-06 | | G/0.998/0.192/0.00711 | | C/0.995/0.04/0.02577 | | C/0.994/-0.024/0.2255 |
| rs192568883 | 12 |  | [CRY1 (118528)](http://genome.ucsc.edu/cgi-bin/hgTracks?hgHubConnect.destUrl=..%2Fcgi-bin%2FhgTracks&clade=mammal&org=Human&db=hg19&position=CRY1) | [BTBD11 (106070)](http://genome.ucsc.edu/cgi-bin/hgTracks?hgHubConnect.destUrl=..%2Fcgi-bin%2FhgTracks&clade=mammal&org=Human&db=hg19&position=BTBD11) | T/0.999/0.098/0.1136 | T/0.999/0.098/0.1569 | T/1/0.809/9.3e-07 | | T/0.999/0.428/0.00515 | | T/0.002/-0.059/0.09714 | | T/0.003/-0.005/0.882 |
| rs181697701 | 12 |  | [CRY1 (123452)](http://genome.ucsc.edu/cgi-bin/hgTracks?hgHubConnect.destUrl=..%2Fcgi-bin%2FhgTracks&clade=mammal&org=Human&db=hg19&position=CRY1) | [BTBD11 (101146)](http://genome.ucsc.edu/cgi-bin/hgTracks?hgHubConnect.destUrl=..%2Fcgi-bin%2FhgTracks&clade=mammal&org=Human&db=hg19&position=BTBD11) | C/0.999/0.097/0.1149 | C/0.999/0.097/0.1578 | C/1/0.765/2.83e-06 | | C/0.999/0.43/0.00491 | | C/0.002/-0.058/0.1057 | | C/0.003/-0.005/0.8805 |
| rs189340018 | 12 |  | [CRY1 (141082)](http://genome.ucsc.edu/cgi-bin/hgTracks?hgHubConnect.destUrl=..%2Fcgi-bin%2FhgTracks&clade=mammal&org=Human&db=hg19&position=CRY1) | [BTBD11 (83516)](http://genome.ucsc.edu/cgi-bin/hgTracks?hgHubConnect.destUrl=..%2Fcgi-bin%2FhgTracks&clade=mammal&org=Human&db=hg19&position=BTBD11) | C/0.999/0.095/0.1189 | C/0.999/0.096/0.1623 | C/1/0.804/1.04e-06 | | C/0.999/0.428/0.00498 | | T/0.998/0.058/0.1036 | | T/0.997/0.004/0.9047 |
| rs192256367 | 12 |  |  | [BTBD11 (63791)](http://genome.ucsc.edu/cgi-bin/hgTracks?hgHubConnect.destUrl=..%2Fcgi-bin%2FhgTracks&clade=mammal&org=Human&db=hg19&position=BTBD11) | T/0.999/0.095/0.1188 | T/0.999/0.095/0.1649 | T/1/0.801/1.07e-06 | | T/0.999/0.43/0.00472 | | T/0.002/-0.056/0.1104 | | T/0.003/-0.003/0.9366 |
| rs182328274 | 12 |  |  | [BTBD11 (44394)](http://genome.ucsc.edu/cgi-bin/hgTracks?hgHubConnect.destUrl=..%2Fcgi-bin%2FhgTracks&clade=mammal&org=Human&db=hg19&position=BTBD11) | T/0.999/0.094/0.1196 | T/0.999/0.094/0.1657 | T/1/0.801/1.06e-06 | | T/0.999/0.429/0.00479 | | T/0.002/-0.056/0.1148 | | T/0.003/-0.002/0.9558 |
| rs183032784 | 12 | [KSR2](http://genome.ucsc.edu/cgi-bin/hgTracks?hgHubConnect.destUrl=..%2Fcgi-bin%2FhgTracks&clade=mammal&org=Human&db=hg19&position=KSR2) |  | [RFC5 (197291)](http://genome.ucsc.edu/cgi-bin/hgTracks?hgHubConnect.destUrl=..%2Fcgi-bin%2FhgTracks&clade=mammal&org=Human&db=hg19&position=RFC5) | A/0.997/-0.002/0.9257 | A/0.997/-0.009/0.7892 | A/1/0.471/5.87e-09 | | A/0.998/0.257/0.00068 | | A/0.003/-0.034/0.1043 | | A/0.003/0.001/0.9762 |
| rs185216905 | 12 |  | [NCOR2 (35182)](http://genome.ucsc.edu/cgi-bin/hgTracks?hgHubConnect.destUrl=..%2Fcgi-bin%2FhgTracks&clade=mammal&org=Human&db=hg19&position=NCOR2) | [SCARB1 (174981)](http://genome.ucsc.edu/cgi-bin/hgTracks?hgHubConnect.destUrl=..%2Fcgi-bin%2FhgTracks&clade=mammal&org=Human&db=hg19&position=SCARB1) | NA | NA | T/0.999/0.447/6.84e-07 | | T/0.997/0.205/0.02203 | | NA | | NA |
| rs190312121 | 13 |  | [PHF2P1 (39121)](http://genome.ucsc.edu/cgi-bin/hgTracks?hgHubConnect.destUrl=..%2Fcgi-bin%2FhgTracks&clade=mammal&org=Human&db=hg19&position=PHF2P1) | [TUBA3C (16365)](http://genome.ucsc.edu/cgi-bin/hgTracks?hgHubConnect.destUrl=..%2Fcgi-bin%2FhgTracks&clade=mammal&org=Human&db=hg19&position=TUBA3C) | NA | NA | A/1/0.764/4.58e-06 | | A/0.999/0.473/0.00209 | | NA | | NA |
| rs183816717 | 13 | [RB1](http://genome.ucsc.edu/cgi-bin/hgTracks?hgHubConnect.destUrl=..%2Fcgi-bin%2FhgTracks&clade=mammal&org=Human&db=hg19&position=RB1) | [LPAR6 (1189)](http://genome.ucsc.edu/cgi-bin/hgTracks?hgHubConnect.destUrl=..%2Fcgi-bin%2FhgTracks&clade=mammal&org=Human&db=hg19&position=LPAR6) | [RCBTB2 (43069)](http://genome.ucsc.edu/cgi-bin/hgTracks?hgHubConnect.destUrl=..%2Fcgi-bin%2FhgTracks&clade=mammal&org=Human&db=hg19&position=RCBTB2) | NA | NA | A/0.999/0.465/2.05e-06 | | A/0.998/0.276/0.00424 | | NA | | NA |
| rs185837131 | 13 | [RB1](http://genome.ucsc.edu/cgi-bin/hgTracks?hgHubConnect.destUrl=..%2Fcgi-bin%2FhgTracks&clade=mammal&org=Human&db=hg19&position=RB1) | [LPAR6 (10764)](http://genome.ucsc.edu/cgi-bin/hgTracks?hgHubConnect.destUrl=..%2Fcgi-bin%2FhgTracks&clade=mammal&org=Human&db=hg19&position=LPAR6) | [RCBTB2 (33494)](http://genome.ucsc.edu/cgi-bin/hgTracks?hgHubConnect.destUrl=..%2Fcgi-bin%2FhgTracks&clade=mammal&org=Human&db=hg19&position=RCBTB2) | NA | NA | T/0.999/0.453/1.84e-06 | | T/0.998/0.268/0.00418 | | NA | | NA |
| rs187785117 | 13 |  | [RCBTB2 (39753)](http://genome.ucsc.edu/cgi-bin/hgTracks?hgHubConnect.destUrl=..%2Fcgi-bin%2FhgTracks&clade=mammal&org=Human&db=hg19&position=RCBTB2) | [CYSLTR2 (77993)](http://genome.ucsc.edu/cgi-bin/hgTracks?hgHubConnect.destUrl=..%2Fcgi-bin%2FhgTracks&clade=mammal&org=Human&db=hg19&position=CYSLTR2) | NA | NA | T/0.999/0.405/2.27e-06 | | T/0.998/0.245/0.00392 | | NA | | NA |
| rs193251247 | 13 |  | [RCBTB2 (53062)](http://genome.ucsc.edu/cgi-bin/hgTracks?hgHubConnect.destUrl=..%2Fcgi-bin%2FhgTracks&clade=mammal&org=Human&db=hg19&position=RCBTB2) | [CYSLTR2 (64684)](http://genome.ucsc.edu/cgi-bin/hgTracks?hgHubConnect.destUrl=..%2Fcgi-bin%2FhgTracks&clade=mammal&org=Human&db=hg19&position=CYSLTR2) | NA | NA | A/0.999/0.4/2.29e-06 | | A/0.998/0.233/0.00561 | | NA | | NA |
| rs193182884 | 13 | [CYSLTR2](http://genome.ucsc.edu/cgi-bin/hgTracks?hgHubConnect.destUrl=..%2Fcgi-bin%2FhgTracks&clade=mammal&org=Human&db=hg19&position=CYSLTR2) | [RCBTB2 (122701)](http://genome.ucsc.edu/cgi-bin/hgTracks?hgHubConnect.destUrl=..%2Fcgi-bin%2FhgTracks&clade=mammal&org=Human&db=hg19&position=RCBTB2) |  | NA | NA | A/0.999/0.541/4.17e-06 | | A/0.999/0.416/0.00066 | | NA | | NA |
| rs188360948 | 13 |  |  |  | T/0.97/0.002/0.8733 | T/0.969/-0.016/0.3148 | T/0.949/-0.043/1.56e-06 | | T/0.962/-0.023/0.2473 | | T/0.042/0.025/0.00026 | | T/0.03/0.013/0.2574 |
| rs185603859 | 13 |  |  |  | G/0.991/0.018/0.3112 | G/0.991/0.023/0.339 | G/0.999/0.229/4.89e-06 | | G/0.996/0.09/0.07471 | | T/0.992/0.019/0.175 | | T/0.991/-0.011/0.5087 |
| rs182426545 | 13 |  |  | [OR7E156P (218222)](http://genome.ucsc.edu/cgi-bin/hgTracks?hgHubConnect.destUrl=..%2Fcgi-bin%2FhgTracks&clade=mammal&org=Human&db=hg19&position=OR7E156P) | T/0.991/0.019/0.3375 | T/0.991/0.017/0.5259 | T/0.999/0.24/2.88e-06 | | T/0.996/0.105/0.04742 | | A/0.992/0.029/0.06199 | | A/0.991/-0.008/0.6526 |
| rs182755327 | 13 |  |  | [OR7E156P (186964)](http://genome.ucsc.edu/cgi-bin/hgTracks?hgHubConnect.destUrl=..%2Fcgi-bin%2FhgTracks&clade=mammal&org=Human&db=hg19&position=OR7E156P) | T/0.992/0.025/0.21 | T/0.992/0.023/0.3844 | T/0.999/0.236/3.52e-06 | | T/0.996/0.102/0.05223 | | A/0.993/0.031/0.04699 | | A/0.992/-0.005/0.7645 |
| rs141318698 | 13 |  |  |  | NA | NA | G/0.989/0.08/3.97e-07 | | G/0.983/0.036/0.1108 | | NA | | NA |
| rs148342344 | 13 |  |  |  | NA | NA | G/0.989/0.08/3.72e-07 | | G/0.983/0.037/0.1085 | | NA | | NA |
| rs138607199 | 13 |  |  |  | NA | NA | A/0.989/0.08/3.69e-07 | | A/0.983/0.037/0.1081 | | NA | | NA |
| rs141455065 | 13 |  |  |  | NA | NA | T/0.992/0.087/3.36e-06 | | T/0.986/0.03/0.2476 | | NA | | NA |
| rs141008343 | 13 |  |  |  | NA | NA | A/0.989/0.08/4.06e-07 | | A/0.982/0.036/0.1124 | | NA | | NA |
| rs148788110 | 13 |  |  |  | NA | NA | C/0.99/0.086/1.77e-07 | | C/0.982/0.035/0.1204 | | NA | | NA |
| rs9599394 | 13 |  |  |  | T/0.811/-0.004/0.1776 | T/0.812/-0.008/0.05587 | T/0.951/0.034/2.86e-06 | | T/0.94/0.026/0.03463 | | T/0.168/0/0.9396 | | T/0.181/0.006/0.0443 |
| rs9599403 | 13 |  |  |  | A/0.812/-0.004/0.1949 | A/0.814/-0.008/0.07612 | A/0.934/0.03/2.6e-06 | | A/0.922/0.029/0.00684 | | A/0.166/-0.001/0.7422 | | A/0.18/0.006/0.0698 |
| rs150369165 | 13 |  |  |  | NA | NA | G/0.995/0.187/3.42e-06 | | G/0.993/0.043/0.3629 | | NA | | NA |
| rs191275341 | 13 |  | [GPC6 (108333)](http://genome.ucsc.edu/cgi-bin/hgTracks?hgHubConnect.destUrl=..%2Fcgi-bin%2FhgTracks&clade=mammal&org=Human&db=hg19&position=GPC6) | [DCT (24719)](http://genome.ucsc.edu/cgi-bin/hgTracks?hgHubConnect.destUrl=..%2Fcgi-bin%2FhgTracks&clade=mammal&org=Human&db=hg19&position=DCT) | NA | NA | C/1/0.481/2.27e-07 | | C/0.999/0.257/0.00315 | | NA | | NA |
| rs184940006 | 13 |  |  |  | NA | NA | G/1/0.577/4.69e-08 | | G/0.999/0.351/0.00047 | | NA | | NA |
| rs146346907 | 13 |  | [TNFSF13B (72439)](http://genome.ucsc.edu/cgi-bin/hgTracks?hgHubConnect.destUrl=..%2Fcgi-bin%2FhgTracks&clade=mammal&org=Human&db=hg19&position=TNFSF13B) | [MYO16 (215228)](http://genome.ucsc.edu/cgi-bin/hgTracks?hgHubConnect.destUrl=..%2Fcgi-bin%2FhgTracks&clade=mammal&org=Human&db=hg19&position=MYO16) | G/0.999/-0.029/0.5844 | G/0.999/0.028/0.6575 | G/1/0.75/3.44e-06 | | G/0.999/0.414/0.00564 | | A/0.999/-0.012/0.7953 | | A/0.999/-0.014/0.8008 |
| rs146868790 | 14 | [NRL](http://genome.ucsc.edu/cgi-bin/hgTracks?hgHubConnect.destUrl=..%2Fcgi-bin%2FhgTracks&clade=mammal&org=Human&db=hg19&position=NRL) | [PCK2 (1577)](http://genome.ucsc.edu/cgi-bin/hgTracks?hgHubConnect.destUrl=..%2Fcgi-bin%2FhgTracks&clade=mammal&org=Human&db=hg19&position=PCK2) | [DCAF11 (8989)](http://genome.ucsc.edu/cgi-bin/hgTracks?hgHubConnect.destUrl=..%2Fcgi-bin%2FhgTracks&clade=mammal&org=Human&db=hg19&position=DCAF11) | NA | NA | C/0.999/0.34/4.86e-06 | | C/0.998/0.304/0.00038 | | NA | | NA |
| rs192030704 | 14 |  | [NOVA1 (179887)](http://genome.ucsc.edu/cgi-bin/hgTracks?hgHubConnect.destUrl=..%2Fcgi-bin%2FhgTracks&clade=mammal&org=Human&db=hg19&position=NOVA1) |  | NA | NA | C/0.998/0.258/3.17e-06 | | C/0.997/0.157/0.02215 | | NA | | NA |
| rs111668083 | 14 |  | [BC148262 (133339)](http://genome.ucsc.edu/cgi-bin/hgTracks?hgHubConnect.destUrl=..%2Fcgi-bin%2FhgTracks&clade=mammal&org=Human&db=hg19&position=BC148262) |  | NA | NA | G/0.937/0.036/4.81e-06 | | G/0.926/0.036/0.00546 | | NA | | NA |
| rs116590076 | 14 |  |  |  | NA | NA | C/0.999/0.285/3.36e-06 | | C/0.997/0.195/0.0028 | | NA | | NA |
| rs190971703 | 14 |  |  |  | NA | NA | T/0.999/0.285/2.86e-06 | | T/0.997/0.195/0.00267 | | NA | | NA |
| rs115034739 | 14 |  |  |  | NA | NA | G/0.999/0.285/2.11e-06 | | G/0.997/0.196/0.00232 | | NA | | NA |
| rs74492808 | 14 |  |  |  | NA | NA | G/0.999/0.285/2.13e-06 | | G/0.997/0.196/0.00226 | | NA | | NA |
| rs114791918 | 14 |  |  |  | NA | NA | A/0.999/0.284/2.29e-06 | | A/0.997/0.199/0.00203 | | NA | | NA |
| rs78242550 | 14 |  |  |  | NA | NA | A/0.999/0.284/2.41e-06 | | A/0.997/0.201/0.00179 | | NA | | NA |
| rs116335274 | 14 |  |  |  | NA | NA | G/0.999/0.286/2.76e-06 | | G/0.997/0.202/0.00199 | | NA | | NA |
| rs146750542 | 14 |  | [NUBPL (169742)](http://genome.ucsc.edu/cgi-bin/hgTracks?hgHubConnect.destUrl=..%2Fcgi-bin%2FhgTracks&clade=mammal&org=Human&db=hg19&position=NUBPL) | [ARHGAP5-AS1 (44453)](http://genome.ucsc.edu/cgi-bin/hgTracks?hgHubConnect.destUrl=..%2Fcgi-bin%2FhgTracks&clade=mammal&org=Human&db=hg19&position=ARHGAP5-AS1) | NA | NA | A/0.998/0.214/3.1e-06 | | A/0.996/0.168/0.00189 | | NA | | NA |
| rs181881613 | 14 |  |  |  | NA | NA | T/0.999/0.363/4.49e-06 | | T/0.998/0.285/0.00136 | | NA | | NA |
| rs191739273 | 14 |  |  |  | NA | NA | G/1/0.495/3.38e-09 | | G/0.998/0.3/0.00011 | | NA | | NA |
| rs191755093 | 14 |  |  |  | NA | NA | T/1/0.5/3.14e-09 | | T/0.998/0.302/0.00012 | | NA | | NA |
| rs192522312 | 14 |  |  | [RPL10L (37748)](http://genome.ucsc.edu/cgi-bin/hgTracks?hgHubConnect.destUrl=..%2Fcgi-bin%2FhgTracks&clade=mammal&org=Human&db=hg19&position=RPL10L) | NA | NA | C/1/0.579/2.94e-09 | | C/0.998/0.348/0.00012 | | NA | | NA |
| rs185449237 | 14 |  |  | [MDGA2 (109494)](http://genome.ucsc.edu/cgi-bin/hgTracks?hgHubConnect.destUrl=..%2Fcgi-bin%2FhgTracks&clade=mammal&org=Human&db=hg19&position=MDGA2) | NA | NA | A/1/0.632/3.8e-09 | | A/0.999/0.383/0.00012 | | NA | | NA |
| rs192383543 | 14 |  |  | [MDGA2 (107965)](http://genome.ucsc.edu/cgi-bin/hgTracks?hgHubConnect.destUrl=..%2Fcgi-bin%2FhgTracks&clade=mammal&org=Human&db=hg19&position=MDGA2) | NA | NA | C/1/0.633/3.81e-09 | | C/0.999/0.383/0.00012 | | NA | | NA |
| rs188669819 | 14 | [MDGA2](http://genome.ucsc.edu/cgi-bin/hgTracks?hgHubConnect.destUrl=..%2Fcgi-bin%2FhgTracks&clade=mammal&org=Human&db=hg19&position=MDGA2) |  |  | NA | NA | T/1/0.799/7.57e-08 | | T/0.999/0.505/0.00024 | | NA | | NA |
| rs188838161 | 14 |  | [FRMD6 (90664)](http://genome.ucsc.edu/cgi-bin/hgTracks?hgHubConnect.destUrl=..%2Fcgi-bin%2FhgTracks&clade=mammal&org=Human&db=hg19&position=FRMD6) | [GNG2 (25843)](http://genome.ucsc.edu/cgi-bin/hgTracks?hgHubConnect.destUrl=..%2Fcgi-bin%2FhgTracks&clade=mammal&org=Human&db=hg19&position=GNG2) | NA | NA | T/0.999/0.527/1.94e-06 | | T/0.999/0.318/0.00308 | | NA | | NA |
| rs183755958 | 14 |  | [FRMD6 (110034)](http://genome.ucsc.edu/cgi-bin/hgTracks?hgHubConnect.destUrl=..%2Fcgi-bin%2FhgTracks&clade=mammal&org=Human&db=hg19&position=FRMD6) | [GNG2 (6473)](http://genome.ucsc.edu/cgi-bin/hgTracks?hgHubConnect.destUrl=..%2Fcgi-bin%2FhgTracks&clade=mammal&org=Human&db=hg19&position=GNG2) | NA | NA | T/1/0.551/7.24e-07 | | T/0.999/0.327/0.00234 | | NA | | NA |
| rs192180133 | 14 | [GNG2](http://genome.ucsc.edu/cgi-bin/hgTracks?hgHubConnect.destUrl=..%2Fcgi-bin%2FhgTracks&clade=mammal&org=Human&db=hg19&position=GNG2) | [FRMD6 (134802)](http://genome.ucsc.edu/cgi-bin/hgTracks?hgHubConnect.destUrl=..%2Fcgi-bin%2FhgTracks&clade=mammal&org=Human&db=hg19&position=FRMD6) | [C14orf166 (123981)](http://genome.ucsc.edu/cgi-bin/hgTracks?hgHubConnect.destUrl=..%2Fcgi-bin%2FhgTracks&clade=mammal&org=Human&db=hg19&position=C14orf166) | NA | NA | C/0.999/0.519/2.01e-06 | | C/0.999/0.309/0.00356 | | NA | | NA |
| rs192692653 | 14 | [GNG2](http://genome.ucsc.edu/cgi-bin/hgTracks?hgHubConnect.destUrl=..%2Fcgi-bin%2FhgTracks&clade=mammal&org=Human&db=hg19&position=GNG2) | [FRMD6 (187042)](http://genome.ucsc.edu/cgi-bin/hgTracks?hgHubConnect.destUrl=..%2Fcgi-bin%2FhgTracks&clade=mammal&org=Human&db=hg19&position=FRMD6) | [C14orf166 (71741)](http://genome.ucsc.edu/cgi-bin/hgTracks?hgHubConnect.destUrl=..%2Fcgi-bin%2FhgTracks&clade=mammal&org=Human&db=hg19&position=C14orf166) | NA | NA | T/1/0.571/4.03e-07 | | T/0.999/0.324/0.0029 | | NA | | T/0.002/0.064/0.2614 |
| rs79534246 | 14 | [FERMT2](http://genome.ucsc.edu/cgi-bin/hgTracks?hgHubConnect.destUrl=..%2Fcgi-bin%2FhgTracks&clade=mammal&org=Human&db=hg19&position=FERMT2) | [GNPNAT1 (128056)](http://genome.ucsc.edu/cgi-bin/hgTracks?hgHubConnect.destUrl=..%2Fcgi-bin%2FhgTracks&clade=mammal&org=Human&db=hg19&position=GNPNAT1) | [DDHD1 (117015)](http://genome.ucsc.edu/cgi-bin/hgTracks?hgHubConnect.destUrl=..%2Fcgi-bin%2FhgTracks&clade=mammal&org=Human&db=hg19&position=DDHD1) | A/0.98/0/0.9721 | A/0.982/0.023/0.2375 | A/0.996/0.17/4.52e-06 | | A/0.993/0.109/0.02087 | | A/0.017/-0.019/0.09364 | | A/0.017/-0.006/0.6668 |
| rs73276024 | 14 |  | [LINC00520 (93529)](http://genome.ucsc.edu/cgi-bin/hgTracks?hgHubConnect.destUrl=..%2Fcgi-bin%2FhgTracks&clade=mammal&org=Human&db=hg19&position=LINC00520) |  | A/0.942/0.001/0.8645 | A/0.939/0.001/0.8532 | A/0.825/-0.02/3.43e-06 | | A/0.856/-0.018/0.03049 | | A/0.12/0.009/0.00382 | | A/0.066/0.002/0.7592 |
| rs181293434 | 14 |  | [PRKCH (146)](http://genome.ucsc.edu/cgi-bin/hgTracks?hgHubConnect.destUrl=..%2Fcgi-bin%2FhgTracks&clade=mammal&org=Human&db=hg19&position=PRKCH) | [FLJ22447 (19413)](http://genome.ucsc.edu/cgi-bin/hgTracks?hgHubConnect.destUrl=..%2Fcgi-bin%2FhgTracks&clade=mammal&org=Human&db=hg19&position=FLJ22447) | NA | NA | T/0.999/0.282/2.89e-06 | | T/0.997/0.144/0.01403 | | NA | | NA |
| rs186116546 | 14 | [FUT8](http://genome.ucsc.edu/cgi-bin/hgTracks?hgHubConnect.destUrl=..%2Fcgi-bin%2FhgTracks&clade=mammal&org=Human&db=hg19&position=FUT8) | [Mir_625 (48272)](http://genome.ucsc.edu/cgi-bin/hgTracks?hgHubConnect.destUrl=..%2Fcgi-bin%2FhgTracks&clade=mammal&org=Human&db=hg19&position=Mir_625) |  | NA | NA | T/1/0.559/2.6e-06 | | T/0.999/0.317/0.00428 | | NA | | NA |
| rs182491339 | 14 | [FUT8](http://genome.ucsc.edu/cgi-bin/hgTracks?hgHubConnect.destUrl=..%2Fcgi-bin%2FhgTracks&clade=mammal&org=Human&db=hg19&position=FUT8) | [Mir_625 (112025)](http://genome.ucsc.edu/cgi-bin/hgTracks?hgHubConnect.destUrl=..%2Fcgi-bin%2FhgTracks&clade=mammal&org=Human&db=hg19&position=Mir_625) |  | NA | NA | G/1/0.581/2.4e-06 | | G/0.999/0.333/0.00378 | | NA | | NA |
| rs117911949 | 14 | [SIPA1L1](http://genome.ucsc.edu/cgi-bin/hgTracks?hgHubConnect.destUrl=..%2Fcgi-bin%2FhgTracks&clade=mammal&org=Human&db=hg19&position=SIPA1L1) | [LOC145474 (60369)](http://genome.ucsc.edu/cgi-bin/hgTracks?hgHubConnect.destUrl=..%2Fcgi-bin%2FhgTracks&clade=mammal&org=Human&db=hg19&position=LOC145474) |  | A/0.995/0.007/0.743 | A/0.994/-0.042/0.1082 | A/0.999/0.42/3.95e-06 | | A/0.998/0.19/0.02368 | | A/0.005/-0.027/0.143 | | A/0.006/0.017/0.3937 |
| rs181902743 | 14 | [CEP128](http://genome.ucsc.edu/cgi-bin/hgTracks?hgHubConnect.destUrl=..%2Fcgi-bin%2FhgTracks&clade=mammal&org=Human&db=hg19&position=CEP128) |  |  | NA | NA | G/1/0.503/3.27e-06 | | G/0.999/0.297/0.0032 | | NA | | NA |
| rs140660435 | 14 |  |  |  | NA | NA | C/1/0.51/1.52e-06 | | C/0.999/0.365/0.00058 | | NA | | NA |
| rs138048027 | 14 |  |  |  | NA | NA | A/0.999/0.472/3.87e-06 | | A/0.999/0.324/0.00198 | | NA | | NA |
| rs185677526 | 14 |  |  | [BX248253 (49415)](http://genome.ucsc.edu/cgi-bin/hgTracks?hgHubConnect.destUrl=..%2Fcgi-bin%2FhgTracks&clade=mammal&org=Human&db=hg19&position=BX248253) | NA | NA | T/0.998/0.183/7.34e-07 | | T/0.994/0.097/0.02128 | | NA | | NA |
| rs191926782 | 14 |  |  | [BX248253 (35305)](http://genome.ucsc.edu/cgi-bin/hgTracks?hgHubConnect.destUrl=..%2Fcgi-bin%2FhgTracks&clade=mammal&org=Human&db=hg19&position=BX248253) | NA | NA | A/0.998/0.166/1.19e-06 | | A/0.994/0.092/0.01911 | | NA | | NA |
| rs192253363 | 14 |  |  | [BX248253 (18076)](http://genome.ucsc.edu/cgi-bin/hgTracks?hgHubConnect.destUrl=..%2Fcgi-bin%2FhgTracks&clade=mammal&org=Human&db=hg19&position=BX248253) | NA | NA | G/0.998/0.161/1.79e-06 | | G/0.994/0.092/0.01863 | | NA | | NA |
| rs112458284 | 14 | [PPP4R4](http://genome.ucsc.edu/cgi-bin/hgTracks?hgHubConnect.destUrl=..%2Fcgi-bin%2FhgTracks&clade=mammal&org=Human&db=hg19&position=PPP4R4) | [IFI27L2 (76774)](http://genome.ucsc.edu/cgi-bin/hgTracks?hgHubConnect.destUrl=..%2Fcgi-bin%2FhgTracks&clade=mammal&org=Human&db=hg19&position=IFI27L2) | [SERPINA10 (76918)](http://genome.ucsc.edu/cgi-bin/hgTracks?hgHubConnect.destUrl=..%2Fcgi-bin%2FhgTracks&clade=mammal&org=Human&db=hg19&position=SERPINA10) | C/0.964/0.026/0.0019 | C/0.96/0.016/0.1269 | C/0.993/0.109/1.33e-06 | | C/0.988/0.092/0.00214 | | T/0.965/0.041/9.12e-11 | | T/0.959/0.023/0.00111 |
| rs2402444 | 14 |  | [SERPINA10 (6076)](http://genome.ucsc.edu/cgi-bin/hgTracks?hgHubConnect.destUrl=..%2Fcgi-bin%2FhgTracks&clade=mammal&org=Human&db=hg19&position=SERPINA10) | [SERPINA6 (4912)](http://genome.ucsc.edu/cgi-bin/hgTracks?hgHubConnect.destUrl=..%2Fcgi-bin%2FhgTracks&clade=mammal&org=Human&db=hg19&position=SERPINA6) | G/0.803/0.005/0.1278 | G/0.797/0.002/0.5827 | G/0.907/0.028/1.97e-06 | | G/0.892/0.026/0.00969 | | T/0.819/0.011/1.14e-05 | | T/0.795/0.004/0.1843 |
| rs142936968 | 15 | [OCA2](http://genome.ucsc.edu/cgi-bin/hgTracks?hgHubConnect.destUrl=..%2Fcgi-bin%2FhgTracks&clade=mammal&org=Human&db=hg19&position=OCA2) |  | [HERC2 (131256)](http://genome.ucsc.edu/cgi-bin/hgTracks?hgHubConnect.destUrl=..%2Fcgi-bin%2FhgTracks&clade=mammal&org=Human&db=hg19&position=HERC2) | T/0.997/0.075/0.00863 | T/0.997/0.055/0.1023 | T/0.993/0.111/3.65e-06 | | T/0.989/0.089/0.00635 | | T/0.005/-0.073/5.27e-06 | | T/0.003/-0.041/0.08728 |
| rs187260613 | 15 |  | [BC039545 (11326)](http://genome.ucsc.edu/cgi-bin/hgTracks?hgHubConnect.destUrl=..%2Fcgi-bin%2FhgTracks&clade=mammal&org=Human&db=hg19&position=BC039545) | [BC037952 (87787)](http://genome.ucsc.edu/cgi-bin/hgTracks?hgHubConnect.destUrl=..%2Fcgi-bin%2FhgTracks&clade=mammal&org=Human&db=hg19&position=BC037952) | NA | NA | A/0.996/0.172/2.74e-06 | | A/0.993/0.116/0.00914 | | NA | | NA |
| rs180709182 | 15 | [SEMA6D](http://genome.ucsc.edu/cgi-bin/hgTracks?hgHubConnect.destUrl=..%2Fcgi-bin%2FhgTracks&clade=mammal&org=Human&db=hg19&position=SEMA6D) |  |  | NA | NA | C/1/0.602/4.74e-07 | | C/0.999/0.35/0.00162 | | NA | | NA |
| rs192229215 | 15 | [SEMA6D](http://genome.ucsc.edu/cgi-bin/hgTracks?hgHubConnect.destUrl=..%2Fcgi-bin%2FhgTracks&clade=mammal&org=Human&db=hg19&position=SEMA6D) |  |  | NA | NA | A/1/0.634/1.05e-06 | | A/0.999/0.376/0.00172 | | NA | | NA |
| rs114831632 | 15 |  | [POLR2M (75617)](http://genome.ucsc.edu/cgi-bin/hgTracks?hgHubConnect.destUrl=..%2Fcgi-bin%2FhgTracks&clade=mammal&org=Human&db=hg19&position=POLR2M) | [ALDH1A2 (160249)](http://genome.ucsc.edu/cgi-bin/hgTracks?hgHubConnect.destUrl=..%2Fcgi-bin%2FhgTracks&clade=mammal&org=Human&db=hg19&position=ALDH1A2) | NA | NA | A/0.995/0.122/1.07e-06 | | A/0.988/0.048/0.09849 | | NA | | NA |
| rs149074315 | 15 |  | [POLR2M (79134)](http://genome.ucsc.edu/cgi-bin/hgTracks?hgHubConnect.destUrl=..%2Fcgi-bin%2FhgTracks&clade=mammal&org=Human&db=hg19&position=POLR2M) | [ALDH1A2 (156732)](http://genome.ucsc.edu/cgi-bin/hgTracks?hgHubConnect.destUrl=..%2Fcgi-bin%2FhgTracks&clade=mammal&org=Human&db=hg19&position=ALDH1A2) | NA | NA | C/0.996/0.14/4.27e-07 | | C/0.99/0.065/0.0418 | | NA | | NA |
| rs140526800 | 15 |  | [POLR2M (79580)](http://genome.ucsc.edu/cgi-bin/hgTracks?hgHubConnect.destUrl=..%2Fcgi-bin%2FhgTracks&clade=mammal&org=Human&db=hg19&position=POLR2M) | [ALDH1A2 (156286)](http://genome.ucsc.edu/cgi-bin/hgTracks?hgHubConnect.destUrl=..%2Fcgi-bin%2FhgTracks&clade=mammal&org=Human&db=hg19&position=ALDH1A2) | NA | NA | T/0.995/0.121/1.14e-06 | | T/0.988/0.049/0.09506 | | NA | | NA |
| rs145330511 | 15 |  | [POLR2M (90837)](http://genome.ucsc.edu/cgi-bin/hgTracks?hgHubConnect.destUrl=..%2Fcgi-bin%2FhgTracks&clade=mammal&org=Human&db=hg19&position=POLR2M) | [ALDH1A2 (145029)](http://genome.ucsc.edu/cgi-bin/hgTracks?hgHubConnect.destUrl=..%2Fcgi-bin%2FhgTracks&clade=mammal&org=Human&db=hg19&position=ALDH1A2) | NA | NA | T/0.995/0.127/1.34e-06 | | T/0.988/0.051/0.1071 | | NA | | NA |
| rs140949321 | 15 |  | [POLR2M (92109)](http://genome.ucsc.edu/cgi-bin/hgTracks?hgHubConnect.destUrl=..%2Fcgi-bin%2FhgTracks&clade=mammal&org=Human&db=hg19&position=POLR2M) | [ALDH1A2 (143757)](http://genome.ucsc.edu/cgi-bin/hgTracks?hgHubConnect.destUrl=..%2Fcgi-bin%2FhgTracks&clade=mammal&org=Human&db=hg19&position=ALDH1A2) | NA | NA | A/0.994/0.127/1.38e-06 | | A/0.988/0.05/0.1138 | | NA | | NA |
| rs148246786 | 15 |  | [POLR2M (92228)](http://genome.ucsc.edu/cgi-bin/hgTracks?hgHubConnect.destUrl=..%2Fcgi-bin%2FhgTracks&clade=mammal&org=Human&db=hg19&position=POLR2M) | [ALDH1A2 (143638)](http://genome.ucsc.edu/cgi-bin/hgTracks?hgHubConnect.destUrl=..%2Fcgi-bin%2FhgTracks&clade=mammal&org=Human&db=hg19&position=ALDH1A2) | NA | NA | C/0.974/0.059/5.16e-07 | | C/0.966/0.064/0.00058 | | NA | | NA |
| rs7164035 | 15 |  | [POLR2M (92486)](http://genome.ucsc.edu/cgi-bin/hgTracks?hgHubConnect.destUrl=..%2Fcgi-bin%2FhgTracks&clade=mammal&org=Human&db=hg19&position=POLR2M) | [ALDH1A2 (143380)](http://genome.ucsc.edu/cgi-bin/hgTracks?hgHubConnect.destUrl=..%2Fcgi-bin%2FhgTracks&clade=mammal&org=Human&db=hg19&position=ALDH1A2) | NA | NA | A/0.973/0.058/6.55e-07 | | A/0.965/0.065/0.00056 | | NA | | NA |
| rs146071159 | 15 |  | [POLR2M (94478)](http://genome.ucsc.edu/cgi-bin/hgTracks?hgHubConnect.destUrl=..%2Fcgi-bin%2FhgTracks&clade=mammal&org=Human&db=hg19&position=POLR2M) | [ALDH1A2 (141388)](http://genome.ucsc.edu/cgi-bin/hgTracks?hgHubConnect.destUrl=..%2Fcgi-bin%2FhgTracks&clade=mammal&org=Human&db=hg19&position=ALDH1A2) | NA | NA | C/0.994/0.12/3.6e-06 | | C/0.988/0.046/0.1434 | | NA | | NA |
| rs113870173 | 15 |  | [POLR2M (95044)](http://genome.ucsc.edu/cgi-bin/hgTracks?hgHubConnect.destUrl=..%2Fcgi-bin%2FhgTracks&clade=mammal&org=Human&db=hg19&position=POLR2M) | [ALDH1A2 (140822)](http://genome.ucsc.edu/cgi-bin/hgTracks?hgHubConnect.destUrl=..%2Fcgi-bin%2FhgTracks&clade=mammal&org=Human&db=hg19&position=ALDH1A2) | NA | NA | A/0.994/0.119/3.52e-06 | | A/0.987/0.042/0.1745 | | NA | | NA |
| rs115942227 | 15 |  | [POLR2M (95278)](http://genome.ucsc.edu/cgi-bin/hgTracks?hgHubConnect.destUrl=..%2Fcgi-bin%2FhgTracks&clade=mammal&org=Human&db=hg19&position=POLR2M) | [ALDH1A2 (140588)](http://genome.ucsc.edu/cgi-bin/hgTracks?hgHubConnect.destUrl=..%2Fcgi-bin%2FhgTracks&clade=mammal&org=Human&db=hg19&position=ALDH1A2) | NA | NA | A/0.992/0.114/4.16e-07 | | A/0.984/0.047/0.08409 | | NA | | NA |
| rs17486278 | 15 | [CHRNA5](http://genome.ucsc.edu/cgi-bin/hgTracks?hgHubConnect.destUrl=..%2Fcgi-bin%2FhgTracks&clade=mammal&org=Human&db=hg19&position=CHRNA5) | [PSMA4 (25919)](http://genome.ucsc.edu/cgi-bin/hgTracks?hgHubConnect.destUrl=..%2Fcgi-bin%2FhgTracks&clade=mammal&org=Human&db=hg19&position=PSMA4) | [CHRNA3 (17912)](http://genome.ucsc.edu/cgi-bin/hgTracks?hgHubConnect.destUrl=..%2Fcgi-bin%2FhgTracks&clade=mammal&org=Human&db=hg19&position=CHRNA3) | C/0.632/0.017/1.24e-09 | C/0.608/0.013/0.00032 | C/0.709/0.016/3.14e-06 | | C/0.676/0.011/0.08466 | | A/0.65/0.017/4.48e-20 | | A/0.601/0.012/6.7e-07 |
| rs191299947 | 15 | [ARNT2](http://genome.ucsc.edu/cgi-bin/hgTracks?hgHubConnect.destUrl=..%2Fcgi-bin%2FhgTracks&clade=mammal&org=Human&db=hg19&position=ARNT2) | [AB240015 (218036)](http://genome.ucsc.edu/cgi-bin/hgTracks?hgHubConnect.destUrl=..%2Fcgi-bin%2FhgTracks&clade=mammal&org=Human&db=hg19&position=AB240015) | [FAM108C1 (110094)](http://genome.ucsc.edu/cgi-bin/hgTracks?hgHubConnect.destUrl=..%2Fcgi-bin%2FhgTracks&clade=mammal&org=Human&db=hg19&position=FAM108C1) | NA | NA | A/0.994/0.107/2.29e-06 | | A/0.988/0.037/0.1921 | | NA | | NA |
| rs147415896 | 15 | [SLCO3A1](http://genome.ucsc.edu/cgi-bin/hgTracks?hgHubConnect.destUrl=..%2Fcgi-bin%2FhgTracks&clade=mammal&org=Human&db=hg19&position=SLCO3A1) |  |  | NA | NA | C/0.994/0.137/4.88e-06 | | C/0.99/0.111/0.00366 | | NA | | NA |
| rs140011970 | 15 | [SLCO3A1](http://genome.ucsc.edu/cgi-bin/hgTracks?hgHubConnect.destUrl=..%2Fcgi-bin%2FhgTracks&clade=mammal&org=Human&db=hg19&position=SLCO3A1) |  |  | NA | NA | G/0.993/0.117/4.09e-06 | | G/0.99/0.114/0.00151 | | NA | | NA |
| rs184252378 | 16 |  |  |  | NA | NA | G/1/1.006/1.07e-06 | | G/0.999/0.501/0.00898 | | NA | | NA |
| rs186108482 | 16 |  |  | [ERCC4 (154822)](http://genome.ucsc.edu/cgi-bin/hgTracks?hgHubConnect.destUrl=..%2Fcgi-bin%2FhgTracks&clade=mammal&org=Human&db=hg19&position=ERCC4) | NA | NA | G/1/0.811/6.17e-08 | | G/0.999/0.461/0.00087 | | NA | | NA |
| rs191732819 | 16 |  |  | [ERCC4 (148786)](http://genome.ucsc.edu/cgi-bin/hgTracks?hgHubConnect.destUrl=..%2Fcgi-bin%2FhgTracks&clade=mammal&org=Human&db=hg19&position=ERCC4) | NA | NA | A/1/1.028/6.03e-08 | | A/0.999/0.585/0.00085 | | NA | | NA |
| rs142916547 | 16 |  | [ZKSCAN2 (231587)](http://genome.ucsc.edu/cgi-bin/hgTracks?hgHubConnect.destUrl=..%2Fcgi-bin%2FhgTracks&clade=mammal&org=Human&db=hg19&position=ZKSCAN2) |  | NA | NA | G/0.999/0.422/8.47e-07 | | G/0.998/0.21/0.00898 | | NA | | NA |
| rs148319405 | 16 |  | [CYLD (72972)](http://genome.ucsc.edu/cgi-bin/hgTracks?hgHubConnect.destUrl=..%2Fcgi-bin%2FhgTracks&clade=mammal&org=Human&db=hg19&position=CYLD) |  | NA | NA | T/0.999/0.53/1.61e-07 | | T/0.998/0.302/0.00215 | | NA | | NA |
| rs147153117 | 16 |  | [CYLD (249676)](http://genome.ucsc.edu/cgi-bin/hgTracks?hgHubConnect.destUrl=..%2Fcgi-bin%2FhgTracks&clade=mammal&org=Human&db=hg19&position=CYLD) | [SALL1 (108357)](http://genome.ucsc.edu/cgi-bin/hgTracks?hgHubConnect.destUrl=..%2Fcgi-bin%2FhgTracks&clade=mammal&org=Human&db=hg19&position=SALL1) | NA | NA | T/0.999/0.342/3.02e-06 | | T/0.997/0.154/0.0292 | | NA | | NA |
| rs150896862 | 16 |  |  |  | NA | NA | T/0.999/0.381/1.08e-07 | | T/0.998/0.275/0.00028 | | NA | | NA |
| rs188330159 | 16 |  |  |  | NA | NA | G/0.999/0.379/1.03e-07 | | G/0.998/0.274/0.00026 | | NA | | NA |
| rs114607539 | 16 |  |  |  | NA | NA | A/0.999/0.379/1.06e-07 | | A/0.998/0.271/0.00028 | | NA | | NA |
| rs146680296 | 16 |  |  |  | NA | NA | G/0.999/0.379/1.07e-07 | | G/0.998/0.27/0.00029 | | NA | | NA |
| rs184181234 | 16 |  |  |  | NA | NA | C/0.999/0.377/1.15e-07 | | C/0.998/0.267/3e-04 | | NA | | NA |
| rs116768843 | 16 |  |  |  | A/0.998/0.035/0.4156 | A/0.998/0.09/0.1043 | A/0.999/0.371/1.82e-07 | | A/0.997/0.26/0.00045 | | A/0.002/-0.091/0.00112 | | A/0.002/-0.082/0.01844 |
| rs143132326 | 16 |  |  |  | NA | NA | G/0.999/0.377/1.16e-07 | | G/0.998/0.267/3e-04 | | NA | | NA |
| rs74681184 | 16 |  |  |  | NA | NA | T/0.999/0.377/1.2e-07 | | T/0.998/0.267/0.00031 | | NA | | NA |
| rs143661751 | 16 |  |  |  | G/0.996/0.006/0.7816 | G/0.996/-0.016/0.6025 | G/1/0.464/1.59e-06 | | G/0.999/0.291/0.0035 | | A/0.996/0.027/0.2286 | | A/0.996/-0.016/0.5885 |
| rs181167431 | 16 |  |  |  | A/0.995/0.014/0.5065 | A/0.994/-0.01/0.7215 | A/1/0.541/1.49e-07 | | A/0.999/0.267/0.00552 | | NA | | A/0.006/0.01/0.721 |
| rs9935937 | 16 | [WDR59](http://genome.ucsc.edu/cgi-bin/hgTracks?hgHubConnect.destUrl=..%2Fcgi-bin%2FhgTracks&clade=mammal&org=Human&db=hg19&position=WDR59) | [FA2H (235481)](http://genome.ucsc.edu/cgi-bin/hgTracks?hgHubConnect.destUrl=..%2Fcgi-bin%2FhgTracks&clade=mammal&org=Human&db=hg19&position=FA2H) | [ZNRF1 (46429)](http://genome.ucsc.edu/cgi-bin/hgTracks?hgHubConnect.destUrl=..%2Fcgi-bin%2FhgTracks&clade=mammal&org=Human&db=hg19&position=ZNRF1) | C/0.948/-0.001/0.9066 | C/0.948/-0.01/0.2467 | C/0.911/-0.027/3.99e-06 | | C/0.931/-0.035/0.00537 | | T/0.933/-0.012/0.00221 | | T/0.948/-0.009/0.09994 |
| rs58423913 | 16 | [WWOX](http://genome.ucsc.edu/cgi-bin/hgTracks?hgHubConnect.destUrl=..%2Fcgi-bin%2FhgTracks&clade=mammal&org=Human&db=hg19&position=WWOX) |  |  | C/0.83/0.006/0.09476 | C/0.826/0.008/0.08537 | C/0.957/0.038/4.25e-06 | | C/0.946/0.035/0.00863 | | T/0.834/0.006/0.01339 | | T/0.817/0.001/0.6753 |
| rs2047925 | 16 | [WWOX](http://genome.ucsc.edu/cgi-bin/hgTracks?hgHubConnect.destUrl=..%2Fcgi-bin%2FhgTracks&clade=mammal&org=Human&db=hg19&position=WWOX) |  |  | C/0.831/0.005/0.1332 | C/0.827/0.008/0.1003 | C/0.958/0.042/9.59e-07 | | C/0.944/0.032/0.02227 | | C/0.166/-0.006/0.01909 | | C/0.181/-0.001/0.8076 |
| rs4888873 | 16 | [WWOX](http://genome.ucsc.edu/cgi-bin/hgTracks?hgHubConnect.destUrl=..%2Fcgi-bin%2FhgTracks&clade=mammal&org=Human&db=hg19&position=WWOX) |  |  | C/0.832/0.006/0.1242 | C/0.827/0.008/0.1072 | C/0.957/0.04/3.46e-06 | | C/0.945/0.032/0.02067 | | T/0.835/0.006/0.01993 | | T/0.819/0.001/0.8117 |
| rs62038064 | 16 | [WWOX](http://genome.ucsc.edu/cgi-bin/hgTracks?hgHubConnect.destUrl=..%2Fcgi-bin%2FhgTracks&clade=mammal&org=Human&db=hg19&position=WWOX) |  |  | G/0.831/0.005/0.1377 | G/0.826/0.007/0.1116 | G/0.956/0.042/1.04e-06 | | G/0.943/0.031/0.02631 | | C/0.834/0.006/0.02046 | | C/0.818/0.001/0.8431 |
| rs59626546 | 16 | [WWOX](http://genome.ucsc.edu/cgi-bin/hgTracks?hgHubConnect.destUrl=..%2Fcgi-bin%2FhgTracks&clade=mammal&org=Human&db=hg19&position=WWOX) |  |  | G/0.831/0.005/0.1453 | G/0.826/0.007/0.1229 | G/0.954/0.04/2.66e-06 | | G/0.942/0.03/0.0281 | | A/0.834/0.006/0.02232 | | A/0.818/0/0.875 |
| rs55760002 | 16 | [WWOX](http://genome.ucsc.edu/cgi-bin/hgTracks?hgHubConnect.destUrl=..%2Fcgi-bin%2FhgTracks&clade=mammal&org=Human&db=hg19&position=WWOX) |  |  | G/0.831/0.005/0.1469 | G/0.826/0.007/0.1252 | G/0.955/0.04/2.76e-06 | | G/0.943/0.03/0.0279 | | C/0.834/0.006/0.02382 | | C/0.818/0/0.875 |
| rs1541692 | 16 |  |  |  | T/0.433/-0.002/0.5008 | T/0.433/-0.005/0.1806 | C/0.327/-0.016/2.65e-06 | | C/0.327/-0.007/0.2461 | | T/0.537/-0.004/0.03398 | | T/0.503/0.001/0.6922 |
| rs181710595 | 16 | [CDH13](http://genome.ucsc.edu/cgi-bin/hgTracks?hgHubConnect.destUrl=..%2Fcgi-bin%2FhgTracks&clade=mammal&org=Human&db=hg19&position=CDH13) |  |  | NA | NA | G/0.999/0.433/9.89e-07 | | G/0.998/0.172/0.03757 | | NA | | NA |
| rs181225015 | 16 | [DNAAF1](http://genome.ucsc.edu/cgi-bin/hgTracks?hgHubConnect.destUrl=..%2Fcgi-bin%2FhgTracks&clade=mammal&org=Human&db=hg19&position=DNAAF1) | [HSDL1 (25578)](http://genome.ucsc.edu/cgi-bin/hgTracks?hgHubConnect.destUrl=..%2Fcgi-bin%2FhgTracks&clade=mammal&org=Human&db=hg19&position=HSDL1) | [TAF1C (7074)](http://genome.ucsc.edu/cgi-bin/hgTracks?hgHubConnect.destUrl=..%2Fcgi-bin%2FhgTracks&clade=mammal&org=Human&db=hg19&position=TAF1C) | NA | NA | T/0.999/0.347/4.41e-06 | | T/0.998/0.165/0.0195 | | NA | | T/0/-0.133/0.4592 |
| rs186788172 | 17 |  | [TUSC5 (34908)](http://genome.ucsc.edu/cgi-bin/hgTracks?hgHubConnect.destUrl=..%2Fcgi-bin%2FhgTracks&clade=mammal&org=Human&db=hg19&position=TUSC5) | [YWHAE (8644)](http://genome.ucsc.edu/cgi-bin/hgTracks?hgHubConnect.destUrl=..%2Fcgi-bin%2FhgTracks&clade=mammal&org=Human&db=hg19&position=YWHAE) | C/0.997/0.014/0.5952 | C/0.997/0.024/0.5048 | C/0.999/0.321/2.06e-06 | | C/0.998/0.22/0.0031 | | T/0.997/0.033/0.1154 | | T/0.997/0.019/0.4431 |
| rs147068743 | 17 |  | [CRK (3914)](http://genome.ucsc.edu/cgi-bin/hgTracks?hgHubConnect.destUrl=..%2Fcgi-bin%2FhgTracks&clade=mammal&org=Human&db=hg19&position=CRK) | [MYO1C (4004)](http://genome.ucsc.edu/cgi-bin/hgTracks?hgHubConnect.destUrl=..%2Fcgi-bin%2FhgTracks&clade=mammal&org=Human&db=hg19&position=MYO1C) | A/0.997/0.004/0.9124 | A/0.998/0.024/0.6412 | A/1/0.637/4.63e-07 | | A/0.999/0.32/0.00668 | | NA | | A/0.002/-0.018/0.6495 |
| rs17607 | 17 | [CD68](http://genome.ucsc.edu/cgi-bin/hgTracks?hgHubConnect.destUrl=..%2Fcgi-bin%2FhgTracks&clade=mammal&org=Human&db=hg19&position=CD68) | [EIF4A1 (2458)](http://genome.ucsc.edu/cgi-bin/hgTracks?hgHubConnect.destUrl=..%2Fcgi-bin%2FhgTracks&clade=mammal&org=Human&db=hg19&position=EIF4A1) | [MPDU1 (2182)](http://genome.ucsc.edu/cgi-bin/hgTracks?hgHubConnect.destUrl=..%2Fcgi-bin%2FhgTracks&clade=mammal&org=Human&db=hg19&position=MPDU1) | G/0.047/-0.001/0.8214 | G/0.047/-0.003/0.6754 | A/0.993/0.11/4e-06 | | A/0.989/0.09/0.00573 | | A/0.674/-0.006/0.2799 | | A/0.586/-0.004/0.5553 |
| rs144908958 | 17 | [ACCN1](http://genome.ucsc.edu/cgi-bin/hgTracks?hgHubConnect.destUrl=..%2Fcgi-bin%2FhgTracks&clade=mammal&org=Human&db=hg19&position=ACCN1) | [SPACA3 (207522)](http://genome.ucsc.edu/cgi-bin/hgTracks?hgHubConnect.destUrl=..%2Fcgi-bin%2FhgTracks&clade=mammal&org=Human&db=hg19&position=SPACA3) |  | A/0.998/-0.029/0.5039 | A/0.998/0.022/0.7702 | A/1/0.616/4.63e-06 | | A/0.999/0.386/0.00206 | | A/0.002/-0.003/0.9284 | | A/0.002/0.024/0.6284 |
| rs76839023 | 17 |  |  |  | G/0.902/0.002/0.6461 | G/0.902/0.003/0.6453 | G/0.975/0.057/3.89e-06 | | G/0.964/0.014/0.4383 | | A/0.91/0.005/0.12 | | A/0.904/0/0.9755 |
| rs146255527 | 17 | [PPM1E](http://genome.ucsc.edu/cgi-bin/hgTracks?hgHubConnect.destUrl=..%2Fcgi-bin%2FhgTracks&clade=mammal&org=Human&db=hg19&position=PPM1E) | [RAD51C (138876)](http://genome.ucsc.edu/cgi-bin/hgTracks?hgHubConnect.destUrl=..%2Fcgi-bin%2FhgTracks&clade=mammal&org=Human&db=hg19&position=RAD51C) | [TRIM37 (109431)](http://genome.ucsc.edu/cgi-bin/hgTracks?hgHubConnect.destUrl=..%2Fcgi-bin%2FhgTracks&clade=mammal&org=Human&db=hg19&position=TRIM37) | NA | NA | T/0.997/0.233/1.23e-06 | | T/0.995/0.148/0.01129 | | NA | | NA |
| rs138387343 | 17 | [PPM1E](http://genome.ucsc.edu/cgi-bin/hgTracks?hgHubConnect.destUrl=..%2Fcgi-bin%2FhgTracks&clade=mammal&org=Human&db=hg19&position=PPM1E) |  | [TRIM37 (14927)](http://genome.ucsc.edu/cgi-bin/hgTracks?hgHubConnect.destUrl=..%2Fcgi-bin%2FhgTracks&clade=mammal&org=Human&db=hg19&position=TRIM37) | NA | NA | C/0.997/0.232/1.26e-06 | | C/0.995/0.146/0.01196 | | NA | | NA |
| rs186171056 | 17 |  | [TBX4 (43618)](http://genome.ucsc.edu/cgi-bin/hgTracks?hgHubConnect.destUrl=..%2Fcgi-bin%2FhgTracks&clade=mammal&org=Human&db=hg19&position=TBX4) | [NACA2 (61704)](http://genome.ucsc.edu/cgi-bin/hgTracks?hgHubConnect.destUrl=..%2Fcgi-bin%2FhgTracks&clade=mammal&org=Human&db=hg19&position=NACA2) | NA | NA | T/1/0.349/1.26e-06 | | T/0.998/0.228/0.00193 | | NA | | NA |
| rs113936974 | 17 |  | [LINC00469 (61954)](http://genome.ucsc.edu/cgi-bin/hgTracks?hgHubConnect.destUrl=..%2Fcgi-bin%2FhgTracks&clade=mammal&org=Human&db=hg19&position=LINC00469) |  | T/0.994/-0.06/0.00939 | T/0.995/-0.08/0.01483 | T/1/0.409/2.24e-06 | | T/0.999/0.263/0.00197 | | T/0.005/0.012/0.5352 | | T/0.005/0.028/0.2332 |
| rs111926416 | 17 |  | [LINC00469 (118899)](http://genome.ucsc.edu/cgi-bin/hgTracks?hgHubConnect.destUrl=..%2Fcgi-bin%2FhgTracks&clade=mammal&org=Human&db=hg19&position=LINC00469) |  | A/0.99/-0.044/0.00513 | A/0.991/-0.05/0.02101 | A/0.999/0.33/1.44e-06 | | A/0.998/0.263/0.00041 | | A/0.009/0.017/0.1993 | | A/0.009/0.027/0.108 |
| rs142991058 | 17 | [KCTD2](http://genome.ucsc.edu/cgi-bin/hgTracks?hgHubConnect.destUrl=..%2Fcgi-bin%2FhgTracks&clade=mammal&org=Human&db=hg19&position=KCTD2) | [ATP5H (3096)](http://genome.ucsc.edu/cgi-bin/hgTracks?hgHubConnect.destUrl=..%2Fcgi-bin%2FhgTracks&clade=mammal&org=Human&db=hg19&position=ATP5H) | [SLC16A5 (37651)](http://genome.ucsc.edu/cgi-bin/hgTracks?hgHubConnect.destUrl=..%2Fcgi-bin%2FhgTracks&clade=mammal&org=Human&db=hg19&position=SLC16A5) | NA | NA | G/0.996/0.15/8.29e-07 | | G/0.992/0.04/0.2919 | | NA | | NA |
| rs79765414 | 18 |  | [MYL12B (47327)](http://genome.ucsc.edu/cgi-bin/hgTracks?hgHubConnect.destUrl=..%2Fcgi-bin%2FhgTracks&clade=mammal&org=Human&db=hg19&position=MYL12B) | [TGIF1 (86462)](http://genome.ucsc.edu/cgi-bin/hgTracks?hgHubConnect.destUrl=..%2Fcgi-bin%2FhgTracks&clade=mammal&org=Human&db=hg19&position=TGIF1) | NA | NA | A/0.999/0.418/3.08e-06 | | A/0.998/0.292/0.00178 | | NA | | A/0.006/0.046/0.4621 |
| rs141475580 | 18 |  | [TMEM200C (44378)](http://genome.ucsc.edu/cgi-bin/hgTracks?hgHubConnect.destUrl=..%2Fcgi-bin%2FhgTracks&clade=mammal&org=Human&db=hg19&position=TMEM200C) | [L3MBTL4 (18223)](http://genome.ucsc.edu/cgi-bin/hgTracks?hgHubConnect.destUrl=..%2Fcgi-bin%2FhgTracks&clade=mammal&org=Human&db=hg19&position=L3MBTL4) | NA | NA | A/0.999/0.416/7.82e-07 | | A/0.998/0.297/0.00109 | | NA | | NA |
| rs141318925 | 18 |  | [LOC100130480 (32388)](http://genome.ucsc.edu/cgi-bin/hgTracks?hgHubConnect.destUrl=..%2Fcgi-bin%2FhgTracks&clade=mammal&org=Human&db=hg19&position=LOC100130480) | [ARHGAP28 (165452)](http://genome.ucsc.edu/cgi-bin/hgTracks?hgHubConnect.destUrl=..%2Fcgi-bin%2FhgTracks&clade=mammal&org=Human&db=hg19&position=ARHGAP28) | NA | NA | C/1/0.459/7.91e-07 | | C/0.998/0.284/0.0011 | | NA | | NA |
| rs7243817 | 18 |  | [HRH4 (72573)](http://genome.ucsc.edu/cgi-bin/hgTracks?hgHubConnect.destUrl=..%2Fcgi-bin%2FhgTracks&clade=mammal&org=Human&db=hg19&position=HRH4) |  | C/0.212/0.001/0.7694 | C/0.212/-0.003/0.4803 | C/0.134/0.021/3.8e-06 | | C/0.134/0.027/0.0042 | | T/0.805/0.006/0.00485 | | T/0.786/0.003/0.3243 |
| rs76752096 | 18 | [AK127888](http://genome.ucsc.edu/cgi-bin/hgTracks?hgHubConnect.destUrl=..%2Fcgi-bin%2FhgTracks&clade=mammal&org=Human&db=hg19&position=AK127888) |  |  | NA | NA | A/0.999/0.431/7.77e-09 | | A/0.998/0.325/3.56e-05 | | NA | | NA |
| rs192178588 | 18 |  |  |  | NA | NA | A/0.996/0.171/5.12e-07 | | A/0.993/0.155/0.00021 | | NA | | NA |
| rs148187869 | 18 |  |  |  | NA | NA | A/0.997/0.228/3.42e-07 | | A/0.995/0.24/8.15e-06 | | NA | | NA |
| rs146111930 | 18 |  |  |  | NA | NA | A/0.997/0.228/3.46e-07 | | A/0.995/0.24/8.15e-06 | | NA | | NA |
| rs149083189 | 18 |  |  |  | NA | NA | G/0.998/0.292/3.91e-09 | | G/0.996/0.249/4.3e-06 | | NA | | NA |
| rs190650493 | 18 | [C18orf34](http://genome.ucsc.edu/cgi-bin/hgTracks?hgHubConnect.destUrl=..%2Fcgi-bin%2FhgTracks&clade=mammal&org=Human&db=hg19&position=C18orf34) |  |  | NA | NA | G/0.999/0.362/2.97e-06 | | G/0.998/0.165/0.02856 | | NA | | NA |
| rs190343823 | 18 | [MOCOS](http://genome.ucsc.edu/cgi-bin/hgTracks?hgHubConnect.destUrl=..%2Fcgi-bin%2FhgTracks&clade=mammal&org=Human&db=hg19&position=MOCOS) | [BC009735 (67938)](http://genome.ucsc.edu/cgi-bin/hgTracks?hgHubConnect.destUrl=..%2Fcgi-bin%2FhgTracks&clade=mammal&org=Human&db=hg19&position=BC009735) | [FHOD3 (42365)](http://genome.ucsc.edu/cgi-bin/hgTracks?hgHubConnect.destUrl=..%2Fcgi-bin%2FhgTracks&clade=mammal&org=Human&db=hg19&position=FHOD3) | NA | NA | C/0.999/0.549/2.18e-07 | | C/0.999/0.375/0.00034 | | NA | | NA |
| rs184167040 | 18 |  |  |  | NA | NA | A/1/0.544/1.41e-06 | | A/0.999/0.379/0.00053 | | NA | | NA |
| rs148138116 | 18 | [TCF4](http://genome.ucsc.edu/cgi-bin/hgTracks?hgHubConnect.destUrl=..%2Fcgi-bin%2FhgTracks&clade=mammal&org=Human&db=hg19&position=TCF4) |  |  | NA | NA | T/0.997/0.175/3.04e-06 | | T/0.995/0.175/3e-04 | | NA | | NA |
| rs189265675 | 19 | [VMAC](http://genome.ucsc.edu/cgi-bin/hgTracks?hgHubConnect.destUrl=..%2Fcgi-bin%2FhgTracks&clade=mammal&org=Human&db=hg19&position=VMAC) | [NDUFA11 (3399)](http://genome.ucsc.edu/cgi-bin/hgTracks?hgHubConnect.destUrl=..%2Fcgi-bin%2FhgTracks&clade=mammal&org=Human&db=hg19&position=NDUFA11) | [CAPS (6769)](http://genome.ucsc.edu/cgi-bin/hgTracks?hgHubConnect.destUrl=..%2Fcgi-bin%2FhgTracks&clade=mammal&org=Human&db=hg19&position=CAPS) | NA | NA | T/1/0.597/4.55e-06 | | T/0.999/0.387/0.00197 | | NA | | NA |
| rs111563375 | 19 | [VMAC](http://genome.ucsc.edu/cgi-bin/hgTracks?hgHubConnect.destUrl=..%2Fcgi-bin%2FhgTracks&clade=mammal&org=Human&db=hg19&position=VMAC) | [NDUFA11 (4738)](http://genome.ucsc.edu/cgi-bin/hgTracks?hgHubConnect.destUrl=..%2Fcgi-bin%2FhgTracks&clade=mammal&org=Human&db=hg19&position=NDUFA11) | [CAPS (5430)](http://genome.ucsc.edu/cgi-bin/hgTracks?hgHubConnect.destUrl=..%2Fcgi-bin%2FhgTracks&clade=mammal&org=Human&db=hg19&position=CAPS) | NA | NA | T/1/0.591/4.84e-06 | | T/0.999/0.384/0.00196 | | NA | | NA |
| rs142119409 | 19 |  | [OR10H4 (55663)](http://genome.ucsc.edu/cgi-bin/hgTracks?hgHubConnect.destUrl=..%2Fcgi-bin%2FhgTracks&clade=mammal&org=Human&db=hg19&position=OR10H4) | [LOC126536 (10012)](http://genome.ucsc.edu/cgi-bin/hgTracks?hgHubConnect.destUrl=..%2Fcgi-bin%2FhgTracks&clade=mammal&org=Human&db=hg19&position=LOC126536) | C/0.995/-0.006/0.82 | C/0.996/-0.017/0.7031 | C/0.999/0.308/8.97e-07 | | C/0.997/0.197/0.00274 | | NA | | T/0.996/-0.017/0.7023 |
| rs184381023 | 19 |  | [ATP5SL (1382)](http://genome.ucsc.edu/cgi-bin/hgTracks?hgHubConnect.destUrl=..%2Fcgi-bin%2FhgTracks&clade=mammal&org=Human&db=hg19&position=ATP5SL) | [C19orf69 (1837)](http://genome.ucsc.edu/cgi-bin/hgTracks?hgHubConnect.destUrl=..%2Fcgi-bin%2FhgTracks&clade=mammal&org=Human&db=hg19&position=C19orf69) | NA | NA | G/0.992/-0.104/4.45e-06 | | G/0.996/-0.173/0.1472 | | NA | | NA |
| rs139704751 | 19 |  | [PTPRH (14604)](http://genome.ucsc.edu/cgi-bin/hgTracks?hgHubConnect.destUrl=..%2Fcgi-bin%2FhgTracks&clade=mammal&org=Human&db=hg19&position=PTPRH) | [TMEM86B (2523)](http://genome.ucsc.edu/cgi-bin/hgTracks?hgHubConnect.destUrl=..%2Fcgi-bin%2FhgTracks&clade=mammal&org=Human&db=hg19&position=TMEM86B) | T/0.997/-0.027/0.4699 | T/0.997/-0.066/0.1501 | T/1/0.59/1.31e-06 | | T/0.999/0.335/0.00303 | | NA | | T/0.003/0.066/0.1498 |
| rs191335472 | 20 |  | [AK090681 (54189)](http://genome.ucsc.edu/cgi-bin/hgTracks?hgHubConnect.destUrl=..%2Fcgi-bin%2FhgTracks&clade=mammal&org=Human&db=hg19&position=AK090681) | [STK35 (40175)](http://genome.ucsc.edu/cgi-bin/hgTracks?hgHubConnect.destUrl=..%2Fcgi-bin%2FhgTracks&clade=mammal&org=Human&db=hg19&position=STK35) | NA | NA | A/0.999/0.482/2.56e-06 | | A/0.998/0.295/0.00323 | | NA | | NA |
| rs190821608 | 20 | [KIF16B](http://genome.ucsc.edu/cgi-bin/hgTracks?hgHubConnect.destUrl=..%2Fcgi-bin%2FhgTracks&clade=mammal&org=Human&db=hg19&position=KIF16B) | [MACROD2 (249603)](http://genome.ucsc.edu/cgi-bin/hgTracks?hgHubConnect.destUrl=..%2Fcgi-bin%2FhgTracks&clade=mammal&org=Human&db=hg19&position=MACROD2) |  | NA | NA | C/1/0.488/2.92e-06 | | C/0.999/0.338/0.00061 | | NA | | NA |
| rs143677104 | 20 |  | [miR-449b-3P (58598)](http://genome.ucsc.edu/cgi-bin/hgTracks?hgHubConnect.destUrl=..%2Fcgi-bin%2FhgTracks&clade=mammal&org=Human&db=hg19&position=miR-449b-3P) |  | NA | NA | A/1/0.562/4.68e-06 | | A/0.999/0.269/0.01855 | | NA | | NA |
| rs187497509 | 20 | [R3HDML](http://genome.ucsc.edu/cgi-bin/hgTracks?hgHubConnect.destUrl=..%2Fcgi-bin%2FhgTracks&clade=mammal&org=Human&db=hg19&position=R3HDML) | [FITM2 (39682)](http://genome.ucsc.edu/cgi-bin/hgTracks?hgHubConnect.destUrl=..%2Fcgi-bin%2FhgTracks&clade=mammal&org=Human&db=hg19&position=FITM2) | [HNF4A (4869)](http://genome.ucsc.edu/cgi-bin/hgTracks?hgHubConnect.destUrl=..%2Fcgi-bin%2FhgTracks&clade=mammal&org=Human&db=hg19&position=HNF4A) | G/0.998/-0.004/0.9138 | G/0.998/-0.001/0.992 | G/1/0.404/4.63e-06 | | G/0.999/0.285/0.00055 | | C/0.998/0.072/0.01644 | | C/0.997/0.052/0.1372 |
| rs74275091 | 20 |  | [SLC2A10 (153448)](http://genome.ucsc.edu/cgi-bin/hgTracks?hgHubConnect.destUrl=..%2Fcgi-bin%2FhgTracks&clade=mammal&org=Human&db=hg19&position=SLC2A10) | [EYA2 (4829)](http://genome.ucsc.edu/cgi-bin/hgTracks?hgHubConnect.destUrl=..%2Fcgi-bin%2FhgTracks&clade=mammal&org=Human&db=hg19&position=EYA2) | NA | NA | T/1/0.675/2.64e-06 | | T/0.999/0.354/0.00946 | | NA | | NA |
| rs6069919 | 20 |  | [TFAP2C (241371)](http://genome.ucsc.edu/cgi-bin/hgTracks?hgHubConnect.destUrl=..%2Fcgi-bin%2FhgTracks&clade=mammal&org=Human&db=hg19&position=TFAP2C) |  | NA | NA | T/0.908/0.028/2.28e-06 | | T/0.89/0.016/0.1163 | | NA | | NA |
| rs117094540 | 20 | [NPEPL1](http://genome.ucsc.edu/cgi-bin/hgTracks?hgHubConnect.destUrl=..%2Fcgi-bin%2FhgTracks&clade=mammal&org=Human&db=hg19&position=NPEPL1) | [STX16 (16082)](http://genome.ucsc.edu/cgi-bin/hgTracks?hgHubConnect.destUrl=..%2Fcgi-bin%2FhgTracks&clade=mammal&org=Human&db=hg19&position=STX16) | [BC007940 (58741)](http://genome.ucsc.edu/cgi-bin/hgTracks?hgHubConnect.destUrl=..%2Fcgi-bin%2FhgTracks&clade=mammal&org=Human&db=hg19&position=BC007940) | T/0.995/0.031/0.2443 | T/0.994/0.008/0.8177 | T/0.999/0.35/1.02e-06 | | T/0.998/0.215/0.00845 | | T/0.005/-0.067/0.00083 | | T/0.006/-0.026/0.2418 |
| rs139222478 | 20 | [NPEPL1](http://genome.ucsc.edu/cgi-bin/hgTracks?hgHubConnect.destUrl=..%2Fcgi-bin%2FhgTracks&clade=mammal&org=Human&db=hg19&position=NPEPL1) | [STX16 (30347)](http://genome.ucsc.edu/cgi-bin/hgTracks?hgHubConnect.destUrl=..%2Fcgi-bin%2FhgTracks&clade=mammal&org=Human&db=hg19&position=STX16) | [BC007940 (44476)](http://genome.ucsc.edu/cgi-bin/hgTracks?hgHubConnect.destUrl=..%2Fcgi-bin%2FhgTracks&clade=mammal&org=Human&db=hg19&position=BC007940) | G/0.996/0.033/0.2564 | G/0.996/0.014/0.7127 | G/0.999/0.366/3.16e-06 | | G/0.998/0.228/0.01318 | | T/0.996/0.073/0.00109 | | T/0.995/0.029/0.2503 |
| rs76507695 | 20 |  | [BC007940 (24160)](http://genome.ucsc.edu/cgi-bin/hgTracks?hgHubConnect.destUrl=..%2Fcgi-bin%2FhgTracks&clade=mammal&org=Human&db=hg19&position=BC007940) | [MIR296 (36618)](http://genome.ucsc.edu/cgi-bin/hgTracks?hgHubConnect.destUrl=..%2Fcgi-bin%2FhgTracks&clade=mammal&org=Human&db=hg19&position=MIR296) | G/0.994/0.03/0.2036 | G/0.994/0.014/0.6531 | G/0.999/0.328/2.77e-07 | | G/0.998/0.187/0.00851 | | A/0.995/0.059/0.00106 | | A/0.994/0.021/0.2966 |
| rs186485497 | 20 | [GNAS](http://genome.ucsc.edu/cgi-bin/hgTracks?hgHubConnect.destUrl=..%2Fcgi-bin%2FhgTracks&clade=mammal&org=Human&db=hg19&position=GNAS) | [GNAS-AS1 (31384)](http://genome.ucsc.edu/cgi-bin/hgTracks?hgHubConnect.destUrl=..%2Fcgi-bin%2FhgTracks&clade=mammal&org=Human&db=hg19&position=GNAS-AS1) | [AK093534 (12909)](http://genome.ucsc.edu/cgi-bin/hgTracks?hgHubConnect.destUrl=..%2Fcgi-bin%2FhgTracks&clade=mammal&org=Human&db=hg19&position=AK093534) | A/0.995/0.031/0.2079 | A/0.995/0.018/0.5615 | A/0.999/0.278/2.45e-06 | | A/0.998/0.141/0.03902 | | A/0.004/-0.07/0.00033 | | A/0.005/-0.03/0.1888 |
| rs117041046 | 20 | [GNAS](http://genome.ucsc.edu/cgi-bin/hgTracks?hgHubConnect.destUrl=..%2Fcgi-bin%2FhgTracks&clade=mammal&org=Human&db=hg19&position=GNAS) | [AK093534 (11501)](http://genome.ucsc.edu/cgi-bin/hgTracks?hgHubConnect.destUrl=..%2Fcgi-bin%2FhgTracks&clade=mammal&org=Human&db=hg19&position=AK093534) | [TH1L (74070)](http://genome.ucsc.edu/cgi-bin/hgTracks?hgHubConnect.destUrl=..%2Fcgi-bin%2FhgTracks&clade=mammal&org=Human&db=hg19&position=TH1L) | C/0.996/0.022/0.3895 | C/0.996/0.01/0.757 | C/0.999/0.309/9.34e-07 | | C/0.998/0.156/0.02265 | | A/0.997/0.07/6e-04 | | A/0.996/0.033/0.1741 |
| rs147130662 | 21 |  |  |  | NA | NA | C/0.998/0.227/3.68e-06 | | C/0.996/0.122/0.01267 | | NA | | NA |
| rs139916902 | 21 |  |  |  | NA | NA | G/0.999/0.263/2.08e-07 | | G/0.996/0.116/0.0168 | | NA | | NA |
| rs142181472 | 21 |  |  |  | NA | NA | C/0.998/0.238/3.49e-07 | | C/0.996/0.114/0.01546 | | NA | | NA |
| rs142429672 | 21 |  |  |  | NA | NA | C/0.999/0.274/9.73e-08 | | C/0.996/0.114/0.0186 | | NA | | NA |
| rs141838403 | 21 |  |  |  | NA | NA | C/0.999/0.282/9.22e-08 | | C/0.996/0.118/0.01691 | | NA | | NA |
| rs139202427 | 21 |  |  |  | NA | NA | A/0.999/0.301/1.09e-07 | | A/0.996/0.126/0.01892 | | NA | | NA |
| rs142976294 | 21 |  |  |  | NA | NA | G/0.999/0.314/1.29e-07 | | G/0.996/0.131/0.02009 | | NA | | NA |
| rs182382461 | 22 | [AK131325](http://genome.ucsc.edu/cgi-bin/hgTracks?hgHubConnect.destUrl=..%2Fcgi-bin%2FhgTracks&clade=mammal&org=Human&db=hg19&position=AK131325) | [TOP3B (21754)](http://genome.ucsc.edu/cgi-bin/hgTracks?hgHubConnect.destUrl=..%2Fcgi-bin%2FhgTracks&clade=mammal&org=Human&db=hg19&position=TOP3B) | [BC089413 (21520)](http://genome.ucsc.edu/cgi-bin/hgTracks?hgHubConnect.destUrl=..%2Fcgi-bin%2FhgTracks&clade=mammal&org=Human&db=hg19&position=BC089413) | NA | NA | G/0.999/0.509/1.06e-06 | | G/0.998/0.187/0.06294 | | NA | | NA |
| rs139509979 | 22 |  | [SYN3 (182339)](http://genome.ucsc.edu/cgi-bin/hgTracks?hgHubConnect.destUrl=..%2Fcgi-bin%2FhgTracks&clade=mammal&org=Human&db=hg19&position=SYN3) | [LARGE (83913)](http://genome.ucsc.edu/cgi-bin/hgTracks?hgHubConnect.destUrl=..%2Fcgi-bin%2FhgTracks&clade=mammal&org=Human&db=hg19&position=LARGE) | NA | NA | G/0.999/0.41/4.81e-06 | | G/0.998/0.27/0.00196 | | NA | | T/0.994/-0.133/0.05566 |
| rs183544847 | 22 | [LOC646851](http://genome.ucsc.edu/cgi-bin/hgTracks?hgHubConnect.destUrl=..%2Fcgi-bin%2FhgTracks&clade=mammal&org=Human&db=hg19&position=LOC646851) | [DMC1 (25364)](http://genome.ucsc.edu/cgi-bin/hgTracks?hgHubConnect.destUrl=..%2Fcgi-bin%2FhgTracks&clade=mammal&org=Human&db=hg19&position=DMC1) | [CBY1 (61104)](http://genome.ucsc.edu/cgi-bin/hgTracks?hgHubConnect.destUrl=..%2Fcgi-bin%2FhgTracks&clade=mammal&org=Human&db=hg19&position=CBY1) | G/0.997/0.058/0.1612 | G/0.996/0.013/0.7888 | G/0.999/0.384/3.34e-06 | | G/0.998/0.204/0.00825 | | NA | | A/0.993/-0.007/0.8348 |
| rs149157446 | 22 |  | [PNPLA5 (17681)](http://genome.ucsc.edu/cgi-bin/hgTracks?hgHubConnect.destUrl=..%2Fcgi-bin%2FhgTracks&clade=mammal&org=Human&db=hg19&position=PNPLA5) | [PNPLA3 (14044)](http://genome.ucsc.edu/cgi-bin/hgTracks?hgHubConnect.destUrl=..%2Fcgi-bin%2FhgTracks&clade=mammal&org=Human&db=hg19&position=PNPLA3) | G/0.996/0.012/0.6921 | G/0.995/0.013/0.7169 | G/0.999/0.502/3.02e-06 | | G/0.998/0.25/0.0132 | | A/0.995/0.044/0.04201 | | A/0.994/0.022/0.3273 |

**Table S2:** Top SNPs with a p-value < 5E-06 for FEV1 among AA in COPDGene.

| SNP | Chr | Gene | Nearest Gene Up | Nearest Gene Down | COPDGene NHW All subjects | COPDGene NHW Cases Only | COPDGene AA All subjects | COPDGene AA Cases Only | Meta-analysis All Subjects | Meta-analysis Cases Only |
| --- | --- | --- | --- | --- | --- | --- | --- | --- | --- | --- |
|  |  |  |  |  | Coded Allele /Beta/P | Coded Allele /Beta/P | Coded Allele /Beta/P | Coded Allele / Beta/ P | Coded Allele / Beta/ P | Coded Allele / Beta/ P |
| rs140401531 | 1 | [CAMTA1](http://genome.ucsc.edu/cgi-bin/hgTracks?hgHubConnect.destUrl=..%2Fcgi-bin%2FhgTracks&clade=mammal&org=Human&db=hg19&position=CAMTA1) |  | [VAMP3 (177126)](http://genome.ucsc.edu/cgi-bin/hgTracks?hgHubConnect.destUrl=..%2Fcgi-bin%2FhgTracks&clade=mammal&org=Human&db=hg19&position=VAMP3) | A/0.989/-0.146/0.09483 | A/0.99/-0.05/0.6326 | A/0.874/0.137/2e-06 | A/0.852/0.054/0.1859 | A/0.11/-0.108/5.25e-05 | A/0.113/-0.044/0.214 |
| rs183468661 | 1 | [RLF](http://genome.ucsc.edu/cgi-bin/hgTracks?hgHubConnect.destUrl=..%2Fcgi-bin%2FhgTracks&clade=mammal&org=Human&db=hg19&position=RLF) | [PPT1 (71700)](http://genome.ucsc.edu/cgi-bin/hgTracks?hgHubConnect.destUrl=..%2Fcgi-bin%2FhgTracks&clade=mammal&org=Human&db=hg19&position=PPT1) | [TMCO2 (78730)](http://genome.ucsc.edu/cgi-bin/hgTracks?hgHubConnect.destUrl=..%2Fcgi-bin%2FhgTracks&clade=mammal&org=Human&db=hg19&position=TMCO2) | NA | NA | T/0.999/1.915/2.45e-06 | T/0.998/1.132/0.00187 | NA | NA |
| rs139175374 | 1 | [USP33](http://genome.ucsc.edu/cgi-bin/hgTracks?hgHubConnect.destUrl=..%2Fcgi-bin%2FhgTracks&clade=mammal&org=Human&db=hg19&position=USP33) | [ZZZ3 (60312)](http://genome.ucsc.edu/cgi-bin/hgTracks?hgHubConnect.destUrl=..%2Fcgi-bin%2FhgTracks&clade=mammal&org=Human&db=hg19&position=ZZZ3) | [FAM73A (35892)](http://genome.ucsc.edu/cgi-bin/hgTracks?hgHubConnect.destUrl=..%2Fcgi-bin%2FhgTracks&clade=mammal&org=Human&db=hg19&position=FAM73A) | C/0.997/0.086/0.642 | C/0.996/0.23/0.2355 | C/0.999/1.8/4.24e-06 | C/0.999/1.038/0.01413 | NA | NA |
| rs181870845 | 1 | [GIPC2](http://genome.ucsc.edu/cgi-bin/hgTracks?hgHubConnect.destUrl=..%2Fcgi-bin%2FhgTracks&clade=mammal&org=Human&db=hg19&position=GIPC2) | [DNAJB4 (49570)](http://genome.ucsc.edu/cgi-bin/hgTracks?hgHubConnect.destUrl=..%2Fcgi-bin%2FhgTracks&clade=mammal&org=Human&db=hg19&position=DNAJB4) | [MGC27382 (162717)](http://genome.ucsc.edu/cgi-bin/hgTracks?hgHubConnect.destUrl=..%2Fcgi-bin%2FhgTracks&clade=mammal&org=Human&db=hg19&position=MGC27382) | NA | NA | A/0.999/1.909/3.62e-06 | A/0.999/1.14/0.01102 | NA | NA |
| rs114304737 | 1 |  | [COL24A1 (109275)](http://genome.ucsc.edu/cgi-bin/hgTracks?hgHubConnect.destUrl=..%2Fcgi-bin%2FhgTracks&clade=mammal&org=Human&db=hg19&position=COL24A1) | [ODF2L (81077)](http://genome.ucsc.edu/cgi-bin/hgTracks?hgHubConnect.destUrl=..%2Fcgi-bin%2FhgTracks&clade=mammal&org=Human&db=hg19&position=ODF2L) | A/0.98/-0.027/0.5593 | A/0.98/-0.139/0.00897 | A/0.997/0.771/2.9e-06 | A/0.994/0.41/0.01812 | A/0.02/0.011/0.7625 | A/0.02/0.067/0.06594 |
| rs55916418 | 1 |  | [COL24A1 (130764)](http://genome.ucsc.edu/cgi-bin/hgTracks?hgHubConnect.destUrl=..%2Fcgi-bin%2FhgTracks&clade=mammal&org=Human&db=hg19&position=COL24A1) | [ODF2L (59588)](http://genome.ucsc.edu/cgi-bin/hgTracks?hgHubConnect.destUrl=..%2Fcgi-bin%2FhgTracks&clade=mammal&org=Human&db=hg19&position=ODF2L) | T/0.991/0.014/0.8525 | T/0.991/-0.072/0.3913 | T/0.999/1.159/2.1e-06 | T/0.997/0.58/0.01753 | A/0.992/0.067/0.2742 | A/0.992/-0.01/0.8645 |
| rs138897663 | 1 |  |  |  | NA | NA | A/0.999/1.327/7.08e-07 | A/0.997/0.74/0.00326 | NA | NA |
| rs150884133 | 1 |  |  |  | NA | NA | A/0.998/1.221/2.56e-07 | A/0.996/0.674/0.00207 | NA | NA |
| rs137917644 | 1 |  |  |  | NA | NA | T/0.998/1.212/2.39e-07 | T/0.996/0.668/0.002 | NA | NA |
| rs187464554 | 1 |  |  |  | NA | NA | T/0.998/1.212/2.39e-07 | T/0.996/0.668/0.002 | NA | NA |
| rs187226391 | 1 |  |  |  | NA | NA | C/0.998/1.235/1.94e-07 | C/0.996/0.682/0.00173 | NA | NA |
| rs191953093 | 1 | [MR1](http://genome.ucsc.edu/cgi-bin/hgTracks?hgHubConnect.destUrl=..%2Fcgi-bin%2FhgTracks&clade=mammal&org=Human&db=hg19&position=MR1) | [STX6 (14835)](http://genome.ucsc.edu/cgi-bin/hgTracks?hgHubConnect.destUrl=..%2Fcgi-bin%2FhgTracks&clade=mammal&org=Human&db=hg19&position=STX6) | [IER5 (50756)](http://genome.ucsc.edu/cgi-bin/hgTracks?hgHubConnect.destUrl=..%2Fcgi-bin%2FhgTracks&clade=mammal&org=Human&db=hg19&position=IER5) | A/0.998/-0.352/0.1137 | A/0.999/0.149/0.6369 | A/1/2.752/1.75e-06 | A/0.999/1.498/0.00115 | NA | NA |
| rs148428208 | 1 | [AF508906](http://genome.ucsc.edu/cgi-bin/hgTracks?hgHubConnect.destUrl=..%2Fcgi-bin%2FhgTracks&clade=mammal&org=Human&db=hg19&position=AF508906) |  | [AF508907 (9764)](http://genome.ucsc.edu/cgi-bin/hgTracks?hgHubConnect.destUrl=..%2Fcgi-bin%2FhgTracks&clade=mammal&org=Human&db=hg19&position=AF508907) | G/0.994/0.126/0.223 | G/0.994/-0.042/0.7073 | G/0.998/-1.173/2.54e-06 | G/0.999/1.127/0.6425 | T/0.994/-0.024/0.7475 | T/0.993/-0.021/0.764 |
| rs191506206 | 1 |  |  |  | NA | NA | G/0.999/1.375/2.74e-06 | G/0.998/0.688/0.01733 | NA | NA |
| rs138157192 | 1 |  |  | [RGS18 (84134)](http://genome.ucsc.edu/cgi-bin/hgTracks?hgHubConnect.destUrl=..%2Fcgi-bin%2FhgTracks&clade=mammal&org=Human&db=hg19&position=RGS18) | NA | NA | A/0.999/1.452/1.82e-06 | A/0.998/0.703/0.01501 | NA | NA |
| rs187061080 | 1 | [RGS21](http://genome.ucsc.edu/cgi-bin/hgTracks?hgHubConnect.destUrl=..%2Fcgi-bin%2FhgTracks&clade=mammal&org=Human&db=hg19&position=RGS21) |  |  | A/0.999/0.322/0.2541 | A/0.999/0.243/0.563 | A/0.999/1.501/2.56e-06 | A/0.998/0.724/0.01208 | NA | NA |
| rs79237515 | 1 |  |  |  | T/0.988/-0.023/0.7446 | T/0.988/0/0.9995 | T/0.998/1.326/2.51e-07 | T/0.996/0.499/0.02647 | T/0.012/-0.016/0.7735 | T/0.012/-0.015/0.7804 |
| rs138325697 | 1 |  |  |  | T/0.985/-0.053/0.4588 | T/0.985/-0.026/0.7605 | T/0.997/1.132/4.06e-07 | T/0.995/0.435/0.03482 | T/0.014/-0.017/0.7615 | T/0.015/-0.008/0.8892 |
| rs144324177 | 1 |  |  |  | T/0.988/0.014/0.8728 | T/0.988/-0.038/0.7097 | T/0.998/1.171/6.91e-07 | T/0.996/0.521/0.01644 | T/0.011/-0.117/0.1033 | T/0.011/-0.014/0.842 |
| rs144276489 | 1 |  |  |  | A/0.986/0/0.9956 | A/0.987/-0.037/0.6929 | A/0.997/1.032/3.64e-06 | A/0.995/0.509/0.01767 | A/0.012/-0.092/0.1694 | A/0.012/-0.02/0.7585 |
| rs78702938 | 1 |  |  |  | G/0.991/-0.022/0.8381 | G/0.99/-0.062/0.5963 | G/0.997/1.088/5.11e-07 | G/0.994/0.423/0.03269 | NA | NA |
| rs74507156 | 1 |  |  |  | A/0.997/0.012/0.9355 | A/0.997/-0.269/0.1093 | A/1/2.162/3.62e-06 | A/0.999/1.333/0.00054 | A/0.003/-0.061/0.608 | A/0.003/0.008/0.949 |
| rs189354479 | 1 |  |  |  | G/0.999/-0.142/0.619 | G/0.999/-0.389/0.302 | G/1/2.728/1.93e-06 | G/0.999/1.515/0.00061 | T/0.999/0.175/0.4082 | T/0.999/0.07/0.73 |
| rs191211294 | 1 | [KCNT2](http://genome.ucsc.edu/cgi-bin/hgTracks?hgHubConnect.destUrl=..%2Fcgi-bin%2FhgTracks&clade=mammal&org=Human&db=hg19&position=KCNT2) |  |  | NA | NA | A/0.999/1.47/4.1e-06 | A/0.998/0.89/0.00183 | NA | NA |
| rs139052640 | 1 |  | [NENF (108649)](http://genome.ucsc.edu/cgi-bin/hgTracks?hgHubConnect.destUrl=..%2Fcgi-bin%2FhgTracks&clade=mammal&org=Human&db=hg19&position=NENF) | [ATF3 (10305)](http://genome.ucsc.edu/cgi-bin/hgTracks?hgHubConnect.destUrl=..%2Fcgi-bin%2FhgTracks&clade=mammal&org=Human&db=hg19&position=ATF3) | C/0.998/0.581/0.03101 | C/0.998/0.645/0.02366 | C/1/3.397/2.29e-06 | C/0.999/1.853/0.00085 | T/0.998/0.584/0.00274 | T/0.998/0.316/0.0785 |
| rs79131758 | 1 | [ESRRG](http://genome.ucsc.edu/cgi-bin/hgTracks?hgHubConnect.destUrl=..%2Fcgi-bin%2FhgTracks&clade=mammal&org=Human&db=hg19&position=ESRRG) |  |  | C/0.994/0.27/0.02279 | C/0.994/0.149/0.2282 | C/0.998/1.231/7.72e-07 | C/0.996/0.405/0.05665 | C/0.009/-0.166/0.01912 | C/0.009/-0.083/0.1986 |
| rs187984862 | 1 |  |  |  | C/0.998/0.067/0.728 | C/0.998/0.068/0.7535 | C/1/2.334/4.1e-07 | C/0.998/1.058/0.00296 | NA | NA |
| rs185275068 | 1 | [CHRM3](http://genome.ucsc.edu/cgi-bin/hgTracks?hgHubConnect.destUrl=..%2Fcgi-bin%2FhgTracks&clade=mammal&org=Human&db=hg19&position=CHRM3) |  |  | A/0.992/0.103/0.413 | A/0.992/0.075/0.5933 | A/0.999/1.622/1.36e-06 | A/0.997/0.701/0.00819 | NA | NA |
| rs188686089 | 1 | [CHRM3](http://genome.ucsc.edu/cgi-bin/hgTracks?hgHubConnect.destUrl=..%2Fcgi-bin%2FhgTracks&clade=mammal&org=Human&db=hg19&position=CHRM3) |  |  | A/0.993/0.127/0.3608 | A/0.993/0.106/0.4946 | A/0.999/1.758/1.15e-06 | A/0.997/0.705/0.01295 | NA | NA |
| rs77830002 | 1 | [CHRM3](http://genome.ucsc.edu/cgi-bin/hgTracks?hgHubConnect.destUrl=..%2Fcgi-bin%2FhgTracks&clade=mammal&org=Human&db=hg19&position=CHRM3) |  |  | T/0.995/0.094/0.5256 | T/0.995/0.041/0.8036 | T/0.999/1.776/1.22e-06 | T/0.998/0.747/0.00894 | NA | NA |
| rs76759221 | 1 | [CHRM3](http://genome.ucsc.edu/cgi-bin/hgTracks?hgHubConnect.destUrl=..%2Fcgi-bin%2FhgTracks&clade=mammal&org=Human&db=hg19&position=CHRM3) |  |  | NA | NA | C/0.998/1.438/7.28e-07 | C/0.996/0.559/0.02604 | NA | NA |
| rs116229052 | 1 | [RGS7](http://genome.ucsc.edu/cgi-bin/hgTracks?hgHubConnect.destUrl=..%2Fcgi-bin%2FhgTracks&clade=mammal&org=Human&db=hg19&position=RGS7) |  |  | T/0.963/-0.005/0.9075 | T/0.962/-0.015/0.7417 | T/0.991/0.497/4.64e-06 | T/0.986/0.043/0.7392 | T/0.036/-0.041/0.1877 | T/0.037/-0.003/0.9199 |
| rs115107872 | 1 | [SMYD3](http://genome.ucsc.edu/cgi-bin/hgTracks?hgHubConnect.destUrl=..%2Fcgi-bin%2FhgTracks&clade=mammal&org=Human&db=hg19&position=SMYD3) | [KIF26B (225155)](http://genome.ucsc.edu/cgi-bin/hgTracks?hgHubConnect.destUrl=..%2Fcgi-bin%2FhgTracks&clade=mammal&org=Human&db=hg19&position=KIF26B) |  | NA | NA | G/0.973/0.275/3.56e-06 | G/0.965/0.106/0.1652 | NA | NA |
| rs116335148 | 1 | [SMYD3](http://genome.ucsc.edu/cgi-bin/hgTracks?hgHubConnect.destUrl=..%2Fcgi-bin%2FhgTracks&clade=mammal&org=Human&db=hg19&position=SMYD3) | [KIF26B (228691)](http://genome.ucsc.edu/cgi-bin/hgTracks?hgHubConnect.destUrl=..%2Fcgi-bin%2FhgTracks&clade=mammal&org=Human&db=hg19&position=KIF26B) |  | NA | NA | T/0.973/0.273/3.66e-06 | T/0.964/0.107/0.1602 | NA | NA |
| rs116647499 | 1 | [SMYD3](http://genome.ucsc.edu/cgi-bin/hgTracks?hgHubConnect.destUrl=..%2Fcgi-bin%2FhgTracks&clade=mammal&org=Human&db=hg19&position=SMYD3) | [KIF26B (230775)](http://genome.ucsc.edu/cgi-bin/hgTracks?hgHubConnect.destUrl=..%2Fcgi-bin%2FhgTracks&clade=mammal&org=Human&db=hg19&position=KIF26B) |  | NA | NA | A/0.973/0.274/3.58e-06 | A/0.965/0.106/0.1657 | NA | NA |
| rs150411716 | 2 |  | [FAM150B (86401)](http://genome.ucsc.edu/cgi-bin/hgTracks?hgHubConnect.destUrl=..%2Fcgi-bin%2FhgTracks&clade=mammal&org=Human&db=hg19&position=FAM150B) |  | T/0.991/-0.074/0.4886 | T/0.991/-0.178/0.1079 | T/0.999/1.69/2.54e-06 | T/0.997/0.967/0.00248 | NA | NA |
| rs116554435 | 2 |  | [FAM150B (92976)](http://genome.ucsc.edu/cgi-bin/hgTracks?hgHubConnect.destUrl=..%2Fcgi-bin%2FhgTracks&clade=mammal&org=Human&db=hg19&position=FAM150B) |  | G/0.991/-0.077/0.4707 | G/0.991/-0.173/0.1186 | G/0.999/1.686/2.24e-06 | G/0.997/0.962/0.00256 | NA | NA |
| rs116375068 | 2 | [AK001558](http://genome.ucsc.edu/cgi-bin/hgTracks?hgHubConnect.destUrl=..%2Fcgi-bin%2FhgTracks&clade=mammal&org=Human&db=hg19&position=AK001558) | [SNORD18 (42305)](http://genome.ucsc.edu/cgi-bin/hgTracks?hgHubConnect.destUrl=..%2Fcgi-bin%2FhgTracks&clade=mammal&org=Human&db=hg19&position=SNORD18) |  | A/0.99/-0.035/0.6403 | A/0.99/-0.042/0.6163 | A/0.998/1.007/9.42e-07 | A/0.995/0.428/0.02589 | A/0.011/-0.068/0.2075 | A/0.012/-0.069/0.1752 |
| rs146495511 | 2 | [CIB4](http://genome.ucsc.edu/cgi-bin/hgTracks?hgHubConnect.destUrl=..%2Fcgi-bin%2FhgTracks&clade=mammal&org=Human&db=hg19&position=CIB4) | [C2orf70 (23303)](http://genome.ucsc.edu/cgi-bin/hgTracks?hgHubConnect.destUrl=..%2Fcgi-bin%2FhgTracks&clade=mammal&org=Human&db=hg19&position=C2orf70) | [KCNK3 (89882)](http://genome.ucsc.edu/cgi-bin/hgTracks?hgHubConnect.destUrl=..%2Fcgi-bin%2FhgTracks&clade=mammal&org=Human&db=hg19&position=KCNK3) | A/0.995/0.091/0.537 | A/0.996/0.12/0.4916 | A/0.998/1.449/2.89e-06 | A/0.996/0.857/0.00255 | A/0.004/-0.069/0.515 | A/0.004/-0.054/0.6024 |
| rs148732433 | 2 |  | [SLC8A1 (234257)](http://genome.ucsc.edu/cgi-bin/hgTracks?hgHubConnect.destUrl=..%2Fcgi-bin%2FhgTracks&clade=mammal&org=Human&db=hg19&position=SLC8A1) |  | NA | NA | T/0.992/0.466/1.98e-06 | T/0.986/0.18/0.09596 | NA | NA |
| rs180692309 | 2 |  |  |  | NA | NA | T/0.999/2.682/1.81e-06 | T/0.998/1.602/0.00032 | NA | NA |
| rs190183456 | 2 |  |  |  | NA | NA | C/1/2.826/2.48e-06 | C/0.999/1.35/0.00428 | NA | NA |
| rs185857144 | 2 | [FLJ16124](http://genome.ucsc.edu/cgi-bin/hgTracks?hgHubConnect.destUrl=..%2Fcgi-bin%2FhgTracks&clade=mammal&org=Human&db=hg19&position=FLJ16124) |  |  | NA | NA | C/1/4.664/1.73e-06 | C/1/2.669/4e-04 | NA | NA |
| rs187013403 | 2 |  | [PLEK (2413)](http://genome.ucsc.edu/cgi-bin/hgTracks?hgHubConnect.destUrl=..%2Fcgi-bin%2FhgTracks&clade=mammal&org=Human&db=hg19&position=PLEK) | [FBXO48 (62506)](http://genome.ucsc.edu/cgi-bin/hgTracks?hgHubConnect.destUrl=..%2Fcgi-bin%2FhgTracks&clade=mammal&org=Human&db=hg19&position=FBXO48) | NA | NA | C/1/1.966/4.11e-06 | C/0.998/0.749/0.02244 | NA | NA |
| rs187803700 | 2 |  | [PLEK (5971)](http://genome.ucsc.edu/cgi-bin/hgTracks?hgHubConnect.destUrl=..%2Fcgi-bin%2FhgTracks&clade=mammal&org=Human&db=hg19&position=PLEK) | [FBXO48 (58948)](http://genome.ucsc.edu/cgi-bin/hgTracks?hgHubConnect.destUrl=..%2Fcgi-bin%2FhgTracks&clade=mammal&org=Human&db=hg19&position=FBXO48) | NA | NA | T/1/1.988/3.47e-06 | T/0.998/0.76/0.021 | NA | NA |
| rs181794654 | 2 |  | [PLEK (14099)](http://genome.ucsc.edu/cgi-bin/hgTracks?hgHubConnect.destUrl=..%2Fcgi-bin%2FhgTracks&clade=mammal&org=Human&db=hg19&position=PLEK) | [FBXO48 (50820)](http://genome.ucsc.edu/cgi-bin/hgTracks?hgHubConnect.destUrl=..%2Fcgi-bin%2FhgTracks&clade=mammal&org=Human&db=hg19&position=FBXO48) | NA | NA | G/1/2.022/2.59e-06 | G/0.998/0.78/0.01849 | NA | NA |
| rs147031024 | 2 |  | [PLEK (14313)](http://genome.ucsc.edu/cgi-bin/hgTracks?hgHubConnect.destUrl=..%2Fcgi-bin%2FhgTracks&clade=mammal&org=Human&db=hg19&position=PLEK) | [FBXO48 (50606)](http://genome.ucsc.edu/cgi-bin/hgTracks?hgHubConnect.destUrl=..%2Fcgi-bin%2FhgTracks&clade=mammal&org=Human&db=hg19&position=FBXO48) | NA | NA | T/1/2.023/2.58e-06 | T/0.998/0.78/0.01841 | NA | NA |
| rs187068710 | 2 |  | [PLEK (15131)](http://genome.ucsc.edu/cgi-bin/hgTracks?hgHubConnect.destUrl=..%2Fcgi-bin%2FhgTracks&clade=mammal&org=Human&db=hg19&position=PLEK) | [FBXO48 (49788)](http://genome.ucsc.edu/cgi-bin/hgTracks?hgHubConnect.destUrl=..%2Fcgi-bin%2FhgTracks&clade=mammal&org=Human&db=hg19&position=FBXO48) | NA | NA | T/1/2.034/2.47e-06 | T/0.998/0.785/0.01813 | NA | NA |
| rs142609709 | 2 |  | [PLEK (17835)](http://genome.ucsc.edu/cgi-bin/hgTracks?hgHubConnect.destUrl=..%2Fcgi-bin%2FhgTracks&clade=mammal&org=Human&db=hg19&position=PLEK) | [FBXO48 (47084)](http://genome.ucsc.edu/cgi-bin/hgTracks?hgHubConnect.destUrl=..%2Fcgi-bin%2FhgTracks&clade=mammal&org=Human&db=hg19&position=FBXO48) | NA | NA | T/1/2.066/2.28e-06 | T/0.998/0.8/0.01741 | NA | NA |
| rs189505973 | 2 |  | [PLEK (22280)](http://genome.ucsc.edu/cgi-bin/hgTracks?hgHubConnect.destUrl=..%2Fcgi-bin%2FhgTracks&clade=mammal&org=Human&db=hg19&position=PLEK) | [FBXO48 (42639)](http://genome.ucsc.edu/cgi-bin/hgTracks?hgHubConnect.destUrl=..%2Fcgi-bin%2FhgTracks&clade=mammal&org=Human&db=hg19&position=FBXO48) | NA | NA | G/1/2.127/1.82e-06 | G/0.998/0.826/0.01594 | NA | NA |
| rs116765889 | 2 |  |  | [CYP26B1 (142809)](http://genome.ucsc.edu/cgi-bin/hgTracks?hgHubConnect.destUrl=..%2Fcgi-bin%2FhgTracks&clade=mammal&org=Human&db=hg19&position=CYP26B1) | A/0.998/0.016/0.928 | A/0.998/-0.002/0.9911 | A/0.999/2.508/3.72e-07 | A/0.999/1.527/0.00047 | A/0.002/-0.298/0.04933 | A/0.002/-0.203/0.1643 |
| rs187133462 | 2 |  |  |  | A/0.999/-0.151/0.5637 | A/0.999/0.8/0.02045 | A/1/3.055/9.87e-08 | A/0.999/1.655/0.00017 | NA | NA |
| rs187203999 | 2 | [LRP1B](http://genome.ucsc.edu/cgi-bin/hgTracks?hgHubConnect.destUrl=..%2Fcgi-bin%2FhgTracks&clade=mammal&org=Human&db=hg19&position=LRP1B) |  |  | T/0.968/-0.013/0.8383 | T/0.966/-0.09/0.2062 | T/0.982/0.455/2.23e-06 | T/0.978/0.415/0.00031 | NA | NA |
| rs111899708 | 2 |  |  |  | A/0.982/0.15/0.01411 | A/0.979/-0.056/0.3859 | A/0.994/0.807/4.76e-06 | A/0.991/0.519/0.01438 | A/0.018/-0.104/0.01853 | A/0.02/0.01/0.8099 |
| rs187076367 | 2 | [BC046497](http://genome.ucsc.edu/cgi-bin/hgTracks?hgHubConnect.destUrl=..%2Fcgi-bin%2FhgTracks&clade=mammal&org=Human&db=hg19&position=BC046497) | [CHRNA1 (7714)](http://genome.ucsc.edu/cgi-bin/hgTracks?hgHubConnect.destUrl=..%2Fcgi-bin%2FhgTracks&clade=mammal&org=Human&db=hg19&position=CHRNA1) | [CHN1 (27127)](http://genome.ucsc.edu/cgi-bin/hgTracks?hgHubConnect.destUrl=..%2Fcgi-bin%2FhgTracks&clade=mammal&org=Human&db=hg19&position=CHN1) | A/0.998/-0.019/0.9046 | A/0.998/-0.146/0.4331 | A/0.999/1.863/1.11e-06 | A/0.998/1.024/0.00069 | A/0.002/-0.19/0.118 | A/0.002/-0.03/0.7873 |
| rs188245701 | 2 | [CHN1](http://genome.ucsc.edu/cgi-bin/hgTracks?hgHubConnect.destUrl=..%2Fcgi-bin%2FhgTracks&clade=mammal&org=Human&db=hg19&position=CHN1) | [BC046497 (212035)](http://genome.ucsc.edu/cgi-bin/hgTracks?hgHubConnect.destUrl=..%2Fcgi-bin%2FhgTracks&clade=mammal&org=Human&db=hg19&position=BC046497) | [AF283776 (85388)](http://genome.ucsc.edu/cgi-bin/hgTracks?hgHubConnect.destUrl=..%2Fcgi-bin%2FhgTracks&clade=mammal&org=Human&db=hg19&position=AF283776) | T/0.992/-0.103/0.3867 | T/0.993/-0.187/0.1875 | T/0.998/1.685/1.84e-07 | T/0.997/1.059/0.00018 | T/0.007/-0.072/0.4346 | T/0.007/0.028/0.756 |
| rs114230339 | 2 | [KIAA1715](http://genome.ucsc.edu/cgi-bin/hgTracks?hgHubConnect.destUrl=..%2Fcgi-bin%2FhgTracks&clade=mammal&org=Human&db=hg19&position=KIAA1715) |  | [EVX2 (85311)](http://genome.ucsc.edu/cgi-bin/hgTracks?hgHubConnect.destUrl=..%2Fcgi-bin%2FhgTracks&clade=mammal&org=Human&db=hg19&position=EVX2) | NA | NA | T/0.966/0.252/4.86e-06 | T/0.954/0.03/0.6772 | NA | NA |
| rs114396821 | 2 | [DIRC3](http://genome.ucsc.edu/cgi-bin/hgTracks?hgHubConnect.destUrl=..%2Fcgi-bin%2FhgTracks&clade=mammal&org=Human&db=hg19&position=DIRC3) |  |  | NA | NA | G/0.995/0.623/2.55e-06 | G/0.992/0.487/0.00148 | NA | NA |
| rs141521978 | 2 | [SP100](http://genome.ucsc.edu/cgi-bin/hgTracks?hgHubConnect.destUrl=..%2Fcgi-bin%2FhgTracks&clade=mammal&org=Human&db=hg19&position=SP100) | [SP140L (43932)](http://genome.ucsc.edu/cgi-bin/hgTracks?hgHubConnect.destUrl=..%2Fcgi-bin%2FhgTracks&clade=mammal&org=Human&db=hg19&position=SP140L) |  | NA | NA | T/0.996/0.831/2.14e-06 | T/0.993/0.408/0.01575 | NA | NA |
| rs184963741 | 2 | [SP100](http://genome.ucsc.edu/cgi-bin/hgTracks?hgHubConnect.destUrl=..%2Fcgi-bin%2FhgTracks&clade=mammal&org=Human&db=hg19&position=SP100) | [SP140L (65041)](http://genome.ucsc.edu/cgi-bin/hgTracks?hgHubConnect.destUrl=..%2Fcgi-bin%2FhgTracks&clade=mammal&org=Human&db=hg19&position=SP140L) | [LOC151475 (222149)](http://genome.ucsc.edu/cgi-bin/hgTracks?hgHubConnect.destUrl=..%2Fcgi-bin%2FhgTracks&clade=mammal&org=Human&db=hg19&position=LOC151475) | NA | NA | C/0.997/0.836/1.69e-06 | C/0.993/0.398/0.01772 | NA | NA |
| rs144060362 | 2 |  | [CXCR7 (92728)](http://genome.ucsc.edu/cgi-bin/hgTracks?hgHubConnect.destUrl=..%2Fcgi-bin%2FhgTracks&clade=mammal&org=Human&db=hg19&position=CXCR7) |  | NA | NA | T/0.999/1.44/2.83e-07 | T/0.997/0.714/0.00322 | NA | NA |
| rs145287362 | 3 |  | [MIR548AC (106788)](http://genome.ucsc.edu/cgi-bin/hgTracks?hgHubConnect.destUrl=..%2Fcgi-bin%2FhgTracks&clade=mammal&org=Human&db=hg19&position=MIR548AC) | [UBE2E1 (91713)](http://genome.ucsc.edu/cgi-bin/hgTracks?hgHubConnect.destUrl=..%2Fcgi-bin%2FhgTracks&clade=mammal&org=Human&db=hg19&position=UBE2E1) | G/0.97/0.038/0.4082 | G/0.971/0.07/0.176 | G/0.995/0.632/2.69e-06 | G/0.99/0.383/0.00691 | A/0.973/0.075/0.03957 | A/0.973/0.043/0.2319 |
| rs187206368 | 3 |  |  | [RBMS3 (241540)](http://genome.ucsc.edu/cgi-bin/hgTracks?hgHubConnect.destUrl=..%2Fcgi-bin%2FhgTracks&clade=mammal&org=Human&db=hg19&position=RBMS3) | A/0.995/0.04/0.7646 | A/0.996/0.158/0.3352 | A/0.997/0.943/4.43e-06 | A/0.994/0.611/0.0055 | A/0.004/-0.204/0.03295 | A/0.005/-0.153/0.1096 |
| rs115082929 | 3 |  | [ZNF621 (217869)](http://genome.ucsc.edu/cgi-bin/hgTracks?hgHubConnect.destUrl=..%2Fcgi-bin%2FhgTracks&clade=mammal&org=Human&db=hg19&position=ZNF621) |  | NA | NA | G/0.996/0.735/1.64e-06 | G/0.992/0.507/0.00361 | NA | NA |
| rs182923613 | 3 |  | [ABHD5 (69006)](http://genome.ucsc.edu/cgi-bin/hgTracks?hgHubConnect.destUrl=..%2Fcgi-bin%2FhgTracks&clade=mammal&org=Human&db=hg19&position=ABHD5) |  | T/0.994/-0.263/0.0056 | T/0.995/-0.036/0.7798 | T/0.998/0.89/1.26e-06 | T/0.995/0.409/0.02011 | A/0.995/-0.017/0.82 | A/0.995/0.054/0.4801 |
| rs182544594 | 3 |  |  |  | NA | NA | G/0.996/0.694/4.02e-06 | G/0.991/0.276/0.05957 | NA | NA |
| rs182242981 | 3 |  |  |  | NA | NA | T/0.996/0.687/3.97e-06 | T/0.991/0.276/0.05656 | NA | NA |
| rs183369465 | 3 | [ROBO2](http://genome.ucsc.edu/cgi-bin/hgTracks?hgHubConnect.destUrl=..%2Fcgi-bin%2FhgTracks&clade=mammal&org=Human&db=hg19&position=ROBO2) |  |  | NA | NA | T/1/4.98/1.51e-06 | T/0.999/2.702/0.00066 | NA | NA |
| rs182019417 | 3 |  |  |  | NA | NA | G/1/4.972/1.61e-06 | G/1/2.433/0.00234 | NA | NA |
| rs181585632 | 3 |  |  |  | NA | NA | C/1/4.957/1.61e-06 | C/1/2.433/0.00225 | NA | NA |
| rs185969863 | 3 |  |  | [HTR1F (204528)](http://genome.ucsc.edu/cgi-bin/hgTracks?hgHubConnect.destUrl=..%2Fcgi-bin%2FhgTracks&clade=mammal&org=Human&db=hg19&position=HTR1F) | NA | NA | A/0.999/1.808/1.88e-06 | A/0.998/0.964/0.00498 | NA | NA |
| rs182728150 | 3 |  |  | [HTR1F (140510)](http://genome.ucsc.edu/cgi-bin/hgTracks?hgHubConnect.destUrl=..%2Fcgi-bin%2FhgTracks&clade=mammal&org=Human&db=hg19&position=HTR1F) | NA | NA | T/1/2.099/3.46e-06 | T/0.999/1.052/0.00257 | NA | NA |
| rs190321959 | 3 | [UROC1](http://genome.ucsc.edu/cgi-bin/hgTracks?hgHubConnect.destUrl=..%2Fcgi-bin%2FhgTracks&clade=mammal&org=Human&db=hg19&position=UROC1) | [ZXDC (28727)](http://genome.ucsc.edu/cgi-bin/hgTracks?hgHubConnect.destUrl=..%2Fcgi-bin%2FhgTracks&clade=mammal&org=Human&db=hg19&position=ZXDC) | [CHST13 (19641)](http://genome.ucsc.edu/cgi-bin/hgTracks?hgHubConnect.destUrl=..%2Fcgi-bin%2FhgTracks&clade=mammal&org=Human&db=hg19&position=CHST13) | NA | NA | T/0.997/0.965/3.24e-07 | T/0.995/0.75/0.00029 | NA | NA |
| rs188764199 | 3 | [TXNRD3NB](http://genome.ucsc.edu/cgi-bin/hgTracks?hgHubConnect.destUrl=..%2Fcgi-bin%2FhgTracks&clade=mammal&org=Human&db=hg19&position=TXNRD3NB) | [C3orf22 (26554)](http://genome.ucsc.edu/cgi-bin/hgTracks?hgHubConnect.destUrl=..%2Fcgi-bin%2FhgTracks&clade=mammal&org=Human&db=hg19&position=C3orf22) | [TXNRD3 (21582)](http://genome.ucsc.edu/cgi-bin/hgTracks?hgHubConnect.destUrl=..%2Fcgi-bin%2FhgTracks&clade=mammal&org=Human&db=hg19&position=TXNRD3) | NA | NA | C/0.997/0.803/3.7e-06 | C/0.995/0.664/0.00062 | NA | NA |
| rs191347135 | 3 | [NUP210P1](http://genome.ucsc.edu/cgi-bin/hgTracks?hgHubConnect.destUrl=..%2Fcgi-bin%2FhgTracks&clade=mammal&org=Human&db=hg19&position=NUP210P1) | [TXNRD3 (9366)](http://genome.ucsc.edu/cgi-bin/hgTracks?hgHubConnect.destUrl=..%2Fcgi-bin%2FhgTracks&clade=mammal&org=Human&db=hg19&position=TXNRD3) | [CHCHD6 (39784)](http://genome.ucsc.edu/cgi-bin/hgTracks?hgHubConnect.destUrl=..%2Fcgi-bin%2FhgTracks&clade=mammal&org=Human&db=hg19&position=CHCHD6) | NA | NA | A/0.998/0.852/1.67e-06 | A/0.996/0.647/0.00094 | NA | NA |
| rs186679854 | 3 |  | [BC015846 (32379)](http://genome.ucsc.edu/cgi-bin/hgTracks?hgHubConnect.destUrl=..%2Fcgi-bin%2FhgTracks&clade=mammal&org=Human&db=hg19&position=BC015846) | [BX537548 (57640)](http://genome.ucsc.edu/cgi-bin/hgTracks?hgHubConnect.destUrl=..%2Fcgi-bin%2FhgTracks&clade=mammal&org=Human&db=hg19&position=BX537548) | NA | NA | A/0.998/0.903/3.4e-06 | A/0.995/0.396/0.03633 | NA | NA |
| rs112448655 | 3 | [CLSTN2](http://genome.ucsc.edu/cgi-bin/hgTracks?hgHubConnect.destUrl=..%2Fcgi-bin%2FhgTracks&clade=mammal&org=Human&db=hg19&position=CLSTN2) |  |  | A/0.99/-0.001/0.993 | A/0.99/0.002/0.9744 | A/0.997/0.777/1.16e-06 | A/0.994/0.516/0.00105 | A/0.01/-0.132/0.00955 | A/0.01/-0.045/0.3497 |
| rs189504960 | 3 |  |  | [ZIC4 (156315)](http://genome.ucsc.edu/cgi-bin/hgTracks?hgHubConnect.destUrl=..%2Fcgi-bin%2FhgTracks&clade=mammal&org=Human&db=hg19&position=ZIC4) | T/0.984/0.009/0.899 | T/0.983/0.054/0.4944 | T/0.946/0.204/3.38e-06 | T/0.936/0.124/0.03785 | A/0.959/0.128/0.00029 | A/0.96/0.091/0.0302 |
| rs142900875 | 3 |  |  |  | T/0.994/-0.028/0.7854 | T/0.993/-0.135/0.2437 | T/0.999/2.257/9.09e-08 | T/0.998/1.326/0.00136 | T/0.006/-0.174/0.02878 | T/0.007/-0.075/0.3145 |
| rs181840477 | 3 |  |  |  | T/0.994/-0.04/0.6861 | T/0.993/-0.131/0.2434 | T/0.999/2.235/5.16e-08 | T/0.999/1.325/0.00119 | T/0.006/-0.16/0.0385 | T/0.007/-0.07/0.3331 |
| rs185832426 | 3 |  |  |  | G/0.993/-0.039/0.6843 | G/0.993/-0.172/0.1133 | G/0.999/2.231/4.66e-08 | G/0.999/1.325/0.00116 | T/0.993/0.152/0.0425 | T/0.993/0.049/0.4913 |
| rs114239771 | 3 |  |  |  | A/0.993/-0.032/0.7023 | A/0.993/-0.17/0.07637 | A/0.999/2.157/2.66e-08 | A/0.999/1.298/0.00108 | A/0.007/-0.133/0.05353 | A/0.007/-0.026/0.6905 |
| rs148200394 | 3 |  |  |  | T/0.993/-0.033/0.6921 | T/0.993/-0.171/0.07095 | T/0.999/2.133/2.11e-08 | T/0.999/1.286/0.00107 | A/0.993/0.13/0.05378 | A/0.993/0.024/0.7175 |
| rs140910939 | 3 |  |  |  | T/0.997/0.102/0.5072 | T/0.997/-0.154/0.3514 | T/1/2.096/2.17e-07 | T/0.999/1.346/0.00293 | NA | NA |
| rs149843939 | 3 |  |  |  | C/0.996/0.026/0.8481 | C/0.996/-0.202/0.1799 | C/0.999/1.986/8.63e-08 | C/0.998/1.35/0.00155 | NA | NA |
| rs139873132 | 3 |  |  |  | NA | NA | T/0.984/-0.337/1.41e-06 | T/0.991/0.037/0.7978 | NA | NA |
| rs191765304 | 3 |  | [TRIM59 (8743)](http://genome.ucsc.edu/cgi-bin/hgTracks?hgHubConnect.destUrl=..%2Fcgi-bin%2FhgTracks&clade=mammal&org=Human&db=hg19&position=TRIM59) | [KPNA4 (36413)](http://genome.ucsc.edu/cgi-bin/hgTracks?hgHubConnect.destUrl=..%2Fcgi-bin%2FhgTracks&clade=mammal&org=Human&db=hg19&position=KPNA4) | NA | NA | T/1/3.4/5.36e-07 | T/0.999/1.591/0.00236 | NA | NA |
| rs188694422 | 3 |  | [TRIM59 (8891)](http://genome.ucsc.edu/cgi-bin/hgTracks?hgHubConnect.destUrl=..%2Fcgi-bin%2FhgTracks&clade=mammal&org=Human&db=hg19&position=TRIM59) | [KPNA4 (36265)](http://genome.ucsc.edu/cgi-bin/hgTracks?hgHubConnect.destUrl=..%2Fcgi-bin%2FhgTracks&clade=mammal&org=Human&db=hg19&position=KPNA4) | NA | NA | A/1/3.398/5.33e-07 | A/0.999/1.59/0.00236 | NA | NA |
| rs190230151 | 3 | [KPNA4](http://genome.ucsc.edu/cgi-bin/hgTracks?hgHubConnect.destUrl=..%2Fcgi-bin%2FhgTracks&clade=mammal&org=Human&db=hg19&position=KPNA4) | [SCARNA7 (13682)](http://genome.ucsc.edu/cgi-bin/hgTracks?hgHubConnect.destUrl=..%2Fcgi-bin%2FhgTracks&clade=mammal&org=Human&db=hg19&position=SCARNA7) | [BC125159 (38750)](http://genome.ucsc.edu/cgi-bin/hgTracks?hgHubConnect.destUrl=..%2Fcgi-bin%2FhgTracks&clade=mammal&org=Human&db=hg19&position=BC125159) | NA | NA | G/1/3.075/5.58e-07 | G/0.999/1.457/0.00209 | NA | NA |
| rs190431552 | 3 |  | [ARL14 (23111)](http://genome.ucsc.edu/cgi-bin/hgTracks?hgHubConnect.destUrl=..%2Fcgi-bin%2FhgTracks&clade=mammal&org=Human&db=hg19&position=ARL14) | [PPM1L (54649)](http://genome.ucsc.edu/cgi-bin/hgTracks?hgHubConnect.destUrl=..%2Fcgi-bin%2FhgTracks&clade=mammal&org=Human&db=hg19&position=PPM1L) | NA | NA | G/1/2.422/7e-07 | G/0.999/1.169/0.00188 | NA | NA |
| rs146844429 | 3 |  | [ARL14 (75520)](http://genome.ucsc.edu/cgi-bin/hgTracks?hgHubConnect.destUrl=..%2Fcgi-bin%2FhgTracks&clade=mammal&org=Human&db=hg19&position=ARL14) | [PPM1L (2240)](http://genome.ucsc.edu/cgi-bin/hgTracks?hgHubConnect.destUrl=..%2Fcgi-bin%2FhgTracks&clade=mammal&org=Human&db=hg19&position=PPM1L) | NA | NA | G/0.999/2.36/3.41e-07 | G/0.998/1.04/0.00372 | NA | NA |
| rs186451686 | 3 | [PPM1L](http://genome.ucsc.edu/cgi-bin/hgTracks?hgHubConnect.destUrl=..%2Fcgi-bin%2FhgTracks&clade=mammal&org=Human&db=hg19&position=PPM1L) |  | [AK055323 (239084)](http://genome.ucsc.edu/cgi-bin/hgTracks?hgHubConnect.destUrl=..%2Fcgi-bin%2FhgTracks&clade=mammal&org=Human&db=hg19&position=AK055323) | NA | NA | A/1/2.398/7e-07 | A/0.998/1.153/0.00195 | NA | NA |
| rs149794616 | 3 | [PPM1L](http://genome.ucsc.edu/cgi-bin/hgTracks?hgHubConnect.destUrl=..%2Fcgi-bin%2FhgTracks&clade=mammal&org=Human&db=hg19&position=PPM1L) |  | [AK055323 (237576)](http://genome.ucsc.edu/cgi-bin/hgTracks?hgHubConnect.destUrl=..%2Fcgi-bin%2FhgTracks&clade=mammal&org=Human&db=hg19&position=AK055323) | NA | NA | C/0.999/2.324/5.55e-07 | C/0.998/1.033/0.00402 | NA | NA |
| rs188531091 | 3 | [PPM1L](http://genome.ucsc.edu/cgi-bin/hgTracks?hgHubConnect.destUrl=..%2Fcgi-bin%2FhgTracks&clade=mammal&org=Human&db=hg19&position=PPM1L) |  | [AK055323 (230124)](http://genome.ucsc.edu/cgi-bin/hgTracks?hgHubConnect.destUrl=..%2Fcgi-bin%2FhgTracks&clade=mammal&org=Human&db=hg19&position=AK055323) | NA | NA | G/1/2.397/7.02e-07 | G/0.998/1.153/0.00195 | NA | NA |
| rs150391545 | 3 | [PPM1L](http://genome.ucsc.edu/cgi-bin/hgTracks?hgHubConnect.destUrl=..%2Fcgi-bin%2FhgTracks&clade=mammal&org=Human&db=hg19&position=PPM1L) |  | [AK055323 (211003)](http://genome.ucsc.edu/cgi-bin/hgTracks?hgHubConnect.destUrl=..%2Fcgi-bin%2FhgTracks&clade=mammal&org=Human&db=hg19&position=AK055323) | NA | NA | G/1/2.295/1.7e-06 | G/0.998/1.152/0.00191 | NA | NA |
| rs182275499 | 3 | [PPM1L](http://genome.ucsc.edu/cgi-bin/hgTracks?hgHubConnect.destUrl=..%2Fcgi-bin%2FhgTracks&clade=mammal&org=Human&db=hg19&position=PPM1L) |  | [AK055323 (201945)](http://genome.ucsc.edu/cgi-bin/hgTracks?hgHubConnect.destUrl=..%2Fcgi-bin%2FhgTracks&clade=mammal&org=Human&db=hg19&position=AK055323) | NA | NA | G/0.999/2.294/1.41e-06 | G/0.998/1.164/0.00169 | NA | NA |
| rs182750324 | 3 | [PPM1L](http://genome.ucsc.edu/cgi-bin/hgTracks?hgHubConnect.destUrl=..%2Fcgi-bin%2FhgTracks&clade=mammal&org=Human&db=hg19&position=PPM1L) |  | [AK055323 (190782)](http://genome.ucsc.edu/cgi-bin/hgTracks?hgHubConnect.destUrl=..%2Fcgi-bin%2FhgTracks&clade=mammal&org=Human&db=hg19&position=AK055323) | NA | NA | A/1/2.373/8.24e-07 | A/0.999/1.149/0.00194 | NA | NA |
| rs145882615 | 3 | [PPM1L](http://genome.ucsc.edu/cgi-bin/hgTracks?hgHubConnect.destUrl=..%2Fcgi-bin%2FhgTracks&clade=mammal&org=Human&db=hg19&position=PPM1L) |  | [AK055323 (75653)](http://genome.ucsc.edu/cgi-bin/hgTracks?hgHubConnect.destUrl=..%2Fcgi-bin%2FhgTracks&clade=mammal&org=Human&db=hg19&position=AK055323) | NA | NA | G/1/2.216/3.06e-06 | G/0.998/1.126/0.00221 | NA | NA |
| rs191390172 | 3 | [PPM1L](http://genome.ucsc.edu/cgi-bin/hgTracks?hgHubConnect.destUrl=..%2Fcgi-bin%2FhgTracks&clade=mammal&org=Human&db=hg19&position=PPM1L) |  | [AK055323 (27057)](http://genome.ucsc.edu/cgi-bin/hgTracks?hgHubConnect.destUrl=..%2Fcgi-bin%2FhgTracks&clade=mammal&org=Human&db=hg19&position=AK055323) | NA | NA | C/1/2.22/2.94e-06 | C/0.998/1.127/0.00218 | NA | NA |
| rs192294496 | 3 |  | [B3GALNT1 (8414)](http://genome.ucsc.edu/cgi-bin/hgTracks?hgHubConnect.destUrl=..%2Fcgi-bin%2FhgTracks&clade=mammal&org=Human&db=hg19&position=B3GALNT1) | [NMD3 (107524)](http://genome.ucsc.edu/cgi-bin/hgTracks?hgHubConnect.destUrl=..%2Fcgi-bin%2FhgTracks&clade=mammal&org=Human&db=hg19&position=NMD3) | NA | NA | T/1/2.389/7.77e-07 | T/0.999/1.149/0.00204 | NA | NA |
| rs187303806 | 3 |  | [B3GALNT1 (58315)](http://genome.ucsc.edu/cgi-bin/hgTracks?hgHubConnect.destUrl=..%2Fcgi-bin%2FhgTracks&clade=mammal&org=Human&db=hg19&position=B3GALNT1) | [NMD3 (57623)](http://genome.ucsc.edu/cgi-bin/hgTracks?hgHubConnect.destUrl=..%2Fcgi-bin%2FhgTracks&clade=mammal&org=Human&db=hg19&position=NMD3) | NA | NA | C/1/2.295/1.34e-06 | C/0.998/1.109/0.00252 | NA | NA |
| rs140014224 | 3 | [NMD3](http://genome.ucsc.edu/cgi-bin/hgTracks?hgHubConnect.destUrl=..%2Fcgi-bin%2FhgTracks&clade=mammal&org=Human&db=hg19&position=NMD3) | [B3GALNT1 (133614)](http://genome.ucsc.edu/cgi-bin/hgTracks?hgHubConnect.destUrl=..%2Fcgi-bin%2FhgTracks&clade=mammal&org=Human&db=hg19&position=B3GALNT1) | [SPTSSB (105805)](http://genome.ucsc.edu/cgi-bin/hgTracks?hgHubConnect.destUrl=..%2Fcgi-bin%2FhgTracks&clade=mammal&org=Human&db=hg19&position=SPTSSB) | NA | NA | G/1/2.355/8.01e-07 | G/0.999/1.135/0.00217 | NA | NA |
| rs142560558 | 3 |  | [NMD3 (69887)](http://genome.ucsc.edu/cgi-bin/hgTracks?hgHubConnect.destUrl=..%2Fcgi-bin%2FhgTracks&clade=mammal&org=Human&db=hg19&position=NMD3) | [SPTSSB (21372)](http://genome.ucsc.edu/cgi-bin/hgTracks?hgHubConnect.destUrl=..%2Fcgi-bin%2FhgTracks&clade=mammal&org=Human&db=hg19&position=SPTSSB) | NA | NA | G/1/2.349/8.59e-07 | G/0.998/1.104/0.00301 | NA | NA |
| rs192376484 | 3 |  | [NMD3 (84495)](http://genome.ucsc.edu/cgi-bin/hgTracks?hgHubConnect.destUrl=..%2Fcgi-bin%2FhgTracks&clade=mammal&org=Human&db=hg19&position=NMD3) | [SPTSSB (6764)](http://genome.ucsc.edu/cgi-bin/hgTracks?hgHubConnect.destUrl=..%2Fcgi-bin%2FhgTracks&clade=mammal&org=Human&db=hg19&position=SPTSSB) | NA | NA | A/1/2.355/5.38e-07 | A/0.998/1.077/0.00297 | NA | NA |
| rs190957092 | 3 |  |  |  | C/0.997/-0.123/0.5241 | C/0.997/-0.247/0.2571 | C/0.999/2.21/8.82e-08 | C/0.998/0.759/0.0198 | T/0.997/0.101/0.4578 | T/0.997/0.001/0.9955 |
| rs145669774 | 3 |  | [IGF2BP2 (2675)](http://genome.ucsc.edu/cgi-bin/hgTracks?hgHubConnect.destUrl=..%2Fcgi-bin%2FhgTracks&clade=mammal&org=Human&db=hg19&position=IGF2BP2) | [TRA2B (86855)](http://genome.ucsc.edu/cgi-bin/hgTracks?hgHubConnect.destUrl=..%2Fcgi-bin%2FhgTracks&clade=mammal&org=Human&db=hg19&position=TRA2B) | A/0.986/0.027/0.6936 | A/0.987/0.107/0.1679 | A/0.996/0.689/1.78e-06 | A/0.992/0.456/0.00131 | A/0.013/-0.08/0.1187 | A/0.014/-0.089/0.06619 |
| rs147922770 | 3 |  | [IGF2BP2 (46114)](http://genome.ucsc.edu/cgi-bin/hgTracks?hgHubConnect.destUrl=..%2Fcgi-bin%2FhgTracks&clade=mammal&org=Human&db=hg19&position=IGF2BP2) | [TRA2B (43416)](http://genome.ucsc.edu/cgi-bin/hgTracks?hgHubConnect.destUrl=..%2Fcgi-bin%2FhgTracks&clade=mammal&org=Human&db=hg19&position=TRA2B) | G/0.978/-0.033/0.6044 | G/0.978/-0.012/0.8634 | G/0.994/0.712/1.24e-06 | G/0.989/0.452/0.00152 | C/0.98/0.064/0.1925 | C/0.979/0.028/0.549 |
| rs114946066 | 3 | [TRA2B](http://genome.ucsc.edu/cgi-bin/hgTracks?hgHubConnect.destUrl=..%2Fcgi-bin%2FhgTracks&clade=mammal&org=Human&db=hg19&position=TRA2B) | [IGF2BP2 (110270)](http://genome.ucsc.edu/cgi-bin/hgTracks?hgHubConnect.destUrl=..%2Fcgi-bin%2FhgTracks&clade=mammal&org=Human&db=hg19&position=IGF2BP2) | [LOC344887 (24660)](http://genome.ucsc.edu/cgi-bin/hgTracks?hgHubConnect.destUrl=..%2Fcgi-bin%2FhgTracks&clade=mammal&org=Human&db=hg19&position=LOC344887) | C/0.976/-0.032/0.5997 | C/0.978/0.055/0.4544 | C/0.993/0.683/1.21e-06 | C/0.988/0.427/0.00137 | T/0.979/0.074/0.1153 | T/0.978/0.062/0.18 |
| rs150450027 | 4 | [LOC152742](http://genome.ucsc.edu/cgi-bin/hgTracks?hgHubConnect.destUrl=..%2Fcgi-bin%2FhgTracks&clade=mammal&org=Human&db=hg19&position=LOC152742) |  |  | NA | NA | T/1/2.105/2.51e-07 | T/0.998/1.158/0.00022 | NA | NA |
| rs115971165 | 4 |  | [ANAPC4 (241986)](http://genome.ucsc.edu/cgi-bin/hgTracks?hgHubConnect.destUrl=..%2Fcgi-bin%2FhgTracks&clade=mammal&org=Human&db=hg19&position=ANAPC4) | [SLC34A2 (17062)](http://genome.ucsc.edu/cgi-bin/hgTracks?hgHubConnect.destUrl=..%2Fcgi-bin%2FhgTracks&clade=mammal&org=Human&db=hg19&position=SLC34A2) | NA | NA | T/0.985/0.415/7.82e-07 | T/0.982/0.35/0.00206 | NA | NA |
| rs189578969 | 4 |  |  | [COX7B2 (4556)](http://genome.ucsc.edu/cgi-bin/hgTracks?hgHubConnect.destUrl=..%2Fcgi-bin%2FhgTracks&clade=mammal&org=Human&db=hg19&position=COX7B2) | T/0.996/0.146/0.2333 | T/0.995/0.16/0.212 | T/0.999/1.509/4.98e-06 | T/0.998/0.732/0.00695 | T/0.005/-0.22/0.009 | T/0.006/-0.187/0.01135 |
| rs193022444 | 4 | [COX7B2](http://genome.ucsc.edu/cgi-bin/hgTracks?hgHubConnect.destUrl=..%2Fcgi-bin%2FhgTracks&clade=mammal&org=Human&db=hg19&position=COX7B2) |  | [GABRA4 (151540)](http://genome.ucsc.edu/cgi-bin/hgTracks?hgHubConnect.destUrl=..%2Fcgi-bin%2FhgTracks&clade=mammal&org=Human&db=hg19&position=GABRA4) | C/0.996/0.15/0.2411 | C/0.995/0.165/0.2187 | C/0.999/1.592/3.94e-06 | C/0.998/0.776/0.00689 | T/0.995/0.221/0.01214 | T/0.994/0.186/0.0161 |
| rs142210607 | 4 |  | [KIT (144813)](http://genome.ucsc.edu/cgi-bin/hgTracks?hgHubConnect.destUrl=..%2Fcgi-bin%2FhgTracks&clade=mammal&org=Human&db=hg19&position=KIT) | [KDR (192731)](http://genome.ucsc.edu/cgi-bin/hgTracks?hgHubConnect.destUrl=..%2Fcgi-bin%2FhgTracks&clade=mammal&org=Human&db=hg19&position=KDR) | NA | NA | T/1/3.918/1.5e-06 | T/1/1.888/0.016 | NA | NA |
| rs144575900 | 4 |  |  |  | NA | NA | G/0.991/-0.469/2.68e-06 | G/0.997/0.02/0.9415 | NA | NA |
| rs4865309 | 4 |  |  |  | G/0.799/0.011/0.5058 | G/0.8/-0.017/0.3713 | G/0.76/-0.1/3.65e-06 | G/0.772/-0.078/0.01863 | A/0.79/-0.026/0.0259 | A/0.799/-0.015/0.2261 |
| rs28875200 | 4 | [FAM13A](http://genome.ucsc.edu/cgi-bin/hgTracks?hgHubConnect.destUrl=..%2Fcgi-bin%2FhgTracks&clade=mammal&org=Human&db=hg19&position=FAM13A) |  | [TIGD2 (6039)](http://genome.ucsc.edu/cgi-bin/hgTracks?hgHubConnect.destUrl=..%2Fcgi-bin%2FhgTracks&clade=mammal&org=Human&db=hg19&position=TIGD2) | NA | NA | A/0.742/0.084/4.12e-06 | A/0.71/0.026/0.3176 | NA | NA |
| rs191279589 | 4 |  | [AK094561 (8652)](http://genome.ucsc.edu/cgi-bin/hgTracks?hgHubConnect.destUrl=..%2Fcgi-bin%2FhgTracks&clade=mammal&org=Human&db=hg19&position=AK094561) |  | T/0.995/-0.125/0.3345 | T/0.996/0.081/0.6524 | T/0.999/1.544/3.08e-06 | T/0.999/1.056/0.00347 | A/0.995/0.003/0.9782 | A/0.995/-0.102/0.3086 |
| rs191097752 | 4 | [TET2](http://genome.ucsc.edu/cgi-bin/hgTracks?hgHubConnect.destUrl=..%2Fcgi-bin%2FhgTracks&clade=mammal&org=Human&db=hg19&position=TET2) |  | [PPA2 (187242)](http://genome.ucsc.edu/cgi-bin/hgTracks?hgHubConnect.destUrl=..%2Fcgi-bin%2FhgTracks&clade=mammal&org=Human&db=hg19&position=PPA2) | C/0.997/0.02/0.8775 | C/0.997/0.208/0.2127 | C/0.999/1.539/4.63e-06 | C/0.999/1.067/0.00367 | T/0.997/0.138/0.1516 | T/0.996/0.021/0.8243 |
| rs1512973 | 4 |  | [IL2 (128406)](http://genome.ucsc.edu/cgi-bin/hgTracks?hgHubConnect.destUrl=..%2Fcgi-bin%2FhgTracks&clade=mammal&org=Human&db=hg19&position=IL2) | [IL21 (27726)](http://genome.ucsc.edu/cgi-bin/hgTracks?hgHubConnect.destUrl=..%2Fcgi-bin%2FhgTracks&clade=mammal&org=Human&db=hg19&position=IL21) | G/0.339/-0.004/0.7694 | G/0.339/-0.004/0.8059 | G/0.4/-0.073/4.89e-06 | G/0.4/-0.069/0.00434 | A/0.562/-0.024/0.00738 | A/0.502/-0.007/0.434 |
| rs13103766 | 4 |  | [IL2 (139583)](http://genome.ucsc.edu/cgi-bin/hgTracks?hgHubConnect.destUrl=..%2Fcgi-bin%2FhgTracks&clade=mammal&org=Human&db=hg19&position=IL2) | [IL21 (16549)](http://genome.ucsc.edu/cgi-bin/hgTracks?hgHubConnect.destUrl=..%2Fcgi-bin%2FhgTracks&clade=mammal&org=Human&db=hg19&position=IL21) | A/0.66/0.004/0.7856 | A/0.659/0.003/0.8237 | A/0.596/0.076/2.87e-06 | A/0.567/0.071/0.00401 | A/0.361/-0.024/0.00709 | A/0.356/-0.007/0.4626 |
| rs141644443 | 4 |  | [TTC29 (109690)](http://genome.ucsc.edu/cgi-bin/hgTracks?hgHubConnect.destUrl=..%2Fcgi-bin%2FhgTracks&clade=mammal&org=Human&db=hg19&position=TTC29) |  | NA | NA | G/1/2.399/2.55e-06 | G/0.999/1.203/0.00223 | NA | NA |
| rs151121608 | 5 |  | [IPO11 (211578)](http://genome.ucsc.edu/cgi-bin/hgTracks?hgHubConnect.destUrl=..%2Fcgi-bin%2FhgTracks&clade=mammal&org=Human&db=hg19&position=IPO11) |  | NA | NA | G/0.982/-0.335/7.56e-07 | G/0.991/-0.034/0.8321 | NA | NA |
| rs189845032 | 5 | [MAST4](http://genome.ucsc.edu/cgi-bin/hgTracks?hgHubConnect.destUrl=..%2Fcgi-bin%2FhgTracks&clade=mammal&org=Human&db=hg19&position=MAST4) |  | [BC080587 (87783)](http://genome.ucsc.edu/cgi-bin/hgTracks?hgHubConnect.destUrl=..%2Fcgi-bin%2FhgTracks&clade=mammal&org=Human&db=hg19&position=BC080587) | NA | NA | A/0.997/0.76/3.69e-06 | A/0.994/0.518/0.00267 | NA | NA |
| rs192514502 | 5 | [MCTP1](http://genome.ucsc.edu/cgi-bin/hgTracks?hgHubConnect.destUrl=..%2Fcgi-bin%2FhgTracks&clade=mammal&org=Human&db=hg19&position=MCTP1) | [ANKRD32 (243842)](http://genome.ucsc.edu/cgi-bin/hgTracks?hgHubConnect.destUrl=..%2Fcgi-bin%2FhgTracks&clade=mammal&org=Human&db=hg19&position=ANKRD32) |  | T/1/0.721/0.1441 | T/1/0.545/0.1498 | T/0.999/2.597/4.32e-07 | T/0.998/1.539/0.00015 | A/1/1.183/0.00012 | A/0.999/0.784/0.00114 |
| rs141026162 | 5 |  |  | [RGMB (118369)](http://genome.ucsc.edu/cgi-bin/hgTracks?hgHubConnect.destUrl=..%2Fcgi-bin%2FhgTracks&clade=mammal&org=Human&db=hg19&position=RGMB) | NA | NA | C/0.998/1.115/3.37e-06 | C/0.996/0.707/0.00469 | NA | NA |
| rs143404520 | 5 | [SPOCK1](http://genome.ucsc.edu/cgi-bin/hgTracks?hgHubConnect.destUrl=..%2Fcgi-bin%2FhgTracks&clade=mammal&org=Human&db=hg19&position=SPOCK1) |  |  | NA | NA | T/0.999/1.382/9.34e-07 | T/0.997/0.909/0.00039 | NA | NA |
| rs185914258 | 6 |  | [FOXQ1 (802)](http://genome.ucsc.edu/cgi-bin/hgTracks?hgHubConnect.destUrl=..%2Fcgi-bin%2FhgTracks&clade=mammal&org=Human&db=hg19&position=FOXQ1) | [FOXF2 (74273)](http://genome.ucsc.edu/cgi-bin/hgTracks?hgHubConnect.destUrl=..%2Fcgi-bin%2FhgTracks&clade=mammal&org=Human&db=hg19&position=FOXF2) | C/0.999/0.359/0.3305 | C/0.999/0.861/0.05279 | C/1/3.495/4.19e-06 | C/0.999/2.46/0.00075 | NA | NA |
| rs147846168 | 6 |  | [FOXQ1 (25749)](http://genome.ucsc.edu/cgi-bin/hgTracks?hgHubConnect.destUrl=..%2Fcgi-bin%2FhgTracks&clade=mammal&org=Human&db=hg19&position=FOXQ1) | [FOXF2 (49326)](http://genome.ucsc.edu/cgi-bin/hgTracks?hgHubConnect.destUrl=..%2Fcgi-bin%2FhgTracks&clade=mammal&org=Human&db=hg19&position=FOXF2) | NA | NA | A/1/3.109/1.05e-08 | A/0.999/2.496/0.0013 | NA | NA |
| rs187885921 | 6 |  | [FAM50B (42764)](http://genome.ucsc.edu/cgi-bin/hgTracks?hgHubConnect.destUrl=..%2Fcgi-bin%2FhgTracks&clade=mammal&org=Human&db=hg19&position=FAM50B) | [PRPF4B (127253)](http://genome.ucsc.edu/cgi-bin/hgTracks?hgHubConnect.destUrl=..%2Fcgi-bin%2FhgTracks&clade=mammal&org=Human&db=hg19&position=PRPF4B) | NA | NA | C/0.999/1.821/4.51e-06 | C/0.998/1.459/0.00016 | NA | NA |
| rs115144339 | 6 |  |  | [SLC35B3 (29816)](http://genome.ucsc.edu/cgi-bin/hgTracks?hgHubConnect.destUrl=..%2Fcgi-bin%2FhgTracks&clade=mammal&org=Human&db=hg19&position=SLC35B3) | T/0.991/0.122/0.2072 | T/0.991/0.15/0.1809 | T/0.997/1.063/1.28e-06 | T/0.995/0.623/0.00396 | T/0.015/-0.097/0.0806 | T/0.015/-0.096/0.06182 |
| rs184686040 | 6 | [LOC100506207](http://genome.ucsc.edu/cgi-bin/hgTracks?hgHubConnect.destUrl=..%2Fcgi-bin%2FhgTracks&clade=mammal&org=Human&db=hg19&position=LOC100506207) | [SLC35B3 (15269)](http://genome.ucsc.edu/cgi-bin/hgTracks?hgHubConnect.destUrl=..%2Fcgi-bin%2FhgTracks&clade=mammal&org=Human&db=hg19&position=SLC35B3) |  | G/0.992/0.092/0.3888 | G/0.993/0.138/0.2713 | G/0.998/1.222/7.92e-07 | G/0.996/0.694/0.00341 | T/0.986/0.082/0.1642 | T/0.986/0.081/0.1383 |
| rs182482821 | 6 | [LOC100506207](http://genome.ucsc.edu/cgi-bin/hgTracks?hgHubConnect.destUrl=..%2Fcgi-bin%2FhgTracks&clade=mammal&org=Human&db=hg19&position=LOC100506207) | [SLC35B3 (100638)](http://genome.ucsc.edu/cgi-bin/hgTracks?hgHubConnect.destUrl=..%2Fcgi-bin%2FhgTracks&clade=mammal&org=Human&db=hg19&position=SLC35B3) |  | C/0.994/0.108/0.3348 | C/0.994/0.168/0.2011 | C/0.999/1.637/1.17e-06 | C/0.998/0.806/0.0086 | C/0.009/-0.074/0.3095 | C/0.009/-0.068/0.3371 |
| rs185751587 | 6 |  | [LOC100506207 (30817)](http://genome.ucsc.edu/cgi-bin/hgTracks?hgHubConnect.destUrl=..%2Fcgi-bin%2FhgTracks&clade=mammal&org=Human&db=hg19&position=LOC100506207) |  | A/0.993/0.166/0.09649 | A/0.993/0.215/0.06283 | A/0.999/1.575/8.33e-07 | A/0.997/0.745/0.01019 | A/0.009/-0.105/0.1233 | A/0.009/-0.073/0.2664 |
| rs146939578 | 6 |  | [TMEM200A (82195)](http://genome.ucsc.edu/cgi-bin/hgTracks?hgHubConnect.destUrl=..%2Fcgi-bin%2FhgTracks&clade=mammal&org=Human&db=hg19&position=TMEM200A) |  | NA | NA | A/0.991/0.41/3.67e-06 | A/0.986/0.151/0.1553 | NA | NA |
| rs143997509 | 6 |  | [TMEM200A (82532)](http://genome.ucsc.edu/cgi-bin/hgTracks?hgHubConnect.destUrl=..%2Fcgi-bin%2FhgTracks&clade=mammal&org=Human&db=hg19&position=TMEM200A) |  | NA | NA | C/0.991/0.411/3.61e-06 | C/0.986/0.152/0.1545 | NA | NA |
| rs189211803 | 6 |  | [TMEM200A (116314)](http://genome.ucsc.edu/cgi-bin/hgTracks?hgHubConnect.destUrl=..%2Fcgi-bin%2FhgTracks&clade=mammal&org=Human&db=hg19&position=TMEM200A) |  | NA | NA | C/0.995/0.557/3.26e-06 | C/0.992/0.17/0.222 | NA | NA |
| rs184241568 | 6 | [BC041459](http://genome.ucsc.edu/cgi-bin/hgTracks?hgHubConnect.destUrl=..%2Fcgi-bin%2FhgTracks&clade=mammal&org=Human&db=hg19&position=BC041459) |  | [TCF21 (33630)](http://genome.ucsc.edu/cgi-bin/hgTracks?hgHubConnect.destUrl=..%2Fcgi-bin%2FhgTracks&clade=mammal&org=Human&db=hg19&position=TCF21) | NA | NA | G/0.998/1.266/2.5e-06 | G/0.996/0.771/0.00253 | NA | NA |
| rs147990222 | 6 | [BC041459](http://genome.ucsc.edu/cgi-bin/hgTracks?hgHubConnect.destUrl=..%2Fcgi-bin%2FhgTracks&clade=mammal&org=Human&db=hg19&position=BC041459) |  | [TCF21 (16790)](http://genome.ucsc.edu/cgi-bin/hgTracks?hgHubConnect.destUrl=..%2Fcgi-bin%2FhgTracks&clade=mammal&org=Human&db=hg19&position=TCF21) | NA | NA | C/0.999/1.632/2.95e-06 | C/0.997/0.728/0.00785 | NA | NA |
| rs77295064 | 6 |  | [RAET1L (21657)](http://genome.ucsc.edu/cgi-bin/hgTracks?hgHubConnect.destUrl=..%2Fcgi-bin%2FhgTracks&clade=mammal&org=Human&db=hg19&position=RAET1L) | [ULBP3 (15015)](http://genome.ucsc.edu/cgi-bin/hgTracks?hgHubConnect.destUrl=..%2Fcgi-bin%2FhgTracks&clade=mammal&org=Human&db=hg19&position=ULBP3) | T/0.976/-0.042/0.3679 | T/0.974/-0.013/0.7961 | T/0.988/0.439/2.46e-06 | T/0.984/0.326/0.00599 | T/0.025/0.011/0.7365 | T/0.027/0.018/0.5677 |
| rs59071593 | 6 | [SYNE1](http://genome.ucsc.edu/cgi-bin/hgTracks?hgHubConnect.destUrl=..%2Fcgi-bin%2FhgTracks&clade=mammal&org=Human&db=hg19&position=SYNE1) | [ESR1 (216978)](http://genome.ucsc.edu/cgi-bin/hgTracks?hgHubConnect.destUrl=..%2Fcgi-bin%2FhgTracks&clade=mammal&org=Human&db=hg19&position=ESR1) |  | NA | NA | T/0.845/0.107/8.83e-07 | T/0.817/0.034/0.2883 | NA | NA |
| rs75556600 | 6 | [SYNE1](http://genome.ucsc.edu/cgi-bin/hgTracks?hgHubConnect.destUrl=..%2Fcgi-bin%2FhgTracks&clade=mammal&org=Human&db=hg19&position=SYNE1) | [ESR1 (218426)](http://genome.ucsc.edu/cgi-bin/hgTracks?hgHubConnect.destUrl=..%2Fcgi-bin%2FhgTracks&clade=mammal&org=Human&db=hg19&position=ESR1) |  | NA | NA | T/0.862/0.114/1.47e-06 | T/0.838/0.037/0.2889 | NA | NA |
| rs17082463 | 6 | [SYNE1](http://genome.ucsc.edu/cgi-bin/hgTracks?hgHubConnect.destUrl=..%2Fcgi-bin%2FhgTracks&clade=mammal&org=Human&db=hg19&position=SYNE1) | [ESR1 (221335)](http://genome.ucsc.edu/cgi-bin/hgTracks?hgHubConnect.destUrl=..%2Fcgi-bin%2FhgTracks&clade=mammal&org=Human&db=hg19&position=ESR1) |  | NA | NA | G/0.844/0.106/8.09e-07 | G/0.816/0.035/0.2602 | NA | NA |
| rs7741183 | 6 | [SYNE1](http://genome.ucsc.edu/cgi-bin/hgTracks?hgHubConnect.destUrl=..%2Fcgi-bin%2FhgTracks&clade=mammal&org=Human&db=hg19&position=SYNE1) | [ESR1 (221600)](http://genome.ucsc.edu/cgi-bin/hgTracks?hgHubConnect.destUrl=..%2Fcgi-bin%2FhgTracks&clade=mammal&org=Human&db=hg19&position=ESR1) |  | C/0.028/0.021/0.5743 | C/0.028/-0.026/0.5526 | C/0.162/-0.108/3.7e-07 | C/0.162/-0.039/0.2123 | T/0.807/-0.07/8.59e-05 | T/0.705/-0.032/0.1682 |
| rs73783833 | 6 | [SYNE1](http://genome.ucsc.edu/cgi-bin/hgTracks?hgHubConnect.destUrl=..%2Fcgi-bin%2FhgTracks&clade=mammal&org=Human&db=hg19&position=SYNE1) | [ESR1 (223779)](http://genome.ucsc.edu/cgi-bin/hgTracks?hgHubConnect.destUrl=..%2Fcgi-bin%2FhgTracks&clade=mammal&org=Human&db=hg19&position=ESR1) |  | NA | NA | T/0.844/0.105/1.06e-06 | T/0.816/0.036/0.2571 | NA | NA |
| rs73783837 | 6 | [SYNE1](http://genome.ucsc.edu/cgi-bin/hgTracks?hgHubConnect.destUrl=..%2Fcgi-bin%2FhgTracks&clade=mammal&org=Human&db=hg19&position=SYNE1) | [ESR1 (224939)](http://genome.ucsc.edu/cgi-bin/hgTracks?hgHubConnect.destUrl=..%2Fcgi-bin%2FhgTracks&clade=mammal&org=Human&db=hg19&position=ESR1) |  | NA | NA | G/0.834/0.098/4.35e-06 | G/0.809/0.032/0.3111 | NA | NA |
| rs73783839 | 6 | [SYNE1](http://genome.ucsc.edu/cgi-bin/hgTracks?hgHubConnect.destUrl=..%2Fcgi-bin%2FhgTracks&clade=mammal&org=Human&db=hg19&position=SYNE1) | [ESR1 (225078)](http://genome.ucsc.edu/cgi-bin/hgTracks?hgHubConnect.destUrl=..%2Fcgi-bin%2FhgTracks&clade=mammal&org=Human&db=hg19&position=ESR1) |  | NA | NA | G/0.843/0.104/1.67e-06 | G/0.817/0.036/0.2565 | NA | NA |
| rs9322369 | 6 | [SYNE1](http://genome.ucsc.edu/cgi-bin/hgTracks?hgHubConnect.destUrl=..%2Fcgi-bin%2FhgTracks&clade=mammal&org=Human&db=hg19&position=SYNE1) | [ESR1 (247875)](http://genome.ucsc.edu/cgi-bin/hgTracks?hgHubConnect.destUrl=..%2Fcgi-bin%2FhgTracks&clade=mammal&org=Human&db=hg19&position=ESR1) |  | T/0.602/0.008/0.5762 | T/0.604/0.009/0.5523 | T/0.37/0.079/2.17e-06 | T/0.34/0.069/0.00667 | A/0.536/0.027/0.00254 | A/0.566/0.016/0.09754 |
| rs893239 | 7 | [LFNG](http://genome.ucsc.edu/cgi-bin/hgTracks?hgHubConnect.destUrl=..%2Fcgi-bin%2FhgTracks&clade=mammal&org=Human&db=hg19&position=LFNG) | [BC034268 (77783)](http://genome.ucsc.edu/cgi-bin/hgTracks?hgHubConnect.destUrl=..%2Fcgi-bin%2FhgTracks&clade=mammal&org=Human&db=hg19&position=BC034268) | [MIR4648 (1439)](http://genome.ucsc.edu/cgi-bin/hgTracks?hgHubConnect.destUrl=..%2Fcgi-bin%2FhgTracks&clade=mammal&org=Human&db=hg19&position=MIR4648) | C/0.737/-0.009/0.595 | C/0.735/-0.008/0.691 | C/0.523/-0.117/9.62e-07 | C/0.546/-0.115/0.00283 | A/0.681/-0.038/0.00228 | A/0.717/-0.025/0.06781 |
| rs1009115 | 7 |  | [AX746880 (47330)](http://genome.ucsc.edu/cgi-bin/hgTracks?hgHubConnect.destUrl=..%2Fcgi-bin%2FhgTracks&clade=mammal&org=Human&db=hg19&position=AX746880) |  | C/0.739/-0.006/0.6671 | C/0.74/-0.008/0.6357 | C/0.932/0.15/2.6e-06 | C/0.913/0.093/0.03261 | T/0.762/0.012/0.2534 | T/0.75/-0.011/0.3186 |
| rs75890985 | 7 |  | [AX746880 (63260)](http://genome.ucsc.edu/cgi-bin/hgTracks?hgHubConnect.destUrl=..%2Fcgi-bin%2FhgTracks&clade=mammal&org=Human&db=hg19&position=AX746880) |  | C/0.853/-0.017/0.3498 | C/0.859/0.012/0.5663 | C/0.954/0.199/9.74e-07 | C/0.941/0.107/0.04746 | T/0.865/0.014/0.3133 | T/0.862/0.002/0.8984 |
| rs111751834 | 7 |  | [AX746880 (64084)](http://genome.ucsc.edu/cgi-bin/hgTracks?hgHubConnect.destUrl=..%2Fcgi-bin%2FhgTracks&clade=mammal&org=Human&db=hg19&position=AX746880) |  | G/0.853/-0.017/0.3524 | G/0.859/0.012/0.5629 | G/0.954/0.199/1e-06 | G/0.94/0.108/0.04725 | A/0.865/0.014/0.3114 | A/0.862/0.002/0.8933 |
| rs80328607 | 7 |  | [AX746880 (64271)](http://genome.ucsc.edu/cgi-bin/hgTracks?hgHubConnect.destUrl=..%2Fcgi-bin%2FhgTracks&clade=mammal&org=Human&db=hg19&position=AX746880) |  | T/0.853/-0.017/0.3532 | T/0.859/0.012/0.5618 | T/0.954/0.198/1.01e-06 | T/0.94/0.107/0.04733 | T/0.135/-0.014/0.3091 | T/0.138/-0.002/0.8889 |
| rs116884796 | 7 |  |  |  | G/0.99/-0.016/0.8802 | G/0.99/0.148/0.2316 | G/0.992/0.564/9.46e-07 | G/0.988/0.331/0.01584 | NA | NA |
| rs190130937 | 7 |  |  |  | NA | NA | C/0.993/0.517/2.95e-06 | C/0.989/0.381/0.00502 | NA | NA |
| rs74629737 | 7 | [BMPER](http://genome.ucsc.edu/cgi-bin/hgTracks?hgHubConnect.destUrl=..%2Fcgi-bin%2FhgTracks&clade=mammal&org=Human&db=hg19&position=BMPER) |  |  | NA | NA | C/1/4.013/7.84e-07 | C/0.999/2.199/0.00048 | NA | NA |
| rs146622168 | 7 |  | [NPSR1 (19593)](http://genome.ucsc.edu/cgi-bin/hgTracks?hgHubConnect.destUrl=..%2Fcgi-bin%2FhgTracks&clade=mammal&org=Human&db=hg19&position=NPSR1) | [DPY19L1 (23543)](http://genome.ucsc.edu/cgi-bin/hgTracks?hgHubConnect.destUrl=..%2Fcgi-bin%2FhgTracks&clade=mammal&org=Human&db=hg19&position=DPY19L1) | T/0.996/-0.008/0.9408 | T/0.995/0.015/0.8881 | T/1/2.101/2.95e-06 | T/0.999/1.244/0.00032 | T/0.004/-0.152/0.07079 | T/0.005/-0.182/0.01647 |
| rs189806577 | 7 | [DPY19L1](http://genome.ucsc.edu/cgi-bin/hgTracks?hgHubConnect.destUrl=..%2Fcgi-bin%2FhgTracks&clade=mammal&org=Human&db=hg19&position=DPY19L1) | [NPSR1 (59326)](http://genome.ucsc.edu/cgi-bin/hgTracks?hgHubConnect.destUrl=..%2Fcgi-bin%2FhgTracks&clade=mammal&org=Human&db=hg19&position=NPSR1) | [AJ011981 (2494)](http://genome.ucsc.edu/cgi-bin/hgTracks?hgHubConnect.destUrl=..%2Fcgi-bin%2FhgTracks&clade=mammal&org=Human&db=hg19&position=AJ011981) | T/0.996/0.021/0.8642 | T/0.996/0.058/0.6466 | T/1/2.193/3.75e-06 | T/0.999/1.309/0.00033 | T/0.004/-0.212/0.03526 | T/0.004/-0.228/0.01142 |
| rs73098786 | 7 | [DPY19L1](http://genome.ucsc.edu/cgi-bin/hgTracks?hgHubConnect.destUrl=..%2Fcgi-bin%2FhgTracks&clade=mammal&org=Human&db=hg19&position=DPY19L1) | [AJ011981 (42591)](http://genome.ucsc.edu/cgi-bin/hgTracks?hgHubConnect.destUrl=..%2Fcgi-bin%2FhgTracks&clade=mammal&org=Human&db=hg19&position=AJ011981) | [BC084560 (59751)](http://genome.ucsc.edu/cgi-bin/hgTracks?hgHubConnect.destUrl=..%2Fcgi-bin%2FhgTracks&clade=mammal&org=Human&db=hg19&position=BC084560) | T/0.996/0.017/0.8934 | T/0.996/0.063/0.6286 | T/0.999/2.194/4.7e-06 | T/0.998/1.321/0.00035 | T/0.004/-0.209/0.04292 | T/0.004/-0.23/0.01275 |
| rs183498415 | 7 | [DPY19L2P1](http://genome.ucsc.edu/cgi-bin/hgTracks?hgHubConnect.destUrl=..%2Fcgi-bin%2FhgTracks&clade=mammal&org=Human&db=hg19&position=DPY19L2P1) | [BC084560 (69237)](http://genome.ucsc.edu/cgi-bin/hgTracks?hgHubConnect.destUrl=..%2Fcgi-bin%2FhgTracks&clade=mammal&org=Human&db=hg19&position=BC084560) | [TBX20 (88640)](http://genome.ucsc.edu/cgi-bin/hgTracks?hgHubConnect.destUrl=..%2Fcgi-bin%2FhgTracks&clade=mammal&org=Human&db=hg19&position=TBX20) | T/0.997/0.008/0.9546 | T/0.996/0.084/0.5625 | T/1/2.365/3.7e-06 | T/0.999/1.417/0.00032 | NA | NA |
| rs186289784 | 7 | [AMPH](http://genome.ucsc.edu/cgi-bin/hgTracks?hgHubConnect.destUrl=..%2Fcgi-bin%2FhgTracks&clade=mammal&org=Human&db=hg19&position=AMPH) | [LOC100506776 (209153)](http://genome.ucsc.edu/cgi-bin/hgTracks?hgHubConnect.destUrl=..%2Fcgi-bin%2FhgTracks&clade=mammal&org=Human&db=hg19&position=LOC100506776) | [FAM183B (97554)](http://genome.ucsc.edu/cgi-bin/hgTracks?hgHubConnect.destUrl=..%2Fcgi-bin%2FhgTracks&clade=mammal&org=Human&db=hg19&position=FAM183B) | A/1/-0.403/0.4477 | A/1/-14.363/0.04347 | A/1/2.507/4.76e-06 | A/0.999/1.126/0.00747 | NA | NA |
| rs150347869 | 7 |  |  |  | A/0.997/0.259/0.06707 | A/0.996/0.069/0.6148 | A/1/2.647/3.5e-07 | A/0.999/1.472/0.00031 | A/0.004/-0.328/0.00151 | A/0.004/-0.119/0.1704 |
| rs186739251 | 7 |  | [GRB10 (205783)](http://genome.ucsc.edu/cgi-bin/hgTracks?hgHubConnect.destUrl=..%2Fcgi-bin%2FhgTracks&clade=mammal&org=Human&db=hg19&position=GRB10) | [COBL (27861)](http://genome.ucsc.edu/cgi-bin/hgTracks?hgHubConnect.destUrl=..%2Fcgi-bin%2FhgTracks&clade=mammal&org=Human&db=hg19&position=COBL) | NA | NA | C/0.999/1.943/1.78e-08 | C/0.998/0.968/0.0029 | NA | NA |
| rs114659987 | 7 | [COBL](http://genome.ucsc.edu/cgi-bin/hgTracks?hgHubConnect.destUrl=..%2Fcgi-bin%2FhgTracks&clade=mammal&org=Human&db=hg19&position=COBL) | [GRB10 (244699)](http://genome.ucsc.edu/cgi-bin/hgTracks?hgHubConnect.destUrl=..%2Fcgi-bin%2FhgTracks&clade=mammal&org=Human&db=hg19&position=GRB10) |  | NA | NA | A/0.999/2.463/3.21e-09 | A/0.998/1.014/0.00338 | NA | NA |
| rs111588657 | 7 |  |  |  | NA | NA | A/0.983/-0.29/4.09e-06 | A/0.989/-0.322/0.01105 | NA | NA |
| rs190817748 | 7 |  |  |  | NA | NA | A/0.984/-0.312/2.99e-06 | A/0.99/-0.319/0.01993 | NA | NA |
| rs114302618 | 7 |  |  | [COL1A2 (56812)](http://genome.ucsc.edu/cgi-bin/hgTracks?hgHubConnect.destUrl=..%2Fcgi-bin%2FhgTracks&clade=mammal&org=Human&db=hg19&position=COL1A2) | NA | NA | A/0.997/0.845/7.6e-07 | A/0.994/0.389/0.02518 | NA | NA |
| rs182858794 | 7 |  |  | [COL1A2 (42320)](http://genome.ucsc.edu/cgi-bin/hgTracks?hgHubConnect.destUrl=..%2Fcgi-bin%2FhgTracks&clade=mammal&org=Human&db=hg19&position=COL1A2) | NA | NA | A/0.996/0.812/4.51e-07 | A/0.993/0.376/0.02327 | NA | NA |
| rs189936349 | 7 |  |  | [COL1A2 (23398)](http://genome.ucsc.edu/cgi-bin/hgTracks?hgHubConnect.destUrl=..%2Fcgi-bin%2FhgTracks&clade=mammal&org=Human&db=hg19&position=COL1A2) | NA | NA | A/0.998/1.002/5.49e-08 | A/0.994/0.568/0.00179 | NA | NA |
| rs191979398 | 7 |  |  | [COL1A2 (15865)](http://genome.ucsc.edu/cgi-bin/hgTracks?hgHubConnect.destUrl=..%2Fcgi-bin%2FhgTracks&clade=mammal&org=Human&db=hg19&position=COL1A2) | NA | NA | A/0.997/0.998/5.64e-08 | A/0.994/0.564/0.00192 | NA | NA |
| rs191698899 | 7 |  |  | [COL1A2 (8142)](http://genome.ucsc.edu/cgi-bin/hgTracks?hgHubConnect.destUrl=..%2Fcgi-bin%2FhgTracks&clade=mammal&org=Human&db=hg19&position=COL1A2) | NA | NA | A/0.997/0.994/6.12e-08 | A/0.994/0.562/0.00199 | NA | NA |
| rs183110392 | 7 | [COL1A2](http://genome.ucsc.edu/cgi-bin/hgTracks?hgHubConnect.destUrl=..%2Fcgi-bin%2FhgTracks&clade=mammal&org=Human&db=hg19&position=COL1A2) |  | [CASD1 (85879)](http://genome.ucsc.edu/cgi-bin/hgTracks?hgHubConnect.destUrl=..%2Fcgi-bin%2FhgTracks&clade=mammal&org=Human&db=hg19&position=CASD1) | NA | NA | T/0.996/0.787/3.09e-06 | T/0.993/0.487/0.00535 | NA | NA |
| rs185746565 | 7 | [NUP205](http://genome.ucsc.edu/cgi-bin/hgTracks?hgHubConnect.destUrl=..%2Fcgi-bin%2FhgTracks&clade=mammal&org=Human&db=hg19&position=NUP205) | [CNOT4 (65174)](http://genome.ucsc.edu/cgi-bin/hgTracks?hgHubConnect.destUrl=..%2Fcgi-bin%2FhgTracks&clade=mammal&org=Human&db=hg19&position=CNOT4) | [C7orf73 (87171)](http://genome.ucsc.edu/cgi-bin/hgTracks?hgHubConnect.destUrl=..%2Fcgi-bin%2FhgTracks&clade=mammal&org=Human&db=hg19&position=C7orf73) | NA | NA | C/1/2.84/2.92e-06 | C/0.999/1.509/0.00164 | NA | NA |
| rs116129203 | 8 |  | [DEFB1 (43794)](http://genome.ucsc.edu/cgi-bin/hgTracks?hgHubConnect.destUrl=..%2Fcgi-bin%2FhgTracks&clade=mammal&org=Human&db=hg19&position=DEFB1) | [DEFA6 (2892)](http://genome.ucsc.edu/cgi-bin/hgTracks?hgHubConnect.destUrl=..%2Fcgi-bin%2FhgTracks&clade=mammal&org=Human&db=hg19&position=DEFA6) | NA | NA | A/0.996/0.938/2.71e-06 | A/0.994/0.661/0.00298 | NA | NA |
| rs147851892 | 8 |  | [ADAM28 (40743)](http://genome.ucsc.edu/cgi-bin/hgTracks?hgHubConnect.destUrl=..%2Fcgi-bin%2FhgTracks&clade=mammal&org=Human&db=hg19&position=ADAM28) | [ADAMDEC1 (7442)](http://genome.ucsc.edu/cgi-bin/hgTracks?hgHubConnect.destUrl=..%2Fcgi-bin%2FhgTracks&clade=mammal&org=Human&db=hg19&position=ADAMDEC1) | NA | NA | G/0.998/1.122/8.76e-07 | G/0.996/0.688/0.00069 | NA | NA |
| rs182387400 | 8 | [DOCK5](http://genome.ucsc.edu/cgi-bin/hgTracks?hgHubConnect.destUrl=..%2Fcgi-bin%2FhgTracks&clade=mammal&org=Human&db=hg19&position=DOCK5) |  | [PPP2R2A (100366)](http://genome.ucsc.edu/cgi-bin/hgTracks?hgHubConnect.destUrl=..%2Fcgi-bin%2FhgTracks&clade=mammal&org=Human&db=hg19&position=PPP2R2A) | NA | NA | T/0.994/0.68/2.45e-06 | T/0.99/0.347/0.02675 | NA | NA |
| rs147913210 | 8 | [DOCK5](http://genome.ucsc.edu/cgi-bin/hgTracks?hgHubConnect.destUrl=..%2Fcgi-bin%2FhgTracks&clade=mammal&org=Human&db=hg19&position=DOCK5) |  | [PPP2R2A (99841)](http://genome.ucsc.edu/cgi-bin/hgTracks?hgHubConnect.destUrl=..%2Fcgi-bin%2FhgTracks&clade=mammal&org=Human&db=hg19&position=PPP2R2A) | NA | NA | G/0.994/0.679/2.5e-06 | G/0.99/0.347/0.02687 | NA | NA |
| rs75455452 | 8 |  | [TRPA1 (3761)](http://genome.ucsc.edu/cgi-bin/hgTracks?hgHubConnect.destUrl=..%2Fcgi-bin%2FhgTracks&clade=mammal&org=Human&db=hg19&position=TRPA1) |  | NA | NA | T/0.994/0.578/3.71e-06 | T/0.99/0.401/0.00566 | NA | NA |
| rs187636186 | 8 |  | [TRPA1 (14649)](http://genome.ucsc.edu/cgi-bin/hgTracks?hgHubConnect.destUrl=..%2Fcgi-bin%2FhgTracks&clade=mammal&org=Human&db=hg19&position=TRPA1) |  | NA | NA | T/0.994/0.578/3.68e-06 | T/0.99/0.402/0.00559 | NA | NA |
| rs192074192 | 8 |  | [TRPA1 (38014)](http://genome.ucsc.edu/cgi-bin/hgTracks?hgHubConnect.destUrl=..%2Fcgi-bin%2FhgTracks&clade=mammal&org=Human&db=hg19&position=TRPA1) |  | NA | NA | G/0.994/0.579/3.53e-06 | G/0.99/0.401/0.00563 | NA | NA |
| rs192994403 | 8 |  |  | [HNF4G (108849)](http://genome.ucsc.edu/cgi-bin/hgTracks?hgHubConnect.destUrl=..%2Fcgi-bin%2FhgTracks&clade=mammal&org=Human&db=hg19&position=HNF4G) | NA | NA | C/0.999/1.985/2.83e-06 | C/0.998/0.867/0.01194 | NA | NA |
| rs188809019 | 8 |  | [MIR1208 (123801)](http://genome.ucsc.edu/cgi-bin/hgTracks?hgHubConnect.destUrl=..%2Fcgi-bin%2FhgTracks&clade=mammal&org=Human&db=hg19&position=MIR1208) |  | NA | NA | T/0.997/0.827/3.87e-06 | T/0.995/0.475/0.01411 | NA | NA |
| rs7815327 | 8 |  |  |  | T/0.993/0.02/0.8566 | T/0.993/-0.103/0.4062 | T/0.999/2.281/2.67e-06 | T/0.998/0.794/0.05017 | T/0.014/-0.087/0.1502 | T/0.014/-0.009/0.8606 |
| rs183726757 | 9 |  |  | [GLIS3 (29027)](http://genome.ucsc.edu/cgi-bin/hgTracks?hgHubConnect.destUrl=..%2Fcgi-bin%2FhgTracks&clade=mammal&org=Human&db=hg19&position=GLIS3) | NA | NA | T/1/2.616/4.79e-06 | T/0.999/1.29/0.00335 | NA | NA |
| rs190509410 | 10 |  |  | [ARID5B (34518)](http://genome.ucsc.edu/cgi-bin/hgTracks?hgHubConnect.destUrl=..%2Fcgi-bin%2FhgTracks&clade=mammal&org=Human&db=hg19&position=ARID5B) | A/0.998/-0.229/0.1766 | A/0.998/-0.333/0.1812 | A/1/2.674/1.99e-06 | A/0.999/1.141/0.01057 | NA | NA |
| rs182554582 | 10 |  | [GRID1 (68709)](http://genome.ucsc.edu/cgi-bin/hgTracks?hgHubConnect.destUrl=..%2Fcgi-bin%2FhgTracks&clade=mammal&org=Human&db=hg19&position=GRID1) | [WAPAL (53)](http://genome.ucsc.edu/cgi-bin/hgTracks?hgHubConnect.destUrl=..%2Fcgi-bin%2FhgTracks&clade=mammal&org=Human&db=hg19&position=WAPAL) | NA | NA | A/0.999/2.216/5.28e-07 | A/0.998/1.097/0.0017 | NA | NA |
| rs184737911 | 10 | [LDB3](http://genome.ucsc.edu/cgi-bin/hgTracks?hgHubConnect.destUrl=..%2Fcgi-bin%2FhgTracks&clade=mammal&org=Human&db=hg19&position=LDB3) | [OPN4 (4687)](http://genome.ucsc.edu/cgi-bin/hgTracks?hgHubConnect.destUrl=..%2Fcgi-bin%2FhgTracks&clade=mammal&org=Human&db=hg19&position=OPN4) | [AX747977 (60856)](http://genome.ucsc.edu/cgi-bin/hgTracks?hgHubConnect.destUrl=..%2Fcgi-bin%2FhgTracks&clade=mammal&org=Human&db=hg19&position=AX747977) | NA | NA | G/0.999/1.323/9.44e-07 | G/0.996/0.647/0.00359 | NA | NA |
| rs181778161 | 10 | [AK093219](http://genome.ucsc.edu/cgi-bin/hgTracks?hgHubConnect.destUrl=..%2Fcgi-bin%2FhgTracks&clade=mammal&org=Human&db=hg19&position=AK093219) | [LOC643529 (90882)](http://genome.ucsc.edu/cgi-bin/hgTracks?hgHubConnect.destUrl=..%2Fcgi-bin%2FhgTracks&clade=mammal&org=Human&db=hg19&position=LOC643529) |  | NA | NA | G/0.999/2.685/5.69e-07 | G/0.998/1.456/0.00075 | NA | NA |
| rs4076943 | 11 |  |  |  | T/0.652/0.007/0.5845 | T/0.649/0.018/0.2236 | T/0.725/-0.094/2.43e-07 | T/0.759/-0.041/0.1625 | A/0.668/-0.024/0.01022 | A/0.657/0.006/0.5359 |
| rs147831109 | 11 |  |  |  | T/0.991/-0.018/0.8203 | T/0.991/-0.032/0.7286 | T/0.998/1.013/3.59e-06 | T/0.995/0.528/0.00696 | NA | NA |
| rs187478995 | 11 |  |  |  | T/0.989/-0.01/0.8873 | T/0.99/-0.003/0.9656 | T/0.998/0.948/2.38e-06 | T/0.995/0.518/0.00427 | NA | NA |
| rs184852128 | 11 | [LRRC4C](http://genome.ucsc.edu/cgi-bin/hgTracks?hgHubConnect.destUrl=..%2Fcgi-bin%2FhgTracks&clade=mammal&org=Human&db=hg19&position=LRRC4C) |  |  | NA | NA | T/1/2.156/1.19e-06 | T/0.999/1.093/0.00138 | NA | NA |
| rs77771494 | 11 | [MS4A6E](http://genome.ucsc.edu/cgi-bin/hgTracks?hgHubConnect.destUrl=..%2Fcgi-bin%2FhgTracks&clade=mammal&org=Human&db=hg19&position=MS4A6E) | [MS4A4A (31655)](http://genome.ucsc.edu/cgi-bin/hgTracks?hgHubConnect.destUrl=..%2Fcgi-bin%2FhgTracks&clade=mammal&org=Human&db=hg19&position=MS4A4A) | [MS4A14 (37857)](http://genome.ucsc.edu/cgi-bin/hgTracks?hgHubConnect.destUrl=..%2Fcgi-bin%2FhgTracks&clade=mammal&org=Human&db=hg19&position=MS4A14) | NA | NA | T/0.963/0.211/2.54e-06 | T/0.95/0.091/0.1169 | NA | NA |
| rs79010836 | 11 |  | [MS4A6E (2162)](http://genome.ucsc.edu/cgi-bin/hgTracks?hgHubConnect.destUrl=..%2Fcgi-bin%2FhgTracks&clade=mammal&org=Human&db=hg19&position=MS4A6E) | [MS4A14 (35354)](http://genome.ucsc.edu/cgi-bin/hgTracks?hgHubConnect.destUrl=..%2Fcgi-bin%2FhgTracks&clade=mammal&org=Human&db=hg19&position=MS4A14) | NA | NA | C/0.963/0.212/2.41e-06 | C/0.95/0.092/0.1148 | NA | NA |
| rs114021604 | 11 |  | [MS4A6E (2362)](http://genome.ucsc.edu/cgi-bin/hgTracks?hgHubConnect.destUrl=..%2Fcgi-bin%2FhgTracks&clade=mammal&org=Human&db=hg19&position=MS4A6E) | [MS4A14 (35154)](http://genome.ucsc.edu/cgi-bin/hgTracks?hgHubConnect.destUrl=..%2Fcgi-bin%2FhgTracks&clade=mammal&org=Human&db=hg19&position=MS4A14) | NA | NA | G/0.963/0.212/2.42e-06 | G/0.95/0.092/0.1146 | NA | NA |
| rs75092417 | 11 |  | [MS4A6E (6316)](http://genome.ucsc.edu/cgi-bin/hgTracks?hgHubConnect.destUrl=..%2Fcgi-bin%2FhgTracks&clade=mammal&org=Human&db=hg19&position=MS4A6E) | [MS4A14 (31200)](http://genome.ucsc.edu/cgi-bin/hgTracks?hgHubConnect.destUrl=..%2Fcgi-bin%2FhgTracks&clade=mammal&org=Human&db=hg19&position=MS4A14) | NA | NA | G/0.963/0.201/4.79e-06 | G/0.95/0.097/0.09481 | NA | NA |
| rs115301024 | 11 |  | [MS4A6E (13300)](http://genome.ucsc.edu/cgi-bin/hgTracks?hgHubConnect.destUrl=..%2Fcgi-bin%2FhgTracks&clade=mammal&org=Human&db=hg19&position=MS4A6E) | [MS4A14 (24216)](http://genome.ucsc.edu/cgi-bin/hgTracks?hgHubConnect.destUrl=..%2Fcgi-bin%2FhgTracks&clade=mammal&org=Human&db=hg19&position=MS4A14) | NA | NA | A/0.962/0.201/4.29e-06 | A/0.95/0.097/0.09443 | NA | NA |
| rs114615507 | 11 |  | [MS4A6E (17376)](http://genome.ucsc.edu/cgi-bin/hgTracks?hgHubConnect.destUrl=..%2Fcgi-bin%2FhgTracks&clade=mammal&org=Human&db=hg19&position=MS4A6E) | [MS4A14 (20140)](http://genome.ucsc.edu/cgi-bin/hgTracks?hgHubConnect.destUrl=..%2Fcgi-bin%2FhgTracks&clade=mammal&org=Human&db=hg19&position=MS4A14) | NA | NA | G/0.962/0.2/4.45e-06 | G/0.95/0.096/0.09457 | NA | NA |
| rs114894087 | 11 |  | [MS4A6E (18405)](http://genome.ucsc.edu/cgi-bin/hgTracks?hgHubConnect.destUrl=..%2Fcgi-bin%2FhgTracks&clade=mammal&org=Human&db=hg19&position=MS4A6E) | [MS4A14 (19111)](http://genome.ucsc.edu/cgi-bin/hgTracks?hgHubConnect.destUrl=..%2Fcgi-bin%2FhgTracks&clade=mammal&org=Human&db=hg19&position=MS4A14) | NA | NA | G/0.962/0.2/4.52e-06 | G/0.95/0.096/0.09468 | NA | NA |
| rs115028228 | 11 |  | [MS4A6E (25089)](http://genome.ucsc.edu/cgi-bin/hgTracks?hgHubConnect.destUrl=..%2Fcgi-bin%2FhgTracks&clade=mammal&org=Human&db=hg19&position=MS4A6E) | [MS4A14 (12427)](http://genome.ucsc.edu/cgi-bin/hgTracks?hgHubConnect.destUrl=..%2Fcgi-bin%2FhgTracks&clade=mammal&org=Human&db=hg19&position=MS4A14) | NA | NA | T/0.962/0.199/4.91e-06 | T/0.95/0.096/0.0949 | NA | NA |
| rs75368561 | 11 | [MS4A14](http://genome.ucsc.edu/cgi-bin/hgTracks?hgHubConnect.destUrl=..%2Fcgi-bin%2FhgTracks&clade=mammal&org=Human&db=hg19&position=MS4A14) | [MS4A7 (17349)](http://genome.ucsc.edu/cgi-bin/hgTracks?hgHubConnect.destUrl=..%2Fcgi-bin%2FhgTracks&clade=mammal&org=Human&db=hg19&position=MS4A7) | [MS4A5 (16286)](http://genome.ucsc.edu/cgi-bin/hgTracks?hgHubConnect.destUrl=..%2Fcgi-bin%2FhgTracks&clade=mammal&org=Human&db=hg19&position=MS4A5) | NA | NA | G/0.969/0.221/4.78e-06 | G/0.957/0.118/0.06143 | NA | NA |
| rs77253690 | 11 |  | [MS4A14 (7085)](http://genome.ucsc.edu/cgi-bin/hgTracks?hgHubConnect.destUrl=..%2Fcgi-bin%2FhgTracks&clade=mammal&org=Human&db=hg19&position=MS4A14) | [MS4A5 (4748)](http://genome.ucsc.edu/cgi-bin/hgTracks?hgHubConnect.destUrl=..%2Fcgi-bin%2FhgTracks&clade=mammal&org=Human&db=hg19&position=MS4A5) | NA | NA | G/0.97/0.228/3.04e-06 | G/0.958/0.13/0.03625 | NA | NA |
| rs138753278 | 11 |  | [MS4A14 (9586)](http://genome.ucsc.edu/cgi-bin/hgTracks?hgHubConnect.destUrl=..%2Fcgi-bin%2FhgTracks&clade=mammal&org=Human&db=hg19&position=MS4A14) | [MS4A5 (2247)](http://genome.ucsc.edu/cgi-bin/hgTracks?hgHubConnect.destUrl=..%2Fcgi-bin%2FhgTracks&clade=mammal&org=Human&db=hg19&position=MS4A5) | A/0.994/-0.02/0.8522 | A/0.994/-0.122/0.3322 | A/0.966/0.216/4e-06 | A/0.954/0.141/0.01998 | A/0.028/-0.165/7.08e-05 | A/0.033/-0.056/0.2644 |
| rs149746592 | 11 |  | [AHNAK (11375)](http://genome.ucsc.edu/cgi-bin/hgTracks?hgHubConnect.destUrl=..%2Fcgi-bin%2FhgTracks&clade=mammal&org=Human&db=hg19&position=AHNAK) | [EEF1G (1365)](http://genome.ucsc.edu/cgi-bin/hgTracks?hgHubConnect.destUrl=..%2Fcgi-bin%2FhgTracks&clade=mammal&org=Human&db=hg19&position=EEF1G) | A/0.996/0.063/0.7301 | A/0.996/-0.023/0.907 | A/0.976/0.262/1.33e-06 | A/0.967/0.248/0.00056 | NA | NA |
| rs116766958 | 11 | [TUT1](http://genome.ucsc.edu/cgi-bin/hgTracks?hgHubConnect.destUrl=..%2Fcgi-bin%2FhgTracks&clade=mammal&org=Human&db=hg19&position=TUT1) | [EEF1G (4109)](http://genome.ucsc.edu/cgi-bin/hgTracks?hgHubConnect.destUrl=..%2Fcgi-bin%2FhgTracks&clade=mammal&org=Human&db=hg19&position=EEF1G) | [MTA2 (14164)](http://genome.ucsc.edu/cgi-bin/hgTracks?hgHubConnect.destUrl=..%2Fcgi-bin%2FhgTracks&clade=mammal&org=Human&db=hg19&position=MTA2) | NA | NA | T/0.978/0.266/1.82e-06 | T/0.97/0.273/0.00023 | NA | NA |
| rs146799327 | 11 | [TUT1](http://genome.ucsc.edu/cgi-bin/hgTracks?hgHubConnect.destUrl=..%2Fcgi-bin%2FhgTracks&clade=mammal&org=Human&db=hg19&position=TUT1) | [EEF1G (4630)](http://genome.ucsc.edu/cgi-bin/hgTracks?hgHubConnect.destUrl=..%2Fcgi-bin%2FhgTracks&clade=mammal&org=Human&db=hg19&position=EEF1G) | [MTA2 (13643)](http://genome.ucsc.edu/cgi-bin/hgTracks?hgHubConnect.destUrl=..%2Fcgi-bin%2FhgTracks&clade=mammal&org=Human&db=hg19&position=MTA2) | NA | NA | T/0.996/0.822/1.38e-06 | T/0.993/0.485/0.00813 | NA | NA |
| rs111394397 | 11 | [TUT1](http://genome.ucsc.edu/cgi-bin/hgTracks?hgHubConnect.destUrl=..%2Fcgi-bin%2FhgTracks&clade=mammal&org=Human&db=hg19&position=TUT1) | [EEF1G (9290)](http://genome.ucsc.edu/cgi-bin/hgTracks?hgHubConnect.destUrl=..%2Fcgi-bin%2FhgTracks&clade=mammal&org=Human&db=hg19&position=EEF1G) | [MTA2 (8983)](http://genome.ucsc.edu/cgi-bin/hgTracks?hgHubConnect.destUrl=..%2Fcgi-bin%2FhgTracks&clade=mammal&org=Human&db=hg19&position=MTA2) | NA | NA | T/0.979/0.263/3.09e-06 | T/0.972/0.268/4e-04 | NA | NA |
| rs180910263 | 11 |  | [CTTN (28355)](http://genome.ucsc.edu/cgi-bin/hgTracks?hgHubConnect.destUrl=..%2Fcgi-bin%2FhgTracks&clade=mammal&org=Human&db=hg19&position=CTTN) | [SHANK2 (2915)](http://genome.ucsc.edu/cgi-bin/hgTracks?hgHubConnect.destUrl=..%2Fcgi-bin%2FhgTracks&clade=mammal&org=Human&db=hg19&position=SHANK2) | G/0.997/0.393/0.04862 | G/0.997/0.618/0.00274 | G/1/3.166/8.65e-07 | G/0.999/1.448/0.00379 | NA | NA |
| rs143737902 | 11 | [AK128563](http://genome.ucsc.edu/cgi-bin/hgTracks?hgHubConnect.destUrl=..%2Fcgi-bin%2FhgTracks&clade=mammal&org=Human&db=hg19&position=AK128563) | [CTTN (37733)](http://genome.ucsc.edu/cgi-bin/hgTracks?hgHubConnect.destUrl=..%2Fcgi-bin%2FhgTracks&clade=mammal&org=Human&db=hg19&position=CTTN) | [SHANK2 (15847)](http://genome.ucsc.edu/cgi-bin/hgTracks?hgHubConnect.destUrl=..%2Fcgi-bin%2FhgTracks&clade=mammal&org=Human&db=hg19&position=SHANK2) | G/0.998/0.478/0.02925 | G/0.998/0.664/0.0024 | G/1/3.126/1.35e-06 | G/0.999/1.425/0.00452 | NA | NA |
| rs117767473 | 11 | [BC127192](http://genome.ucsc.edu/cgi-bin/hgTracks?hgHubConnect.destUrl=..%2Fcgi-bin%2FhgTracks&clade=mammal&org=Human&db=hg19&position=BC127192) | [AK128563 (5318)](http://genome.ucsc.edu/cgi-bin/hgTracks?hgHubConnect.destUrl=..%2Fcgi-bin%2FhgTracks&clade=mammal&org=Human&db=hg19&position=AK128563) | [SHANK2 (9677)](http://genome.ucsc.edu/cgi-bin/hgTracks?hgHubConnect.destUrl=..%2Fcgi-bin%2FhgTracks&clade=mammal&org=Human&db=hg19&position=SHANK2) | A/0.996/0.541/0.00346 | A/0.996/0.639/0.00044 | A/0.999/2.856/2.22e-08 | A/0.998/1.495/0.00022 | NA | NA |
| rs144903619 | 11 | [BC127192](http://genome.ucsc.edu/cgi-bin/hgTracks?hgHubConnect.destUrl=..%2Fcgi-bin%2FhgTracks&clade=mammal&org=Human&db=hg19&position=BC127192) | [AK128563 (24334)](http://genome.ucsc.edu/cgi-bin/hgTracks?hgHubConnect.destUrl=..%2Fcgi-bin%2FhgTracks&clade=mammal&org=Human&db=hg19&position=AK128563) | [BC131767 (103798)](http://genome.ucsc.edu/cgi-bin/hgTracks?hgHubConnect.destUrl=..%2Fcgi-bin%2FhgTracks&clade=mammal&org=Human&db=hg19&position=BC131767) | T/0.998/0.482/0.03226 | T/0.998/0.706/0.00154 | T/1/3.123/1.54e-06 | T/0.999/1.423/0.00496 | NA | NA |
| rs189780858 | 11 | [DLG2](http://genome.ucsc.edu/cgi-bin/hgTracks?hgHubConnect.destUrl=..%2Fcgi-bin%2FhgTracks&clade=mammal&org=Human&db=hg19&position=DLG2) |  |  | T/0.989/-0.066/0.3119 | T/0.989/0.016/0.8327 | T/0.998/0.82/3.93e-06 | T/0.994/0.365/0.02008 | T/0.01/-0.004/0.9333 | T/0.01/-0.04/0.4117 |
| rs12807873 | 11 | [GRIA4](http://genome.ucsc.edu/cgi-bin/hgTracks?hgHubConnect.destUrl=..%2Fcgi-bin%2FhgTracks&clade=mammal&org=Human&db=hg19&position=GRIA4) |  |  | T/0.995/-0.04/0.7556 | T/0.996/0.167/0.2644 | T/0.999/2.582/4.57e-06 | T/0.999/1.287/0.00815 | T/0.004/0.028/0.8069 | T/0.004/-0.12/0.3114 |
| rs7941140 | 11 |  | [SIK3 (27768)](http://genome.ucsc.edu/cgi-bin/hgTracks?hgHubConnect.destUrl=..%2Fcgi-bin%2FhgTracks&clade=mammal&org=Human&db=hg19&position=SIK3) | [AB231710 (10500)](http://genome.ucsc.edu/cgi-bin/hgTracks?hgHubConnect.destUrl=..%2Fcgi-bin%2FhgTracks&clade=mammal&org=Human&db=hg19&position=AB231710) | NA | NA | T/0.909/0.133/1.96e-06 | T/0.884/0.041/0.2701 | NA | NA |
| rs191000678 | 12 |  | [PTHLH (164096)](http://genome.ucsc.edu/cgi-bin/hgTracks?hgHubConnect.destUrl=..%2Fcgi-bin%2FhgTracks&clade=mammal&org=Human&db=hg19&position=PTHLH) | [CCDC91 (45219)](http://genome.ucsc.edu/cgi-bin/hgTracks?hgHubConnect.destUrl=..%2Fcgi-bin%2FhgTracks&clade=mammal&org=Human&db=hg19&position=CCDC91) | NA | NA | T/0.998/0.884/1.02e-06 | T/0.995/0.631/0.00048 | NA | NA |
| rs181609621 | 12 |  | [FGD4 (74870)](http://genome.ucsc.edu/cgi-bin/hgTracks?hgHubConnect.destUrl=..%2Fcgi-bin%2FhgTracks&clade=mammal&org=Human&db=hg19&position=FGD4) | [DNM1L (21377)](http://genome.ucsc.edu/cgi-bin/hgTracks?hgHubConnect.destUrl=..%2Fcgi-bin%2FhgTracks&clade=mammal&org=Human&db=hg19&position=DNM1L) | C/0.994/0.115/0.2122 | C/0.994/0.138/0.1925 | C/1/2.19/3.08e-06 | C/0.998/0.991/0.00655 | A/0.994/0.102/0.1579 | A/0.994/0.153/0.02871 |
| rs183884080 | 12 |  | [IL26 (2406)](http://genome.ucsc.edu/cgi-bin/hgTracks?hgHubConnect.destUrl=..%2Fcgi-bin%2FhgTracks&clade=mammal&org=Human&db=hg19&position=IL26) | [IL22 (20047)](http://genome.ucsc.edu/cgi-bin/hgTracks?hgHubConnect.destUrl=..%2Fcgi-bin%2FhgTracks&clade=mammal&org=Human&db=hg19&position=IL22) | G/0.995/0/0.9969 | G/0.995/-0.148/0.1747 | G/0.999/1.307/1.6e-06 | G/0.997/0.688/0.01053 | C/0.996/0.14/0.08671 | C/0.996/0.012/0.8823 |
| rs188255132 | 12 | [PPFIA2](http://genome.ucsc.edu/cgi-bin/hgTracks?hgHubConnect.destUrl=..%2Fcgi-bin%2FhgTracks&clade=mammal&org=Human&db=hg19&position=PPFIA2) | [ACSS3 (73606)](http://genome.ucsc.edu/cgi-bin/hgTracks?hgHubConnect.destUrl=..%2Fcgi-bin%2FhgTracks&clade=mammal&org=Human&db=hg19&position=ACSS3) |  | NA | NA | A/1/2.347/4.07e-06 | A/0.999/1.262/0.00155 | NA | NA |
| rs183160876 | 12 |  |  |  | T/0.999/0.29/0.2803 | T/0.999/0.045/0.8884 | T/1/2.194/8.92e-07 | T/0.999/1.109/0.00129 | T/0.001/-0.475/0.00672 | T/0.001/-0.298/0.06415 |
| rs4542491 | 12 |  |  | [CCDC59 (209238)](http://genome.ucsc.edu/cgi-bin/hgTracks?hgHubConnect.destUrl=..%2Fcgi-bin%2FhgTracks&clade=mammal&org=Human&db=hg19&position=CCDC59) | C/0.999/0.166/0.5745 | C/0.999/-0.031/0.9415 | C/1/2.107/3.45e-06 | C/0.999/1.08/0.00199 | T/0.999/0.428/0.02419 | T/0.999/0.347/0.0557 |
| rs139236384 | 12 |  |  | [CCDC59 (196057)](http://genome.ucsc.edu/cgi-bin/hgTracks?hgHubConnect.destUrl=..%2Fcgi-bin%2FhgTracks&clade=mammal&org=Human&db=hg19&position=CCDC59) | G/0.999/0.158/0.5953 | G/0.999/-0.041/0.9236 | G/1/2.192/1.14e-06 | G/0.998/1.117/0.00131 | C/0.999/0.438/0.02113 | C/0.999/0.357/0.04979 |
| rs116910038 | 12 | [TSPAN19](http://genome.ucsc.edu/cgi-bin/hgTracks?hgHubConnect.destUrl=..%2Fcgi-bin%2FhgTracks&clade=mammal&org=Human&db=hg19&position=TSPAN19) | [SLC6A15 (117487)](http://genome.ucsc.edu/cgi-bin/hgTracks?hgHubConnect.destUrl=..%2Fcgi-bin%2FhgTracks&clade=mammal&org=Human&db=hg19&position=SLC6A15) | [LRRIQ1 (6005)](http://genome.ucsc.edu/cgi-bin/hgTracks?hgHubConnect.destUrl=..%2Fcgi-bin%2FhgTracks&clade=mammal&org=Human&db=hg19&position=LRRIQ1) | T/0.902/-0.009/0.7324 | T/0.902/0.016/0.567 | T/0.982/0.329/3.04e-06 | T/0.975/0.168/0.06862 | T/0.088/-0.046/0.02214 | T/0.091/-0.053/0.0072 |
| rs187607716 | 12 |  | [KERA (35386)](http://genome.ucsc.edu/cgi-bin/hgTracks?hgHubConnect.destUrl=..%2Fcgi-bin%2FhgTracks&clade=mammal&org=Human&db=hg19&position=KERA) | [LUM (9714)](http://genome.ucsc.edu/cgi-bin/hgTracks?hgHubConnect.destUrl=..%2Fcgi-bin%2FhgTracks&clade=mammal&org=Human&db=hg19&position=LUM) | NA | NA | G/1/2.412/2.84e-07 | G/0.999/1.44/7.48e-05 | NA | NA |
| rs190262953 | 12 |  |  |  | NA | NA | T/1/2.16/2.17e-06 | T/0.999/1.502/7.2e-05 | NA | NA |
| rs189419463 | 12 |  |  | [BTBD11 (10710)](http://genome.ucsc.edu/cgi-bin/hgTracks?hgHubConnect.destUrl=..%2Fcgi-bin%2FhgTracks&clade=mammal&org=Human&db=hg19&position=BTBD11) | C/0.998/0.12/0.4102 | C/0.997/-0.075/0.6112 | C/1/5.813/2.86e-06 | C/1/3.022/0.0016 | C/0.003/0.039/0.7268 | C/0.003/0.072/0.5155 |
| rs139680557 | 12 |  | [BTBD11 (32422)](http://genome.ucsc.edu/cgi-bin/hgTracks?hgHubConnect.destUrl=..%2Fcgi-bin%2FhgTracks&clade=mammal&org=Human&db=hg19&position=BTBD11) | [PWP1 (18000)](http://genome.ucsc.edu/cgi-bin/hgTracks?hgHubConnect.destUrl=..%2Fcgi-bin%2FhgTracks&clade=mammal&org=Human&db=hg19&position=PWP1) | T/0.986/0.01/0.9148 | T/0.985/-0.097/0.3263 | T/0.998/1.44/3.94e-06 | T/0.997/0.966/0.00306 | T/0.012/-0.134/0.06183 | T/0.013/-0.008/0.9015 |
| rs73200436 | 12 |  | [MVK (27622)](http://genome.ucsc.edu/cgi-bin/hgTracks?hgHubConnect.destUrl=..%2Fcgi-bin%2FhgTracks&clade=mammal&org=Human&db=hg19&position=MVK) | [C12orf34 (89493)](http://genome.ucsc.edu/cgi-bin/hgTracks?hgHubConnect.destUrl=..%2Fcgi-bin%2FhgTracks&clade=mammal&org=Human&db=hg19&position=C12orf34) | A/0.981/0.021/0.7042 | A/0.979/-0.069/0.2468 | A/0.997/1.016/3.53e-06 | A/0.996/0.868/0.00032 | A/0.018/-0.066/0.1352 | A/0.02/0.003/0.9491 |
| rs73200449 | 12 |  | [MVK (43896)](http://genome.ucsc.edu/cgi-bin/hgTracks?hgHubConnect.destUrl=..%2Fcgi-bin%2FhgTracks&clade=mammal&org=Human&db=hg19&position=MVK) | [C12orf34 (73219)](http://genome.ucsc.edu/cgi-bin/hgTracks?hgHubConnect.destUrl=..%2Fcgi-bin%2FhgTracks&clade=mammal&org=Human&db=hg19&position=C12orf34) | T/0.964/-0.005/0.9052 | T/0.962/-0.059/0.1838 | T/0.994/0.629/1.73e-06 | T/0.992/0.592/0.00022 | T/0.033/-0.029/0.3546 | T/0.035/0.024/0.4326 |
| rs183032784 | 12 | [KSR2](http://genome.ucsc.edu/cgi-bin/hgTracks?hgHubConnect.destUrl=..%2Fcgi-bin%2FhgTracks&clade=mammal&org=Human&db=hg19&position=KSR2) |  | [RFC5 (197291)](http://genome.ucsc.edu/cgi-bin/hgTracks?hgHubConnect.destUrl=..%2Fcgi-bin%2FhgTracks&clade=mammal&org=Human&db=hg19&position=RFC5) | A/0.997/-0.091/0.4597 | A/0.997/0.006/0.9656 | A/1/1.96/1.48e-06 | A/0.998/0.946/0.00275 | A/0.003/-0.074/0.4673 | A/0.003/-0.106/0.2721 |
| rs75311705 | 12 |  | [AACS (86725)](http://genome.ucsc.edu/cgi-bin/hgTracks?hgHubConnect.destUrl=..%2Fcgi-bin%2FhgTracks&clade=mammal&org=Human&db=hg19&position=AACS) | [TMEM132B (96565)](http://genome.ucsc.edu/cgi-bin/hgTracks?hgHubConnect.destUrl=..%2Fcgi-bin%2FhgTracks&clade=mammal&org=Human&db=hg19&position=TMEM132B) | A/0.999/-0.405/0.05458 | A/0.999/-0.225/0.4906 | A/0.991/0.56/3.24e-07 | A/0.986/0.382/0.00278 | NA | NA |
| rs117876032 | 12 |  | [AACS (108132)](http://genome.ucsc.edu/cgi-bin/hgTracks?hgHubConnect.destUrl=..%2Fcgi-bin%2FhgTracks&clade=mammal&org=Human&db=hg19&position=AACS) | [TMEM132B (75158)](http://genome.ucsc.edu/cgi-bin/hgTracks?hgHubConnect.destUrl=..%2Fcgi-bin%2FhgTracks&clade=mammal&org=Human&db=hg19&position=TMEM132B) | NA | NA | A/0.999/2.11/1.96e-06 | A/0.998/2.037/1.15e-05 | NA | NA |
| rs140979249 | 13 | [DCLK1](http://genome.ucsc.edu/cgi-bin/hgTracks?hgHubConnect.destUrl=..%2Fcgi-bin%2FhgTracks&clade=mammal&org=Human&db=hg19&position=DCLK1) |  | [CCDC169-SOHLH2 (138383)](http://genome.ucsc.edu/cgi-bin/hgTracks?hgHubConnect.destUrl=..%2Fcgi-bin%2FhgTracks&clade=mammal&org=Human&db=hg19&position=CCDC169-SOHLH2) | G/0.956/-0.066/0.08402 | G/0.959/-0.09/0.04888 | G/0.989/0.472/4.04e-06 | G/0.983/0.117/0.3281 | A/0.953/-0.002/0.9557 | A/0.952/0.014/0.6034 |
| rs35822625 | 13 |  | [DCLK1 (8855)](http://genome.ucsc.edu/cgi-bin/hgTracks?hgHubConnect.destUrl=..%2Fcgi-bin%2FhgTracks&clade=mammal&org=Human&db=hg19&position=DCLK1) | [CCDC169-SOHLH2 (27975)](http://genome.ucsc.edu/cgi-bin/hgTracks?hgHubConnect.destUrl=..%2Fcgi-bin%2FhgTracks&clade=mammal&org=Human&db=hg19&position=CCDC169-SOHLH2) | C/0.941/-0.057/0.0502 | C/0.944/-0.051/0.1345 | C/0.987/0.372/3.96e-06 | C/0.982/0.148/0.1489 | T/0.941/-0.009/0.6815 | T/0.941/0.006/0.7951 |
| rs35540212 | 13 | [CCDC169-SOHLH2](http://genome.ucsc.edu/cgi-bin/hgTracks?hgHubConnect.destUrl=..%2Fcgi-bin%2FhgTracks&clade=mammal&org=Human&db=hg19&position=CCDC169-SOHLH2) |  | [CCDC169 (32496)](http://genome.ucsc.edu/cgi-bin/hgTracks?hgHubConnect.destUrl=..%2Fcgi-bin%2FhgTracks&clade=mammal&org=Human&db=hg19&position=CCDC169) | A/0.919/-0.043/0.07576 | A/0.921/-0.022/0.4438 | A/0.98/0.313/2.47e-06 | A/0.972/0.183/0.03451 | A/0.077/0/0.9904 | A/0.077/-0.012/0.4974 |
| rs181987887 | 13 |  |  | [KCTD12 (207784)](http://genome.ucsc.edu/cgi-bin/hgTracks?hgHubConnect.destUrl=..%2Fcgi-bin%2FhgTracks&clade=mammal&org=Human&db=hg19&position=KCTD12) | NA | NA | A/0.999/1.85/2.22e-06 | A/0.998/0.832/0.00847 | NA | NA |
| rs191275341 | 13 |  | [GPC6 (108333)](http://genome.ucsc.edu/cgi-bin/hgTracks?hgHubConnect.destUrl=..%2Fcgi-bin%2FhgTracks&clade=mammal&org=Human&db=hg19&position=GPC6) | [DCT (24719)](http://genome.ucsc.edu/cgi-bin/hgTracks?hgHubConnect.destUrl=..%2Fcgi-bin%2FhgTracks&clade=mammal&org=Human&db=hg19&position=DCT) | NA | NA | C/1/2.669/1.1e-08 | C/0.999/1.393/0.00012 | NA | NA |
| rs74731421 | 14 | [C14orf25](http://genome.ucsc.edu/cgi-bin/hgTracks?hgHubConnect.destUrl=..%2Fcgi-bin%2FhgTracks&clade=mammal&org=Human&db=hg19&position=C14orf25) | [FOXA1 (145797)](http://genome.ucsc.edu/cgi-bin/hgTracks?hgHubConnect.destUrl=..%2Fcgi-bin%2FhgTracks&clade=mammal&org=Human&db=hg19&position=FOXA1) | [TTC6 (31301)](http://genome.ucsc.edu/cgi-bin/hgTracks?hgHubConnect.destUrl=..%2Fcgi-bin%2FhgTracks&clade=mammal&org=Human&db=hg19&position=TTC6) | A/0.956/-0.017/0.6434 | A/0.959/0.016/0.7038 | A/0.993/0.568/1.19e-06 | A/0.989/0.307/0.02833 | A/0.04/-0.054/0.05725 | A/0.04/-0.038/0.1773 |
| rs181881613 | 14 |  |  |  | NA | NA | T/0.999/1.826/4.31e-06 | T/0.998/0.952/0.01031 | NA | NA |
| rs191739273 | 14 |  |  |  | NA | NA | G/1/2.177/2.4e-07 | G/0.998/1.172/3e-04 | NA | NA |
| rs186152940 | 14 |  |  |  | A/0.998/-0.184/0.2965 | A/0.998/-0.006/0.9766 | A/0.999/1.62/3.28e-06 | A/0.998/1.133/0.00028 | A/0.002/-0.245/0.07863 | A/0.002/-0.334/0.01201 |
| rs191755093 | 14 |  |  |  | NA | NA | T/1/2.206/2.01e-07 | T/0.998/1.178/0.00031 | NA | NA |
| rs192522312 | 14 |  |  | [RPL10L (37748)](http://genome.ucsc.edu/cgi-bin/hgTracks?hgHubConnect.destUrl=..%2Fcgi-bin%2FhgTracks&clade=mammal&org=Human&db=hg19&position=RPL10L) | NA | NA | C/1/2.547/2.11e-07 | C/0.998/1.342/0.00038 | NA | NA |
| rs185449237 | 14 |  |  | [MDGA2 (109494)](http://genome.ucsc.edu/cgi-bin/hgTracks?hgHubConnect.destUrl=..%2Fcgi-bin%2FhgTracks&clade=mammal&org=Human&db=hg19&position=MDGA2) | NA | NA | A/1/2.782/2.56e-07 | A/0.999/1.471/4e-04 | NA | NA |
| rs192383543 | 14 |  |  | [MDGA2 (107965)](http://genome.ucsc.edu/cgi-bin/hgTracks?hgHubConnect.destUrl=..%2Fcgi-bin%2FhgTracks&clade=mammal&org=Human&db=hg19&position=MDGA2) | NA | NA | C/1/2.784/2.55e-07 | C/0.999/1.472/4e-04 | NA | NA |
| rs188669819 | 14 | [MDGA2](http://genome.ucsc.edu/cgi-bin/hgTracks?hgHubConnect.destUrl=..%2Fcgi-bin%2FhgTracks&clade=mammal&org=Human&db=hg19&position=MDGA2) |  |  | NA | NA | T/1/3.495/2.92e-06 | T/0.999/1.923/0.00081 | NA | NA |
| rs12586420 | 14 |  |  |  | A/0.664/0.015/0.298 | A/0.665/0.005/0.7536 | A/0.351/0.095/3.32e-07 | A/0.325/0.077/0.00717 | A/0.424/-0.017/0.08045 | A/0.379/0.004/0.6677 |
| rs12586478 | 14 |  |  |  | G/0.666/0.015/0.3082 | G/0.667/0.005/0.7741 | G/0.352/0.095/2.88e-07 | G/0.326/0.077/0.00701 | T/0.577/0.017/0.08428 | T/0.622/-0.005/0.6439 |
| rs138064340 | 14 |  |  |  | NA | NA | C/0.997/1.07/5.85e-08 | C/0.995/0.843/0.00014 | NA | NA |
| rs150978828 | 15 |  | [MEIS2 (109623)](http://genome.ucsc.edu/cgi-bin/hgTracks?hgHubConnect.destUrl=..%2Fcgi-bin%2FhgTracks&clade=mammal&org=Human&db=hg19&position=MEIS2) |  | NA | NA | T/0.954/0.19/4.87e-06 | T/0.943/0.184/0.00122 | NA | NA |
| rs142859932 | 15 |  | [HCN4 (69078)](http://genome.ucsc.edu/cgi-bin/hgTracks?hgHubConnect.destUrl=..%2Fcgi-bin%2FhgTracks&clade=mammal&org=Human&db=hg19&position=HCN4) | [C15orf60 (4815)](http://genome.ucsc.edu/cgi-bin/hgTracks?hgHubConnect.destUrl=..%2Fcgi-bin%2FhgTracks&clade=mammal&org=Human&db=hg19&position=C15orf60) | NA | NA | G/0.991/0.487/3.43e-06 | G/0.985/0.315/0.00601 | NA | NA |
| rs191299947 | 15 | [ARNT2](http://genome.ucsc.edu/cgi-bin/hgTracks?hgHubConnect.destUrl=..%2Fcgi-bin%2FhgTracks&clade=mammal&org=Human&db=hg19&position=ARNT2) | [AB240015 (218036)](http://genome.ucsc.edu/cgi-bin/hgTracks?hgHubConnect.destUrl=..%2Fcgi-bin%2FhgTracks&clade=mammal&org=Human&db=hg19&position=AB240015) | [FAM108C1 (110094)](http://genome.ucsc.edu/cgi-bin/hgTracks?hgHubConnect.destUrl=..%2Fcgi-bin%2FhgTracks&clade=mammal&org=Human&db=hg19&position=FAM108C1) | NA | NA | A/0.994/0.556/1.13e-06 | A/0.988/0.177/0.1387 | NA | NA |
| rs138436123 | 16 | [FLYWCH1](http://genome.ucsc.edu/cgi-bin/hgTracks?hgHubConnect.destUrl=..%2Fcgi-bin%2FhgTracks&clade=mammal&org=Human&db=hg19&position=FLYWCH1) | [FLYWCH2 (28685)](http://genome.ucsc.edu/cgi-bin/hgTracks?hgHubConnect.destUrl=..%2Fcgi-bin%2FhgTracks&clade=mammal&org=Human&db=hg19&position=FLYWCH2) | [KREMEN2 (36148)](http://genome.ucsc.edu/cgi-bin/hgTracks?hgHubConnect.destUrl=..%2Fcgi-bin%2FhgTracks&clade=mammal&org=Human&db=hg19&position=KREMEN2) | C/0.998/-0.074/0.7318 | C/0.998/0.273/0.3467 | C/0.999/2.387/1.27e-06 | C/0.998/1.202/0.00162 | NA | NA |
| rs186112291 | 16 | [PYCARD](http://genome.ucsc.edu/cgi-bin/hgTracks?hgHubConnect.destUrl=..%2Fcgi-bin%2FhgTracks&clade=mammal&org=Human&db=hg19&position=PYCARD) | [FUS (6989)](http://genome.ucsc.edu/cgi-bin/hgTracks?hgHubConnect.destUrl=..%2Fcgi-bin%2FhgTracks&clade=mammal&org=Human&db=hg19&position=FUS) | [TRIM72 (12160)](http://genome.ucsc.edu/cgi-bin/hgTracks?hgHubConnect.destUrl=..%2Fcgi-bin%2FhgTracks&clade=mammal&org=Human&db=hg19&position=TRIM72) | T/0.995/-0.094/0.4136 | T/0.995/0.014/0.9182 | T/0.999/1.388/1.46e-06 | T/0.997/0.591/0.0119 | T/0.005/-0.105/0.2525 | T/0.005/-0.102/0.2474 |
| rs138250198 | 16 |  | [TRIM72 (24026)](http://genome.ucsc.edu/cgi-bin/hgTracks?hgHubConnect.destUrl=..%2Fcgi-bin%2FhgTracks&clade=mammal&org=Human&db=hg19&position=TRIM72) | [ITGAM (9431)](http://genome.ucsc.edu/cgi-bin/hgTracks?hgHubConnect.destUrl=..%2Fcgi-bin%2FhgTracks&clade=mammal&org=Human&db=hg19&position=ITGAM) | T/0.992/-0.129/0.1929 | T/0.993/-0.021/0.8585 | T/0.998/1.315/2.04e-06 | T/0.996/0.553/0.01494 | T/0.008/-0.059/0.4668 | T/0.008/-0.069/0.3865 |
| rs149808359 | 16 | [ITGAX](http://genome.ucsc.edu/cgi-bin/hgTracks?hgHubConnect.destUrl=..%2Fcgi-bin%2FhgTracks&clade=mammal&org=Human&db=hg19&position=ITGAX) | [ITGAM (39914)](http://genome.ucsc.edu/cgi-bin/hgTracks?hgHubConnect.destUrl=..%2Fcgi-bin%2FhgTracks&clade=mammal&org=Human&db=hg19&position=ITGAM) | [ITGAD (20505)](http://genome.ucsc.edu/cgi-bin/hgTracks?hgHubConnect.destUrl=..%2Fcgi-bin%2FhgTracks&clade=mammal&org=Human&db=hg19&position=ITGAD) | G/0.992/-0.044/0.6304 | G/0.992/0.032/0.7625 | G/0.998/1.447/1.2e-07 | G/0.996/0.588/0.00735 | C/0.993/0.108/0.164 | C/0.993/0.111/0.1458 |
| rs139875745 | 16 |  | [ITGAX (2574)](http://genome.ucsc.edu/cgi-bin/hgTracks?hgHubConnect.destUrl=..%2Fcgi-bin%2FhgTracks&clade=mammal&org=Human&db=hg19&position=ITGAX) | [ITGAD (7740)](http://genome.ucsc.edu/cgi-bin/hgTracks?hgHubConnect.destUrl=..%2Fcgi-bin%2FhgTracks&clade=mammal&org=Human&db=hg19&position=ITGAD) | G/0.986/-0.056/0.4534 | G/0.986/0.045/0.6061 | G/0.998/1.381/7.22e-08 | G/0.995/0.526/0.01306 | NA | NA |
| rs145996888 | 16 |  | [C16orf58 (5922)](http://genome.ucsc.edu/cgi-bin/hgTracks?hgHubConnect.destUrl=..%2Fcgi-bin%2FhgTracks&clade=mammal&org=Human&db=hg19&position=C16orf58) | [AHSP (13574)](http://genome.ucsc.edu/cgi-bin/hgTracks?hgHubConnect.destUrl=..%2Fcgi-bin%2FhgTracks&clade=mammal&org=Human&db=hg19&position=AHSP) | T/0.992/-0.095/0.3668 | T/0.992/-0.043/0.713 | T/0.998/1.255/1.94e-06 | T/0.996/0.611/0.0066 | NA | NA |
| rs147174681 | 16 | [SLC6A2](http://genome.ucsc.edu/cgi-bin/hgTracks?hgHubConnect.destUrl=..%2Fcgi-bin%2FhgTracks&clade=mammal&org=Human&db=hg19&position=SLC6A2) | [LPCAT2 (97515)](http://genome.ucsc.edu/cgi-bin/hgTracks?hgHubConnect.destUrl=..%2Fcgi-bin%2FhgTracks&clade=mammal&org=Human&db=hg19&position=LPCAT2) | [CES1P2 (40739)](http://genome.ucsc.edu/cgi-bin/hgTracks?hgHubConnect.destUrl=..%2Fcgi-bin%2FhgTracks&clade=mammal&org=Human&db=hg19&position=CES1P2) | G/1/0.189/0.7077 | G/1/-2.158/0.2102 | G/0.999/2.216/2.7e-06 | G/0.998/1.734/0.00036 | A/1/1.098/0.00022 | A/0.999/0.919/0.00492 |
| rs148560653 | 16 |  |  |  | C/0.984/0.117/0.132 | C/0.984/0.113/0.1882 | C/0.997/0.863/2.47e-06 | C/0.994/0.583/0.00228 | NA | NA |
| rs71403859 | 16 |  | [TAT (29209)](http://genome.ucsc.edu/cgi-bin/hgTracks?hgHubConnect.destUrl=..%2Fcgi-bin%2FhgTracks&clade=mammal&org=Human&db=hg19&position=TAT) | [MARVELD3 (19862)](http://genome.ucsc.edu/cgi-bin/hgTracks?hgHubConnect.destUrl=..%2Fcgi-bin%2FhgTracks&clade=mammal&org=Human&db=hg19&position=MARVELD3) | T/0.851/-0.014/0.4281 | T/0.849/-0.03/0.1435 | T/0.935/0.154/3.07e-06 | T/0.92/0.002/0.9604 | T/0.132/-0.015/0.2535 | T/0.14/0.024/0.08287 |
| rs34381861 | 16 |  | [TAT (29232)](http://genome.ucsc.edu/cgi-bin/hgTracks?hgHubConnect.destUrl=..%2Fcgi-bin%2FhgTracks&clade=mammal&org=Human&db=hg19&position=TAT) | [MARVELD3 (19839)](http://genome.ucsc.edu/cgi-bin/hgTracks?hgHubConnect.destUrl=..%2Fcgi-bin%2FhgTracks&clade=mammal&org=Human&db=hg19&position=MARVELD3) | C/0.849/-0.013/0.4615 | C/0.848/-0.03/0.1486 | C/0.935/0.152/3.88e-06 | C/0.92/0.002/0.9604 | T/0.867/0.016/0.2465 | T/0.859/-0.024/0.07459 |
| rs12927768 | 16 |  | [TAT (34265)](http://genome.ucsc.edu/cgi-bin/hgTracks?hgHubConnect.destUrl=..%2Fcgi-bin%2FhgTracks&clade=mammal&org=Human&db=hg19&position=TAT) | [MARVELD3 (14806)](http://genome.ucsc.edu/cgi-bin/hgTracks?hgHubConnect.destUrl=..%2Fcgi-bin%2FhgTracks&clade=mammal&org=Human&db=hg19&position=MARVELD3) | A/0.855/-0.013/0.4729 | A/0.854/-0.029/0.1605 | A/0.936/0.156/2.14e-06 | A/0.92/0.003/0.9418 | A/0.128/-0.018/0.1852 | A/0.136/0.022/0.107 |
| rs12923781 | 16 |  | [TAT (42466)](http://genome.ucsc.edu/cgi-bin/hgTracks?hgHubConnect.destUrl=..%2Fcgi-bin%2FhgTracks&clade=mammal&org=Human&db=hg19&position=TAT) | [MARVELD3 (6605)](http://genome.ucsc.edu/cgi-bin/hgTracks?hgHubConnect.destUrl=..%2Fcgi-bin%2FhgTracks&clade=mammal&org=Human&db=hg19&position=MARVELD3) | A/0.85/-0.012/0.4911 | A/0.849/-0.029/0.1601 | A/0.936/0.154/2.68e-06 | A/0.922/0.001/0.9754 | A/0.132/-0.017/0.1916 | A/0.14/0.022/0.1096 |
| rs12935422 | 16 | [MARVELD3](http://genome.ucsc.edu/cgi-bin/hgTracks?hgHubConnect.destUrl=..%2Fcgi-bin%2FhgTracks&clade=mammal&org=Human&db=hg19&position=MARVELD3) | [TAT (53227)](http://genome.ucsc.edu/cgi-bin/hgTracks?hgHubConnect.destUrl=..%2Fcgi-bin%2FhgTracks&clade=mammal&org=Human&db=hg19&position=TAT) | [PHLPP2 (7512)](http://genome.ucsc.edu/cgi-bin/hgTracks?hgHubConnect.destUrl=..%2Fcgi-bin%2FhgTracks&clade=mammal&org=Human&db=hg19&position=PHLPP2) | A/0.838/-0.013/0.4547 | A/0.838/-0.024/0.2178 | A/0.935/0.151/3.03e-06 | A/0.92/0/0.995 | A/0.143/-0.019/0.1454 | A/0.151/0.018/0.1597 |
| rs35538253 | 16 | [PHLPP2](http://genome.ucsc.edu/cgi-bin/hgTracks?hgHubConnect.destUrl=..%2Fcgi-bin%2FhgTracks&clade=mammal&org=Human&db=hg19&position=PHLPP2) | [MARVELD3 (2107)](http://genome.ucsc.edu/cgi-bin/hgTracks?hgHubConnect.destUrl=..%2Fcgi-bin%2FhgTracks&clade=mammal&org=Human&db=hg19&position=MARVELD3) | [KIAA0931 (855)](http://genome.ucsc.edu/cgi-bin/hgTracks?hgHubConnect.destUrl=..%2Fcgi-bin%2FhgTracks&clade=mammal&org=Human&db=hg19&position=KIAA0931) | T/0.848/-0.012/0.5329 | T/0.848/-0.033/0.1185 | T/0.937/0.158/2.13e-06 | T/0.921/0.003/0.9522 | T/0.135/-0.021/0.1269 | T/0.144/0.025/0.06549 |
| rs12924650 | 16 | [PHLPP2](http://genome.ucsc.edu/cgi-bin/hgTracks?hgHubConnect.destUrl=..%2Fcgi-bin%2FhgTracks&clade=mammal&org=Human&db=hg19&position=PHLPP2) | [KIAA0931 (24647)](http://genome.ucsc.edu/cgi-bin/hgTracks?hgHubConnect.destUrl=..%2Fcgi-bin%2FhgTracks&clade=mammal&org=Human&db=hg19&position=KIAA0931) | [SNORA70D (26561)](http://genome.ucsc.edu/cgi-bin/hgTracks?hgHubConnect.destUrl=..%2Fcgi-bin%2FhgTracks&clade=mammal&org=Human&db=hg19&position=SNORA70D) | T/0.847/-0.012/0.507 | T/0.846/-0.03/0.1531 | T/0.937/0.158/2.02e-06 | T/0.921/0.002/0.9644 | T/0.136/-0.02/0.1291 | T/0.144/0.021/0.1206 |
| rs12447672 | 16 | [PHLPP2](http://genome.ucsc.edu/cgi-bin/hgTracks?hgHubConnect.destUrl=..%2Fcgi-bin%2FhgTracks&clade=mammal&org=Human&db=hg19&position=PHLPP2) | [KIAA0931 (33870)](http://genome.ucsc.edu/cgi-bin/hgTracks?hgHubConnect.destUrl=..%2Fcgi-bin%2FhgTracks&clade=mammal&org=Human&db=hg19&position=KIAA0931) | [SNORA70D (17338)](http://genome.ucsc.edu/cgi-bin/hgTracks?hgHubConnect.destUrl=..%2Fcgi-bin%2FhgTracks&clade=mammal&org=Human&db=hg19&position=SNORA70D) | A/0.862/-0.012/0.5408 | A/0.86/-0.035/0.1121 | A/0.939/0.164/1.51e-06 | A/0.925/0.01/0.8338 | A/0.122/-0.019/0.1763 | A/0.13/0.025/0.08206 |
| rs17355804 | 16 | [PHLPP2](http://genome.ucsc.edu/cgi-bin/hgTracks?hgHubConnect.destUrl=..%2Fcgi-bin%2FhgTracks&clade=mammal&org=Human&db=hg19&position=PHLPP2) | [KIAA0931 (35953)](http://genome.ucsc.edu/cgi-bin/hgTracks?hgHubConnect.destUrl=..%2Fcgi-bin%2FhgTracks&clade=mammal&org=Human&db=hg19&position=KIAA0931) | [SNORA70D (15255)](http://genome.ucsc.edu/cgi-bin/hgTracks?hgHubConnect.destUrl=..%2Fcgi-bin%2FhgTracks&clade=mammal&org=Human&db=hg19&position=SNORA70D) | G/0.856/-0.007/0.7088 | G/0.854/-0.036/0.09683 | G/0.929/0.152/2.49e-06 | G/0.913/-0.007/0.8687 | A/0.872/0.021/0.1344 | A/0.864/-0.028/0.04985 |
| rs12149264 | 16 | [PHLPP2](http://genome.ucsc.edu/cgi-bin/hgTracks?hgHubConnect.destUrl=..%2Fcgi-bin%2FhgTracks&clade=mammal&org=Human&db=hg19&position=PHLPP2) | [KIAA0931 (39660)](http://genome.ucsc.edu/cgi-bin/hgTracks?hgHubConnect.destUrl=..%2Fcgi-bin%2FhgTracks&clade=mammal&org=Human&db=hg19&position=KIAA0931) | [SNORA70D (11548)](http://genome.ucsc.edu/cgi-bin/hgTracks?hgHubConnect.destUrl=..%2Fcgi-bin%2FhgTracks&clade=mammal&org=Human&db=hg19&position=SNORA70D) | G/0.844/-0.012/0.5115 | G/0.844/-0.033/0.1175 | G/0.936/0.156/2.7e-06 | G/0.92/-0.009/0.8356 | T/0.861/0.02/0.1476 | T/0.853/-0.025/0.07002 |
| rs17356206 | 16 | [PHLPP2](http://genome.ucsc.edu/cgi-bin/hgTracks?hgHubConnect.destUrl=..%2Fcgi-bin%2FhgTracks&clade=mammal&org=Human&db=hg19&position=PHLPP2) | [SNORA70D (3755)](http://genome.ucsc.edu/cgi-bin/hgTracks?hgHubConnect.destUrl=..%2Fcgi-bin%2FhgTracks&clade=mammal&org=Human&db=hg19&position=SNORA70D) | [AP1G1 (26545)](http://genome.ucsc.edu/cgi-bin/hgTracks?hgHubConnect.destUrl=..%2Fcgi-bin%2FhgTracks&clade=mammal&org=Human&db=hg19&position=AP1G1) | C/0.857/-0.008/0.6897 | C/0.856/-0.036/0.09934 | C/0.939/0.162/1.78e-06 | C/0.924/0.007/0.8808 | C/0.13/-0.024/0.08866 | C/0.139/0.026/0.06178 |
| rs12920890 | 16 | [AP1G1](http://genome.ucsc.edu/cgi-bin/hgTracks?hgHubConnect.destUrl=..%2Fcgi-bin%2FhgTracks&clade=mammal&org=Human&db=hg19&position=AP1G1) | [PHLPP2 (29873)](http://genome.ucsc.edu/cgi-bin/hgTracks?hgHubConnect.destUrl=..%2Fcgi-bin%2FhgTracks&clade=mammal&org=Human&db=hg19&position=PHLPP2) | [SNORD71 (4716)](http://genome.ucsc.edu/cgi-bin/hgTracks?hgHubConnect.destUrl=..%2Fcgi-bin%2FhgTracks&clade=mammal&org=Human&db=hg19&position=SNORD71) | A/0.848/-0.017/0.3617 | A/0.85/-0.032/0.1386 | A/0.938/0.158/2.07e-06 | A/0.923/-0.001/0.9828 | A/0.135/-0.02/0.1464 | A/0.143/0.022/0.1171 |
| rs12928939 | 16 | [AP1G1](http://genome.ucsc.edu/cgi-bin/hgTracks?hgHubConnect.destUrl=..%2Fcgi-bin%2FhgTracks&clade=mammal&org=Human&db=hg19&position=AP1G1) | [SNORD71 (7655)](http://genome.ucsc.edu/cgi-bin/hgTracks?hgHubConnect.destUrl=..%2Fcgi-bin%2FhgTracks&clade=mammal&org=Human&db=hg19&position=SNORD71) | [ATXN1L (79853)](http://genome.ucsc.edu/cgi-bin/hgTracks?hgHubConnect.destUrl=..%2Fcgi-bin%2FhgTracks&clade=mammal&org=Human&db=hg19&position=ATXN1L) | G/0.294/0.023/0.1032 | G/0.294/0.036/0.02355 | G/0.107/-0.118/4.2e-06 | G/0.107/-0.027/0.4523 | A/0.612/-0.005/0.6228 | A/0.518/0.02/0.05988 |
| rs12149533 | 16 | [AP1G1](http://genome.ucsc.edu/cgi-bin/hgTracks?hgHubConnect.destUrl=..%2Fcgi-bin%2FhgTracks&clade=mammal&org=Human&db=hg19&position=AP1G1) | [SNORD71 (9556)](http://genome.ucsc.edu/cgi-bin/hgTracks?hgHubConnect.destUrl=..%2Fcgi-bin%2FhgTracks&clade=mammal&org=Human&db=hg19&position=SNORD71) | [ATXN1L (77952)](http://genome.ucsc.edu/cgi-bin/hgTracks?hgHubConnect.destUrl=..%2Fcgi-bin%2FhgTracks&clade=mammal&org=Human&db=hg19&position=ATXN1L) | T/0.859/-0.014/0.488 | T/0.858/-0.036/0.1067 | T/0.939/0.158/2.73e-06 | T/0.926/0.008/0.87 | T/0.124/-0.019/0.1921 | T/0.131/0.025/0.08307 |
| rs71386933 | 16 | [AP1G1](http://genome.ucsc.edu/cgi-bin/hgTracks?hgHubConnect.destUrl=..%2Fcgi-bin%2FhgTracks&clade=mammal&org=Human&db=hg19&position=AP1G1) | [SNORD71 (29410)](http://genome.ucsc.edu/cgi-bin/hgTracks?hgHubConnect.destUrl=..%2Fcgi-bin%2FhgTracks&clade=mammal&org=Human&db=hg19&position=SNORD71) | [ATXN1L (58098)](http://genome.ucsc.edu/cgi-bin/hgTracks?hgHubConnect.destUrl=..%2Fcgi-bin%2FhgTracks&clade=mammal&org=Human&db=hg19&position=ATXN1L) | T/0.849/-0.009/0.6219 | T/0.848/-0.037/0.09243 | T/0.937/0.164/1.29e-06 | T/0.923/0.015/0.755 | A/0.868/0.019/0.1786 | A/0.86/-0.027/0.0557 |
| rs12444156 | 16 | [AP1G1](http://genome.ucsc.edu/cgi-bin/hgTracks?hgHubConnect.destUrl=..%2Fcgi-bin%2FhgTracks&clade=mammal&org=Human&db=hg19&position=AP1G1) | [SNORD71 (48867)](http://genome.ucsc.edu/cgi-bin/hgTracks?hgHubConnect.destUrl=..%2Fcgi-bin%2FhgTracks&clade=mammal&org=Human&db=hg19&position=SNORD71) | [ATXN1L (38641)](http://genome.ucsc.edu/cgi-bin/hgTracks?hgHubConnect.destUrl=..%2Fcgi-bin%2FhgTracks&clade=mammal&org=Human&db=hg19&position=ATXN1L) | T/0.854/-0.009/0.6301 | T/0.853/-0.037/0.08777 | T/0.938/0.156/4.32e-06 | T/0.924/0.005/0.9158 | T/0.128/-0.019/0.1851 | T/0.136/0.028/0.05074 |
| rs181167431 | 16 |  |  |  | A/0.995/0.064/0.54 | A/0.994/-0.012/0.9149 | A/1/2.522/1.14e-06 | A/0.999/1.199/0.00286 | NA | NA |
| rs191151465 | 16 |  | [ADAMTS18 (196670)](http://genome.ucsc.edu/cgi-bin/hgTracks?hgHubConnect.destUrl=..%2Fcgi-bin%2FhgTracks&clade=mammal&org=Human&db=hg19&position=ADAMTS18) | [NUDT7 (161936)](http://genome.ucsc.edu/cgi-bin/hgTracks?hgHubConnect.destUrl=..%2Fcgi-bin%2FhgTracks&clade=mammal&org=Human&db=hg19&position=NUDT7) | C/0.988/0.002/0.9739 | C/0.99/0.113/0.1914 | C/0.999/2.215/4.76e-06 | C/0.998/1.08/0.01452 | T/0.988/-0.01/0.8534 | T/0.989/0.034/0.5531 |
| rs142313660 | 16 | [GAN](http://genome.ucsc.edu/cgi-bin/hgTracks?hgHubConnect.destUrl=..%2Fcgi-bin%2FhgTracks&clade=mammal&org=Human&db=hg19&position=GAN) | [BCMO1 (45393)](http://genome.ucsc.edu/cgi-bin/hgTracks?hgHubConnect.destUrl=..%2Fcgi-bin%2FhgTracks&clade=mammal&org=Human&db=hg19&position=BCMO1) | [MIR4720 (48482)](http://genome.ucsc.edu/cgi-bin/hgTracks?hgHubConnect.destUrl=..%2Fcgi-bin%2FhgTracks&clade=mammal&org=Human&db=hg19&position=MIR4720) | NA | NA | T/0.998/1.264/9.87e-07 | T/0.997/0.676/0.01027 | NA | NA |
| rs28497028 | 16 | [GAN](http://genome.ucsc.edu/cgi-bin/hgTracks?hgHubConnect.destUrl=..%2Fcgi-bin%2FhgTracks&clade=mammal&org=Human&db=hg19&position=GAN) | [BCMO1 (47843)](http://genome.ucsc.edu/cgi-bin/hgTracks?hgHubConnect.destUrl=..%2Fcgi-bin%2FhgTracks&clade=mammal&org=Human&db=hg19&position=BCMO1) | [MIR4720 (46032)](http://genome.ucsc.edu/cgi-bin/hgTracks?hgHubConnect.destUrl=..%2Fcgi-bin%2FhgTracks&clade=mammal&org=Human&db=hg19&position=MIR4720) | NA | NA | A/0.998/1.097/8.6e-07 | A/0.997/0.589/0.00956 | NA | NA |
| rs143522076 | 16 | [GAN](http://genome.ucsc.edu/cgi-bin/hgTracks?hgHubConnect.destUrl=..%2Fcgi-bin%2FhgTracks&clade=mammal&org=Human&db=hg19&position=GAN) | [BCMO1 (51105)](http://genome.ucsc.edu/cgi-bin/hgTracks?hgHubConnect.destUrl=..%2Fcgi-bin%2FhgTracks&clade=mammal&org=Human&db=hg19&position=BCMO1) | [MIR4720 (42770)](http://genome.ucsc.edu/cgi-bin/hgTracks?hgHubConnect.destUrl=..%2Fcgi-bin%2FhgTracks&clade=mammal&org=Human&db=hg19&position=MIR4720) | NA | NA | G/0.998/1.091/8.26e-07 | G/0.997/0.588/0.00921 | NA | NA |
| rs67163887 | 16 | [JPH3](http://genome.ucsc.edu/cgi-bin/hgTracks?hgHubConnect.destUrl=..%2Fcgi-bin%2FhgTracks&clade=mammal&org=Human&db=hg19&position=JPH3) | [BC131758 (144805)](http://genome.ucsc.edu/cgi-bin/hgTracks?hgHubConnect.destUrl=..%2Fcgi-bin%2FhgTracks&clade=mammal&org=Human&db=hg19&position=BC131758) | [FLJ00104 (37712)](http://genome.ucsc.edu/cgi-bin/hgTracks?hgHubConnect.destUrl=..%2Fcgi-bin%2FhgTracks&clade=mammal&org=Human&db=hg19&position=FLJ00104) | G/0.894/0.016/0.4873 | G/0.894/0.004/0.8813 | G/0.917/0.146/3.81e-06 | G/0.903/0.108/0.01628 | C/0.901/0.054/0.00067 | C/0.895/0.028/0.09177 |
| rs78507937 | 17 |  | [CLEC10A (4241)](http://genome.ucsc.edu/cgi-bin/hgTracks?hgHubConnect.destUrl=..%2Fcgi-bin%2FhgTracks&clade=mammal&org=Human&db=hg19&position=CLEC10A) | [ASGR2 (16799)](http://genome.ucsc.edu/cgi-bin/hgTracks?hgHubConnect.destUrl=..%2Fcgi-bin%2FhgTracks&clade=mammal&org=Human&db=hg19&position=ASGR2) | NA | NA | T/0.982/0.298/5.87e-07 | T/0.974/0.129/0.09987 | NA | NA |
| rs188840013 | 17 |  | [KIF2B (89450)](http://genome.ucsc.edu/cgi-bin/hgTracks?hgHubConnect.destUrl=..%2Fcgi-bin%2FhgTracks&clade=mammal&org=Human&db=hg19&position=KIF2B) |  | C/0.996/-0.158/0.2413 | C/0.997/-0.042/0.7976 | C/0.998/1.385/3.02e-06 | C/0.997/0.644/0.02122 | T/0.996/-0.03/0.756 | T/0.996/0.127/0.1811 |
| rs8079658 | 17 | [CEP112](http://genome.ucsc.edu/cgi-bin/hgTracks?hgHubConnect.destUrl=..%2Fcgi-bin%2FhgTracks&clade=mammal&org=Human&db=hg19&position=CEP112) | [AXIN2 (236629)](http://genome.ucsc.edu/cgi-bin/hgTracks?hgHubConnect.destUrl=..%2Fcgi-bin%2FhgTracks&clade=mammal&org=Human&db=hg19&position=AXIN2) |  | A/0.753/-0.009/0.5614 | A/0.751/-0.01/0.5518 | A/0.793/-0.091/4.99e-06 | A/0.817/-0.086/0.00774 | A/0.239/0.022/0.03429 | A/0.243/0.006/0.5973 |
| rs9904045 | 17 | [CEP112](http://genome.ucsc.edu/cgi-bin/hgTracks?hgHubConnect.destUrl=..%2Fcgi-bin%2FhgTracks&clade=mammal&org=Human&db=hg19&position=CEP112) |  | [APOH (156420)](http://genome.ucsc.edu/cgi-bin/hgTracks?hgHubConnect.destUrl=..%2Fcgi-bin%2FhgTracks&clade=mammal&org=Human&db=hg19&position=APOH) | NA | NA | T/0.997/0.884/2.19e-06 | T/0.994/0.49/0.01323 | NA | NA |
| rs148133787 | 18 |  |  |  | NA | NA | C/0.995/0.682/1.51e-06 | C/0.992/0.714/4.43e-05 | NA | NA |
| rs145717157 | 18 |  |  |  | NA | NA | T/0.999/1.584/4.61e-06 | T/0.998/0.986/0.00248 | NA | NA |
| rs190343823 | 18 | [MOCOS](http://genome.ucsc.edu/cgi-bin/hgTracks?hgHubConnect.destUrl=..%2Fcgi-bin%2FhgTracks&clade=mammal&org=Human&db=hg19&position=MOCOS) | [BC009735 (67938)](http://genome.ucsc.edu/cgi-bin/hgTracks?hgHubConnect.destUrl=..%2Fcgi-bin%2FhgTracks&clade=mammal&org=Human&db=hg19&position=BC009735) | [FHOD3 (42365)](http://genome.ucsc.edu/cgi-bin/hgTracks?hgHubConnect.destUrl=..%2Fcgi-bin%2FhgTracks&clade=mammal&org=Human&db=hg19&position=FHOD3) | NA | NA | C/0.999/2.482/3.24e-06 | C/0.999/1.431/0.00108 | NA | NA |
| rs186640425 | 19 | [FCER2](http://genome.ucsc.edu/cgi-bin/hgTracks?hgHubConnect.destUrl=..%2Fcgi-bin%2FhgTracks&clade=mammal&org=Human&db=hg19&position=FCER2) | [TRAPPC5 (19069)](http://genome.ucsc.edu/cgi-bin/hgTracks?hgHubConnect.destUrl=..%2Fcgi-bin%2FhgTracks&clade=mammal&org=Human&db=hg19&position=TRAPPC5) | [CLEC4G (27025)](http://genome.ucsc.edu/cgi-bin/hgTracks?hgHubConnect.destUrl=..%2Fcgi-bin%2FhgTracks&clade=mammal&org=Human&db=hg19&position=CLEC4G) | NA | NA | G/0.999/1.619/2.05e-06 | G/0.998/1.016/0.00103 | NA | NA |
| rs187229201 | 19 |  | [FCER2 (6843)](http://genome.ucsc.edu/cgi-bin/hgTracks?hgHubConnect.destUrl=..%2Fcgi-bin%2FhgTracks&clade=mammal&org=Human&db=hg19&position=FCER2) | [CLEC4G (19967)](http://genome.ucsc.edu/cgi-bin/hgTracks?hgHubConnect.destUrl=..%2Fcgi-bin%2FhgTracks&clade=mammal&org=Human&db=hg19&position=CLEC4G) | NA | NA | G/0.999/1.614/1.17e-06 | G/0.998/0.957/0.00152 | NA | NA |
| rs146861169 | 19 |  | [FCER2 (25211)](http://genome.ucsc.edu/cgi-bin/hgTracks?hgHubConnect.destUrl=..%2Fcgi-bin%2FhgTracks&clade=mammal&org=Human&db=hg19&position=FCER2) | [CLEC4G (1599)](http://genome.ucsc.edu/cgi-bin/hgTracks?hgHubConnect.destUrl=..%2Fcgi-bin%2FhgTracks&clade=mammal&org=Human&db=hg19&position=CLEC4G) | NA | NA | T/0.999/1.465/2.14e-06 | T/0.997/0.718/0.00914 | NA | NA |
| rs142080098 | 19 | [CLEC4G](http://genome.ucsc.edu/cgi-bin/hgTracks?hgHubConnect.destUrl=..%2Fcgi-bin%2FhgTracks&clade=mammal&org=Human&db=hg19&position=CLEC4G) | [FCER2 (29518)](http://genome.ucsc.edu/cgi-bin/hgTracks?hgHubConnect.destUrl=..%2Fcgi-bin%2FhgTracks&clade=mammal&org=Human&db=hg19&position=FCER2) | [CD209 (8330)](http://genome.ucsc.edu/cgi-bin/hgTracks?hgHubConnect.destUrl=..%2Fcgi-bin%2FhgTracks&clade=mammal&org=Human&db=hg19&position=CD209) | NA | NA | C/0.999/1.455/2.28e-06 | C/0.997/0.709/0.0097 | NA | NA |
| rs144975701 | 19 |  | [CLEC4G (3409)](http://genome.ucsc.edu/cgi-bin/hgTracks?hgHubConnect.destUrl=..%2Fcgi-bin%2FhgTracks&clade=mammal&org=Human&db=hg19&position=CLEC4G) | [CD209 (4414)](http://genome.ucsc.edu/cgi-bin/hgTracks?hgHubConnect.destUrl=..%2Fcgi-bin%2FhgTracks&clade=mammal&org=Human&db=hg19&position=CD209) | NA | NA | T/0.999/1.451/2.36e-06 | T/0.997/0.703/0.01025 | NA | NA |
| rs75185364 | 19 | [PRKD2](http://genome.ucsc.edu/cgi-bin/hgTracks?hgHubConnect.destUrl=..%2Fcgi-bin%2FhgTracks&clade=mammal&org=Human&db=hg19&position=PRKD2) | [LOC100506068 (12228)](http://genome.ucsc.edu/cgi-bin/hgTracks?hgHubConnect.destUrl=..%2Fcgi-bin%2FhgTracks&clade=mammal&org=Human&db=hg19&position=LOC100506068) | [MIR320E (19608)](http://genome.ucsc.edu/cgi-bin/hgTracks?hgHubConnect.destUrl=..%2Fcgi-bin%2FhgTracks&clade=mammal&org=Human&db=hg19&position=MIR320E) | NA | NA | C/0.976/0.329/1.12e-06 | C/0.97/0.27/0.00189 | NA | NA |
| rs114855972 | 19 | [PRKD2](http://genome.ucsc.edu/cgi-bin/hgTracks?hgHubConnect.destUrl=..%2Fcgi-bin%2FhgTracks&clade=mammal&org=Human&db=hg19&position=PRKD2) | [LOC100506068 (14173)](http://genome.ucsc.edu/cgi-bin/hgTracks?hgHubConnect.destUrl=..%2Fcgi-bin%2FhgTracks&clade=mammal&org=Human&db=hg19&position=LOC100506068) | [MIR320E (17663)](http://genome.ucsc.edu/cgi-bin/hgTracks?hgHubConnect.destUrl=..%2Fcgi-bin%2FhgTracks&clade=mammal&org=Human&db=hg19&position=MIR320E) | NA | NA | A/0.976/0.329/1.11e-06 | A/0.97/0.27/0.00189 | NA | NA |
| rs147565295 | 19 | [PRKD2](http://genome.ucsc.edu/cgi-bin/hgTracks?hgHubConnect.destUrl=..%2Fcgi-bin%2FhgTracks&clade=mammal&org=Human&db=hg19&position=PRKD2) | [LOC100506068 (14542)](http://genome.ucsc.edu/cgi-bin/hgTracks?hgHubConnect.destUrl=..%2Fcgi-bin%2FhgTracks&clade=mammal&org=Human&db=hg19&position=LOC100506068) | [MIR320E (17294)](http://genome.ucsc.edu/cgi-bin/hgTracks?hgHubConnect.destUrl=..%2Fcgi-bin%2FhgTracks&clade=mammal&org=Human&db=hg19&position=MIR320E) | NA | NA | A/0.976/0.329/1.09e-06 | A/0.97/0.27/0.00187 | NA | NA |
| rs138814219 | 19 | [PRKD2](http://genome.ucsc.edu/cgi-bin/hgTracks?hgHubConnect.destUrl=..%2Fcgi-bin%2FhgTracks&clade=mammal&org=Human&db=hg19&position=PRKD2) | [LOC100506068 (15933)](http://genome.ucsc.edu/cgi-bin/hgTracks?hgHubConnect.destUrl=..%2Fcgi-bin%2FhgTracks&clade=mammal&org=Human&db=hg19&position=LOC100506068) | [MIR320E (15903)](http://genome.ucsc.edu/cgi-bin/hgTracks?hgHubConnect.destUrl=..%2Fcgi-bin%2FhgTracks&clade=mammal&org=Human&db=hg19&position=MIR320E) | NA | NA | T/0.976/0.336/7.32e-07 | T/0.97/0.276/0.00166 | NA | NA |
| rs139399000 | 19 | [PRKD2](http://genome.ucsc.edu/cgi-bin/hgTracks?hgHubConnect.destUrl=..%2Fcgi-bin%2FhgTracks&clade=mammal&org=Human&db=hg19&position=PRKD2) | [LOC100506068 (23728)](http://genome.ucsc.edu/cgi-bin/hgTracks?hgHubConnect.destUrl=..%2Fcgi-bin%2FhgTracks&clade=mammal&org=Human&db=hg19&position=LOC100506068) | [MIR320E (8108)](http://genome.ucsc.edu/cgi-bin/hgTracks?hgHubConnect.destUrl=..%2Fcgi-bin%2FhgTracks&clade=mammal&org=Human&db=hg19&position=MIR320E) | NA | NA | G/0.977/0.329/1.07e-06 | G/0.971/0.27/0.002 | NA | NA |
| rs115824942 | 19 | [PRKD2](http://genome.ucsc.edu/cgi-bin/hgTracks?hgHubConnect.destUrl=..%2Fcgi-bin%2FhgTracks&clade=mammal&org=Human&db=hg19&position=PRKD2) | [LOC100506068 (27669)](http://genome.ucsc.edu/cgi-bin/hgTracks?hgHubConnect.destUrl=..%2Fcgi-bin%2FhgTracks&clade=mammal&org=Human&db=hg19&position=LOC100506068) | [MIR320E (4167)](http://genome.ucsc.edu/cgi-bin/hgTracks?hgHubConnect.destUrl=..%2Fcgi-bin%2FhgTracks&clade=mammal&org=Human&db=hg19&position=MIR320E) | NA | NA | T/0.977/0.329/9.98e-07 | T/0.971/0.27/0.00196 | NA | NA |
| rs151060619 | 19 | [PRKD2](http://genome.ucsc.edu/cgi-bin/hgTracks?hgHubConnect.destUrl=..%2Fcgi-bin%2FhgTracks&clade=mammal&org=Human&db=hg19&position=PRKD2) | [MIR320E (709)](http://genome.ucsc.edu/cgi-bin/hgTracks?hgHubConnect.destUrl=..%2Fcgi-bin%2FhgTracks&clade=mammal&org=Human&db=hg19&position=MIR320E) | [STRN4 (9456)](http://genome.ucsc.edu/cgi-bin/hgTracks?hgHubConnect.destUrl=..%2Fcgi-bin%2FhgTracks&clade=mammal&org=Human&db=hg19&position=STRN4) | NA | NA | A/0.976/0.322/1.27e-06 | A/0.97/0.26/0.00243 | NA | NA |
| rs182429428 | 20 |  | [BTBD3 (767)](http://genome.ucsc.edu/cgi-bin/hgTracks?hgHubConnect.destUrl=..%2Fcgi-bin%2FhgTracks&clade=mammal&org=Human&db=hg19&position=BTBD3) |  | NA | NA | C/0.999/2.627/3.12e-06 | C/0.999/1.155/0.00911 | NA | NA |
| rs184757371 | 20 |  | [BTBD3 (11682)](http://genome.ucsc.edu/cgi-bin/hgTracks?hgHubConnect.destUrl=..%2Fcgi-bin%2FhgTracks&clade=mammal&org=Human&db=hg19&position=BTBD3) |  | NA | NA | A/0.999/2.679/2.87e-06 | A/0.999/1.181/0.0087 | NA | NA |
| rs144394173 | 20 |  | [LINC00494 (125573)](http://genome.ucsc.edu/cgi-bin/hgTracks?hgHubConnect.destUrl=..%2Fcgi-bin%2FhgTracks&clade=mammal&org=Human&db=hg19&position=LINC00494) | [AX746653 (115838)](http://genome.ucsc.edu/cgi-bin/hgTracks?hgHubConnect.destUrl=..%2Fcgi-bin%2FhgTracks&clade=mammal&org=Human&db=hg19&position=AX746653) | T/0.998/-0.307/0.1413 | T/0.999/0.609/0.1041 | T/0.998/1.569/3.81e-06 | T/0.997/0.606/0.06771 | NA | NA |
| rs181124765 | 20 |  | [BCAS1 (24136)](http://genome.ucsc.edu/cgi-bin/hgTracks?hgHubConnect.destUrl=..%2Fcgi-bin%2FhgTracks&clade=mammal&org=Human&db=hg19&position=BCAS1) | [CYP24A1 (58547)](http://genome.ucsc.edu/cgi-bin/hgTracks?hgHubConnect.destUrl=..%2Fcgi-bin%2FhgTracks&clade=mammal&org=Human&db=hg19&position=CYP24A1) | G/0.994/-0.066/0.499 | G/0.994/-0.081/0.4509 | G/0.999/1.829/1.75e-06 | G/0.998/0.882/0.00813 | A/0.994/0.043/0.5776 | A/0.995/-0.004/0.9572 |
| rs188695430 | 20 | [CYP24A1](http://genome.ucsc.edu/cgi-bin/hgTracks?hgHubConnect.destUrl=..%2Fcgi-bin%2FhgTracks&clade=mammal&org=Human&db=hg19&position=CYP24A1) | [BCAS1 (98962)](http://genome.ucsc.edu/cgi-bin/hgTracks?hgHubConnect.destUrl=..%2Fcgi-bin%2FhgTracks&clade=mammal&org=Human&db=hg19&position=BCAS1) |  | T/0.995/-0.051/0.6158 | T/0.995/-0.091/0.4044 | T/0.999/1.705/2.66e-06 | T/0.998/0.811/0.01252 | T/0.005/-0.057/0.4824 | T/0.005/0.007/0.9281 |
| rs142429672 | 21 |  |  |  | NA | NA | C/0.999/1.187/4.3e-06 | C/0.996/0.396/0.04985 | NA | NA |
| rs141838403 | 21 |  |  |  | NA | NA | C/0.999/1.249/2.68e-06 | C/0.996/0.391/0.05806 | NA | NA |
| rs139202427 | 21 |  |  |  | NA | NA | A/0.999/1.348/2.24e-06 | A/0.996/0.457/0.04091 | NA | NA |
| rs142976294 | 21 |  |  |  | NA | NA | G/0.999/1.415/2.31e-06 | G/0.996/0.48/0.04142 | NA | NA |
| rs149318287 | 22 | [LARGE](http://genome.ucsc.edu/cgi-bin/hgTracks?hgHubConnect.destUrl=..%2Fcgi-bin%2FhgTracks&clade=mammal&org=Human&db=hg19&position=LARGE) |  |  | A/0.985/0.065/0.2749 | A/0.984/-0.029/0.6542 | A/0.995/0.65/1.62e-06 | A/0.992/0.644/3.04e-05 | A/0.014/-0.127/0.00479 | A/0.015/-0.07/0.112 |
| rs150663730 | 22 | [LARGE](http://genome.ucsc.edu/cgi-bin/hgTracks?hgHubConnect.destUrl=..%2Fcgi-bin%2FhgTracks&clade=mammal&org=Human&db=hg19&position=LARGE) |  |  | A/0.985/0.064/0.285 | A/0.984/-0.03/0.6432 | A/0.995/0.607/2.15e-06 | A/0.991/0.547/0.00014 | A/0.014/-0.128/0.00423 | A/0.015/-0.068/0.1199 |
| rs5757673 | 22 |  | [LOC100506472 (4787)](http://genome.ucsc.edu/cgi-bin/hgTracks?hgHubConnect.destUrl=..%2Fcgi-bin%2FhgTracks&clade=mammal&org=Human&db=hg19&position=LOC100506472) | [MGAT3 (15404)](http://genome.ucsc.edu/cgi-bin/hgTracks?hgHubConnect.destUrl=..%2Fcgi-bin%2FhgTracks&clade=mammal&org=Human&db=hg19&position=MGAT3) | T/0.702/-0.012/0.4126 | T/0.701/-0.026/0.1136 | T/0.878/0.126/1.05e-06 | T/0.851/0.067/0.06901 | T/0.266/-0.015/0.1473 | T/0.286/0.009/0.4087 |
| rs909674 | 22 | [MGAT3](http://genome.ucsc.edu/cgi-bin/hgTracks?hgHubConnect.destUrl=..%2Fcgi-bin%2FhgTracks&clade=mammal&org=Human&db=hg19&position=MGAT3) | [LOC100506472 (26036)](http://genome.ucsc.edu/cgi-bin/hgTracks?hgHubConnect.destUrl=..%2Fcgi-bin%2FhgTracks&clade=mammal&org=Human&db=hg19&position=LOC100506472) | [SMCR7L (36935)](http://genome.ucsc.edu/cgi-bin/hgTracks?hgHubConnect.destUrl=..%2Fcgi-bin%2FhgTracks&clade=mammal&org=Human&db=hg19&position=SMCR7L) | C/0.724/-0.005/0.7235 | C/0.72/-0.022/0.18 | C/0.926/0.143/2.4e-06 | C/0.905/0.052/0.2104 | A/0.603/0.015/0.1706 | A/0.507/-0.01/0.3406 |
| rs2008174 | 22 | [MGAT3](http://genome.ucsc.edu/cgi-bin/hgTracks?hgHubConnect.destUrl=..%2Fcgi-bin%2FhgTracks&clade=mammal&org=Human&db=hg19&position=MGAT3) | [LOC100506472 (26997)](http://genome.ucsc.edu/cgi-bin/hgTracks?hgHubConnect.destUrl=..%2Fcgi-bin%2FhgTracks&clade=mammal&org=Human&db=hg19&position=LOC100506472) | [SMCR7L (35974)](http://genome.ucsc.edu/cgi-bin/hgTracks?hgHubConnect.destUrl=..%2Fcgi-bin%2FhgTracks&clade=mammal&org=Human&db=hg19&position=SMCR7L) | C/0.722/-0.008/0.577 | C/0.719/-0.021/0.2032 | C/0.925/0.142/3.17e-06 | C/0.904/0.052/0.2104 | T/0.748/0.012/0.2602 | T/0.731/-0.011/0.3126 |
| rs3959642 | 22 | [MGAT3](http://genome.ucsc.edu/cgi-bin/hgTracks?hgHubConnect.destUrl=..%2Fcgi-bin%2FhgTracks&clade=mammal&org=Human&db=hg19&position=MGAT3) | [LOC100506472 (27456)](http://genome.ucsc.edu/cgi-bin/hgTracks?hgHubConnect.destUrl=..%2Fcgi-bin%2FhgTracks&clade=mammal&org=Human&db=hg19&position=LOC100506472) | [SMCR7L (35515)](http://genome.ucsc.edu/cgi-bin/hgTracks?hgHubConnect.destUrl=..%2Fcgi-bin%2FhgTracks&clade=mammal&org=Human&db=hg19&position=SMCR7L) | A/0.723/-0.006/0.704 | A/0.72/-0.022/0.184 | A/0.912/0.149/1.11e-07 | A/0.886/0.075/0.05275 | A/0.249/-0.018/0.09349 | A/0.268/0.008/0.4526 |
| rs7286917 | 22 | [MGAT3](http://genome.ucsc.edu/cgi-bin/hgTracks?hgHubConnect.destUrl=..%2Fcgi-bin%2FhgTracks&clade=mammal&org=Human&db=hg19&position=MGAT3) | [LOC100506472 (27735)](http://genome.ucsc.edu/cgi-bin/hgTracks?hgHubConnect.destUrl=..%2Fcgi-bin%2FhgTracks&clade=mammal&org=Human&db=hg19&position=LOC100506472) | [SMCR7L (35236)](http://genome.ucsc.edu/cgi-bin/hgTracks?hgHubConnect.destUrl=..%2Fcgi-bin%2FhgTracks&clade=mammal&org=Human&db=hg19&position=SMCR7L) | A/0.718/-0.006/0.7068 | A/0.715/-0.023/0.1702 | A/0.898/0.133/1.33e-06 | A/0.878/0.062/0.1082 | A/0.254/-0.017/0.1198 | A/0.273/0.008/0.4665 |

**Table S3:** Top SNPs with a p-value < 5E-06 for FEV1/FVC among AA cases in COPDGene.

| SNP | Chr | Gene | Nearest Gene Up | Nearest Gene Down | | COPDGene NHW All Subjects | COPDGene NHW Cases Only | COPDGene AA All Subjects | COPDGene AA Cases Only | Meta-analysis All subjects | Meta-analysis Cases Only |
| --- | --- | --- | --- | --- | --- | --- | --- | --- | --- | --- | --- |
| Coded Allele /Beta/P | Coded Allele /Beta/P | Coded Allele /Beta/P | Coded Allele / Beta/ P | Coded Allele / Beta/ P | Coded Allele / Beta/ P |
| rs143262451 | 1 |  | [DR1 (45307)](http://genome.ucsc.edu/cgi-bin/hgTracks?hgHubConnect.destUrl=..%2Fcgi-bin%2FhgTracks&clade=mammal&org=Human&db=hg19&position=DR1) | [FNBP1L (40232)](http://genome.ucsc.edu/cgi-bin/hgTracks?hgHubConnect.destUrl=..%2Fcgi-bin%2FhgTracks&clade=mammal&org=Human&db=hg19&position=FNBP1L) | | NA | NA | A/0.996/0.069/0.08893 | A/0.996/0.353/7.28e-07 | NA | NA |
| rs77928077 | 1 | [MPZL1](http://genome.ucsc.edu/cgi-bin/hgTracks?hgHubConnect.destUrl=..%2Fcgi-bin%2FhgTracks&clade=mammal&org=Human&db=hg19&position=MPZL1) | [RCSD1 (55975)](http://genome.ucsc.edu/cgi-bin/hgTracks?hgHubConnect.destUrl=..%2Fcgi-bin%2FhgTracks&clade=mammal&org=Human&db=hg19&position=RCSD1) | [ADCY10 (47163)](http://genome.ucsc.edu/cgi-bin/hgTracks?hgHubConnect.destUrl=..%2Fcgi-bin%2FhgTracks&clade=mammal&org=Human&db=hg19&position=ADCY10) | NA | | NA | G/0.894/0.01/0.04306 | G/0.896/0.043/4.67e-06 | NA | NA |
| rs144177292 | 1 | [RNPEP](http://genome.ucsc.edu/cgi-bin/hgTracks?hgHubConnect.destUrl=..%2Fcgi-bin%2FhgTracks&clade=mammal&org=Human&db=hg19&position=RNPEP) | [TIMM17A (24270)](http://genome.ucsc.edu/cgi-bin/hgTracks?hgHubConnect.destUrl=..%2Fcgi-bin%2FhgTracks&clade=mammal&org=Human&db=hg19&position=TIMM17A) | [ELF3 (13013)](http://genome.ucsc.edu/cgi-bin/hgTracks?hgHubConnect.destUrl=..%2Fcgi-bin%2FhgTracks&clade=mammal&org=Human&db=hg19&position=ELF3) | T/0.994/-0.033/0.1556 | | T/0.995/-0.023/0.492 | T/0.986/0.018/0.2556 | T/0.99/0.178/1.03e-06 | T/0.011/-0.002/0.826 | T/0.007/0.008/0.6908 |
| rs13382275 | 2 | [SNTG2](http://genome.ucsc.edu/cgi-bin/hgTracks?hgHubConnect.destUrl=..%2Fcgi-bin%2FhgTracks&clade=mammal&org=Human&db=hg19&position=SNTG2) |  |  | G/0.83/0.002/0.4936 | | G/0.827/0.003/0.5676 | G/0.956/0.037/2.57e-05 | G/0.95/0.069/4.11e-06 | A/0.843/0.005/0.06896 | A/0.829/0.001/0.814 |
| rs13009756 | 2 |  | [KCNF1 (52562)](http://genome.ucsc.edu/cgi-bin/hgTracks?hgHubConnect.destUrl=..%2Fcgi-bin%2FhgTracks&clade=mammal&org=Human&db=hg19&position=KCNF1) | [AX746649 (132212)](http://genome.ucsc.edu/cgi-bin/hgTracks?hgHubConnect.destUrl=..%2Fcgi-bin%2FhgTracks&clade=mammal&org=Human&db=hg19&position=AX746649) | G/0.774/0.005/0.3636 | | G/0.771/-0.002/0.7158 | G/0.934/0.032/0.00581 | G/0.932/0.094/3.82e-06 | NA | C/0.771/-0.002/0.7202 |
| rs115455308 | 2 | [CLEC4F](http://genome.ucsc.edu/cgi-bin/hgTracks?hgHubConnect.destUrl=..%2Fcgi-bin%2FhgTracks&clade=mammal&org=Human&db=hg19&position=CLEC4F) | [FIGLA (24643)](http://genome.ucsc.edu/cgi-bin/hgTracks?hgHubConnect.destUrl=..%2Fcgi-bin%2FhgTracks&clade=mammal&org=Human&db=hg19&position=FIGLA) | [CD207 (14924)](http://genome.ucsc.edu/cgi-bin/hgTracks?hgHubConnect.destUrl=..%2Fcgi-bin%2FhgTracks&clade=mammal&org=Human&db=hg19&position=CD207) | C/1/-0.028/0.7298 | | C/1/0.111/0.3361 | C/0.991/0.014/0.452 | C/0.992/0.167/4.41e-06 | T/0.992/0.008/0.6234 | T/0.999/-0.022/0.7054 |
| rs79764963 | 2 |  |  |  | T/0.951/-0.002/0.7921 | | T/0.95/-0.002/0.7997 | T/0.99/0.034/0.07954 | T/0.989/0.159/4.09e-06 | T/0.047/-0.002/0.7589 | T/0.05/-0.002/0.7614 |
| rs180791571 | 2 | [LOC254128](http://genome.ucsc.edu/cgi-bin/hgTracks?hgHubConnect.destUrl=..%2Fcgi-bin%2FhgTracks&clade=mammal&org=Human&db=hg19&position=LOC254128) | [CLASP1 (57443)](http://genome.ucsc.edu/cgi-bin/hgTracks?hgHubConnect.destUrl=..%2Fcgi-bin%2FhgTracks&clade=mammal&org=Human&db=hg19&position=CLASP1) | [MKI67IP (20025)](http://genome.ucsc.edu/cgi-bin/hgTracks?hgHubConnect.destUrl=..%2Fcgi-bin%2FhgTracks&clade=mammal&org=Human&db=hg19&position=MKI67IP) | A/0.993/-0.051/0.03629 | | A/0.994/-0.088/0.01976 | A/0.998/0.01/0.8721 | A/0.999/0.982/7.02e-07 | A/0.007/0.025/0.1946 | A/0.007/0.027/0.2761 |
| rs116165861 | 2 |  |  |  | G/0.995/-0.013/0.5675 | | G/0.995/-0.052/0.07 | G/0.999/0.204/0.00023 | G/0.998/0.385/1.53e-06 | A/0.995/0.021/0.1908 | A/0.994/-0.016/0.3658 |
| rs188074355 | 2 |  |  |  | A/0.998/-0.031/0.4444 | | A/0.998/-0.124/0.01653 | A/1/0.328/1e-04 | A/0.999/0.558/4.15e-06 | A/0.002/-0.041/0.179 | A/0.002/0.057/0.1036 |
| rs140381154 | 2 |  |  |  | G/0.995/-0.008/0.7201 | | G/0.995/-0.053/0.06386 | G/0.999/0.211/0.00017 | G/0.998/0.387/1.28e-06 | A/0.995/0.024/0.1339 | A/0.994/-0.016/0.3599 |
| rs142412198 | 2 |  |  |  | A/0.995/-0.008/0.7093 | | A/0.995/-0.054/0.06114 | A/0.999/0.209/0.00018 | A/0.998/0.388/1.24e-06 | A/0.005/-0.024/0.1403 | A/0.006/0.017/0.3374 |
| rs148581520 | 2 |  |  |  | C/0.995/-0.009/0.6789 | | C/0.995/-0.055/0.0559 | C/0.999/0.209/0.00019 | C/0.998/0.388/1.22e-06 | C/0.005/-0.024/0.142 | C/0.006/0.018/0.3234 |
| rs142793646 | 2 |  |  |  | A/0.995/-0.013/0.5621 | | A/0.995/-0.068/0.02013 | A/0.999/0.212/0.00012 | A/0.998/0.404/3.89e-07 | A/0.005/-0.022/0.1666 | A/0.006/0.021/0.2371 |
| rs114979721 | 2 |  |  |  | A/0.994/-0.018/0.3937 | | A/0.994/-0.069/0.01467 | A/0.998/0.19/0.00027 | A/0.998/0.403/4.59e-07 | A/0.006/-0.017/0.2702 | A/0.007/0.021/0.2211 |
| rs138158254 | 2 |  |  |  | A/0.994/-0.019/0.4008 | | A/0.995/-0.079/0.00755 | A/0.998/0.21/8.57e-05 | A/0.998/0.4/5.26e-07 | A/0.005/-0.019/0.2401 | A/0.006/0.027/0.1454 |
| rs190845920 | 2 |  |  |  | NA | | NA | C/0.999/-0.051/0.6004 | C/1/4.466/2.94e-06 | NA | NA |
| rs148411008 | 2 | [LOC285141](http://genome.ucsc.edu/cgi-bin/hgTracks?hgHubConnect.destUrl=..%2Fcgi-bin%2FhgTracks&clade=mammal&org=Human&db=hg19&position=LOC285141) | [AK023515 (26996)](http://genome.ucsc.edu/cgi-bin/hgTracks?hgHubConnect.destUrl=..%2Fcgi-bin%2FhgTracks&clade=mammal&org=Human&db=hg19&position=AK023515) | [GAD1 (18927)](http://genome.ucsc.edu/cgi-bin/hgTracks?hgHubConnect.destUrl=..%2Fcgi-bin%2FhgTracks&clade=mammal&org=Human&db=hg19&position=GAD1) | NA | | NA | T/0.99/0.045/0.1175 | T/0.991/0.243/3.22e-06 | NA | A/0.934/0.035/0.09185 |
| rs45593035 | 2 |  | [LOC285141 (16014)](http://genome.ucsc.edu/cgi-bin/hgTracks?hgHubConnect.destUrl=..%2Fcgi-bin%2FhgTracks&clade=mammal&org=Human&db=hg19&position=LOC285141) | [GAD1 (1709)](http://genome.ucsc.edu/cgi-bin/hgTracks?hgHubConnect.destUrl=..%2Fcgi-bin%2FhgTracks&clade=mammal&org=Human&db=hg19&position=GAD1) | NA | | NA | A/0.99/0.041/0.1455 | A/0.99/0.242/2.59e-06 | NA | A/0.062/-0.03/0.01426 |
| rs113142635 | 2 |  | [AK125001 (6763)](http://genome.ucsc.edu/cgi-bin/hgTracks?hgHubConnect.destUrl=..%2Fcgi-bin%2FhgTracks&clade=mammal&org=Human&db=hg19&position=AK125001) | [ITGA4 (50575)](http://genome.ucsc.edu/cgi-bin/hgTracks?hgHubConnect.destUrl=..%2Fcgi-bin%2FhgTracks&clade=mammal&org=Human&db=hg19&position=ITGA4) | NA | | NA | C/0.99/0.056/0.00476 | C/0.991/0.165/4.66e-06 | NA | NA |
| rs112377817 | 2 |  | [AK125001 (20825)](http://genome.ucsc.edu/cgi-bin/hgTracks?hgHubConnect.destUrl=..%2Fcgi-bin%2FhgTracks&clade=mammal&org=Human&db=hg19&position=AK125001) | [ITGA4 (36513)](http://genome.ucsc.edu/cgi-bin/hgTracks?hgHubConnect.destUrl=..%2Fcgi-bin%2FhgTracks&clade=mammal&org=Human&db=hg19&position=ITGA4) | NA | | NA | G/0.992/0.058/0.00552 | G/0.992/0.184/1.68e-06 | NA | NA |
| rs138263114 | 2 | [ITGA4](http://genome.ucsc.edu/cgi-bin/hgTracks?hgHubConnect.destUrl=..%2Fcgi-bin%2FhgTracks&clade=mammal&org=Human&db=hg19&position=ITGA4) | [AK125001 (78116)](http://genome.ucsc.edu/cgi-bin/hgTracks?hgHubConnect.destUrl=..%2Fcgi-bin%2FhgTracks&clade=mammal&org=Human&db=hg19&position=AK125001) | [CERKL (59004)](http://genome.ucsc.edu/cgi-bin/hgTracks?hgHubConnect.destUrl=..%2Fcgi-bin%2FhgTracks&clade=mammal&org=Human&db=hg19&position=CERKL) | NA | | NA | G/0.996/0.119/2.12e-05 | G/0.995/0.191/4.6e-06 | NA | NA |
| rs800823 | 2 |  | [TMEFF2 (160768)](http://genome.ucsc.edu/cgi-bin/hgTracks?hgHubConnect.destUrl=..%2Fcgi-bin%2FhgTracks&clade=mammal&org=Human&db=hg19&position=TMEFF2) |  | 0 | | 0 | C/0.978/0.033/0.01606 | C/0.976/0.113/4.02e-06 | 0 | 0 |
| rs10439273 | 2 | [SPAG16](http://genome.ucsc.edu/cgi-bin/hgTracks?hgHubConnect.destUrl=..%2Fcgi-bin%2FhgTracks&clade=mammal&org=Human&db=hg19&position=SPAG16) |  |  | A/0.207/0.003/0.3845 | | A/0.207/0.004/0.424 | A/0.04/0.01/0.2332 | A/0.04/0.069/8.67e-07 | A/0.386/0.001/0.8145 | A/0.521/-0.001/0.7602 |
| rs72946119 | 2 | [SPAG16](http://genome.ucsc.edu/cgi-bin/hgTracks?hgHubConnect.destUrl=..%2Fcgi-bin%2FhgTracks&clade=mammal&org=Human&db=hg19&position=SPAG16) |  |  | T/0.8/-0.004/0.293 | | T/0.802/-0.004/0.3324 | T/0.955/-0.014/0.08663 | T/0.955/-0.074/8e-07 | T/0.185/0/0.9414 | T/0.2/0.001/0.8052 |
| rs72952025 | 2 | [SPAG16](http://genome.ucsc.edu/cgi-bin/hgTracks?hgHubConnect.destUrl=..%2Fcgi-bin%2FhgTracks&clade=mammal&org=Human&db=hg19&position=SPAG16) |  |  | C/0.798/-0.003/0.3772 | | C/0.8/-0.004/0.3888 | C/0.949/-0.015/0.04923 | C/0.951/-0.068/1.25e-06 | T/0.813/0.001/0.7334 | T/0.793/0/0.9655 |
| rs10932534 | 2 | [SPAG16](http://genome.ucsc.edu/cgi-bin/hgTracks?hgHubConnect.destUrl=..%2Fcgi-bin%2FhgTracks&clade=mammal&org=Human&db=hg19&position=SPAG16) |  |  | C/0.853/0.002/0.6736 | | C/0.852/0.001/0.7773 | C/0.292/0.005/0.137 | C/0.282/0.03/4.81e-06 | T/0.628/0.003/0.2409 | T/0.853/0.003/0.3788 |
| rs1912191 | 2 | [SPAG16](http://genome.ucsc.edu/cgi-bin/hgTracks?hgHubConnect.destUrl=..%2Fcgi-bin%2FhgTracks&clade=mammal&org=Human&db=hg19&position=SPAG16) |  |  | A/0.857/0.002/0.6404 | | A/0.856/0.004/0.4503 | A/0.291/0.006/0.1021 | A/0.28/0.03/3.83e-06 | A/0.376/-0.003/0.2151 | A/0.142/-0.004/0.2644 |
| rs1912188 | 2 | [SPAG16](http://genome.ucsc.edu/cgi-bin/hgTracks?hgHubConnect.destUrl=..%2Fcgi-bin%2FhgTracks&clade=mammal&org=Human&db=hg19&position=SPAG16) |  |  | A/0.846/0/0.942 | | A/0.847/0.003/0.5626 | A/0.293/0.006/0.1131 | A/0.285/0.033/1.43e-06 | A/0.375/-0.002/0.2687 | A/0.153/-0.004/0.249 |
| rs13017349 | 2 | [SPAG16](http://genome.ucsc.edu/cgi-bin/hgTracks?hgHubConnect.destUrl=..%2Fcgi-bin%2FhgTracks&clade=mammal&org=Human&db=hg19&position=SPAG16) |  |  | A/0.814/0.003/0.4721 | | A/0.811/0.001/0.7777 | A/0.252/0.01/0.01364 | A/0.236/0.035/1.48e-06 | A/0.394/-0.005/0.04452 | A/0.19/-0.003/0.3111 |
| rs116135467 | 2 |  | [WNT6 (3499)](http://genome.ucsc.edu/cgi-bin/hgTracks?hgHubConnect.destUrl=..%2Fcgi-bin%2FhgTracks&clade=mammal&org=Human&db=hg19&position=WNT6) | [WNT10A (2801)](http://genome.ucsc.edu/cgi-bin/hgTracks?hgHubConnect.destUrl=..%2Fcgi-bin%2FhgTracks&clade=mammal&org=Human&db=hg19&position=WNT10A) | NA | | NA | G/0.992/0.048/0.05608 | G/0.993/0.242/6.66e-07 | NA | NA |
| rs116039626 | 2 |  |  | [AGAP1 (48997)](http://genome.ucsc.edu/cgi-bin/hgTracks?hgHubConnect.destUrl=..%2Fcgi-bin%2FhgTracks&clade=mammal&org=Human&db=hg19&position=AGAP1) | T/0.967/0.006/0.5078 | | T/0.966/-0.001/0.9399 | T/0.99/0.102/4.42e-05 | T/0.99/0.218/1.8e-06 | T/0.03/-0.009/0.2056 | T/0.032/0.009/0.2801 |
| rs191663403 | 3 |  |  |  | NA | | NA | C/0.999/0.425/2.99e-07 | C/0.998/0.43/4.85e-06 | NA | NA |
| rs17236697 | 3 | [PPM1L](http://genome.ucsc.edu/cgi-bin/hgTracks?hgHubConnect.destUrl=..%2Fcgi-bin%2FhgTracks&clade=mammal&org=Human&db=hg19&position=PPM1L) |  | [AK055323 (178220)](http://genome.ucsc.edu/cgi-bin/hgTracks?hgHubConnect.destUrl=..%2Fcgi-bin%2FhgTracks&clade=mammal&org=Human&db=hg19&position=AK055323) | G/0.967/-0.002/0.7797 | | G/0.967/-0.002/0.879 | G/0.987/0.06/0.00029 | G/0.985/0.125/3.1e-06 | A/0.97/0.007/0.1997 | A/0.969/-0.002/0.7407 |
| rs9874264 | 3 |  | [LOC344887 (10835)](http://genome.ucsc.edu/cgi-bin/hgTracks?hgHubConnect.destUrl=..%2Fcgi-bin%2FhgTracks&clade=mammal&org=Human&db=hg19&position=LOC344887) | [ETV5 (54605)](http://genome.ucsc.edu/cgi-bin/hgTracks?hgHubConnect.destUrl=..%2Fcgi-bin%2FhgTracks&clade=mammal&org=Human&db=hg19&position=ETV5) | C/0.971/0.01/0.264 | | C/0.968/-0.007/0.5489 | C/0.899/0.017/0.00628 | C/0.893/0.052/4.82e-06 | T/0.93/0.012/0.01039 | T/0.971/-0.008/0.3546 |
| rs142846286 | 4 |  |  | [FLJ13197 (109584)](http://genome.ucsc.edu/cgi-bin/hgTracks?hgHubConnect.destUrl=..%2Fcgi-bin%2FhgTracks&clade=mammal&org=Human&db=hg19&position=FLJ13197) | NA | | NA | A/0.997/0.065/0.1406 | A/0.998/0.398/3.44e-06 | NA | NA |
| rs140671052 | 4 |  |  | [EF565118 (82560)](http://genome.ucsc.edu/cgi-bin/hgTracks?hgHubConnect.destUrl=..%2Fcgi-bin%2FhgTracks&clade=mammal&org=Human&db=hg19&position=EF565118) | NA | | NA | G/0.996/0.088/0.03573 | G/0.996/0.383/4.5e-06 | NA | NA |
| rs7695246 | 4 | [CFI](http://genome.ucsc.edu/cgi-bin/hgTracks?hgHubConnect.destUrl=..%2Fcgi-bin%2FhgTracks&clade=mammal&org=Human&db=hg19&position=CFI) | [PLA2G12A (37570)](http://genome.ucsc.edu/cgi-bin/hgTracks?hgHubConnect.destUrl=..%2Fcgi-bin%2FhgTracks&clade=mammal&org=Human&db=hg19&position=PLA2G12A) | [GAR1 (47853)](http://genome.ucsc.edu/cgi-bin/hgTracks?hgHubConnect.destUrl=..%2Fcgi-bin%2FhgTracks&clade=mammal&org=Human&db=hg19&position=GAR1) | 0 | | 0 | C/0.706/-0.006/0.1393 | C/0.69/-0.033/4.94e-06 | 0 | A/0.93/-0.024/0.03231 |
| rs139817151 | 5 | [C7](http://genome.ucsc.edu/cgi-bin/hgTracks?hgHubConnect.destUrl=..%2Fcgi-bin%2FhgTracks&clade=mammal&org=Human&db=hg19&position=C7) | [CARD6 (112253)](http://genome.ucsc.edu/cgi-bin/hgTracks?hgHubConnect.destUrl=..%2Fcgi-bin%2FhgTracks&clade=mammal&org=Human&db=hg19&position=CARD6) | [HEATR7B2 (30412)](http://genome.ucsc.edu/cgi-bin/hgTracks?hgHubConnect.destUrl=..%2Fcgi-bin%2FhgTracks&clade=mammal&org=Human&db=hg19&position=HEATR7B2) | NA | | NA | G/0.997/0.067/0.02649 | G/0.997/0.249/4.67e-06 | NA | NA |
| rs78958340 | 5 | [CCL28](http://genome.ucsc.edu/cgi-bin/hgTracks?hgHubConnect.destUrl=..%2Fcgi-bin%2FhgTracks&clade=mammal&org=Human&db=hg19&position=CCL28) | [HMGCS1 (98008)](http://genome.ucsc.edu/cgi-bin/hgTracks?hgHubConnect.destUrl=..%2Fcgi-bin%2FhgTracks&clade=mammal&org=Human&db=hg19&position=HMGCS1) | [C5orf28 (32750)](http://genome.ucsc.edu/cgi-bin/hgTracks?hgHubConnect.destUrl=..%2Fcgi-bin%2FhgTracks&clade=mammal&org=Human&db=hg19&position=C5orf28) | NA | | NA | T/0.963/0.015/0.08781 | T/0.964/0.078/1.43e-06 | NA | NA |
| rs183575223 | 5 |  | [CCL28 (5594)](http://genome.ucsc.edu/cgi-bin/hgTracks?hgHubConnect.destUrl=..%2Fcgi-bin%2FhgTracks&clade=mammal&org=Human&db=hg19&position=CCL28) | [C5orf28 (26271)](http://genome.ucsc.edu/cgi-bin/hgTracks?hgHubConnect.destUrl=..%2Fcgi-bin%2FhgTracks&clade=mammal&org=Human&db=hg19&position=C5orf28) | NA | | NA | T/0.962/0.014/0.1086 | T/0.963/0.074/2.51e-06 | NA | NA |
| rs115681743 | 5 |  | [CCL28 (7654)](http://genome.ucsc.edu/cgi-bin/hgTracks?hgHubConnect.destUrl=..%2Fcgi-bin%2FhgTracks&clade=mammal&org=Human&db=hg19&position=CCL28) | [C5orf28 (24211)](http://genome.ucsc.edu/cgi-bin/hgTracks?hgHubConnect.destUrl=..%2Fcgi-bin%2FhgTracks&clade=mammal&org=Human&db=hg19&position=C5orf28) | NA | | NA | G/0.962/0.013/0.1113 | G/0.963/0.074/2.52e-06 | NA | NA |
| rs78044334 | 5 |  | [CCL28 (7773)](http://genome.ucsc.edu/cgi-bin/hgTracks?hgHubConnect.destUrl=..%2Fcgi-bin%2FhgTracks&clade=mammal&org=Human&db=hg19&position=CCL28) | [C5orf28 (24092)](http://genome.ucsc.edu/cgi-bin/hgTracks?hgHubConnect.destUrl=..%2Fcgi-bin%2FhgTracks&clade=mammal&org=Human&db=hg19&position=C5orf28) | NA | | NA | T/0.962/0.013/0.1114 | T/0.963/0.074/2.52e-06 | NA | NA |
| rs75386498 | 5 |  | [CCL28 (13930)](http://genome.ucsc.edu/cgi-bin/hgTracks?hgHubConnect.destUrl=..%2Fcgi-bin%2FhgTracks&clade=mammal&org=Human&db=hg19&position=CCL28) | [C5orf28 (17935)](http://genome.ucsc.edu/cgi-bin/hgTracks?hgHubConnect.destUrl=..%2Fcgi-bin%2FhgTracks&clade=mammal&org=Human&db=hg19&position=C5orf28) | NA | | NA | A/0.962/0.013/0.1184 | A/0.963/0.074/2.29e-06 | NA | NA |
| rs76640434 | 5 |  | [CCL28 (23255)](http://genome.ucsc.edu/cgi-bin/hgTracks?hgHubConnect.destUrl=..%2Fcgi-bin%2FhgTracks&clade=mammal&org=Human&db=hg19&position=CCL28) | [C5orf28 (8610)](http://genome.ucsc.edu/cgi-bin/hgTracks?hgHubConnect.destUrl=..%2Fcgi-bin%2FhgTracks&clade=mammal&org=Human&db=hg19&position=C5orf28) | A/0.998/0.071/0.01418 | | A/0.997/0.044/0.185 | A/0.953/0.009/0.242 | A/0.956/0.071/1.24e-06 | A/0.043/-0.012/0.08564 | A/0.003/-0.048/0.06199 |
| rs189522918 | 5 |  | [CCL28 (27432)](http://genome.ucsc.edu/cgi-bin/hgTracks?hgHubConnect.destUrl=..%2Fcgi-bin%2FhgTracks&clade=mammal&org=Human&db=hg19&position=CCL28) | [C5orf28 (4433)](http://genome.ucsc.edu/cgi-bin/hgTracks?hgHubConnect.destUrl=..%2Fcgi-bin%2FhgTracks&clade=mammal&org=Human&db=hg19&position=C5orf28) | NA | | NA | T/0.962/0.013/0.1304 | T/0.964/0.075/1.83e-06 | NA | NA |
| rs77194569 | 5 | [C5orf28](http://genome.ucsc.edu/cgi-bin/hgTracks?hgHubConnect.destUrl=..%2Fcgi-bin%2FhgTracks&clade=mammal&org=Human&db=hg19&position=C5orf28) | [CCL28 (35761)](http://genome.ucsc.edu/cgi-bin/hgTracks?hgHubConnect.destUrl=..%2Fcgi-bin%2FhgTracks&clade=mammal&org=Human&db=hg19&position=CCL28) | [C5orf34 (38553)](http://genome.ucsc.edu/cgi-bin/hgTracks?hgHubConnect.destUrl=..%2Fcgi-bin%2FhgTracks&clade=mammal&org=Human&db=hg19&position=C5orf34) | NA | | NA | T/0.962/0.013/0.1306 | T/0.964/0.075/1.81e-06 | NA | NA |
| rs116000348 | 5 | [C5orf28](http://genome.ucsc.edu/cgi-bin/hgTracks?hgHubConnect.destUrl=..%2Fcgi-bin%2FhgTracks&clade=mammal&org=Human&db=hg19&position=C5orf28) | [CCL28 (42072)](http://genome.ucsc.edu/cgi-bin/hgTracks?hgHubConnect.destUrl=..%2Fcgi-bin%2FhgTracks&clade=mammal&org=Human&db=hg19&position=CCL28) | [C5orf34 (32242)](http://genome.ucsc.edu/cgi-bin/hgTracks?hgHubConnect.destUrl=..%2Fcgi-bin%2FhgTracks&clade=mammal&org=Human&db=hg19&position=C5orf34) | NA | | NA | G/0.962/0.013/0.1306 | G/0.964/0.075/1.8e-06 | NA | NA |
| rs78321138 | 5 | [C5orf28](http://genome.ucsc.edu/cgi-bin/hgTracks?hgHubConnect.destUrl=..%2Fcgi-bin%2FhgTracks&clade=mammal&org=Human&db=hg19&position=C5orf28) | [CCL28 (43988)](http://genome.ucsc.edu/cgi-bin/hgTracks?hgHubConnect.destUrl=..%2Fcgi-bin%2FhgTracks&clade=mammal&org=Human&db=hg19&position=CCL28) | [C5orf34 (30326)](http://genome.ucsc.edu/cgi-bin/hgTracks?hgHubConnect.destUrl=..%2Fcgi-bin%2FhgTracks&clade=mammal&org=Human&db=hg19&position=C5orf34) | NA | | NA | A/0.962/0.013/0.1305 | A/0.964/0.075/1.8e-06 | NA | NA |
| rs180834883 | 5 | [C5orf28](http://genome.ucsc.edu/cgi-bin/hgTracks?hgHubConnect.destUrl=..%2Fcgi-bin%2FhgTracks&clade=mammal&org=Human&db=hg19&position=C5orf28) | [CCL28 (52655)](http://genome.ucsc.edu/cgi-bin/hgTracks?hgHubConnect.destUrl=..%2Fcgi-bin%2FhgTracks&clade=mammal&org=Human&db=hg19&position=CCL28) | [C5orf34 (21659)](http://genome.ucsc.edu/cgi-bin/hgTracks?hgHubConnect.destUrl=..%2Fcgi-bin%2FhgTracks&clade=mammal&org=Human&db=hg19&position=C5orf34) | G/0.998/0.071/0.01337 | | G/0.997/0.046/0.1651 | G/0.952/0.008/0.2986 | G/0.956/0.068/2.08e-06 | T/0.956/0.012/0.1043 | T/0.997/0.05/0.04746 |
| rs115252650 | 5 | [C5orf28](http://genome.ucsc.edu/cgi-bin/hgTracks?hgHubConnect.destUrl=..%2Fcgi-bin%2FhgTracks&clade=mammal&org=Human&db=hg19&position=C5orf28) | [CCL28 (58172)](http://genome.ucsc.edu/cgi-bin/hgTracks?hgHubConnect.destUrl=..%2Fcgi-bin%2FhgTracks&clade=mammal&org=Human&db=hg19&position=CCL28) | [C5orf34 (16142)](http://genome.ucsc.edu/cgi-bin/hgTracks?hgHubConnect.destUrl=..%2Fcgi-bin%2FhgTracks&clade=mammal&org=Human&db=hg19&position=C5orf34) | T/0.998/0.071/0.01337 | | T/0.997/0.046/0.165 | T/0.952/0.008/0.2983 | T/0.956/0.068/2.07e-06 | T/0.044/-0.012/0.1044 | T/0.003/-0.05/0.04737 |
| rs76677587 | 5 | [C5orf28](http://genome.ucsc.edu/cgi-bin/hgTracks?hgHubConnect.destUrl=..%2Fcgi-bin%2FhgTracks&clade=mammal&org=Human&db=hg19&position=C5orf28) | [CCL28 (70796)](http://genome.ucsc.edu/cgi-bin/hgTracks?hgHubConnect.destUrl=..%2Fcgi-bin%2FhgTracks&clade=mammal&org=Human&db=hg19&position=CCL28) | [C5orf34 (3518)](http://genome.ucsc.edu/cgi-bin/hgTracks?hgHubConnect.destUrl=..%2Fcgi-bin%2FhgTracks&clade=mammal&org=Human&db=hg19&position=C5orf34) | C/0.998/0.071/0.01351 | | C/0.997/0.046/0.1639 | C/0.952/0.008/0.2971 | C/0.956/0.068/2.06e-06 | T/0.956/0.012/0.1041 | T/0.997/0.05/0.04626 |
| rs79923522 | 5 |  | [C5orf28 (1980)](http://genome.ucsc.edu/cgi-bin/hgTracks?hgHubConnect.destUrl=..%2Fcgi-bin%2FhgTracks&clade=mammal&org=Human&db=hg19&position=C5orf28) | [C5orf34 (830)](http://genome.ucsc.edu/cgi-bin/hgTracks?hgHubConnect.destUrl=..%2Fcgi-bin%2FhgTracks&clade=mammal&org=Human&db=hg19&position=C5orf34) | NA | | NA | A/0.962/0.013/0.1295 | A/0.964/0.075/1.74e-06 | NA | NA |
| rs76881288 | 5 | [C5orf34](http://genome.ucsc.edu/cgi-bin/hgTracks?hgHubConnect.destUrl=..%2Fcgi-bin%2FhgTracks&clade=mammal&org=Human&db=hg19&position=C5orf34) | [C5orf28 (4444)](http://genome.ucsc.edu/cgi-bin/hgTracks?hgHubConnect.destUrl=..%2Fcgi-bin%2FhgTracks&clade=mammal&org=Human&db=hg19&position=C5orf28) | [PAIP1 (37933)](http://genome.ucsc.edu/cgi-bin/hgTracks?hgHubConnect.destUrl=..%2Fcgi-bin%2FhgTracks&clade=mammal&org=Human&db=hg19&position=PAIP1) | G/0.998/0.071/0.01353 | | G/0.997/0.046/0.1638 | G/0.952/0.008/0.2967 | G/0.956/0.068/2.06e-06 | C/0.956/0.012/0.1024 | C/0.997/0.05/0.04365 |
| rs146735629 | 5 | [C5orf34](http://genome.ucsc.edu/cgi-bin/hgTracks?hgHubConnect.destUrl=..%2Fcgi-bin%2FhgTracks&clade=mammal&org=Human&db=hg19&position=C5orf34) | [C5orf28 (7947)](http://genome.ucsc.edu/cgi-bin/hgTracks?hgHubConnect.destUrl=..%2Fcgi-bin%2FhgTracks&clade=mammal&org=Human&db=hg19&position=C5orf28) | [PAIP1 (34430)](http://genome.ucsc.edu/cgi-bin/hgTracks?hgHubConnect.destUrl=..%2Fcgi-bin%2FhgTracks&clade=mammal&org=Human&db=hg19&position=PAIP1) | T/0.998/0.071/0.01352 | | T/0.997/0.046/0.1636 | T/0.952/0.008/0.2967 | T/0.956/0.068/2.06e-06 | T/0.044/-0.012/0.1023 | T/0.003/-0.05/0.04351 |
| rs7712914 | 5 | [C5orf34](http://genome.ucsc.edu/cgi-bin/hgTracks?hgHubConnect.destUrl=..%2Fcgi-bin%2FhgTracks&clade=mammal&org=Human&db=hg19&position=C5orf34) | [C5orf28 (10222)](http://genome.ucsc.edu/cgi-bin/hgTracks?hgHubConnect.destUrl=..%2Fcgi-bin%2FhgTracks&clade=mammal&org=Human&db=hg19&position=C5orf28) | [PAIP1 (32155)](http://genome.ucsc.edu/cgi-bin/hgTracks?hgHubConnect.destUrl=..%2Fcgi-bin%2FhgTracks&clade=mammal&org=Human&db=hg19&position=PAIP1) | NA | | NA | G/0.96/0.011/0.177 | G/0.963/0.075/2.13e-06 | NA | NA |
| rs75401687 | 5 | [C5orf34](http://genome.ucsc.edu/cgi-bin/hgTracks?hgHubConnect.destUrl=..%2Fcgi-bin%2FhgTracks&clade=mammal&org=Human&db=hg19&position=C5orf34) | [C5orf28 (31181)](http://genome.ucsc.edu/cgi-bin/hgTracks?hgHubConnect.destUrl=..%2Fcgi-bin%2FhgTracks&clade=mammal&org=Human&db=hg19&position=C5orf28) | [PAIP1 (11196)](http://genome.ucsc.edu/cgi-bin/hgTracks?hgHubConnect.destUrl=..%2Fcgi-bin%2FhgTracks&clade=mammal&org=Human&db=hg19&position=PAIP1) | NA | | NA | T/0.962/0.013/0.1249 | T/0.964/0.075/1.64e-06 | NA | NA |
| rs79942555 | 5 |  | [C5orf34 (5251)](http://genome.ucsc.edu/cgi-bin/hgTracks?hgHubConnect.destUrl=..%2Fcgi-bin%2FhgTracks&clade=mammal&org=Human&db=hg19&position=C5orf34) | [PAIP1 (5845)](http://genome.ucsc.edu/cgi-bin/hgTracks?hgHubConnect.destUrl=..%2Fcgi-bin%2FhgTracks&clade=mammal&org=Human&db=hg19&position=PAIP1) | NA | | NA | T/0.962/0.013/0.1244 | T/0.964/0.075/1.64e-06 | NA | NA |
| rs76396004 | 5 |  | [C5orf34 (9286)](http://genome.ucsc.edu/cgi-bin/hgTracks?hgHubConnect.destUrl=..%2Fcgi-bin%2FhgTracks&clade=mammal&org=Human&db=hg19&position=C5orf34) | [PAIP1 (1810)](http://genome.ucsc.edu/cgi-bin/hgTracks?hgHubConnect.destUrl=..%2Fcgi-bin%2FhgTracks&clade=mammal&org=Human&db=hg19&position=PAIP1) | T/0.998/0.07/0.01386 | | T/0.997/0.046/0.16 | T/0.952/0.008/0.2869 | T/0.956/0.068/2.01e-06 | T/0.044/-0.012/0.09663 | T/0.003/-0.051/0.04116 |
| rs141017100 | 5 | [PAIP1](http://genome.ucsc.edu/cgi-bin/hgTracks?hgHubConnect.destUrl=..%2Fcgi-bin%2FhgTracks&clade=mammal&org=Human&db=hg19&position=PAIP1) | [C5orf34 (16416)](http://genome.ucsc.edu/cgi-bin/hgTracks?hgHubConnect.destUrl=..%2Fcgi-bin%2FhgTracks&clade=mammal&org=Human&db=hg19&position=C5orf34) | [AK096668 (46054)](http://genome.ucsc.edu/cgi-bin/hgTracks?hgHubConnect.destUrl=..%2Fcgi-bin%2FhgTracks&clade=mammal&org=Human&db=hg19&position=AK096668) | NA | | NA | T/0.962/0.013/0.1178 | T/0.963/0.075/1.58e-06 | NA | NA |
| rs115378923 | 5 | [PAIP1](http://genome.ucsc.edu/cgi-bin/hgTracks?hgHubConnect.destUrl=..%2Fcgi-bin%2FhgTracks&clade=mammal&org=Human&db=hg19&position=PAIP1) | [C5orf34 (33415)](http://genome.ucsc.edu/cgi-bin/hgTracks?hgHubConnect.destUrl=..%2Fcgi-bin%2FhgTracks&clade=mammal&org=Human&db=hg19&position=C5orf34) | [AK096668 (29055)](http://genome.ucsc.edu/cgi-bin/hgTracks?hgHubConnect.destUrl=..%2Fcgi-bin%2FhgTracks&clade=mammal&org=Human&db=hg19&position=AK096668) | NA | | NA | T/0.962/0.013/0.1111 | T/0.963/0.075/1.51e-06 | NA | NA |
| rs116695782 | 5 | [PAIP1](http://genome.ucsc.edu/cgi-bin/hgTracks?hgHubConnect.destUrl=..%2Fcgi-bin%2FhgTracks&clade=mammal&org=Human&db=hg19&position=PAIP1) | [C5orf34 (36561)](http://genome.ucsc.edu/cgi-bin/hgTracks?hgHubConnect.destUrl=..%2Fcgi-bin%2FhgTracks&clade=mammal&org=Human&db=hg19&position=C5orf34) | [AK096668 (25909)](http://genome.ucsc.edu/cgi-bin/hgTracks?hgHubConnect.destUrl=..%2Fcgi-bin%2FhgTracks&clade=mammal&org=Human&db=hg19&position=AK096668) | NA | | NA | A/0.962/0.013/0.1109 | A/0.963/0.075/1.51e-06 | NA | NA |
| rs79481971 | 5 | [PAIP1](http://genome.ucsc.edu/cgi-bin/hgTracks?hgHubConnect.destUrl=..%2Fcgi-bin%2FhgTracks&clade=mammal&org=Human&db=hg19&position=PAIP1) | [C5orf34 (38846)](http://genome.ucsc.edu/cgi-bin/hgTracks?hgHubConnect.destUrl=..%2Fcgi-bin%2FhgTracks&clade=mammal&org=Human&db=hg19&position=C5orf34) | [AK096668 (23624)](http://genome.ucsc.edu/cgi-bin/hgTracks?hgHubConnect.destUrl=..%2Fcgi-bin%2FhgTracks&clade=mammal&org=Human&db=hg19&position=AK096668) | NA | | NA | C/0.962/0.013/0.1108 | C/0.963/0.075/1.51e-06 | NA | NA |
| rs77683199 | 5 |  | [PAIP1 (931)](http://genome.ucsc.edu/cgi-bin/hgTracks?hgHubConnect.destUrl=..%2Fcgi-bin%2FhgTracks&clade=mammal&org=Human&db=hg19&position=PAIP1) | [AK096668 (19291)](http://genome.ucsc.edu/cgi-bin/hgTracks?hgHubConnect.destUrl=..%2Fcgi-bin%2FhgTracks&clade=mammal&org=Human&db=hg19&position=AK096668) | NA | | NA | A/0.962/0.013/0.1105 | A/0.963/0.075/1.51e-06 | NA | NA |
| rs114512566 | 5 |  | [PAIP1 (5916)](http://genome.ucsc.edu/cgi-bin/hgTracks?hgHubConnect.destUrl=..%2Fcgi-bin%2FhgTracks&clade=mammal&org=Human&db=hg19&position=PAIP1) | [AK096668 (14306)](http://genome.ucsc.edu/cgi-bin/hgTracks?hgHubConnect.destUrl=..%2Fcgi-bin%2FhgTracks&clade=mammal&org=Human&db=hg19&position=AK096668) | NA | | NA | C/0.962/0.013/0.1161 | C/0.963/0.075/1.48e-06 | NA | NA |
| rs141238427 | 5 |  | [PAIP1 (10071)](http://genome.ucsc.edu/cgi-bin/hgTracks?hgHubConnect.destUrl=..%2Fcgi-bin%2FhgTracks&clade=mammal&org=Human&db=hg19&position=PAIP1) | [AK096668 (10151)](http://genome.ucsc.edu/cgi-bin/hgTracks?hgHubConnect.destUrl=..%2Fcgi-bin%2FhgTracks&clade=mammal&org=Human&db=hg19&position=AK096668) | A/0.998/0.07/0.0145 | | A/0.997/0.048/0.1481 | A/0.952/0.008/0.2708 | A/0.955/0.067/3.24e-06 | A/0.044/-0.012/0.0895 | A/0.003/-0.051/0.03727 |
| rs115426610 | 5 |  | [PAIP1 (10596)](http://genome.ucsc.edu/cgi-bin/hgTracks?hgHubConnect.destUrl=..%2Fcgi-bin%2FhgTracks&clade=mammal&org=Human&db=hg19&position=PAIP1) | [AK096668 (9626)](http://genome.ucsc.edu/cgi-bin/hgTracks?hgHubConnect.destUrl=..%2Fcgi-bin%2FhgTracks&clade=mammal&org=Human&db=hg19&position=AK096668) | T/0.998/0.07/0.01448 | | T/0.997/0.048/0.1478 | T/0.952/0.008/0.271 | T/0.955/0.067/3.27e-06 | A/0.956/0.012/0.092 | A/0.997/0.052/0.03706 |
| rs78646877 | 5 |  | [PAIP1 (12381)](http://genome.ucsc.edu/cgi-bin/hgTracks?hgHubConnect.destUrl=..%2Fcgi-bin%2FhgTracks&clade=mammal&org=Human&db=hg19&position=PAIP1) | [AK096668 (7841)](http://genome.ucsc.edu/cgi-bin/hgTracks?hgHubConnect.destUrl=..%2Fcgi-bin%2FhgTracks&clade=mammal&org=Human&db=hg19&position=AK096668) | G/0.998/0.07/0.01453 | | G/0.997/0.048/0.1472 | G/0.952/0.008/0.2717 | G/0.955/0.067/3.36e-06 | A/0.956/0.012/0.09231 | A/0.997/0.052/0.03706 |
| rs116448453 | 5 |  | [PAIP1 (15613)](http://genome.ucsc.edu/cgi-bin/hgTracks?hgHubConnect.destUrl=..%2Fcgi-bin%2FhgTracks&clade=mammal&org=Human&db=hg19&position=PAIP1) | [AK096668 (4609)](http://genome.ucsc.edu/cgi-bin/hgTracks?hgHubConnect.destUrl=..%2Fcgi-bin%2FhgTracks&clade=mammal&org=Human&db=hg19&position=AK096668) | NA | | NA | T/0.962/0.013/0.1179 | T/0.963/0.075/1.48e-06 | NA | NA |
| rs115648704 | 5 | [AK096668](http://genome.ucsc.edu/cgi-bin/hgTracks?hgHubConnect.destUrl=..%2Fcgi-bin%2FhgTracks&clade=mammal&org=Human&db=hg19&position=AK096668) | [PAIP1 (30307)](http://genome.ucsc.edu/cgi-bin/hgTracks?hgHubConnect.destUrl=..%2Fcgi-bin%2FhgTracks&clade=mammal&org=Human&db=hg19&position=PAIP1) | [NNT (14962)](http://genome.ucsc.edu/cgi-bin/hgTracks?hgHubConnect.destUrl=..%2Fcgi-bin%2FhgTracks&clade=mammal&org=Human&db=hg19&position=NNT) | NA | | NA | G/0.962/0.013/0.118 | G/0.963/0.075/1.47e-06 | NA | NA |
| rs114955764 | 5 | [AK096668](http://genome.ucsc.edu/cgi-bin/hgTracks?hgHubConnect.destUrl=..%2Fcgi-bin%2FhgTracks&clade=mammal&org=Human&db=hg19&position=AK096668) | [PAIP1 (31320)](http://genome.ucsc.edu/cgi-bin/hgTracks?hgHubConnect.destUrl=..%2Fcgi-bin%2FhgTracks&clade=mammal&org=Human&db=hg19&position=PAIP1) | [NNT (13949)](http://genome.ucsc.edu/cgi-bin/hgTracks?hgHubConnect.destUrl=..%2Fcgi-bin%2FhgTracks&clade=mammal&org=Human&db=hg19&position=NNT) | NA | | NA | A/0.962/0.013/0.118 | A/0.963/0.075/1.47e-06 | NA | NA |
| rs76517075 | 5 | [AK096668](http://genome.ucsc.edu/cgi-bin/hgTracks?hgHubConnect.destUrl=..%2Fcgi-bin%2FhgTracks&clade=mammal&org=Human&db=hg19&position=AK096668) | [PAIP1 (32987)](http://genome.ucsc.edu/cgi-bin/hgTracks?hgHubConnect.destUrl=..%2Fcgi-bin%2FhgTracks&clade=mammal&org=Human&db=hg19&position=PAIP1) | [NNT (12282)](http://genome.ucsc.edu/cgi-bin/hgTracks?hgHubConnect.destUrl=..%2Fcgi-bin%2FhgTracks&clade=mammal&org=Human&db=hg19&position=NNT) | NA | | NA | T/0.962/0.013/0.118 | T/0.963/0.075/1.47e-06 | NA | NA |
| rs79482941 | 5 | [AK096668](http://genome.ucsc.edu/cgi-bin/hgTracks?hgHubConnect.destUrl=..%2Fcgi-bin%2FhgTracks&clade=mammal&org=Human&db=hg19&position=AK096668) | [PAIP1 (37070)](http://genome.ucsc.edu/cgi-bin/hgTracks?hgHubConnect.destUrl=..%2Fcgi-bin%2FhgTracks&clade=mammal&org=Human&db=hg19&position=PAIP1) | [NNT (8199)](http://genome.ucsc.edu/cgi-bin/hgTracks?hgHubConnect.destUrl=..%2Fcgi-bin%2FhgTracks&clade=mammal&org=Human&db=hg19&position=NNT) | NA | | NA | T/0.962/0.013/0.1179 | T/0.963/0.075/1.48e-06 | NA | NA |
| rs79548306 | 5 | [AK096668](http://genome.ucsc.edu/cgi-bin/hgTracks?hgHubConnect.destUrl=..%2Fcgi-bin%2FhgTracks&clade=mammal&org=Human&db=hg19&position=AK096668) | [PAIP1 (38824)](http://genome.ucsc.edu/cgi-bin/hgTracks?hgHubConnect.destUrl=..%2Fcgi-bin%2FhgTracks&clade=mammal&org=Human&db=hg19&position=PAIP1) | [NNT (6445)](http://genome.ucsc.edu/cgi-bin/hgTracks?hgHubConnect.destUrl=..%2Fcgi-bin%2FhgTracks&clade=mammal&org=Human&db=hg19&position=NNT) | NA | | NA | A/0.962/0.013/0.1178 | A/0.963/0.075/1.47e-06 | NA | NA |
| rs77333560 | 5 | [AK096668](http://genome.ucsc.edu/cgi-bin/hgTracks?hgHubConnect.destUrl=..%2Fcgi-bin%2FhgTracks&clade=mammal&org=Human&db=hg19&position=AK096668) | [PAIP1 (38825)](http://genome.ucsc.edu/cgi-bin/hgTracks?hgHubConnect.destUrl=..%2Fcgi-bin%2FhgTracks&clade=mammal&org=Human&db=hg19&position=PAIP1) | [NNT (6444)](http://genome.ucsc.edu/cgi-bin/hgTracks?hgHubConnect.destUrl=..%2Fcgi-bin%2FhgTracks&clade=mammal&org=Human&db=hg19&position=NNT) | NA | | NA | T/0.962/0.013/0.1178 | T/0.963/0.075/1.47e-06 | NA | NA |
| rs114176814 | 5 | [AK096668](http://genome.ucsc.edu/cgi-bin/hgTracks?hgHubConnect.destUrl=..%2Fcgi-bin%2FhgTracks&clade=mammal&org=Human&db=hg19&position=AK096668) | [PAIP1 (43455)](http://genome.ucsc.edu/cgi-bin/hgTracks?hgHubConnect.destUrl=..%2Fcgi-bin%2FhgTracks&clade=mammal&org=Human&db=hg19&position=PAIP1) | [NNT (1814)](http://genome.ucsc.edu/cgi-bin/hgTracks?hgHubConnect.destUrl=..%2Fcgi-bin%2FhgTracks&clade=mammal&org=Human&db=hg19&position=NNT) | NA | | NA | T/0.961/0.013/0.1153 | T/0.963/0.075/1.45e-06 | NA | NA |
| rs115760645 | 5 | [NNT](http://genome.ucsc.edu/cgi-bin/hgTracks?hgHubConnect.destUrl=..%2Fcgi-bin%2FhgTracks&clade=mammal&org=Human&db=hg19&position=NNT) | [AK096668 (40060)](http://genome.ucsc.edu/cgi-bin/hgTracks?hgHubConnect.destUrl=..%2Fcgi-bin%2FhgTracks&clade=mammal&org=Human&db=hg19&position=AK096668) |  | NA | | NA | G/0.962/0.013/0.1183 | G/0.963/0.075/1.46e-06 | NA | NA |
| rs74863720 | 5 | [NNT](http://genome.ucsc.edu/cgi-bin/hgTracks?hgHubConnect.destUrl=..%2Fcgi-bin%2FhgTracks&clade=mammal&org=Human&db=hg19&position=NNT) | [AK096668 (46440)](http://genome.ucsc.edu/cgi-bin/hgTracks?hgHubConnect.destUrl=..%2Fcgi-bin%2FhgTracks&clade=mammal&org=Human&db=hg19&position=AK096668) |  | NA | | NA | A/0.962/0.013/0.1212 | A/0.963/0.075/1.45e-06 | NA | NA |
| rs192225282 | 5 | [NNT](http://genome.ucsc.edu/cgi-bin/hgTracks?hgHubConnect.destUrl=..%2Fcgi-bin%2FhgTracks&clade=mammal&org=Human&db=hg19&position=NNT) | [AK096668 (69798)](http://genome.ucsc.edu/cgi-bin/hgTracks?hgHubConnect.destUrl=..%2Fcgi-bin%2FhgTracks&clade=mammal&org=Human&db=hg19&position=AK096668) |  | NA | | NA | A/0.959/0.013/0.1275 | A/0.961/0.074/1.83e-06 | NA | NA |
| rs75999006 | 5 | [NNT](http://genome.ucsc.edu/cgi-bin/hgTracks?hgHubConnect.destUrl=..%2Fcgi-bin%2FhgTracks&clade=mammal&org=Human&db=hg19&position=NNT) | [AK096668 (74588)](http://genome.ucsc.edu/cgi-bin/hgTracks?hgHubConnect.destUrl=..%2Fcgi-bin%2FhgTracks&clade=mammal&org=Human&db=hg19&position=AK096668) |  | NA | | NA | A/0.961/0.013/0.1247 | A/0.963/0.075/1.48e-06 | NA | NA |
| rs7709284 | 5 | [NNT](http://genome.ucsc.edu/cgi-bin/hgTracks?hgHubConnect.destUrl=..%2Fcgi-bin%2FhgTracks&clade=mammal&org=Human&db=hg19&position=NNT) | [AK096668 (85758)](http://genome.ucsc.edu/cgi-bin/hgTracks?hgHubConnect.destUrl=..%2Fcgi-bin%2FhgTracks&clade=mammal&org=Human&db=hg19&position=AK096668) |  | 0 | | 0 | G/0.961/0.013/0.1248 | G/0.963/0.075/1.48e-06 | 0 | 0 |
| rs150379724 | 5 |  | [NNT (16887)](http://genome.ucsc.edu/cgi-bin/hgTracks?hgHubConnect.destUrl=..%2Fcgi-bin%2FhgTracks&clade=mammal&org=Human&db=hg19&position=NNT) |  | NA | | NA | T/0.963/0.013/0.1195 | T/0.965/0.08/6.93e-07 | NA | NA |
| rs76434282 | 5 |  | [NNT (27238)](http://genome.ucsc.edu/cgi-bin/hgTracks?hgHubConnect.destUrl=..%2Fcgi-bin%2FhgTracks&clade=mammal&org=Human&db=hg19&position=NNT) |  | NA | | NA | A/0.962/0.012/0.1507 | A/0.964/0.08/6.53e-07 | NA | NA |
| rs186211928 | 5 |  | [NNT (35885)](http://genome.ucsc.edu/cgi-bin/hgTracks?hgHubConnect.destUrl=..%2Fcgi-bin%2FhgTracks&clade=mammal&org=Human&db=hg19&position=NNT) |  | NA | | NA | C/0.963/0.013/0.12 | C/0.965/0.081/4.64e-07 | NA | NA |
| rs144585579 | 5 |  | [NNT (38477)](http://genome.ucsc.edu/cgi-bin/hgTracks?hgHubConnect.destUrl=..%2Fcgi-bin%2FhgTracks&clade=mammal&org=Human&db=hg19&position=NNT) |  | NA | | NA | G/0.963/0.013/0.1223 | G/0.965/0.081/5.06e-07 | NA | NA |
| rs191814357 | 5 |  | [NNT (39754)](http://genome.ucsc.edu/cgi-bin/hgTracks?hgHubConnect.destUrl=..%2Fcgi-bin%2FhgTracks&clade=mammal&org=Human&db=hg19&position=NNT) |  | NA | | NA | A/0.963/0.013/0.1222 | A/0.965/0.081/5.08e-07 | NA | NA |
| rs185720825 | 5 |  | [NNT (40094)](http://genome.ucsc.edu/cgi-bin/hgTracks?hgHubConnect.destUrl=..%2Fcgi-bin%2FhgTracks&clade=mammal&org=Human&db=hg19&position=NNT) |  | NA | | NA | T/0.963/0.013/0.1221 | T/0.965/0.081/5.11e-07 | NA | NA |
| rs140263876 | 5 |  | [NNT (40876)](http://genome.ucsc.edu/cgi-bin/hgTracks?hgHubConnect.destUrl=..%2Fcgi-bin%2FhgTracks&clade=mammal&org=Human&db=hg19&position=NNT) |  | NA | | NA | A/0.963/0.013/0.1222 | A/0.965/0.081/5.17e-07 | NA | NA |
| rs151142406 | 5 |  | [NNT (46098)](http://genome.ucsc.edu/cgi-bin/hgTracks?hgHubConnect.destUrl=..%2Fcgi-bin%2FhgTracks&clade=mammal&org=Human&db=hg19&position=NNT) |  | NA | | NA | T/0.963/0.013/0.1224 | T/0.965/0.081/5.43e-07 | NA | NA |
| rs114693035 | 5 |  | [NNT (48050)](http://genome.ucsc.edu/cgi-bin/hgTracks?hgHubConnect.destUrl=..%2Fcgi-bin%2FhgTracks&clade=mammal&org=Human&db=hg19&position=NNT) |  | NA | | NA | A/0.963/0.013/0.1226 | A/0.965/0.081/5.54e-07 | NA | NA |
[truncated: 1,745,507 more chars]
